# Supplementary material for: Accessing chiral sulfones bearing quaternary carbon stereocenters via photoinduced radical sulfur dioxide insertion and Truce–Smiles rearrangement
Source: Nat Commun. 2022 Nov 18;13:7081. doi: 10.1038/s41467-022-34836-y (PMC9674831; doi:10.1038/s41467-022-34836-y)
Supplement: Supplementary file 1 — Supplementary Information [file 41467_2022_34836_MOESM1_ESM.pdf]

Supplementary Information

Accessing chiral sulfones bearing quaternary carbon stereocenters via photoinduced radical sulfur dioxide insertion and Truce-Smiles rearrangement

Jiapian Huang[1,#], Fei Liu[1,#], Ling-Hui Zeng[2], Shaoyu Li[1], Zhiyuan Chen[2] & Jie Wu[1,3,4]

[1]School of Pharmaceutical and Materials Engineering &Institute for Advanced Studies, Taizhou University, Taizhou 318000, China.
[2]School of Medicine, Zhejiang University City College, Hangzhou 310015, China.
[3]State Key Laboratory of Organometallic Chemistry, Shanghai Institute of Organic Chemistry, Chinese Academy of Sciences, Shanghai 200032, China.
[4]School of Chemistry and Chemical Engineering, Henan Normal University, Xinxiang 453007, China.
[#]These authors contributed equally to this work.

Email: lisy@tzc.edu.cn; chenzy@zucc.edu.cn; jie_wu@fudan.edu.cn.

Table of Contents

1 Supplementary Notes 2

2 Supplementary Methods 3

2.1 General procedures for the preparation of products **4** 3
2.2 Optimization of the reaction conditions 4
2.3. Mechanistic studies 5
2.3.1 Radical trapping experiments 5
2.3.2 Measurement of quantum yield 6
2.3.3 Stern-Volmer fluorescence quenching experiments. 7
2.3.4 Plausible mechanism of thianthrenium salts system 9
2.4 Synthesis procedures and characterization data of substrates **1** 10
2.5 Characterization data of products **4** 19
2.6 Crystal data and structure refinement for **4p** 64

3 Supplementary Figures 72

3.1 NMR Spectra of substrates **1** 72
3.2 NMR Spectra of products **4** 76

4 Supplementary References 114

1 Supplementary Notes

Chemicals were purchased from commercial suppliers and used without further purification unless otherwise stated. Analytical thin layer chromatography (TLC) was performed on precoated silica gel 60 GF254 plates. Flash column chromatography was performed using Tsingdao silica gel (60, particle size 0.040-0.063 mm). Visualization on TLC was achieved by use of UV light (254 nm). ^{1}H and ^{13}C NMR spectra were recorded on Bruker 400 MHz spectrometer in $CDCl_3$ with tetramethylsilane (TMS) as internal standard. The chemical shifts are expressed in ppm and coupling constants are given in Hz. Data for ^{1}H NMR are recorded as follows: chemical shift (δ, ppm), multiplicity (s = singlet; d = doublet; t = triplet; q = quartet; p = pentet; m = multiplet; brs = broad singlet), coupling constant (Hz), integration. Data for ^{13}C NMR are reported in terms of chemical shift (δ, ppm). The enantiomeric excess values were determined by chiral HPLC with an Shimadzu instrument and a Daicel CHIRALCEL and CHIRALPAK column. High resolution mass spectroscopy (HRMS) analyses were performed at a Q-Exactive (Thermo Scientific) Inc mass instrument (HESI).

2 Supplementary Methods

2.1 General procedures for the preparation of products 4

Set-up of the photoredox reaction: All photoredox reactions were carried out in the apparatus shown below.

Light source: 35 W LED strip, Greethink (Manufacturer), GT-5050-Blue (Model)

Wavelength of peak intensity: 460-470 nm

Material of the irradiation vessel: borosilicate glass

Distance of the irradiation vessel from the light source: approximately 3 cm.

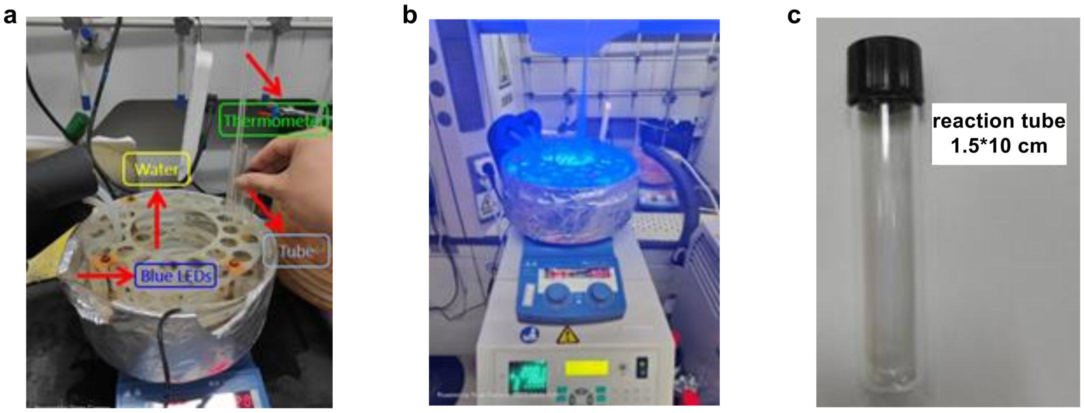

Supplementary Figure 1. Set-up of the photoredox reaction: **a** reaction device, **b** light source, **c** reaction tube.

Method A: aryldiazonium tetrafluoroborates system

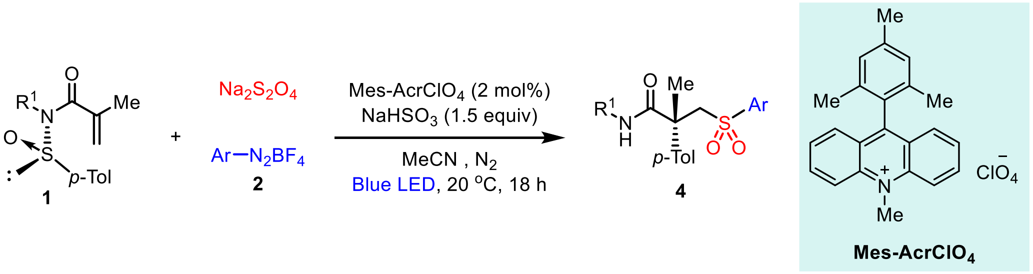

In a glove box, a dry quartz vial equipped with a magnetic stir bar is charged sequentially with **1** (0.2 mmol), **2** (0.4 mmol), $Na_2S_2O_4$ (0.4 mmol), $NaHSO_3$ (0.3 mmol), Mes-AcrClO_4 (2 mol%), and dry MeCN (3.0 mL). The reaction mixture is stirred for 18 h at 900 rpm in a thermostatic water bath at 20 degrees under a 35 W blue LED light. When the reaction is completed (monitored by TLC), the mixture is purified by flash chromatography on silica gel eluted with PE/EA (5/1) to afford the corresponding products.

Method B: thianthrenium salts system

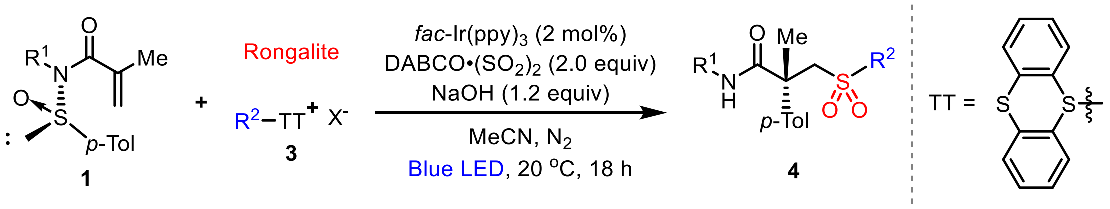

In a glove box, a dry quartz vial equipped with a magnetic stir bar is charged sequentially with **1** (0.2 mmol), **3** (0.4 mmol), Rongalite (0.24 mmol), DABSO (0.4 mmol), NaOH (0.24 mmol), *fac*-Ir(ppy)$_3$ (2 mol%) and dry MeCN (3.0 mL). The reaction mixture is stirred for 18 h at 900 rpm in a thermostatic water bath at 20 degrees under a 35 W blue LED light. When the reaction is completed (monitored by TLC), the mixture is purified by flash chromatography on silica gel eluted with PE/EA (5/1) to afford the corresponding products.

2.2 Optimization of the reaction conditions

Supplementary Table 1. Optimization of the reaction conditions in terms of thianthrenium salts (Method B).[a]

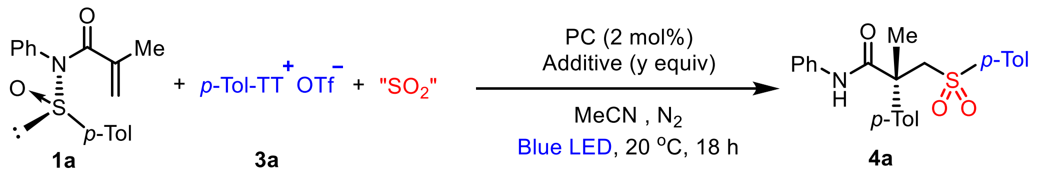

Entry	PC	"SO_2" (x equiv)	Additive (y equiv)	Yield[b] (%)	Ee[c] (%)
1	Mes-AcrClO$_4$	$Na_2S_2O_4$ (2.0)	$NaHSO_3$ (1.5)	trace	/
2	*fac*-Ir(ppy)$_3$	$Na_2S_2O_4$ (2.0)	$NaHSO_3$ (1.5)	43	93
3	*fac*-Ir(ppy)$_3$	DABSO (2.0)	/	38	94
4	*fac*-Ir(ppy)$_3$	$K_2S_2O_5$ (2.0)	/	29	94
5	*fac*-Ir(ppy)$_3$	$NaHSO_3$ (2.0)	/	trace	/
6	*fac*-Ir(ppy)$_3$	Rongalite (2.0)	NaOH (2.0)	47	94
7	*fac*-Ir(ppy)$_3$	Rongalite (2.0)	NaOH (2.0) + $NaHSO_3$ (1.5)	61	94
8	*fac*-Ir(ppy)$_3$	Rongalite (2.0)	NaOH (2.0) + Na_2SO_3 (1.5)	43	94
9	*fac*-Ir(ppy)$_3$	Rongalite (2.0)	NaOH (2.0) + DABSO (1.5)	66	96
10	*fac*-Ir(ppy)$_3$	Rongalite (3.0)	NaOH (3.0) + DABSO (1.5)	69	96
11	*fac*-Ir(ppy)$_3$	Rongalite (1.5)	NaOH (1.5) + DABSO (1.5)	68	97
12	*fac*-Ir(ppy)$_3$	Rongalite (1.2)	NaOH (1.2) + DABSO (1.5)	69	97
13	*fac*-Ir(ppy)$_3$	Rongalite (1.2)	NaOH (1.2) + DABSO (1.0)	67	97
14	*fac*-Ir(ppy)$_3$	Rongalite (1.2)	NaOH (1.2) + DABSO (2.0)	75	97
15	*fac*-Ir(ppy)$_3$	Rongalite (1.2)	NaOH (1.2) + DABSO (3.0)	70	97
16	/	Rongalite (1.2)	NaOH (1.2) + DABSO (2.0)	0	/
17[d]	*fac*-Ir(ppy)$_3$	Rongalite (1.2)	NaOH (1.2) + DABSO (3.0)	0	/

[a] Reaction conditions: **1a** (0.1 mmol), **3a** (0.2 mmol), "SO_2" source (x mmol), photocatalyst (2 mol%), additive (y equiv), solvent (1.5 mL), 35 W blue LED, under N_2 at 20 °C for 18 h. [b] Determined by ^{1}H NMR analysis using 1,3,5-trimethoxybenzene as an internal standard. [c] Determined by HPLC analysis. [d] In the dark.

2.3. Mechanistic studies

2.3.1 Radical trapping experiments

Supplementary Table 2. Radical trapping experiments

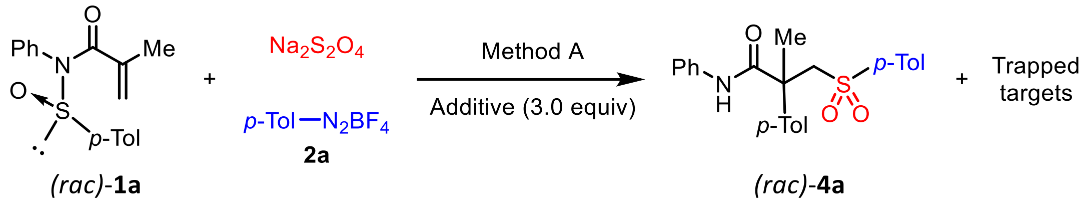

Entry	Additive	*(rac)*-**4a**	Targets
1	TEMPO	0	0
2	BHT	35%	0
3	1,1-Diphenylethylene	0	**5**, 39%

The synthesis of 4a is carried out under standard conditions with the addition of a radical scavenger (3.0 equiv). When the reaction is completed (monitored by TLC), the mixture is purified by flash chromatography on silica gel to afford the product **4a** and trapped targets. The compound structure is determined by ^{1}H NMR analysis using 1,3,5-trimethoxybenzene as an internal standard.

(2-Tosylethene-1,1-diyl)dibenzene (5)

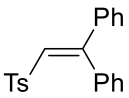

^{1}H NMR (400 MHz, $CDCl_3$) δ 7.47 (d, *J* = 8.2 Hz, 2H), 7.39 – 7.34 (m, 2H), 7.32 – 7.28 (m, 4H), 7.21 – 7.19 (m, 2H), 7.14 (d, *J* = 8.4 Hz, 2H), 7.10 – 7.08 (m, 2H), 6.99 (s, 1H), 2.37 (s, 3H).

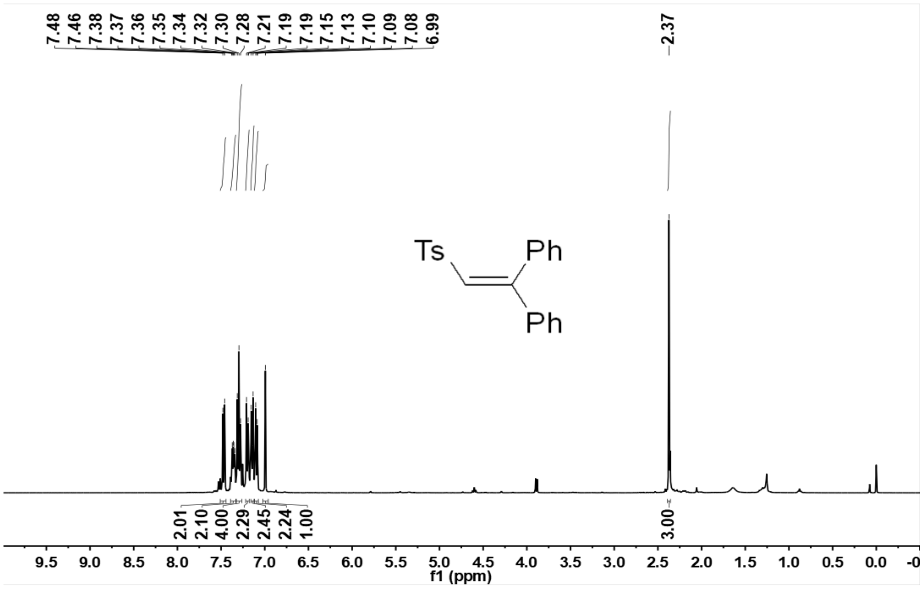

Supplementary Figure 2. ^{1}H NMR-spectrum of **5**, recorded at 400 MHz and 25 °C in $CDCl_3$.

2.3.2 Measurement of quantum yield

The experimental procedures were referred to the published paper.[1] Determination of photon flux of LED blue light by standard potassium ferric oxalate photometric method. A 0.15 M solution of ferrioxalate was prepared by dissolving potassium ferrioxalate hydrate (328mg) in 5 mL of H_2SO_4 (0.20 M) solution. A buffered solution of 1,10-phenanthroline was prepared by dissolving 1,10- phenanthroline (54.1 mg) and sodium acetate (1.23 g) in 20 mL of H_2SO_4 (0.20 M) solution. To determine the photon flux of the LEDs, the ferrioxalate solution (2.0 mL) was placed in a cuvette and irradiated for 90 s at λ_{max} = 420 nm. After irradiation, the phenanthroline solution (0.35 mL) was added to the cuvette, and the mixture was allowed to stir in the dark for 1 h to allow the ferrous ions to completely coordinate with phenanthroline. The absorbance of solution was measured at 510 nm. A non-irradiated sample was also prepared and the absorbance at 510 nm was measured. The calculation method of the amount of ferrous ions generated is as follows:

$$n\ Fe^{2+} = \frac{V \times \Delta A}{l \times \varepsilon}$$

where V is the total volume (0.00235L) of the measurement sample, ΔA is the difference in absorbance at 510 nm between the irradiated and non-irradiated solutions [ΔA = 2.503, l is the optical path of the sample in the spectrophotometer (1 cm), and ε is the extinction coefficient of the complex $Fe^{II}(phen)_3^{2+}$ at 510 nm (11100 L mol^{-1} cm $^{-1}$)

$$photon\ flux = \frac{n\ Fe^{2+}}{\Phi \times t \times f}$$

where Φ is the quantum yield for the ferrioxalate actinometer (1.12 at λ_{ex} = 420 nm), t is the irradiation time (90 s), and f is the fraction of light absorbed at λ_{ex} = 420 nm by the ferrioxalate actinometer.

$$f = 1\text{-}10^{-A\,(420nm)} = 1\text{-}10^{-4.952} = 0.999$$

$$n\ Fe^{2+} = \frac{V \times \Delta A}{l \times \varepsilon} = \frac{0.00235L \times 2.503}{1cm \times 11100L\ mol^{-1}cm^{-1}} = 5.299 \times 10^{-7}\ \text{mol}$$

$$photon\ flux = \frac{n\ Fe^{2+}}{\Phi \times t \times f} = \frac{5.299 \times 10^{-7}}{1.12 \times 90 \times 0.999} = 5.26 \times 10^{-9}\ \text{einstein} \cdot \text{s}^{-1}$$

Method A: aryldiazonium tetrafluoroborates system

Under nitrogen, a dry quartz vial equipped with a magnetic stir bar was charged with **1a** (0.1 mmol), **2a** (0.2 mmol), $Na_2S_2O_4$ (0.2 mmol), $NaHSO_3$ (0.15 mmol) and Mes-AcrClO$_4$ (2 mol%), then add into dry MeCN (1.5 mL). Then cap the vial Close the lid, remove from the glove box, and keep the temperature at 20-22 °C. The reaction mixture was placed in a water bath Stir for 1 hour at 900 rpm under 35 W blue LED light. After the reaction, the solvent was removed under reduced pressure, and 1,3,5-trimethoxybenzene was used as the internal standard, and the product yield was 62.0% (1.00×10^{-5} mol) by ^{1}H NMR

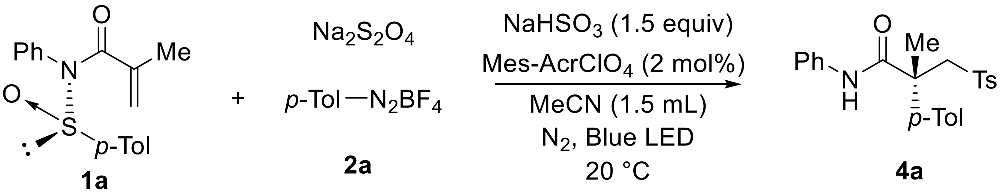

$$\Phi\ (\mathbf{4a}) = \frac{Mol\ product}{flux * t * f} = \frac{6.20 \times 10^{-5}}{5.26 \times 10^{-9} \times 3600 \times 0.999} = 3.28$$

Method B: thianthrenium salts system

Under nitrogen, a dry quartz vial equipped with a magnetic stir bar was charged with **1a** (0.1 mmol), **3a** (0.2 mmol), Rongalite (0.12 mmol), DABCO·$(SO_2)_2$ (0.2 mmol), NaOH (0.12 mmol) and *fac*-Ir(ppy)$_3$ (2 mol%), then add into dry MeCN (1.5 mL). Then cap the vial Close the lid, remove from the glove box, and keep the temperature at 20-22 °C. The reaction mixture was placed in a water bath Stir for 1 hour at 900 rpm under 35 W blue LED light. After the reaction, the solvent was removed under reduced pressure, and 1,3,5-trimethoxybenzene was used as the internal standard, and the product yield was 46.0% (2.60×10^{-5} mol) by ^{1}H NMR.

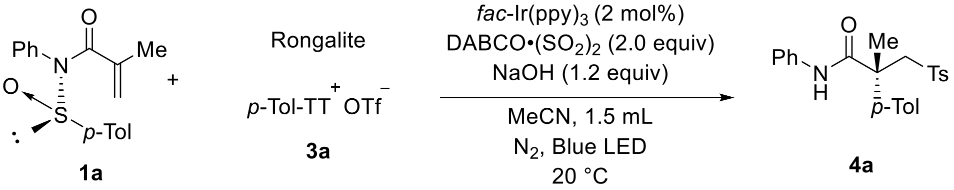

$$\Phi\ (4a) = \frac{Mol\ product}{flux*t*f} = \frac{4.60\times10^{-5}}{5.26\times10^{-9}\times3600\times0.999} = 2.43$$

Conclusion: The quantum yields of the reaction was determined to be 2.43, showing that the extended radical-chain reactions were possible.

2.3.3 Stern-Volmer fluorescence quenching experiments.

Stern-Volmer fluorescence quenching experiments were run with freshly prepared solutions of 0.1 mM Ir(ppy)$_3$ in degassed dry CH_3CN added with the appropriate amount of a quencher in a screw-top quartz cuvette at room temperature. The solutions were irradiated at 395 nm and fluorescence was measured from 460 nm to 640 nm.

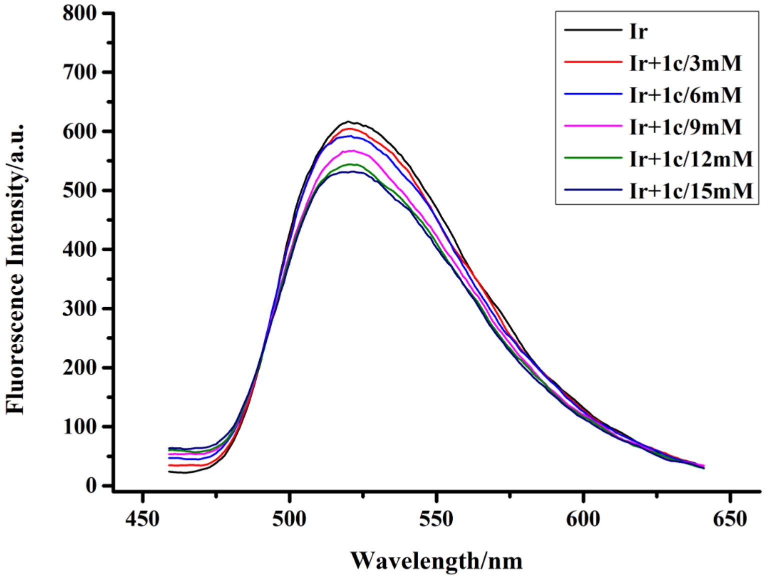

Supplementary Figure 3. Fluorescence quenching experiments of Ir(ppy)$_3$ and **1c**

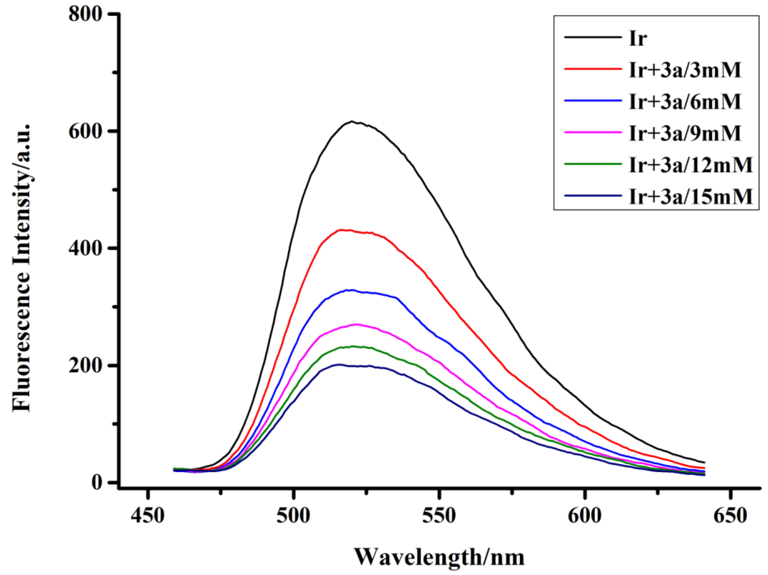

Supplementary Figure 4. Fluorescence quenching experiments of Ir(ppy)3 and **3a**

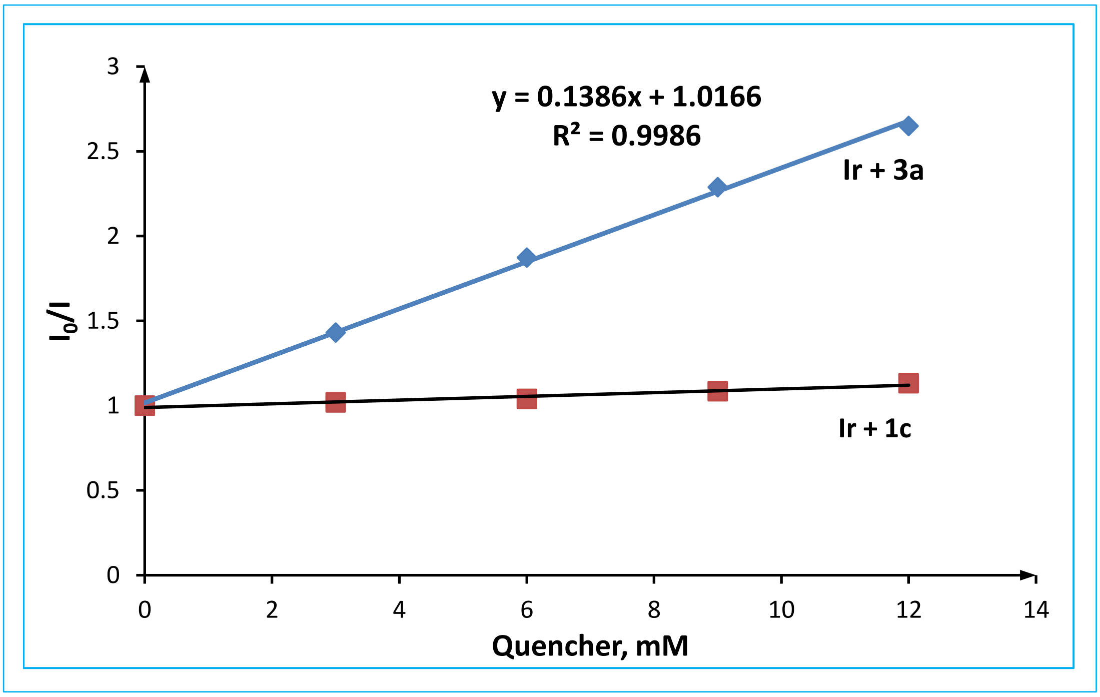

Supplementary Figure 5. Stern-Volmer plots of Ir(ppy)3 with different quenchers

Conclusion: the Stern-Volmer quenching experiments showed that substrate **3a** could effectively quench the excited *Ir(ppy)3, whereas **1c** could not.

2.3.4 Plausible mechanism of thianthrenium salts system

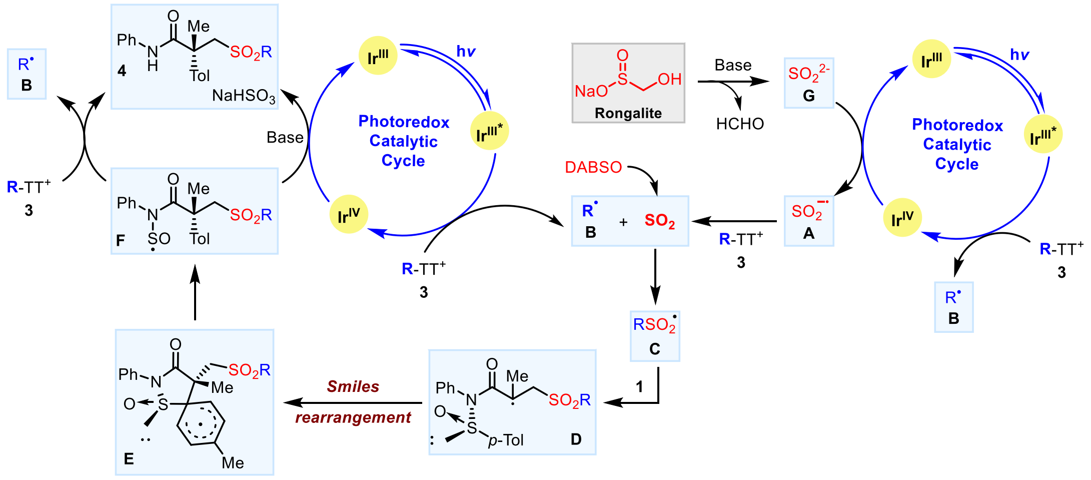

Supplementary Figure 6. Proposed mechanism for thianthrenium salts system

On basis of experimental results and previous reports,[2-6] a plausible mechanism for this transformation is proposed. Irradiation of $Ir(ppy)_3$ with visible light produces a long-lived (t = 1.9 μs) photoexcited state, $*Ir(ppy)_3$ ($E_{1/2}[Ir(ppy)_3^+/*Ir(ppy)_3]$ = -1.73 V vs. SCE), which can be readily oxidized by an appropriate quencher. Initially, photoexcited Ir(III)* would reduce thianthrenium salt **3** via a single electron transfer (SET) to generate radical species **B**, which could readily capture the SO_2 from DABCO·$(SO_2)_2$ to give to sulfonyl radical species **C**.[5] As an intensification strategy for this process, another alternative pathway to provide radical species **B** and **SO_2** was introduced in this system as follows. With the assistance of strong base NaOH, rongalite could release formaldehyde to generate sulfur dioxide anion **G** (SO_2^{2-}),[3,4] which would undergo single-electron transfer (SET) with Ir(IV) to produce sulfur dioxide radical anions **A**. Further single-electron transfer (SET) between dioxide radical anions **A** and thianthrenium salt **3** would occur to release radical species **B**, with simultaneous extrusion of SO_2. Subsequently, sulfonyl radical species **C** would add to the double bond to form new radical intermediate **D**. The radical Truce-Smiles rearrangement proceeds spontaneously to generate the SO-centred radical **F**,[2] traversing through a spirocyclic transition state **E** in an exothermic process.[6] The SO-centred radical **F** undergoes SET oxidation with Ir (IV) and further reacts with NaOH to convert into final product **4** and $NaHSO_3$. Furthermore, the SO-centred radical **F** will reduce thianthrenium salt **3** to generate radical species **B** and re-enter the cycle, thereby rationally explaining the quantum yields of 2.43 for the reaction.

2.4 Synthesis procedures and characterization data of substrates 1

The synthesis of compounds **1** was referred to the published paper.[2]

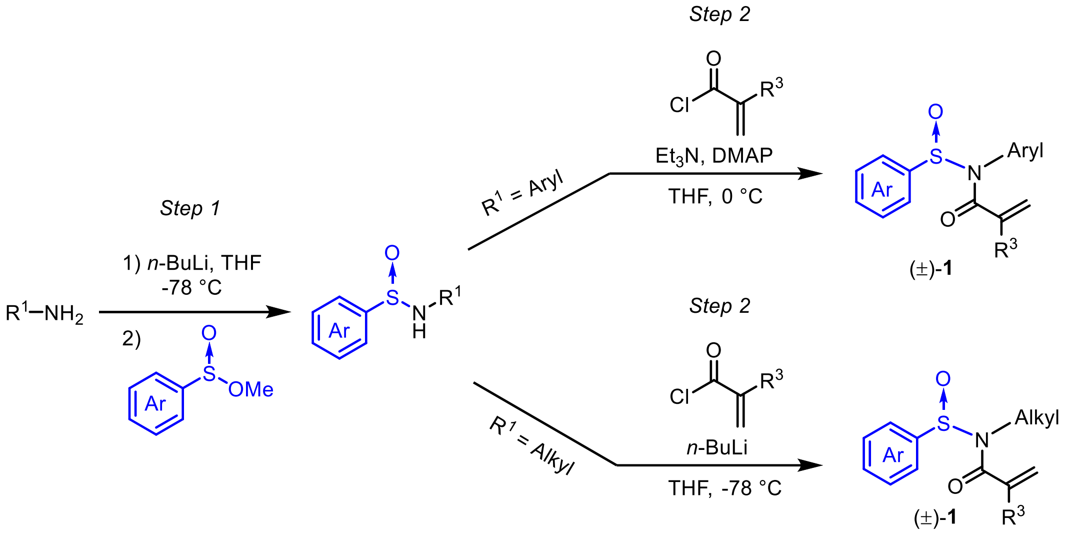

Supplementary Figure 7. Synthetic route of compound (rac)-**1**

<u>Step 1</u>

Formation of racemic *N*-arylsulfinamides

In a 50 mL two-necked round bottomed flask under nitrogen flow, the corresponding aniline (15 mmol, 2.5 equiv) was dissolved in THF (12 mL) and cooled to -78 °C. Then *n*-BuLi solution (1.6 M in *n*-hexane, 5.0 mL, 12.0 mmol, 2 equiv) was added dropwise. After 20 min, a solution of methylsulfinate (6.0 mmol, 1 equiv) in THF (8 mL) was added slowly. After 1 hour, the mixture was quenched at -78 °C with an aqueous saturated $NaHCO_3$ solution (3 x 30 mL). The mixture was extracted with EtOAc (3 x 20 mL). The organic phases were combined, dried over $MgSO_4$, filtered and concentrated in vacuo. *N*-hexane (10 mL) was added to the residue and the precipitate was filtered through a Buchner funnel, washed with additional *n*-hexane (20 mL) and dried in vacuo to afford the pure *N*-arylsulfinamides.

<u>Step 2</u>

Formation of racemic N-aryl-N-sulfinylacrylamides

To an oven-dried two-necked round bottomed flask were sequentially added the corresponding sulfinamide (2.0 mmol, 1 equiv), Et_3N (1.7 mL, 12.0 mmol, 6.0 equiv) and DMAP (12.0 mg, 0.1 mmol, 0.05 equiv) in THF (50 mL, 0.04 M) under nitrogen flow. The mixture was cooled to 0 °C (ice bath) and methacryloyl chloride (0.3 mL, 3.0 mmol, 1.5 equiv) was added dropwise. After 1 hour, the reaction mixture was diluted with EtOAc (15 mL), filtered and transferred into a separatory funnel. The mixture was washed with an aqueous saturated $NaHCO_3$ solution (3 x 30 mL). The combined organic phases were dried over $MgSO_4$, filtered and concentrated in vacuo. *N*-hexane (10 mL) was added to the residue and the precipitate was filtered through a Buchner funnel, washed with additional *n*-hexane (20 mL) and dried in vacuo to afford the sulfinylacrylamides, which were further purified by precipitation or crystallization.

Formation of racemic N-alkyl-N-sulfinylacrylamides

In a 50 mL two-necked round bottomed flask under nitrogen flow, the corresponding *N*-alkylphenylsulfinamide (1 mmol, 1 equiv) was dissolved in THF (25 mL) and cooled to -78 °C. Then *n*-BuLi solution (1.6 M in *n*-hexane, 0.75 mL, 1.2 mmol, 1.2 equiv) was added dropwise. After 20 min, methacryloyl chloride (1.1 mmol, 1.1 equiv) was added slowly. After 2 minutes, the mixture was washed with an aqueous saturated $NaHCO_3$ solution (10 mL). The mixture was extracted with EtOAc (3 x 20 mL). The organic phases were combined, dried over $MgSO_4$, filtered and concentrated in vacuo to afford the sulfinylacrylamides, which were further purified by precipitation or crystallization.

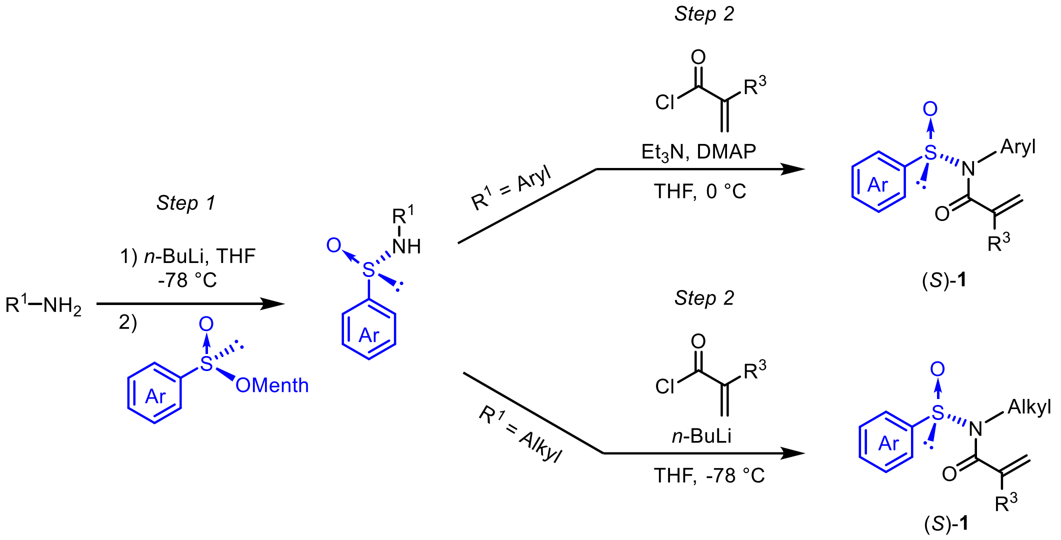

Supplementary Figure 8. Synthetic route of compound (S)-**1**

<u>Step 1</u>

Formation of enantiopure *N*-aryl and *N*-alkylsulfinamides

The same procedures described for the synthesis of racemic *N*-aryl and *N*-alkylsulfinamides were followed using (1*R*, 2*S*, 5*R*)-(-)-Menthyl (*S*)-*p*-toluenesulfinate(Leyan.com).

<u>Step 2</u>

Formation of enantiopure *N*-aryl and *N*-alkyl-*N*-sulfinylacrylamides

The same procedures described for the synthesis of racemic *N*-aryl and *N*-sulfinylacrylamides were followed.

(*S*)-*N*-(*p*-Tolyl)-*N*-(*p*-tolylsulfinyl)methacrylamide (1a)

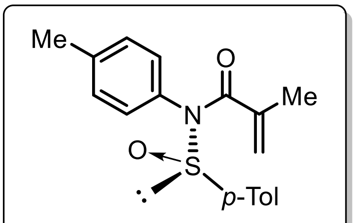

1a

60% yield, > 99.0 %ee

^{1}H NMR (400 MHz, $CDCl_3$) δ 7.32 (d, J = 8.2 Hz, 2H), 7.16 (d, J = 8.0 Hz, 2H), 6.99 (d, J = 8.2 Hz, 2H), 6.62 (d, J = 7.8 Hz, 2H), 5.36 (s, 1H), 5.29 (s, 1H), 2.36 (s, 3H), 2.29 (s, 3H), 1.87 (s, 3H).

HPLC analysis: OJ-H column, *n*-hexane/*i*-PrOH = 90/10, flow rate = 1.0 mL·min^{-1}, λ = 233 nm, t_R = 9.2 min (minor), 16.4 min (major).

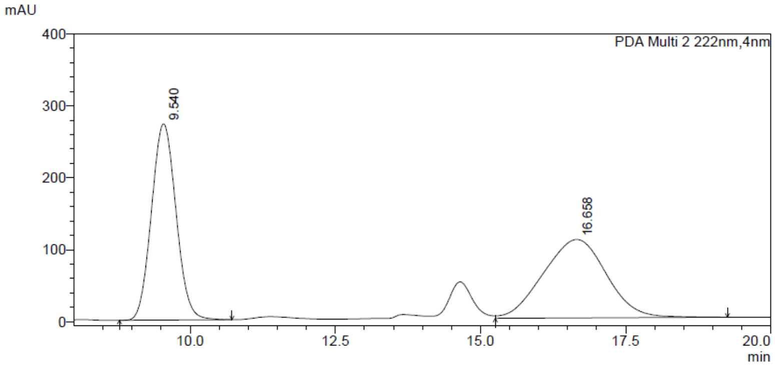

PDA Ch2 222nm

Peak#	Ret. Time	Area	Height	Aera%
1	9.540	8028705	272684	49.254
2	16.658	8271789	109030	50.746

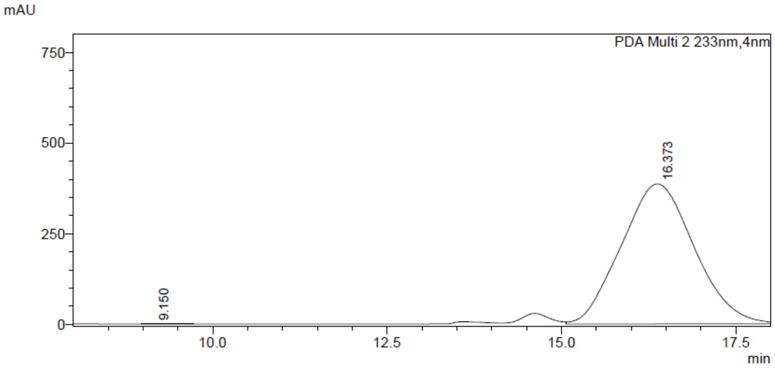

PDA Ch2 233nm

Peak#	Ret. Time	Area	Height	Aera%
1	9.150	2062	131	0.007
2	16.373	28187958	385724	99.993

Supplementary Figure 9. HPLC Spectra of compound **1a**

(*S*)-*N*-(4-Methoxyphenyl)-*N*-(*p*-tolylsulfinyl)methacrylamide (1b)

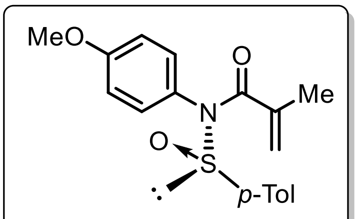

1b

62% yield, > 99.0 %ee

^{1}H NMR (400 MHz, $CDCl_3$) δ 7.31 (d, *J* = 8.2 Hz, 2H), 7.17 (d, *J* = 8.0 Hz, 2H), 6.71 – 6.64 (m, 4H), 5.37 (s, 1H), 5.30 (s, 1H), 3.76 (s, 3H), 2.36 (s, 3H), 1.87 (s, 3H).

HPLC analysis: AS-H column, *n*-hexane/*i*-PrOH = 80/20, flow rate = 1.0 mL·min^{-1}, λ = 254 nm, t_R = 11.2 min (minor), 17.5 min (major).

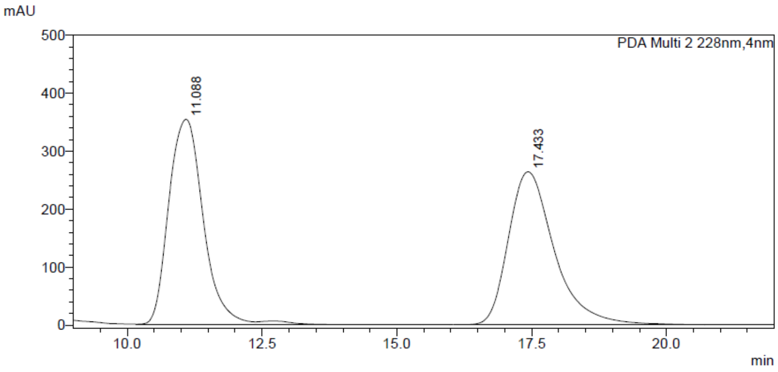

PDA Ch2 228nm

Peak#	Ret. Time	Area	Height	Aera%
1	11.088	15912793	353530	50.586
2	17.433	15544379	263272	49.414

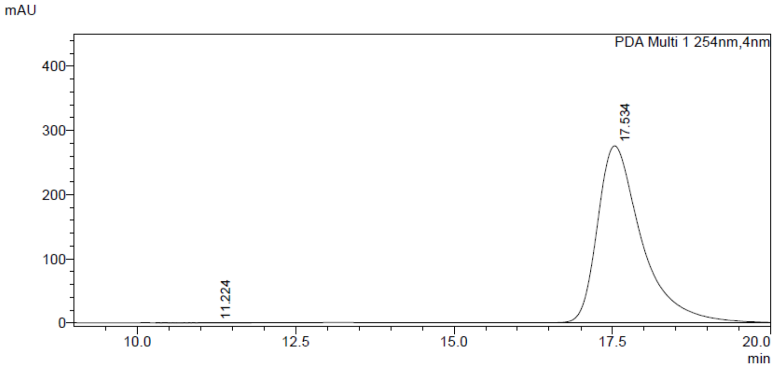

PDA Ch1 254nm

Peak#	Ret. Time	Area	Height	Aera%
1	11.224	2998	80	0.022
2	17.534	13365407	275458	99.978

Supplementary Figure 10. HPLC Spectra of compound **1b**

(*S*)-*N*-(4-Fluorophenyl)-*N*-(*p*-tolylsulfinyl)methacrylamide (1c)

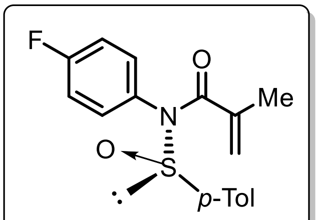

1c

62% yield, > 99.0 %ee

^{1}H NMR (400 MHz, $CDCl_3$) δ 7.32 – 7.24 (m, 2H), 7.17 (d, *J* = 7.9 Hz, 2H), 6.90 – 6.86 (m, 2H), 6.73 (dd, *J* = 8.0, 5.0 Hz, 2H), 5.42 (s, 1H), 5.37 (s, 1H), 2.36 (s, 3H), 1.93 (s, 3H).

HPLC analysis: OJ-H column, *n*-hexane/*i*-PrOH = 90/10, flow rate = 1.0 mL·min^{-1}, λ = 254 nm, t_R = 9.5 min (minor), 13.9 min (major).

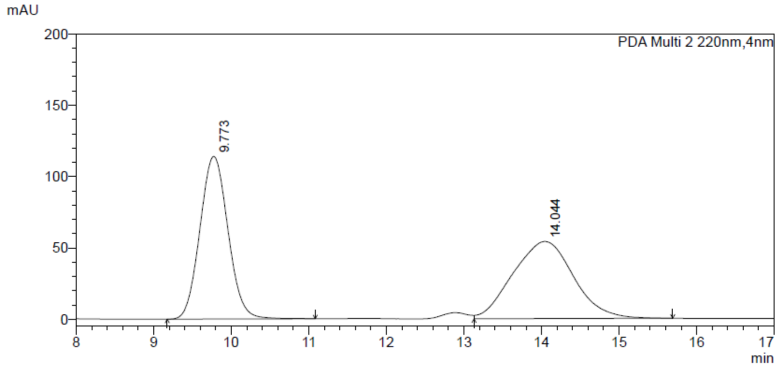

PDA Ch2 220nm

Peak#	Ret. Time	Area	Height	Aera%
1	9.773	2874360	114022	49.392
2	14.044	2945158	54049	50.608

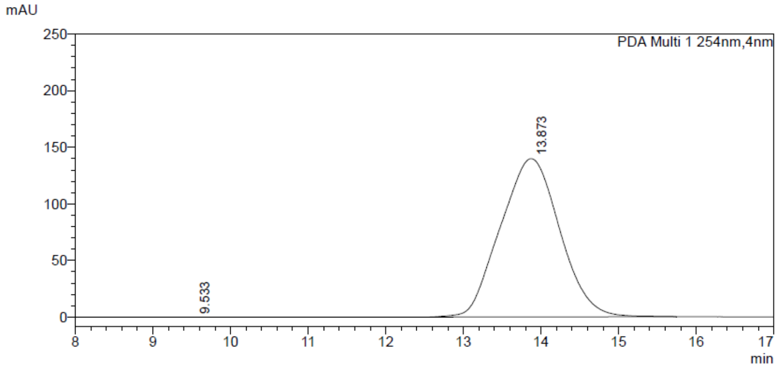

PDA Ch1 254nm

Peak#	Ret. Time	Area	Height	Aera%
1	9.533	56	12	0.001
2	13.873	7424777	139716	99.999

Supplementary Figure 11. HPLC Spectra of compound **1c**

(*S*)-*N*-Benzyl-*N*-(*p*-tolylsulfinyl)methacrylamide (1d)

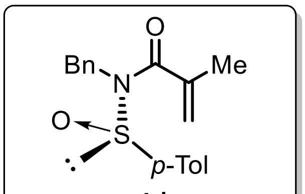

1d

58% yield, > 99.0 %ee

^{1}H NMR (400 MHz, $CDCl_3$) δ 7.47 (d, *J* = 8.2 Hz, 2H), 7.28 – 7.26 (m, 2H), 7.19 – 7.14 (m, 3H), 7.09 (dd, *J* = 7.2, 1.9 Hz, 2H), 5.52 (d, *J* = 8.2 Hz, 2H), 4.39 (d, *J* = 15.0 Hz, 1H), 4.30 (d, *J* = 15.1 Hz, 1H), 2.40 (s, 3H), 2.09 (s, 3H).

HPLC analysis: IA column, *n*-hexane/*i*-PrOH = 80/20, flow rate = 1.0 mL·min^{-1}, λ = 254 nm, t_R = 19.1 min (major), 26.2 min (minor)..

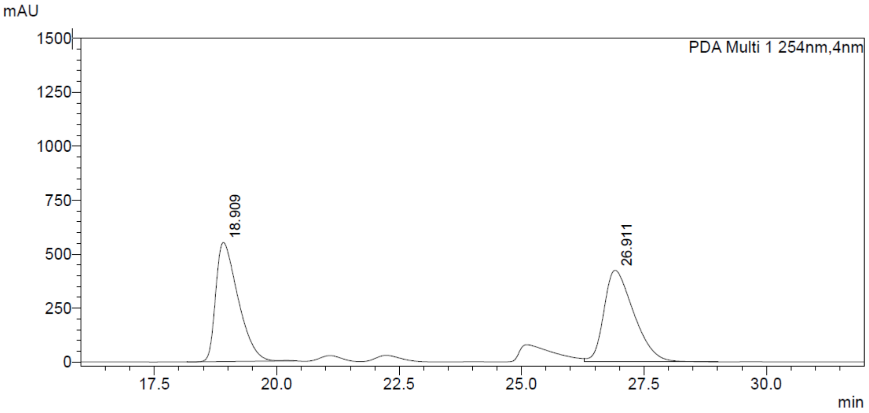

PDA Ch1 254nm

Peak#	Ret. Time	Area	Height	Aera%
1	18.909	17532704	551799	49.259
2	26.911	18060390	424489	50.741

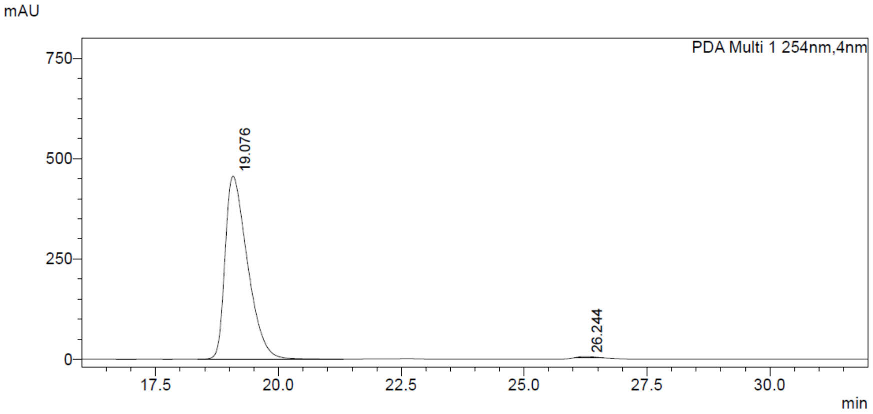

PDA Ch1 254nm

Peak#	Ret. Time	Area	Height	Aera%
1	19.076	14497535	456367	99.546
2	26.244	66108	3013	0.454

Supplementary Figure 12. HPLC Spectra of compound **1d**

(*S*)-*N*-((4-bromophenyl)sulfinyl)-*N*-phenylmethacrylamide (1e)

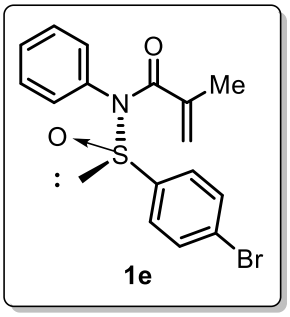

58% yield, > 99.0 %ee

^{1}H NMR (400 MHz, $CDCl_3$) δ 7.50 (d, J = 8.5 Hz, 2H), 7.34 – 7.26 (m, 3H), 7.24 – 7.20 (m, 2H), 6.75 (d, J = 7.4 Hz, 2H), 5.39 (s, 1H), 5.33 (s, 1H), 1.86 (s, 3H).

HPLC analysis: OD-H column, *n*-hexane/*i*-PrOH = 80/20, flow rate = 1.0 mL·min^{-1}, λ = 254 nm, t_R = 14.4 min (major), 22.2 min (minor).

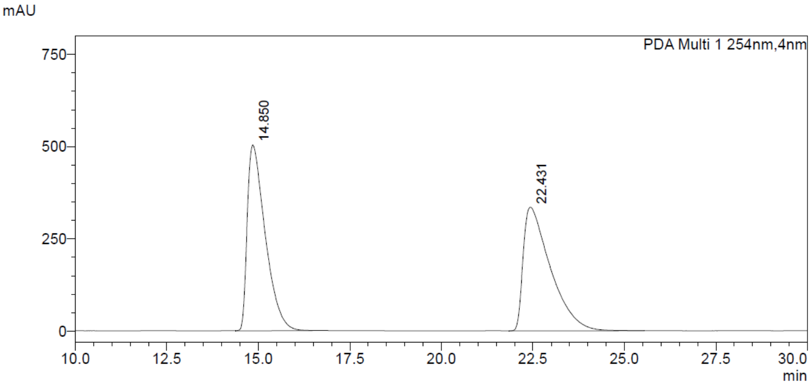

PDA Ch1 254nm

Peak#	Ret. Time	Area	Height	Aera%
1	14.850	17244823	503694	49.810
2	22.431	17376102	335146	50.190

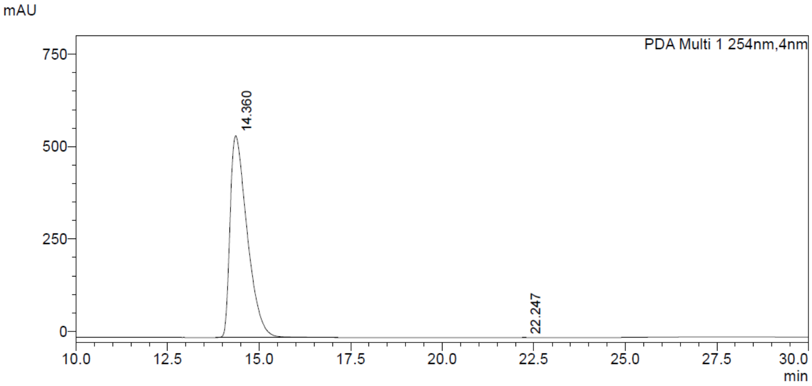

PDA Ch1 254nm

Peak#	Ret. Time	Area	Height	Aera%
1	14.360	17759084	546090	100.000
2	22.247	8	4	0.000

Supplementary Figure 13. HPLC Spectra of compound **1e**

(*S*)-*N*-benzyl-*N*-(*p*-tolylsulfinyl)acrylamide (1f)

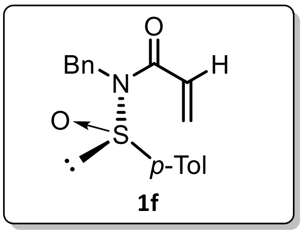

58% yield, 99.0 %ee

^{1}H NMR (400 MHz, $CDCl_3$) δ 7.48 (d, J = 8.1 Hz, 2H), 7.25 (d, J = 8.3 Hz, 2H), 7.21 – 6.97 (m, 6H), δ 6.56 (d, J = 16.6 Hz, 1H), 5.90 (d, J = 10.4 Hz, 1H)., 4.57 – 4.33 (m, 2H), 2.39 (s, 3H).

HPLC analysis: IA column, *n*-hexane/*i*-PrOH = 80/20, flow rate = 1.0 mL·min^{-1}, λ = 254 nm, t_R = 27.7 min (major), 32.5 min (minor).

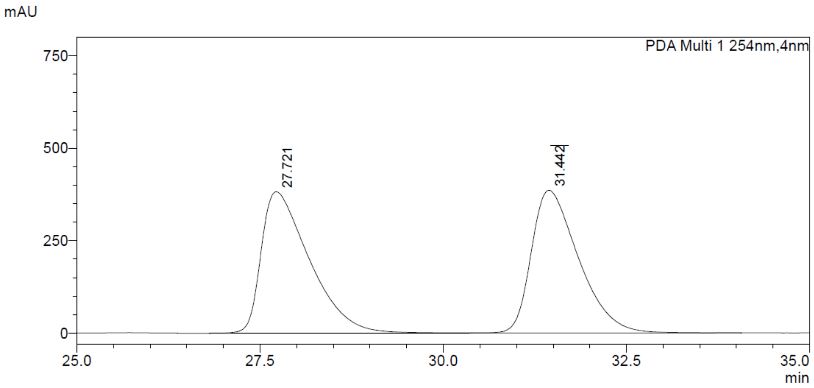

PDA Ch1 254nm

Peak#	Ret. Time	Area	Height	Aera%
1	27.721	17557912	382304	50.283
2	31.442	17360157	386077	49.717

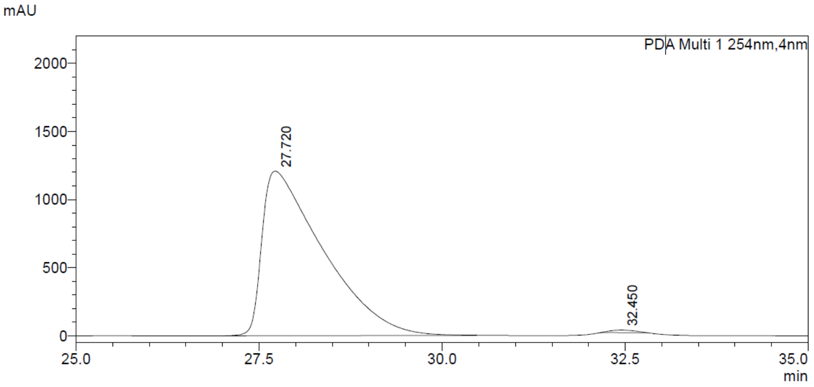

PDA Ch1 254nm

Peak#	Ret. Time	Area	Height	Aera%
1	27.720	70274706	1207622	99.343
2	32.450	464604	19549	0.657

Supplementary Figure 14. HPLC Spectra of compound **1f**

(*S*)-*N*,2-dibenzyl-*N*-(*p*-tolylsulfinyl)acrylamide (1g)

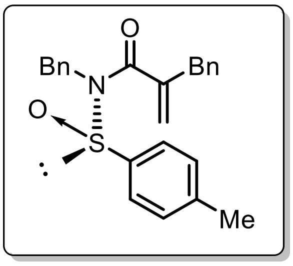

58% yield, 99.0 %ee

^{1}H NMR (400 MHz, $CDCl_3$) δ7.34 – 7.24 (m, 5H), 7.19 – 7.07 (m, 7H), 6.77 (d, *J* = 8.1 Hz, 2H), 5.63 (d, *J* = 13.9 Hz, 2H), 4.14 (s, 2H), 3.88 (d, *J* = 14.4 Hz, 1H), 3.64 (d, *J* = 14.5 Hz, 1H), 2.33 (s, 3H).

HPLC analysis: IA column, *n*-hexane/*i*-PrOH = 80/20, flow rate = 1.0 mL·min^{-1}, λ = 254 nm, t_R = 27.6 min (major), 37.2 min (minor).

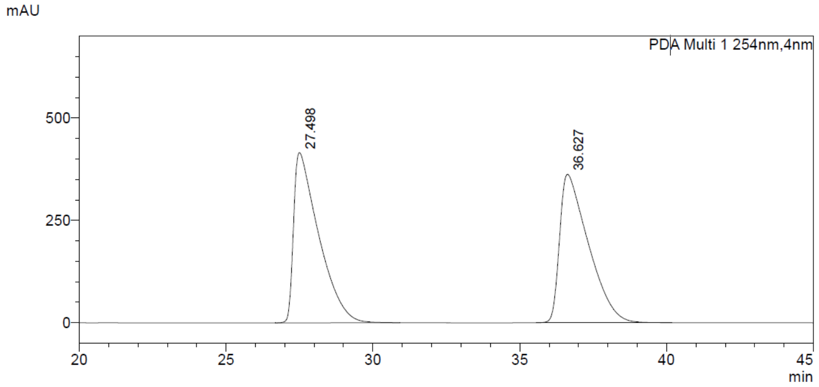

PDA Ch1 254nm

Peak#	Ret. Time	Area	Height	Aera%
1	27.498	24740510	415785	50.020
2	36.627	24720783	362474	49.980

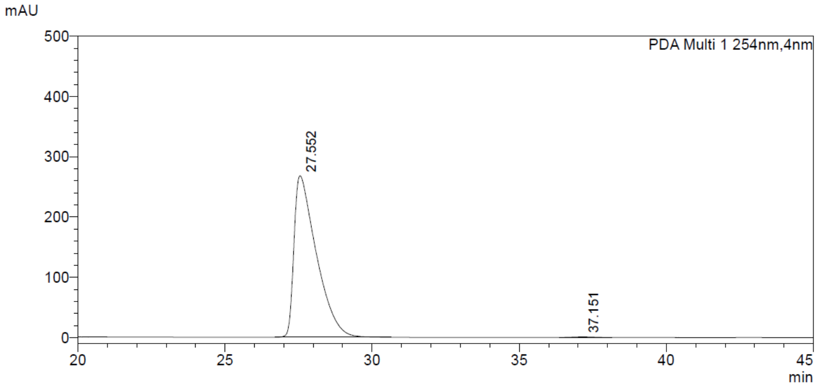

PDA Ch1 254nm

Peak#	Ret. Time	Area	Height	Aera%
1	27.552	14173310	268061	99.655
2	37.151	49035	953	0.345

Supplementary Figure 15. HPLC Spectra of compound **1g**

2.5 Characterization data of products 4

(*R*)-2-Methyl-*N*-phenyl-2-(*p*-tolyl)-3-tosylpropanamide (4a)

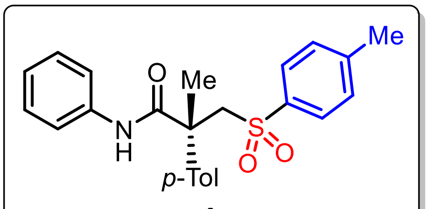

4a

Method A: 78% yield, 96% ee; **Method B**: 75% yield, 97% ee.
^{1}H NMR (400 MHz, $CDCl_3$) δ 7.51 (d, J = 8.2 Hz, 2H), 7.32 – 7.23 (m, 4H), 7.19 – 7.14 (m, 4H), 7.09 – 7.03 (m, 3H), 6.93 (s, 1H), 4.12 (d, J = 14.8 Hz, 1H), 3.82 (d, J = 14.8 Hz, 1H), 2.38 (s, 3H), 2.31 (s, 3H), 2.09 (s, 3H). **^{13}C NMR** (100 MHz, $CDCl_3$) δ 172.9, 144.0, 138.2, 138.0, 137.4, 136.3, 129.6, 129.5, 128.9, 127.6, 127.0, 124.6, 120.1, 64.1, 50.0, 22.7, 21.6, 21.0. **HRMS** (ESI) m/z Calcd for $[C_{24}H_{25}NNaO_3S, M+Na]^+$: 430.1447, found: 430.1452. $[\alpha]_D^{20}$ = -45 (c= 1.0, EtOAc).
HPLC analysis: AD-H column, *n*-hexane/*i*-PrOH = 50/50, flow rate = 1.0 mL·min^{-1}, λ = 254 nm, t_R = 12.0 min (minor), 17.1 min (major).

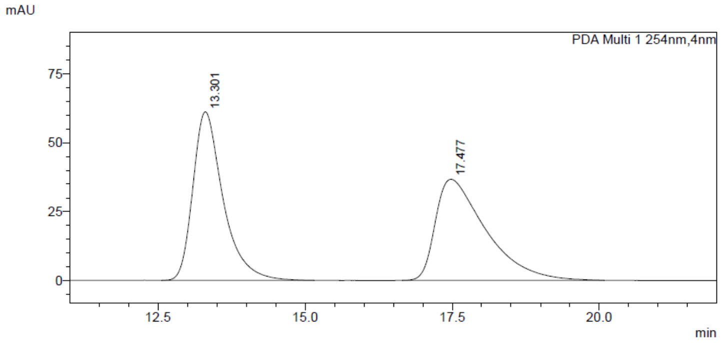

PDA Ch1 254nm

Peak#	Ret. Time	Area	Height	Aera%
1	13.301	2182101	61068	50.021
2	17.477	2180301	36611	49.979

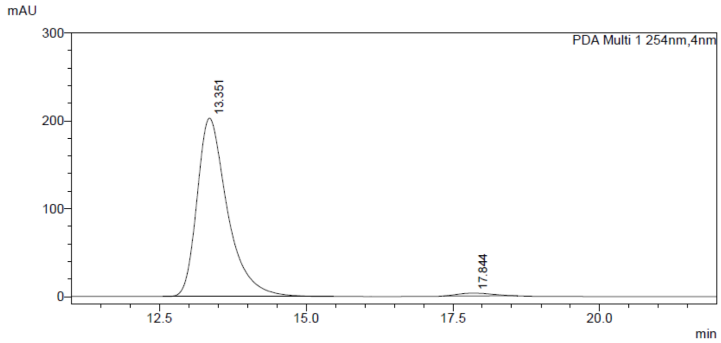

PDA Ch1 254nm

Peak#	Ret. Time	Area	Height	Aera%
1	13.351	7311506	202766	98.264
2	17.844	129165	3237	1.736

Supplementary Figure 16. HPLC Spectra of compound **4a** (Method A)

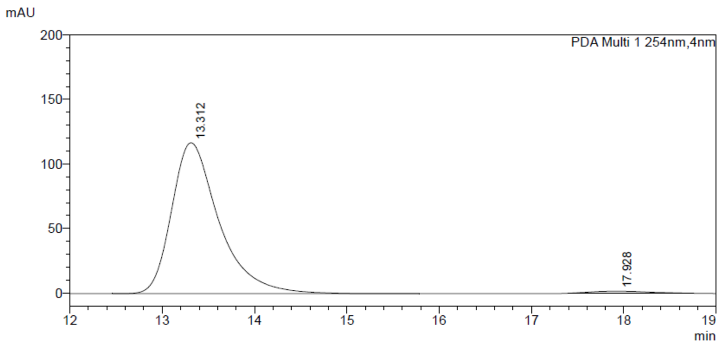

<Peak Table>

PDA Ch1 254nm

Peak#	Ret. Time	Area	Height	Aera%
1	13.312	4174251	116898	98.496
2	17.928	63742	1580	1.504

Supplementary Figure 17. HPLC Spectra of compound **4a** (Method B)

(*R*)-3-((4-Methoxyphenyl)sulfonyl)-2-methyl-*N*-phenyl-2-(*p*-tolyl)propanamide (4b)

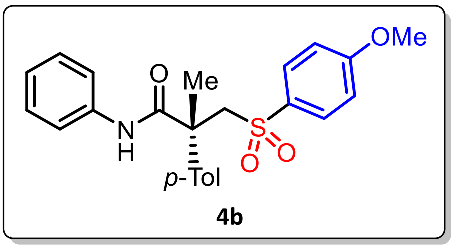

Method A: 75% yield, 97% ee; **Method B:** 62% yield, 98% ee.

^{1}H NMR (400 MHz, $CDCl_3$) δ 7.57 – 7.53 (m, 2H), 7.31 (d, J = 7.6 Hz, 2H), 7.26 (d, J = 7.9 Hz, 2H), 7.19 (d, J = 8.2 Hz, 2H), 7.09 – 7.05 (m, 3H), 6.93 (s, 1H), 6.83 – 6.79 (m, 2H), 4.13 (d, J = 14.8 Hz, 1H), 3.82(d, J = 14.8 Hz, 1H), 3.82 (s, 3H), 2.31 (s, 3H), 2.09 (s, 3H). **^{13}C NMR** (101 MHz, $CDCl_3$) δ 172.9, 163.3, 137.9, 137.4, 136.4, 132.7, 129.7, 129.6, 128.9, 127.0, 124.6, 120.1, 114.1, 64.2, 55.6, 50.0, 22.7, 21.0. **HRMS** (ESI) m/z Calcd for [$C_{24}H_{25}NNaO_4S$, M+Na]$^+$: 446.1397, found: 446.1402. $[\alpha]_D^{20}$ = -30 (c= 0.6, EtOAc).

HPLC analysis: AD-H column, *n*-hexane/*i*-PrOH = 50/50, flow rate = 1.0 mL·min^{-1}, λ = 254 nm, t_R = 13.4 min (minor), 17.8 min (major).

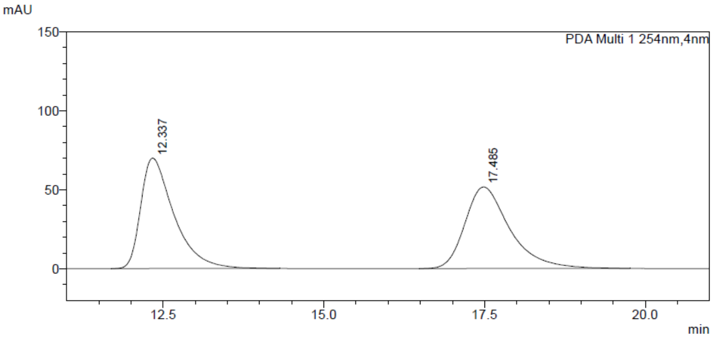

PDA Ch1 254nm

Peak#	Ret. Time	Area	Height	Aera%
1	12.337	2513297	69905	50.056
2	17.485	2507689	51626	49.944

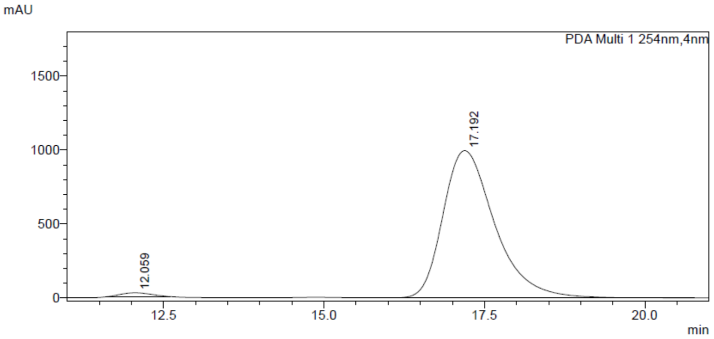

<

PDA Ch1 254nm

Peak#	Ret. Time	Area	Height	Aera%
1	12.059	867027	26498	1.526
2	17.192	55963289	996082	98.474

Supplementary Figure 18. HPLC Spectra of compound **4b** (Method A)

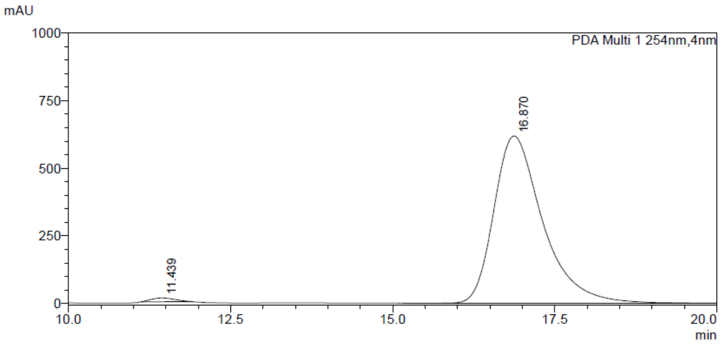

<Peak Table>

PDA Ch1 254nm

Peak#	Ret. Time	Area	Height	Aera%
1	11.439	346807	13744	1.058
2	16.870	32419626	618001	98.942

Supplementary Figure 19. HPLC Spectra of compound **4b** (Method B)

(*R*)-2-Methyl-3-((4-(methylthio)phenyl)sulfonyl)-*N*-phenyl-2-(*p*-tolyl)propanamide (4c)

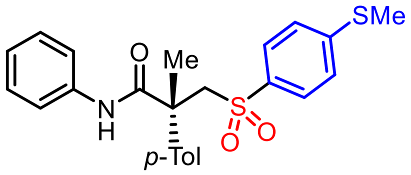

4c

Method A: 81% yield, 94% ee.

^{1}H NMR (400 MHz, $CDCl_3$) δ 7.44 (d, *J* = 8.6 Hz, 2H), 7.30 – 7.22 (m, 4H), 7.14 (d, *J* = 8.2 Hz, 2H), 7.10 – 7.04 (m, 3H), 7.02 (d, *J* = 7.1 Hz, 2H), 6.96 (s, 1H), 4.11 (d, *J* = 14.9 Hz, 1H), 3.84 (d, *J* = 14.9 Hz, 1H), 2.47 (s, 3H), 2.30 (s, 3H), 2.07 (s, 3H). **^{13}C NMR** (100 MHz, $CDCl_3$) δ 172.9, 146.4, 138.1, 137.4, 136.5, 136.1, 129.6, 128.9, 127.8, 127.0, 125.0, 124.7, 120.2, 64.1, 49.9, 22.6, 21.0, 14.8. **HRMS** (ESI) m/z Calcd for $[C_{24}H_{25}NNaO_3S_2, M+Na]^+$: 462.1168, found: 462.1170. $[\alpha]_D^{20}$ = -35 (c=0.12, EtOAc).

HPLC analysis: AD-H column, *n*-hexane/*i*-PrOH = 80/20, flow rate = 1.0 mL·min^{-1}, λ = 254 nm, t_R = 26.7 min (minor), 31.9 min (major).

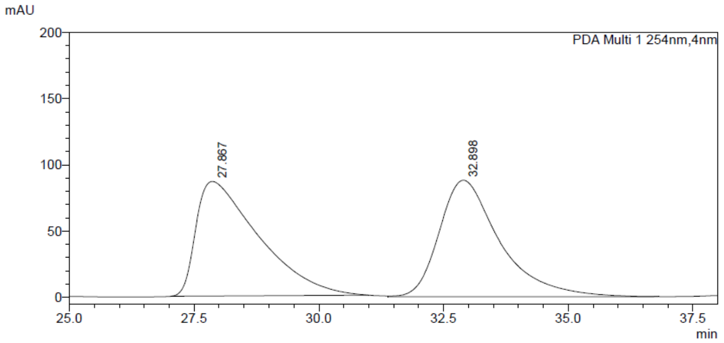

PDA Ch1 254nm

Peak#	Ret. Time	Area	Height	Aera%
1	27.867	7579895	86593	50.375
2	32.898	7466969	88023	49.625

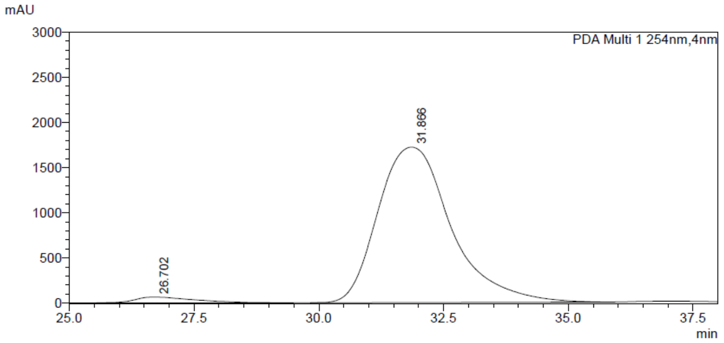

PDA Ch1 254nm

Peak#	Ret. Time	Area	Height	Aera%
1	26.702	5747018	66278	3.168
2	31.866	175674472	1719882	96.832

Supplementary Figure 20. HPLC Spectra of compound **4c** (Method A)

(*R*)-3-((4-(Tert-butyl)phenyl)sulfonyl)-2-methyl-*N*-phenyl-2-(*p*-tolyl)propanamide (4d)

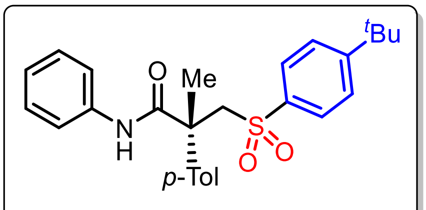

4d

Method A: 59% yield, 97%ee; **Method B:** 60% yield, 96% ee.

^{1}H NMR (400 MHz, $CDCl_3$) δ 7.53 (d, *J* = 8.4 Hz, 2H), 7.35 (d, *J* = 8.4 Hz, 2H), 7.30 (d, *J* = 8.1 Hz, 2H), 7.27 – 7.23 (m, 2H), 7.17 (d, *J* = 8.1 Hz, 2H), 7.08 – 7.01 (m, 3H), 6.90 (s, 1H), 4.12 (d, *J* = 14.9 Hz, 1H), 3.87 (d, *J* = 14.9 Hz, 1H), 2.30 (s, 3H), 2.10 (s, 3H), 1.31 (s, 9H). **^{13}C NMR** (100 MHz, $CDCl_3$) δ 173.0, 156.9, 138.0, 137.8, 137.4, 136.2, 129.6, 128.9, 127.3, 127.0, 125.9, 124.6, 120.1, 64.0, 50.0, 35.1, 31.1, 22.8, 21.1. **HRMS** (ESI) m/z Calcd for $[C_{27}H_{31}NNaO_3S, M+Na]^+$: 472.1917, found: 472.1921. $[\alpha]_D^{20}$ = -19 (c= 0.14, EtOAc).

HPLC analysis: IA column, *n*-hexane/*i*-PrOH = 90/10, flow rate = 1.0 mL·min^{-1}, λ = 254 nm, t_R = 9.8 min (minor), 12.7 min (major).

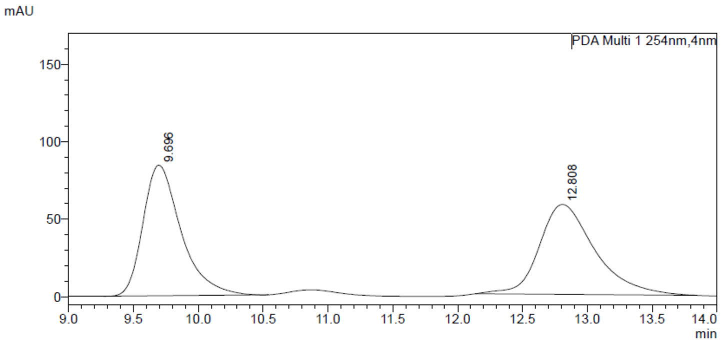

PDA Ch1 254nm

Peak#	Ret. Time	Area	Height	Aera%
1	9.696	1724741	84324	49.816
2	12.808	1737451	58022	50.184

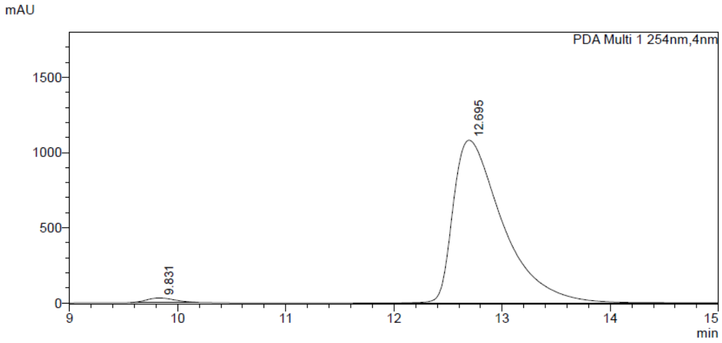

PDA Ch1 254nm

Peak#	Ret. Time	Area	Height	Aera%
1	9.831	526979	30069	1.499
2	12.695	34639762	1081751	98.501

Supplementary Figure 21. HPLC Spectra of compound **4d** (Method A)

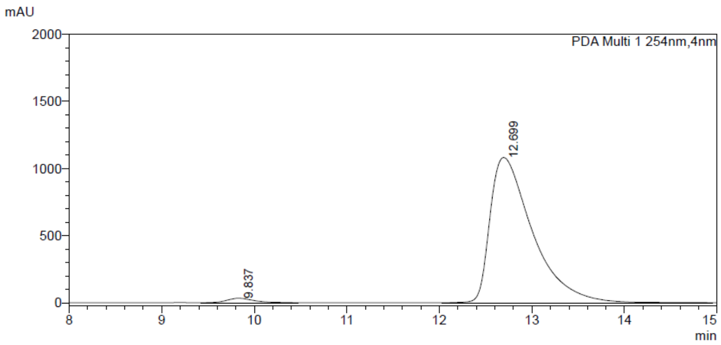

PDA Ch1 254nm

Peak#	Ret. Time	Area	Height	Aera%
1	9.837	677023	33198	1.921
2	12.699	34564341	1081388	98.079

Supplementary Figure 22. HPLC Spectra of compound **4d** (Method B)

(*R*)-2-Methyl-*N*-phenyl-2-(*p*-tolyl)-3-((4-(trifluoromethyl)phenyl)sulfonyl)propanamide (4e)

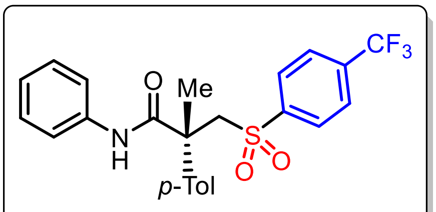

4e

Method A: 65% yield, 95% ee.

^{1}H NMR (400 MHz, $CDCl_3$) δ 7.61 (d, *J* = 8.2 Hz, 2H), 7.53 (d, *J* = 8.3 Hz, 2H), 7.28 – 7.23 (m, 4H), 7.09 – 7.06 (m, 3H), 6.93 (d, *J* = 8.0 Hz, 2H), 6.82 (s, 1H), 4.11 (d, *J* = 15.3 Hz, 1H), 4.00 (d, *J* = 15.3 Hz, 1H), 2.26 (s, 3H), 2.10 (s, 3H). **^{13}C NMR** (100 MHz, $CDCl_3$) δ 172.9, 143.9, 138.4, 137.2, 135.2, 134.3 (q, *J* = 33.0 Hz), 129.6, 129.0, 128.1, 127.2, 125.8 (q, *J* = 4.0 Hz), 124.8, 123.2 (q, *J* = 272.8 Hz), 120.1, 64.0, 49.8, 22.5, 20.8. **HRMS** (ESI) m/z Calcd for $[C_{24}H_{22}F_3NNaO_3S, M+Na]^+$: 484.1165, found: 484.1166. $[\alpha]_D^{20}$ = -45 (c= 0.1, EtOAc).

HPLC analysis: IA column, *n*-hexane/*i*-PrOH = 90/10, flow rate = 1.0 mL·min^{-1}, λ = 254 nm, t_R = 12.3 min (minor), 16.9 min (major).

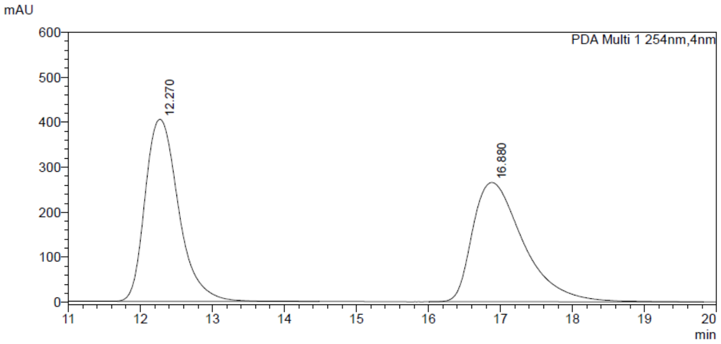

PDA Ch1 254nm

Peak#	Ret. Time	Area	Height	Aera%
1	12.270	13100170	404946	50.181
2	16.880	13005455	265094	49.819

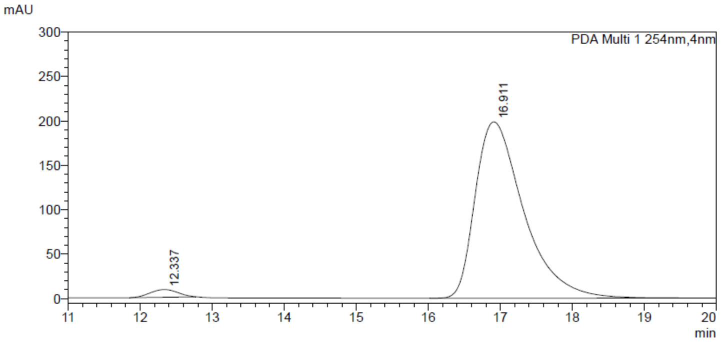

PDA Ch1 254nm

Peak#	Ret. Time	Area	Height	Aera%
1	12.337	229596	8581	2.405
2	16.911	9316316	198526	97.595

Supplementary Figure 23. HPLC Spectra of compound **4e** (Method A)

(*R*)-3-((4-Cyanophenyl)sulfonyl)-2-methyl-*N*-phenyl-2-(*p*-tolyl)propanamide (4f)

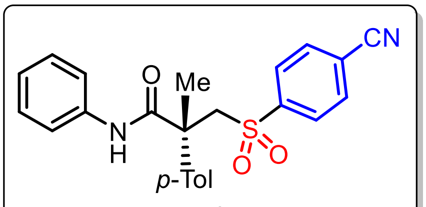

4f

Method A: 65% yield, 96% ee.

^{1}H NMR (400 MHz, $CDCl_3$) δ 7.65 (d, *J* = 8.3 Hz, 2H), 7.59 (d, *J* = 8.2 Hz, 2H), 7.30 – 7.24 (m, 4H), 7.12 – 7.07 (m, 3H), 7.00 (d, *J* = 7.9 Hz, 2H), 6.89 (s, 1H), 4.12 (d, *J* = 15.1 Hz, 1H), 3.94 (d, *J* = 15.1 Hz, 1H), 2.31 (s, 3H), 2.10 (s, 3H). **^{13}C NMR** (100 MHz, $CDCl_3$) δ 172.6, 144.7, 138.6, 137.2, 135.5, 132.5, 129.7, 129.0, 128.3, 127.1, 124.9, 120.2, 117.2, 116.4, 64.2, 49.9, 22.5, 21.0. **HRMS** (ESI) m/z Calcd for $[C_{24}H_{22}N_2NaO_3S, M+Na]^+$: 441.1243, found: 441.1253. $[\alpha]_D^{20}$ = -45 (c= 0.1, EtOAc).

HPLC analysis: IA column, *n*-hexane/*i*-PrOH = 90/10, flow rate = 1.0 mL·min^{-1}, λ = 254 nm, t_R = 25.6 min (minor), 38.2 min (major).

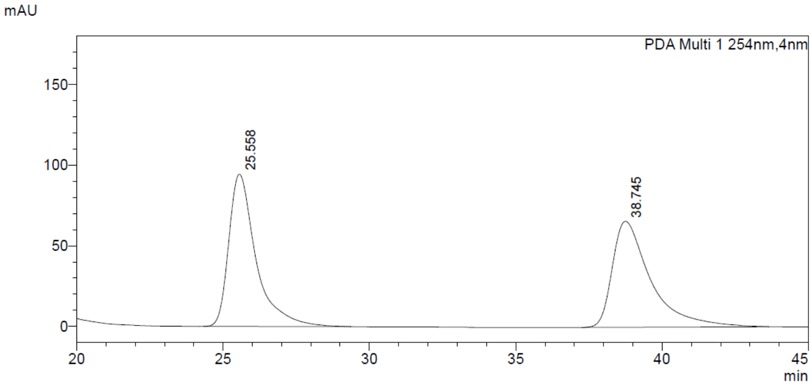

PDA Ch1 254nm

Peak#	Ret. Time	Area	Height	Aera%
1	25.558	6121506	94488	50.548
2	38.745	5988782	65719	49.452

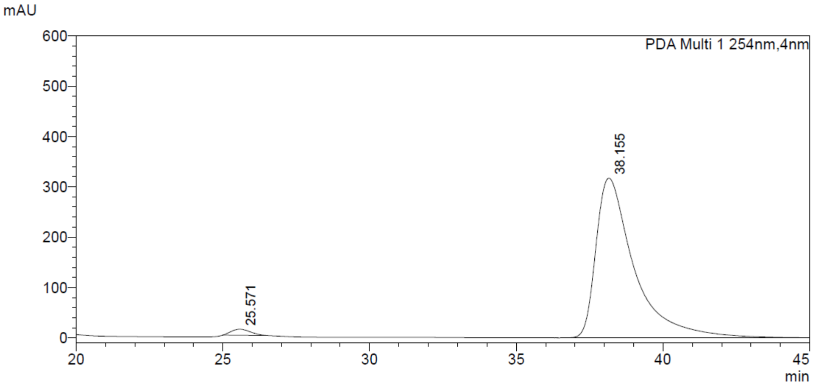

PDA Ch1 254nm

Peak#	Ret. Time	Area	Height	Aera%
1	25.571	545994	11973	1.856
2	38.155	28863914	317289	98.144

Supplementary Figure 24. HPLC Spectra of compound **4f** (Method A)

(*R*)-2-Methyl-3-((4-nitrophenyl)sulfonyl)-*N*-phenyl-2-(*p*-tolyl)propanamide (4g)

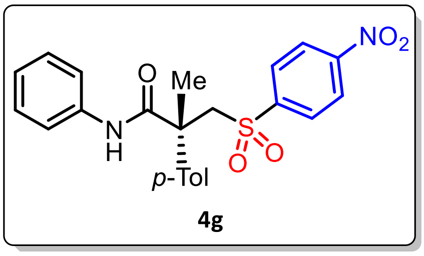

Method A: 54% yield, 95% ee.

^{1}H NMR (400 MHz, $CDCl_3$) δ 8.12 (d, *J* = 8.8 Hz, 2H), 7.70 (d, *J* = 8.8 Hz, 2H), 7.29 – 7.24 (m, 4H), 7.11 – 7.07 (m, 3H), 6.97 (d, *J* = 8.0 Hz, 2H), 6.83 (s, 1H), 4.14 (d, *J* = 15.3 Hz, 1H), 3.99 (d, *J* = 15.3 Hz, 1H), 2.28 (s, 3H), 2.11 (s, 3H). **^{13}C NMR** (100 MHz, $CDCl_3$) δ 172.6, 150.0, 146.0, 138.7, 137.1, 135.4, 129.7, 129.0, 127.2, 124.9, 124.4, 123.9, 120.1, 64.3, 49.9, 22.5, 20.9. **HRMS** (ESI) m/z Calcd for $[C_{23}H_{22}N_2NaO_5S, M+Na]^+$: 461.1142, found: 461.1148. $[\alpha]_D^{20}$ = -40 (c= 0.1, EtOAc).

HPLC analysis: IA column, *n*-hexane/*i*-PrOH = 60/40, flow rate = 1.0 mL·min^{-1}, λ = 254 nm, t_R = 27.0 min (minor), 40.3 min (major).

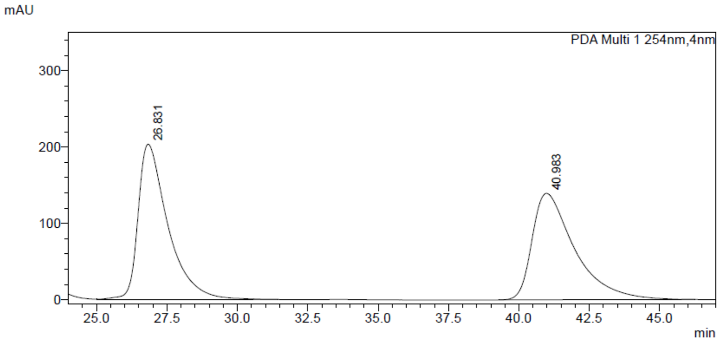

PDA Ch1 254nm

Peak#	Ret. Time	Area	Height	Aera%
1	26.831	15061032	203349	50.710
2	40.983	14639064	139223	49.290

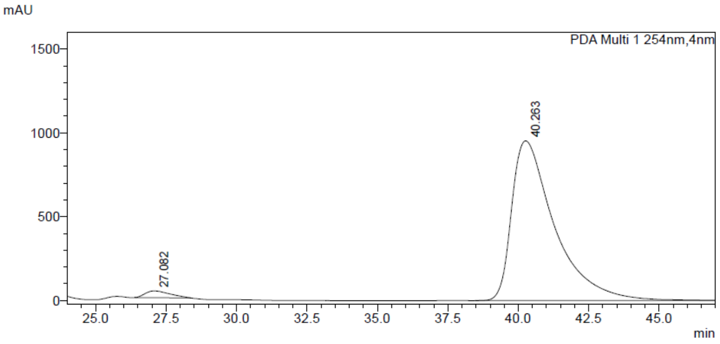

PDA Ch1 254nm

Peak#	Ret. Time	Area	Height	Aera%
1	27.082	2486013	40592	2.408
2	40.263	100764271	952312	97.592

Supplementary Figure 25. HPLC Spectra of compound **4g** (Method A)

(*R*)-3-((4-Fluorophenyl)sulfonyl)-2-methyl-*N*-phenyl-2-(*p*-tolyl)propanamide (4h)

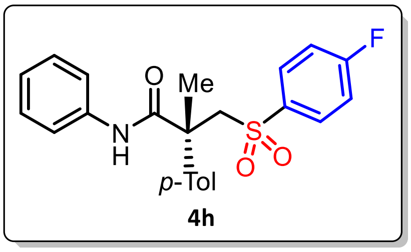

Method A: 65% yield, 95% ee.

^{1}H NMR (400 MHz, $CDCl_3$) δ 7.59 (dd, *J* = 8.7, 5.1 Hz, 2H), 7.31 – 7.23 (m, 4H), 7.15 (d, *J* = 8.2 Hz, 2H), 7.09 – 6.98 (m, 5H), 6.92 (s, 1H), 4.12 (d, *J* = 15.0 Hz, 1H), 3.87 (d, *J* = 15.0 Hz, 1H), 2.31 (s, 3H), 2.10 (s, 3H). **^{13}C NMR** (100 MHz, $CDCl_3$) δ 172.9, 165.4 (d, *J* = 255.6 Hz), 138.2, 137.3, 137.0 (d, *J* = 3.1 Hz), 135.9, 130.4 (d, *J* = 9.6 Hz), 129.7, 129.0, 127.0, 124.8, 120.2, 116.1 (d, *J* = 22.7 Hz), 64.3, 50.0, 22.6, 21.0. **HRMS** (ESI) m/z Calcd for [$C_{23}H_{22}FNNaO_3S$, M+Na]$^+$: 434.1197, found: 434.1201. $[\alpha]_D^{20}$ = -38 (c= 0.12, EtOAc).

HPLC analysis: IA column, *n*-hexane/*i*-PrOH = 90/10, flow rate = 1.0 mL·min^{-1}, λ = 254 nm, t_R = 15.4 min (minor), 24.6 min (major).

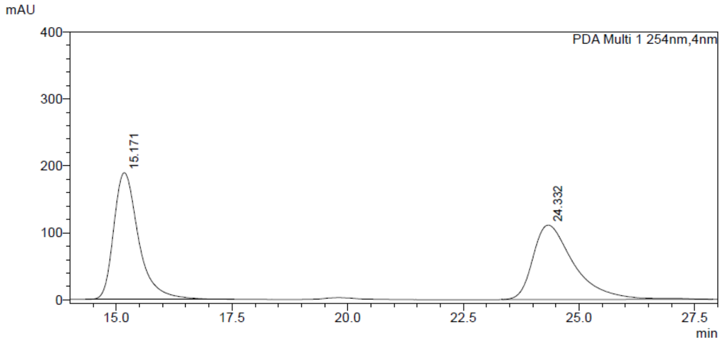

PDA Ch1 254nm

Peak#	Ret. Time	Area	Height	Aera%
1	15.171	7087052	189232	50.745
2	24.332	6879054	111157	49.255

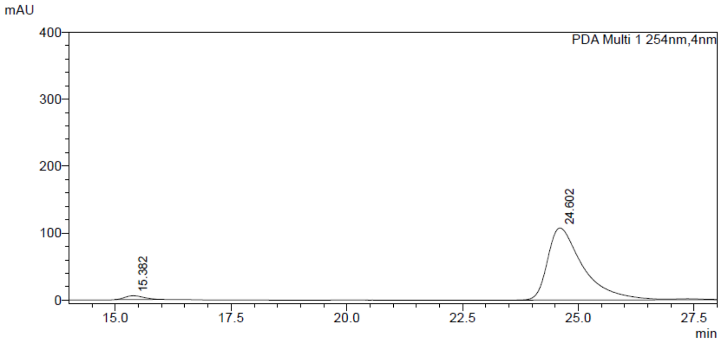

PDA Ch1 254nm

Peak#	Ret. Time	Area	Height	Aera%
1	15.382	157943	5694	2.653
2	24.602	5795322	107512	97.347

Supplementary Figure 26. HPLC Spectra of compound **4h** (Method A)

(*R*)-3-((4-Chlorophenyl)sulfonyl)-2-methyl-*N*-phenyl-2-(*p*-tolyl)propanamide (4i)

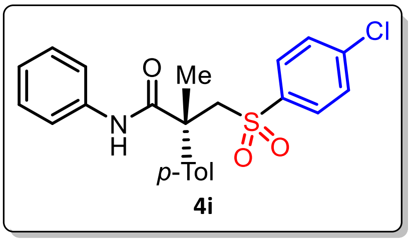

Method A: 63% yield, 95% ee; **Method B:** 60% yield, 96% ee.

^{1}H NMR (400 MHz, $CDCl_3$) δ 7.49 (d, *J* = 8.6 Hz, 2H), 7.33 – 7.24 (m, 6H), 7.13 (d, *J* = 8.2 Hz, 2H), 7.10 – 7.06 (m, 1H), 7.03 (d, *J* = 8.0 Hz, 2H), 6.83 (s, 1H), 4.12 (d, *J* = 15.1 Hz, 1H), 3.89 (d, *J* = 15.1 Hz, 1H), 2.32 (s, 3H), 2.10 (s, 3H). **^{13}C NMR** (100 MHz, $CDCl_3$) δ 172.8, 139.6, 139.2, 138.4, 137.2, 135.6, 129.7, 129.1, 129.1, 129.0, 127.1, 124.8, 120.1, 64.1, 49.9, 22.6, 21.0. **HRMS** (ESI) m/z Calcd for $[C_{23}H_{22}ClNNaO_3S, M+Na]^+$: 450.0901 (452.0872), found: 450.0901 (452.0882). $[\alpha]_D^{20}$ = -30 (c= 0.12, EtOAc).

HPLC analysis: AD-H column, *n*-hexane/*i*-PrOH = 70/30, flow rate = 1.0 mL·min^{-1}, λ = 254 nm, t_R = 19.5 min (major), 25.0 min (minor).

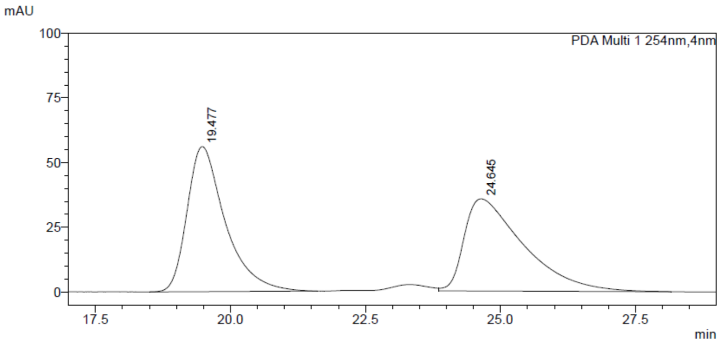

PDA Ch1 254nm

Peak#	Ret. Time	Area	Height	Aera%
1	19.477	2761726	56068	50.345
2	24.645	2723920	35610	49.655

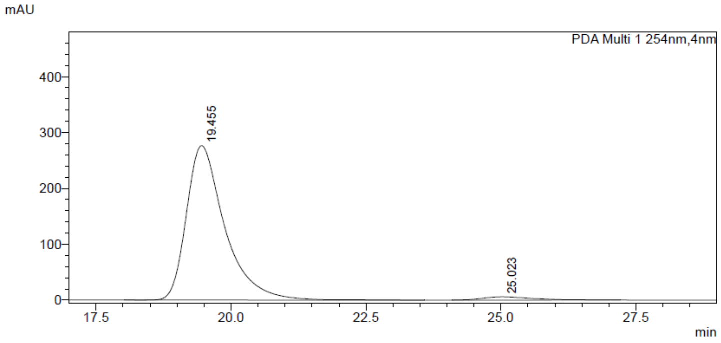

PDA Ch1 254nm

Peak#	Ret. Time	Area	Height	Aera%
1	19.455	13685874	276374	97.342
2	25.023	373721	5791	2.658

Supplementary Figure 27. HPLC Spectra of compound **4i** (Method A)

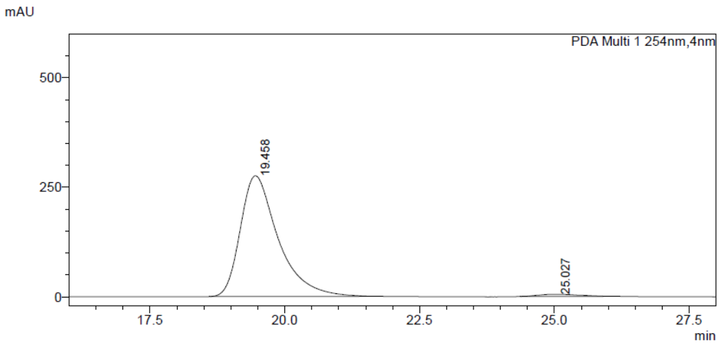

PDA Ch1 254nm

Peak#	Ret. Time	Area	Height	Aera%
1	19.458	13524599	275831	98.104
2	25.027	261438	4991	1.896

Supplementary Figure 28. HPLC Spectra of compound **4i** (Method B)

(*R*)-3-((3-Bromophenyl)sulfonyl)-2-methyl-*N*-phenyl-2-(*p*-tolyl)propanamide (4j)

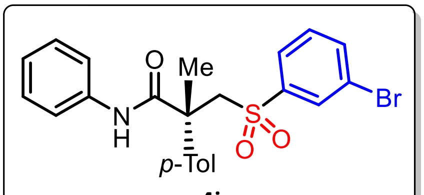

4j

Method A: 61% yield, 94% ee.

^{1}H NMR (400 MHz, $CDCl_3$) δ 7.58 – 7.50 (m, 3H), 7.30 – 7.18 (m, 5H), 7.12 – 7.06 (m, 3H), 7.00 (d, *J* = 8.0 Hz, 2H), 6.82 (s, 1H), 4.09 (d, *J* = 15.2 Hz, 1H), 3.97 (d, *J* = 15.2 Hz, 1H), 2.31 (s, 3H), 2.09 (s, 3H). **^{13}C NMR** (100 MHz, $CDCl_3$) δ 173.0, 142.4, 138.4, 137.3, 135.8, 135.1, 130.6, 130.4, 129.7, 129.0, 127.2, 126.0, 124.8, 122.8, 120.1, 64.0, 49.9, 22.6, 21.2. **HRMS** (ESI) m/z Calcd for $[C_{23}H_{22}BrNNaO_3S, M+Na]^+$: 494.0396 (496.0376), found: 494.0400 (496.0382). $[\alpha]_D^{20}$ = -73 (c= 0.11, EtOAc).

HPLC analysis: IA column, *n*-hexane/*i*-PrOH = 90/10, flow rate = 1.0 mL·min^{-1}, λ = 254 nm, t_R = 11.1 min (minor), 16.4 min (major).

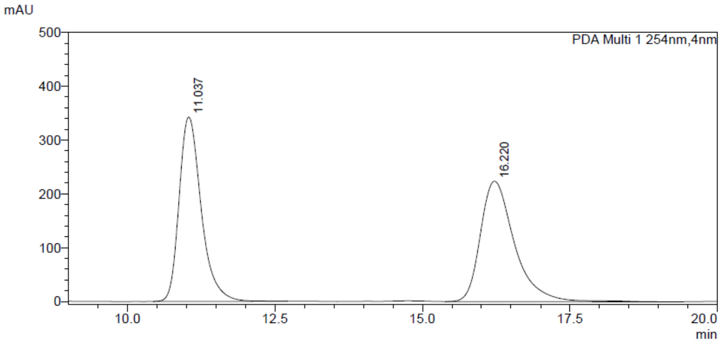

PDA Ch1 254nm

Peak#	Ret. Time	Area	Height	Aera%
1	11.037	8918199	342437	49.829
2	16.220	8979388	223537	50.171

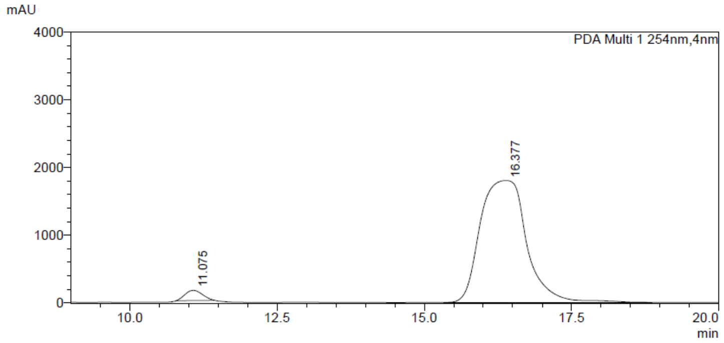

PDA Ch1 254nm

Peak#	Ret. Time	Area	Height	Aera%
1	11.075	3086657	149886	3.034
2	16.377	98639530	1801478	96.966

Supplementary Figure 29. HPLC Spectra of compound **4j** (Method A)

(*R*)-3-((3-(Benzyloxy)phenyl)sulfonyl)-2-methyl-*N*-phenyl-2-(*p*-tolyl)propanamide (4k)

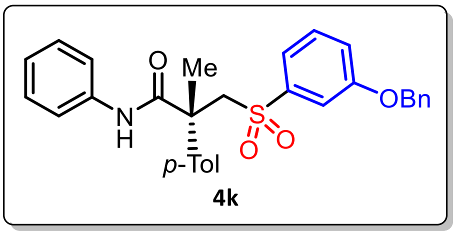

Method A: 35% yield, 96% ee.

^{1}H NMR (400 MHz, $CDCl_3$) δ7.42 – 7.37 (m, 4H), 7.33 – 7.30 (m, 3H), 7.26 – 7.20 (m, 4H), 7.12 – 7.06 (m, 5H), 6.98 (d, J = 8.0 Hz, 2H), 6.93 (s, 1H), 4.98 (s, 2H), 4.11 (d, J = 15.0 Hz, 1H), 3.89 (d, J = 15.0 Hz, 1H), 2.26 (s, 3H), 2.07 (s, 3H). **^{13}C NMR** (100 MHz, $CDCl_3$) δ 173.0, 158.7, 142.0, 138.0, 137.4, 136.0, 135.9, 130.1, 129.6, 129.0, 128.8, 128.4, 127.6, 127.1, 124.7, 120.4, 120.2, 120.0, 112.8, 70.2, 64.0, 50.0, 22.7, 21.0. **HRMS** (ESI) m/z Calcd for $[C_{30}H_{29}NNaO_4S, M+Na]^+$: 522.1710, found: 522.1714. $[\alpha]_D^{20}$ = -40 (c= 0.6, EtOAc).

HPLC analysis: IA column, *n*-hexane/*i*-PrOH = 90/10, flow rate = 1.0 mL·min^{-1}, λ = 254 nm, t_R = 13.3 min (minor), 21.9 min (major).

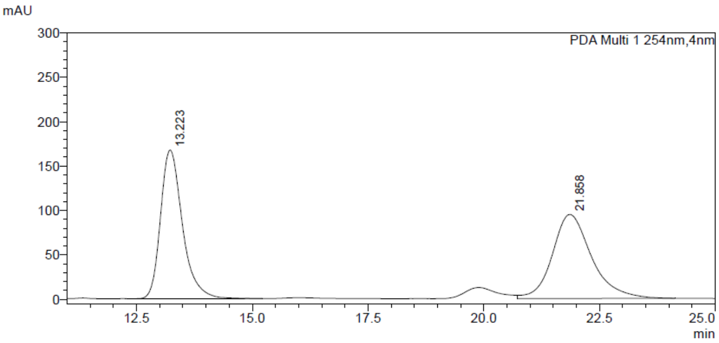

PDA Ch1 254nm

Peak#	Ret. Time	Area	Height	Aera%
1	13.223	5511972	167375	49.731
2	21.858	5571597	94593	50.269

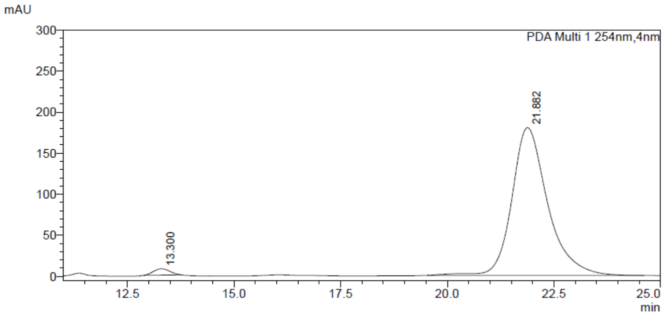

PDA Ch1 254nm

Peak#	Ret. Time	Area	Height	Aera%
1	13.300	204959	7718	1.908
2	21.882	10535799	180060	98.092

Supplementary Figure 30. HPLC Spectra of compound **4k** (Method A)

(*R*)-3-((2,3-Dihydrobenzofuran-5-yl)sulfonyl)-2-methyl-*N*-phenyl-2-(*p*-tolyl)propanamide (4l)

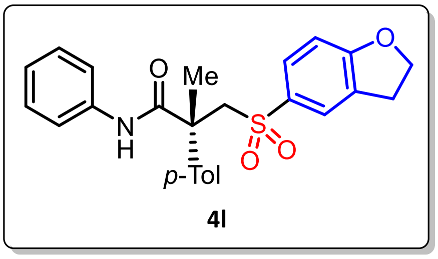

Method A: 74% yield, 96% ee; **Method B:** 61% yield, 95% ee.

^{1}H NMR (400 MHz, $CDCl_3$) δ 7.45 (dd, J = 8.4, 1.8 Hz, 1H), 7.39 (s, 1H), 7.32 – 7.30 (m, 2H), 7.28 – 7.24 (m, 2H), 7.19 (d, J = 8.2 Hz, 2H), 7.09 – 7.06 (m, 3H), 6.95 (s, 1H), 6.70 (d, J = 8.4 Hz, 1H), 4.62 (t, J = 8.8 Hz, 2H), 4.14 (d, J = 14.8 Hz, 1H), 3.80 (d, J = 14.8 Hz, 1H), 3.12 (t, J = 8.7 Hz, 2H), 2.32 (s, 3H), 2.09 (s, 3H). **^{13}C NMR** (100 MHz, $CDCl_3$) δ 172.9, 164.2, 137.8, 137.4, 136.5, 132.6, 129.6, 129.3, 128.9, 128.1, 127.0, 124.9, 124.6, 120.1, 109.4, 72.4, 64.2, 50.0, 28.8, 22.7, 21.0. **HRMS** (ESI) m/z Calcd for $[C_{25}H_{25}NNaO_4S, M+Na]^+$: 458.1397, found: 458.1397. $[\alpha]_D^{20}$ = -24 (c= 0.6, EtOAc).

HPLC analysis: AD-H column, *n*-hexane/*i*-PrOH = 60/40, flow rate = 1.0 mL·min^{-1}, λ = 254 nm, t_R = 14.0 min (minor), 22.2 min (major).

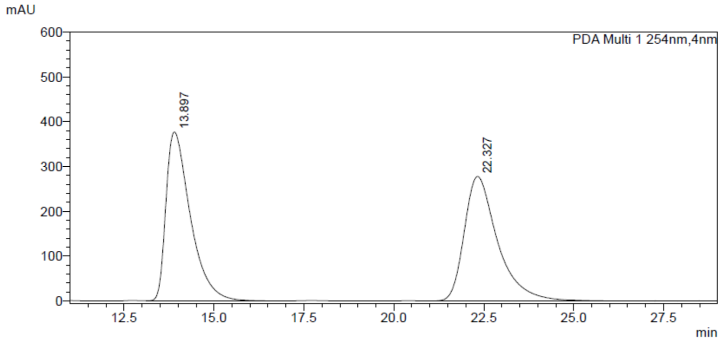

PDA Ch1 254nm

Peak#	Ret. Time	Area	Height	Aera%
1	13.897	17776564	376595	49.867
2	22.327	17871192	277365	50.133

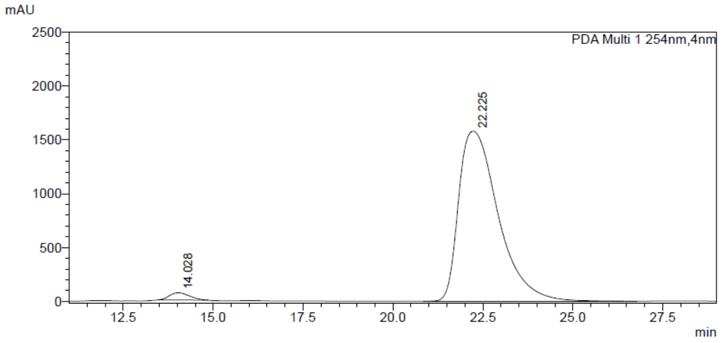

PDA Ch1 254nm

Peak#	Ret. Time	Area	Height	Aera%
1	14.028	2615576	67307	2.120
2	22.225	120763616	1579448	97.880

Supplementary Figure 31. HPLC Spectra of compound **4l** (Method A)

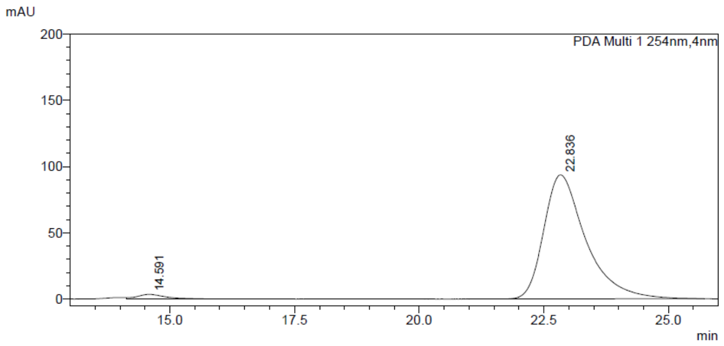

<Peak Table>

PDA Ch1 254nm

Peak#	Ret. Time	Area	Height	Aera%
1	14.591	130328	3421	2.317
2	22.836	5495050	93614	97.683

Supplementary Figure 32. HPLC Spectra of compound **4l** (Method B)

(*R*)-2-Methyl-3-((4-methyl-2-oxo-2*H*-chromen-7-yl)sulfonyl)-*N*-phenyl-2-(*p*-tolyl) Propanamide (4m)

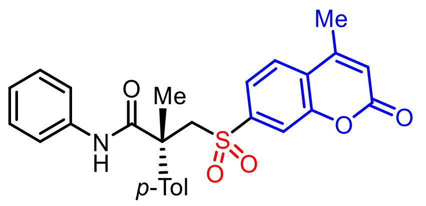

4m

Method A: 51% yield, 98% ee.

^{1}H NMR (400 MHz, $CDCl_3$) δ 7.60 – 7.55 (m, 2H), 7.29 – 7.22 (m, 5H), 7.12 (d, *J* = 8.2 Hz, 2H), 7.09 – 7.05 (m, 1H), 6.96 (d, *J* = 8.0 Hz, 2H), 6.88 (s, 1H), 6.38 (d, *J* = 1.0 Hz, 1H), 4.14 (d, *J* = 15.2 Hz, 1H), 3.99 (d, *J* = 15.2 Hz, 1H), 2.45 (d, *J* = 0.8 Hz, 3H), 2.21 (s, 3H), 2.11 (s, 3H). **^{13}C NMR** (100 MHz, $CDCl_3$) δ 172.7, 159.4, 152.7, 151.1, 143.3, 138.4, 137.2, 135.5, 129.6, 128.9, 127.1, 125.4, 124.8, 123.2, 122.7, 120.1, 117.6, 116.8, 64.2, 49.9, 22.6, 20.9, 18.8. **HRMS** (ESI) m/z Calcd for $[C_{27}H_{25}NNaO_5S, M+Na]^+$: 498.1346, found: 498.1346. $[\alpha]_D^{20}$ = -118 (c= 0.1, EtOAc).

HPLC analysis: AD-H column, *n*-hexane/*i*-PrOH = 60/40, flow rate = 1.0 mL·min^{-1}, λ = 254 nm, t_R = 28.4 min (major), 42.5 min (minor).

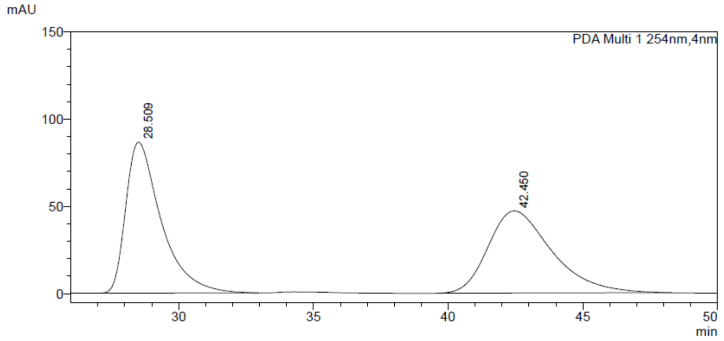

PDA Ch1 254nm

Peak#	Ret. Time	Area	Height	Aera%
1	28.509	7921218	86491	50.531
2	42.450	7754595	47020	49.469

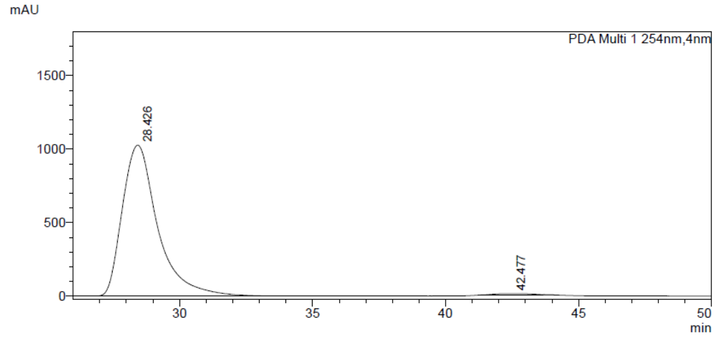

PDA Ch1 254nm

Peak#	Ret. Time	Area	Height	Aera%
1	28.426	94664546	1026753	98.988
2	42.477	967920	9637	1.012

Supplementary Figure 33. HPLC Spectra of compound **4m** (Method A)

(*R*)-2-Methyl-3-(naphthalen-1-ylsulfonyl)-*N*-phenyl-2-(*p*-tolyl)propanamide (4n)

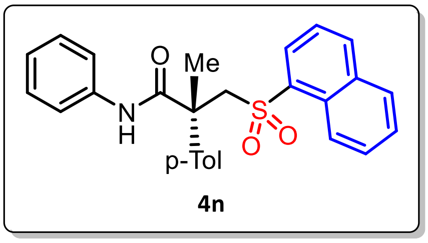

Method A: 59% yield, 86% ee.

^{1}H NMR (400 MHz, $CDCl_3$) δ 8.60 (d, *J* = 7.7 Hz, 1H), 7.95 – 7.78 (m, 3H), 7.66 – 7.57 (m, 2H), 7.28 – 7.24 (m, 5H), 7.04 – 6.97 (m, 3H), 6.81 – 6.80 (m, 3H), 4.28 (d, *J* = 14.8 Hz, 1H), 4.17 (d, *J* = 14.9 Hz, 1H), 2.22 (s, 3H), 2.13 (s, 3H). **^{13}C NMR** (100 MHz, $CDCl_3$) δ 173.2, 137.9, 137.3, 135.5, 135.4, 134.4, 133.9, 130.0, 129.3, 129.1, 128.9, 128.7, 128.3, 126.9, 126.8, 124.6, 124.2, 124.1, 120.0, 63.2, 50.1, 22.8, 21.0. **HRMS** (ESI) m/z Calcd for $[C_{27}H_{25}NNaO_3S, M+Na]^+$: 466.1447,found: 466.1450. $[\alpha]_D^{20}$ = -40 (c= 0.1, EtOAc).

HPLC analysis: IA column, *n*-hexane/*i*-PrOH = 90/10, flow rate = 1.0 mL·min^{-1}, λ = 254 nm, t_R = 11.3 min (minor), 16.5 min (major).

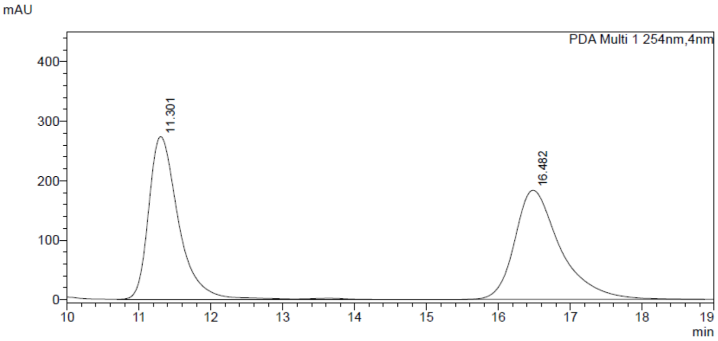

PDA Ch1 254nm

Peak#	Ret. Time	Area	Height	Aera%
1	11.301	7948417	273600	49.600
2	16.482	8076552	183414	50.400

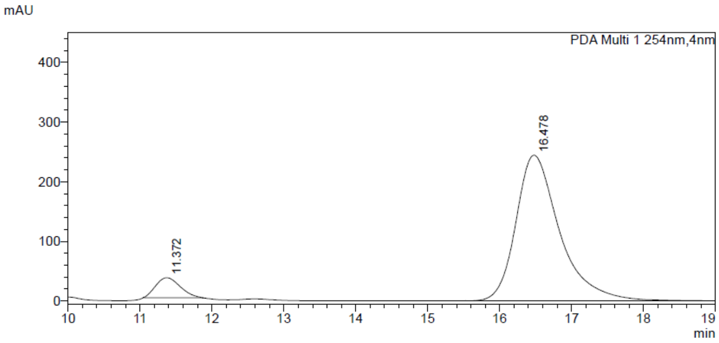

PDA Ch1 254nm

Peak#	Ret. Time	Area	Height	Aera%
1	11.372	797552	33561	7.182
2	16.478	10306776	244063	92.818

Supplementary Figure 34. HPLC Spectra of compound **4n** (Method A)

(*R*)-2-Methyl-3-((4-(methylthio)phenyl)sulfonyl)-*N*,2-di-*p*-tolylpropanamide (4o)

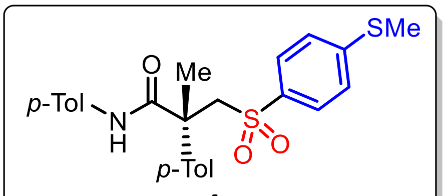

4o

Method A: 70% yield, 93% ee.

^{1}H NMR (400 MHz, $CDCl_3$) δ 7.45 – 7.43 (m, 2H), 7.18 – 7.08 (m, 10H), 6.86 (s, 1H), 4.11 (d, *J* = 14.9 Hz, 1H), 3.85 (d, *J* = 15.0 Hz, 1H), 2.48 (s, 3H), 2.31 (s, 3H), 2.27 (s, 3H), 2.07 (s, 3H). **^{13}C NMR** (100 MHz, $CDCl_3$) δ 172.8, 146.4, 138.0, 136.5, 136.1, 134.8, 134.4, 129.6, 129.4, 127.8, 127.0, 125.0, 120.2, 64.1, 49.9, 22.6, 21.1, 20.9, 14.8. **HRMS** (ESI) m/z Calcd for $[C_{25}H_{27}NNaO_3S_2, M+Na]^+$: 476.1325, found: 476.1329. $[\alpha]_D^{20}$ = -30 (c= 0.12, EtOAc).

HPLC analysis: AD-H column, *n*-hexane/*i*-PrOH = 60/40, flow rate = 1.0 mL·min^{-1}, λ = 254 nm, t_R = 30.9 min (minor), 61.0 min (major).

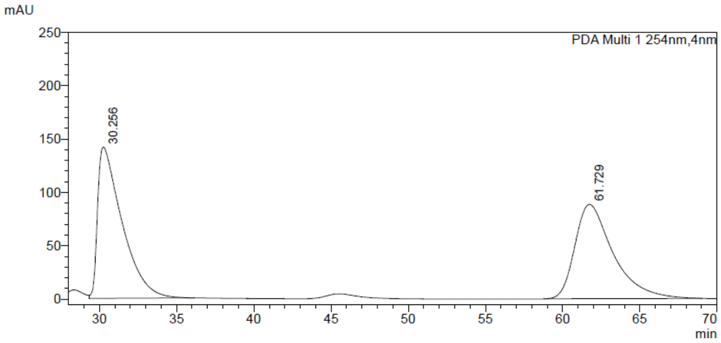

PDA Ch1 254nm

Peak#	Ret. Time	Area	Height	Aera%
1	30.256	15616845	141768	50.629
2	61.729	15228527	88367	49.371

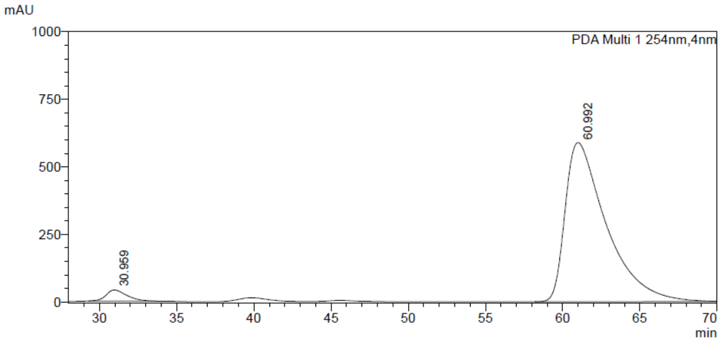

PDA Ch1 254nm

Peak#	Ret. Time	Area	Height	Aera%
1	30.959	3788090	41007	3.343
2	60.992	109526599	589375	96.657

Supplementary Figure 35. HPLC Spectra of compound **4o** (Method A)

(*R*)-3-((2-Chlorophenyl)sulfonyl)-2-methyl-*N*,2-di-*p*-tolylpropanamide (4p)

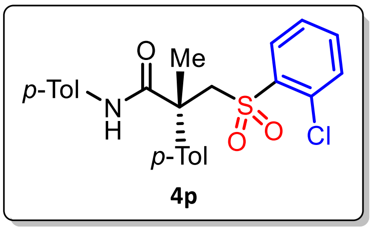

Method A: 63% yield, 94% ee.

^{1}H NMR (400 MHz, $CDCl_3$) δ 7.42 (dd, *J* = 7.9, 1.4 Hz, 1H), 7.33 – 7.25 (m, 2H), 7.09 – 7.04 (m, 5H), 6.97 (d, *J* = 8.3 Hz, 2H), 6.84 (d, *J* = 8.0 Hz, 2H), 6.67 (s, 1H), 4.41 (d, *J* = 15.5 Hz, 1H), 4.07 (d, *J* = 15.5 Hz, 1H), 2.19 (s, 3H), 2.14 (s, 3H), 2.00 (s, 3H). **^{13}C NMR** (100 MHz, $CDCl_3$) δ 173.2, 138.2, 137.9, 135.3, 134.7, 134.4, 133.6, 132.2, 131.4, 131.0, 129.5, 129.4, 127.2, 127.0, 120.1, 61.9, 49.9, 23.1, 21.0, 20.9. **HRMS** (ESI) m/z Calcd for $[C_{24}H_{25}ClNO_3S, M+H]^+$: 442.1238 (444.1209), found: 442.1249 (444.1238). $[\alpha]_D^{20}$ = -33 (c= 0.1, EtOAc).

HPLC analysis: IA column, *n*-hexane/*i*-PrOH = 90/10, flow rate = 1.0 mL·min^{-1}, λ = 254 nm, t_R = 17.3 min (minor), 25.7 min (major).

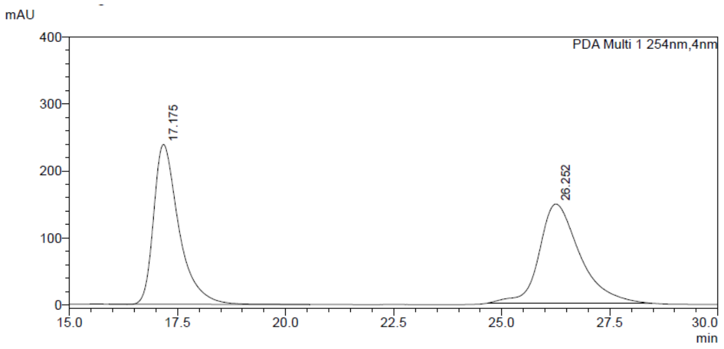

PDA Ch1 254nm

Peak#	Ret. Time	Area	Height	Aera%
1	17.175	9696693	238800	50.050
2	26.252	9677151	148022	49.950

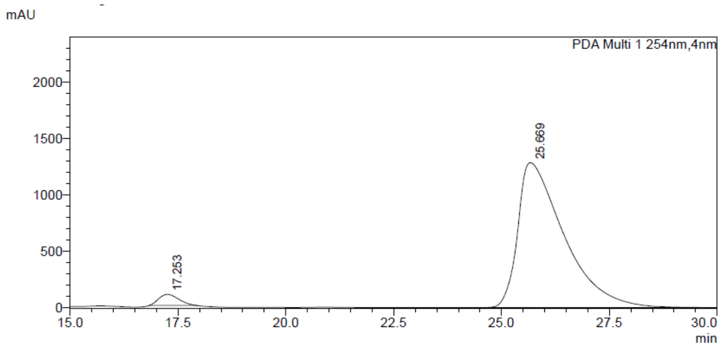

PDA Ch1 254nm

Peak#	Ret. Time	Area	Height	Aera%
1	17.253	3163075	97699	3.195
2	25.669	95841962	1285601	96.805

Supplementary Figure 36. HPLC Spectra of compound **4p** (Method A)

(*R*)-2-Methyl-*N*,2-di-*p*-tolyl-3-(m-tolylsulfonyl)propanamide (4q)

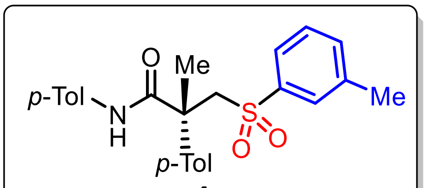

4q

Method A: 57% yield, 92% ee.

^{1}H NMR (400 MHz, $CDCl_3$) δ 7.37 (d, *J* = 7.5 Hz, 1H), 7.26 (s, 1H), 7.22 – 7.15 (m, 2H), 7.12 – 7.08 (m, 4H), 6.99 – 6.93 (m, 4H), 6.77 (s, 1H), 4.03 (d, *J* = 14.9 Hz, 1H), 3.80 (d, *J* = 14.9 Hz, 1H), 2.22 (s, 6H), 2.19 (s, 3H), 2.02 (s, 3H). **^{13}C NMR** (100 MHz, $CDCl_3$) δ 173.0, 140.8, 139.0, 137.9, 136.0, 134.8, 134.4, 133.7, 129.5, 129.4, 128.8, 128.0, 127.1, 124.6, 120.2, 63.9, 49.9, 22.7, 21.2, 21.1, 20.9. **HRMS** (ESI) m/z Calcd for $[C_{25}H_{27}NNaO_3S, M+Na]^+$: 444.1604, found: 444.1612. $[\alpha]_D^{25}$ = -52 (c= 0.16, EtOAc).

HPLC analysis: AD-H column, *n*-hexane/*i*-PrOH = 50/50, flow rate = 1.0 mL·min^{-1}, λ = 254 nm, t_R = 7.5 min (minor), 13.4 min (major).

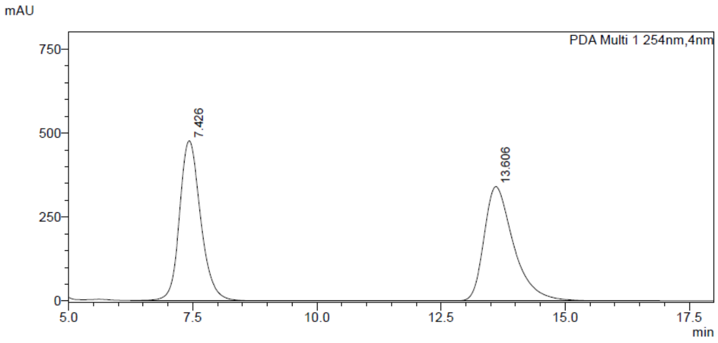

PDA Ch1 254nm

Peak#	Ret. Time	Area	Height	Aera%
1	7.426	14091925	476265	50.059
2	13.606	14058642	340114	49.941

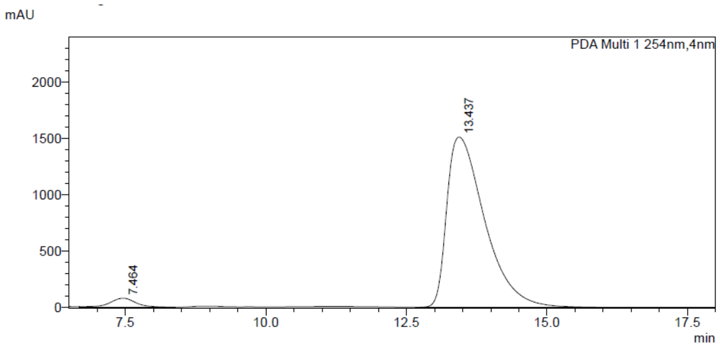

PDA Ch1 254nm

Peak#	Ret. Time	Area	Height	Aera%
1	7.464	2808724	81923	3.791
2	13.437	71277559	1512536	96.209

Supplementary Figure 37. HPLC Spectra of compound **4q** (Method A)

(*R*)-*N*-(4-Methoxyphenyl)-2-methyl-2-(*p*-tolyl)-3-tosylpropanamide (4r)

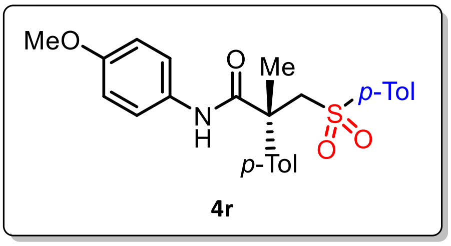

Method A: 59% yield, 98 % ee; **Method B:** 57% yield, 91% ee.

^{1}H NMR (400 MHz, $CDCl_3$) δ 7.48 (d, *J* = 8.1 Hz, 2H), 7.23 – 7.10 (m, 6H), 7.01 (d, *J* = 8.1 Hz, 2H), 6.95 (s, 1H), 6.77 (d, *J* = 8.9 Hz, 2H), 4.11 (d, *J* = 14.8 Hz, 1H), 3.81 (d, *J* = 14.8 Hz, 1H), 3.74 (s, 3H), 2.37 (s, 3H), 2.29 (s, 3H), 2.07 (s, 3H). **^{13}C NMR** (100 MHz, $CDCl_3$) δ 172.8, 156.7, 143.9, 138.1, 137.8, 136.5, 130.4, 129.5, 129.5, 127.6, 126.9, 122.3, 114.0, 64.1, 55.5, 49.8, 22.6, 21.6, 21.0. **HRMS** (ESI) m/z Calcd for $[C_{25}H_{27}NNaO_4S, M+Na]^+$: 460.1553, found: 460.1555. $[\alpha]_D^{20}$ = -34 (c= 1.0, EtOAc).

HPLC analysis: OD-H column, *n*-hexane/*i*-PrOH = 90/10, flow rate = 1.0 mL·min^{-1}, λ = 254 nm, t_R = 32.6 min (major), 40.3 min (minor).

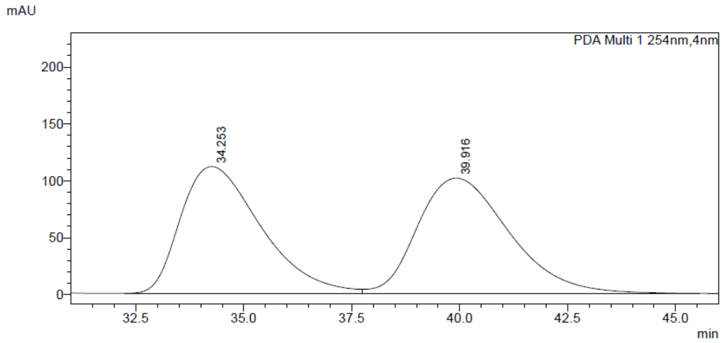

PDA Ch1 254nm

Peak#	Ret. Time	Area	Height	Aera%
1	34.253	14443805	111527	49.094
2	39.916	14976662	101322	50.906

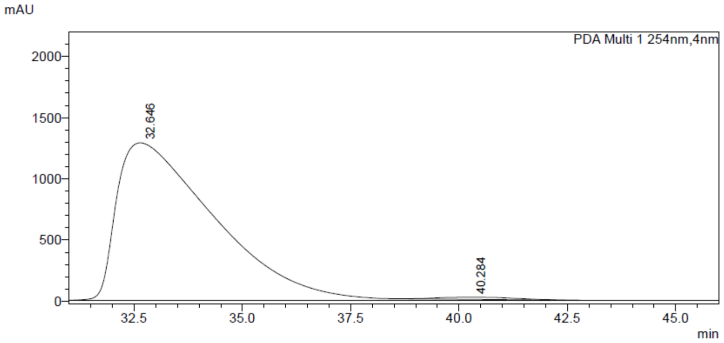

PDA Ch1 254nm

Peak#	Ret. Time	Area	Height	Aera%
1	32.646	204277861	1288143	98.874
2	40.284	2325645	19079	1.126

Supplementary Figure 38. HPLC Spectra of compound **4r** (Method A)

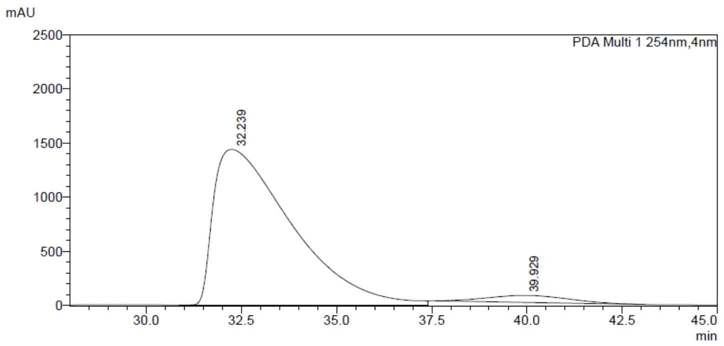

PDA Ch1 254nm

Peak#	Ret. Time	Area	Height	Aera%
1	32.239	206239290	1440947	95.581
2	39.929	9535639	63844	4.419

Supplementary Figure 39. HPLC Spectra of compound **4r** (Method B)

(*R*)-*N*-(4-Fluorophenyl)-2-methyl-2-(*p*-tolyl)-3-tosylpropanamide (4s)

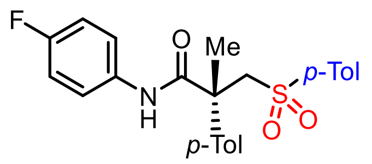

4s

Method A: 50% yield, 95% ee; **Method B:** 51% yield, 97% ee.

^{1}H NMR (400 MHz, CDCl3) δ 7.52 (d, *J* = 8.3 Hz, 2H), 7.30 – 7.25 (m, 2H), 7.19 – 7.16 (m, 4H), 7.05 (d, *J* = 8.1 Hz, 2H), 6.99 (s, 1H), 6.98 – 6.91 (m, 2H), 4.12 (d, *J* = 14.8 Hz, 1H), 3.78 (d, *J* = 14.8 Hz, 1H), 2.39 (s, 3H), 2.31 (s, 3H), 2.09 (s, 3H). **^{13}C NMR** (100 MHz, $CDCl_3$) δ 172.9, 159.6 (d, *J* = 243.9 Hz), 144.1, 138.1, 138.0, 136.3, 133.33 (d, *J* = 2.8 Hz), 129.6, 129.6, 127.6, 126.8, 122.23 (d, *J* = 8.0 Hz), 115.6 (d, *J* = 22.5 Hz), 64.1, 49.9, 22.6, 21.6, 21.0. **HRMS** (ESI) m/z Calcd for $[C_{24}H_{24}FNNaO_3S, M+Na]^+$: 448.1353, found: 448.1358. $[\alpha]_D^{20}$ = -4 (c= 0.11, EtOAc).

HPLC analysis: AS-H column, *n*-hexane/*i*-PrOH = 80/20, flow rate = 1.0 mL·min^{-1}, λ = 254 nm, t_R = 29.2 min (minor), 42.8 min (major).

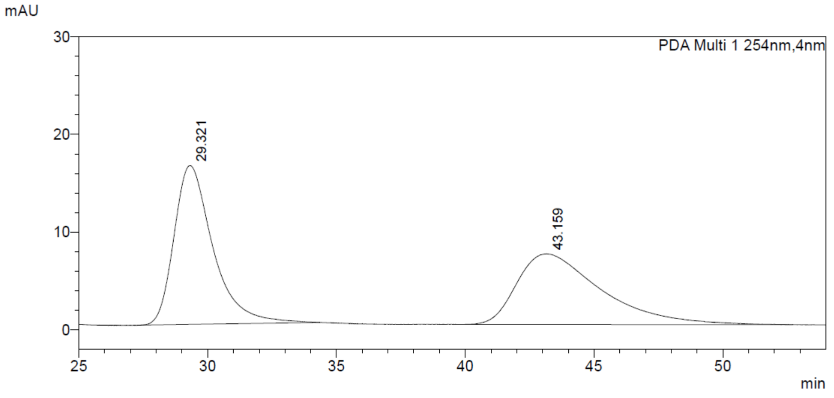

PDA Ch1 254nm

Peak#	Ret. Time	Area	Height	Aera%
1	29.321	1710997	16270	50.933
2	43.159	1648320	7249	49.067

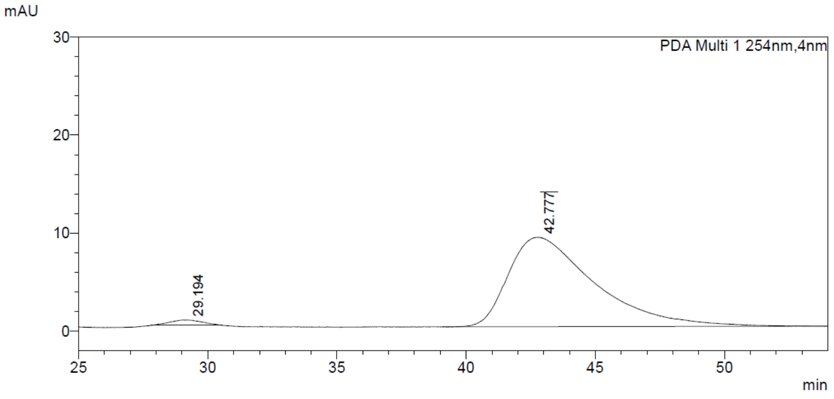

PDA Ch1 254nm

Peak#	Ret. Time	Area	Height	Aera%
1	29.194	49479	559	2.280
2	42.777	2120751	9121	97.720

Supplementary Figure 40. HPLC Spectra of compound **4s** (Method A)

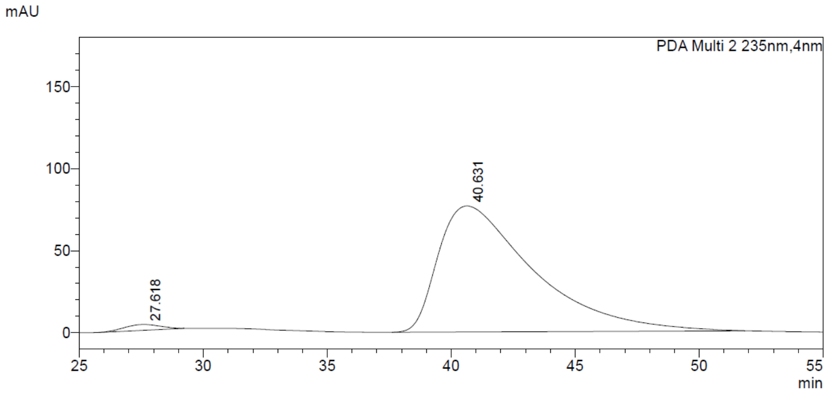

PDA Ch2 235nm

Peak#	Ret. Time	Area	Height	Aera%
1	27.618	358683	3525	1.713
2	40.631	20585651	76822	98.287

Supplementary Figure 41. HPLC Spectra of compound **4s** (Method B)

(*R*)-*N*-Benzyl-2-methyl-2-(*p*-tolyl)-3-tosylpropanamide (4t)

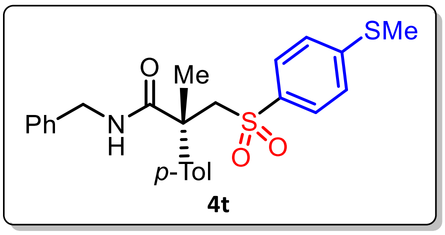

Method A: 45% yield, 92% ee.

^{1}H NMR (400 MHz, $CDCl_3$) δ 7.39 (d, *J* = 8.5 Hz, 2H), 7.21 – 7.15 (m, 3H), 7.06 – 7.00 (m, 6H), 6.91 (d, *J* = 8.0 Hz, 2H), 5.53 (t, *J* = 5.9 Hz, 1H), 4.33 (dd, *J* = 15.0, 5.9 Hz, 1H), 4.22 (dd, *J* = 15.0, 5.9 Hz, 1H), 4.05 (d, *J* = 14.8 Hz, 1H), 3.71 (d, *J* = 14.8 Hz, 1H), 2.41 (s, 3H), 2.21 (s, 3H), 1.93 (s, 3H). **^{13}C NMR** (100 MHz, $CDCl_3$) δ 174.7, 146.3, 138.0, 137.7, 136.8, 136.6, 129.4, 128.6, 127.8, 127.4, 127.4, 126.8, 125.0, 64.4, 49.1, 43.8, 22.5, 21.0, 14.8. **HRMS** (ESI) m/z Calcd for $[C_{25}H_{27}NNaO_3S_2, M+Na]^+$: 476.1325, found: 476.1321. $[\alpha]_D^{20}$ = 6 (c=0.1, EtOAc).

HPLC analysis: AD-H column, *n*-hexane/*i*-PrOH = 60/40, flow rate = 1.0 mL·min^{-1}, λ = 254 nm, t_R = 16.1 min (minor), 22.6 min (major).

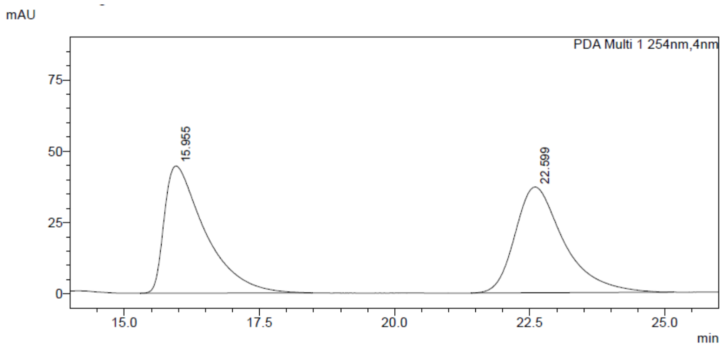

PDA Ch1 254nm

Peak#	Ret. Time	Area	Height	Aera%
1	15.955	2341664	44565	50.057
2	22.599	2336369	37074	49.943

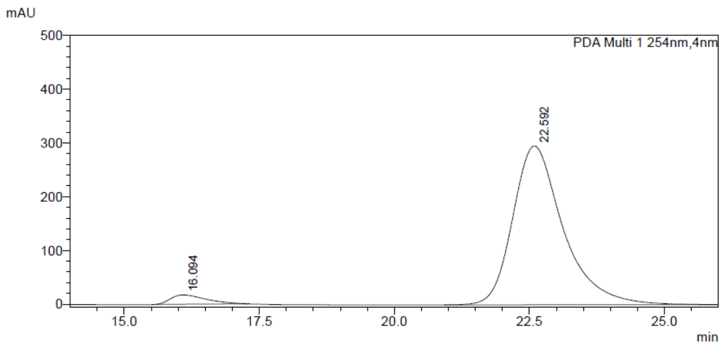

PDA Ch1 254nm

Peak#	Ret. Time	Area	Height	Aera%
1	16.094	790805	17286	3.948
2	22.592	19240951	295273	96.052

Supplementary Figure 42. HPLC Spectra of compound **4t** (Method A)

(*R*)-*N*-benzyl-2-(*p*-tolyl)-3-tosylpropanamide (4u)

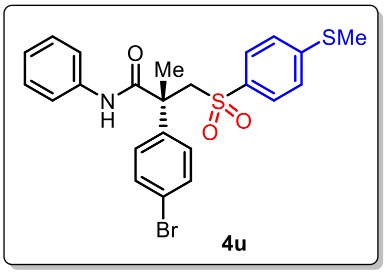

Method B: 46% yield, 97% ee.

^{1}H NMR (400 MHz, $CDCl_3$) δ 7.43 (d, *J* = 8.5 Hz, 2H), 7.35 – 7.25 (m, 6H), 7.15 – 7.08 (m, 5H), 6.87 (s, 1H), 4.07 (d, *J* = 15.0 Hz, 1H), 3.87 (d, *J* = 15.0 Hz, 1H), 2.52 (s, 3H), 2.10 (s, 3H). **^{13}C NMR** (100 MHz, $CDCl_3$) δ 172.0, 146.9, 138.1, 137.1, 136.1, 132.0, 129.0, 128.9, 127.7, 125.0, 125.0, 122.5, 120.2, 64.0, 49.9, 22.59, 14.8. **HRMS** (ESI) m/z Calcd for $[C_{23}H_{22}NNaO_3S_2$, $M+Na]^+$: 526.0122 (528.0102), found: 526.0158 (528.0135). $[\alpha]_D^{20}$ = -56 (c= 0.1, EtOAc).

HPLC analysis: IA column, *n*-hexane/*i*-PrOH = 60/40, flow rate = 1.0 mL·min^{-1}, λ = 254 nm, t_R = 22.0 min (minor), 34.5 min (major).

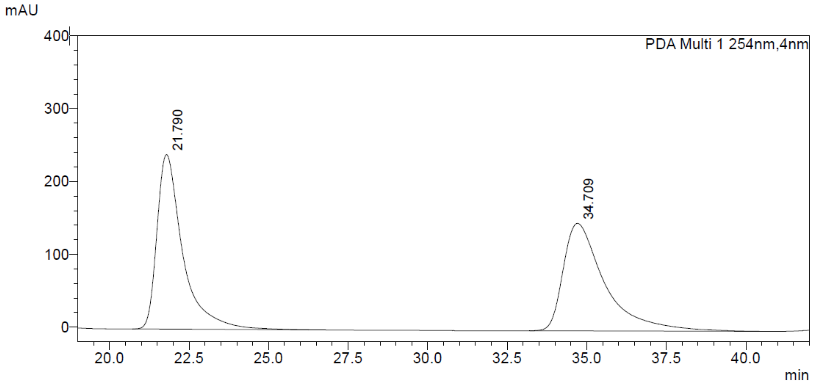

PDA Ch1 254nm

Peak#	Ret. Time	Area	Height	Aera%
1	21.790	13532965	239525	50.183
2	34.709	13434309	147452	49.817

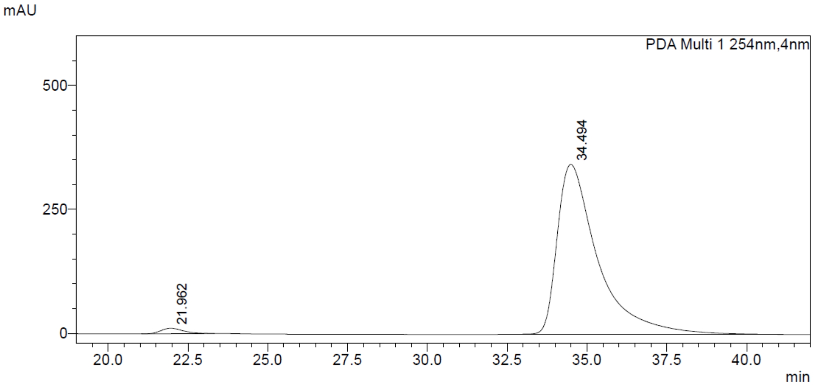

PDA Ch1 254nm

Peak#	Ret. Time	Area	Height	Aera%
1	21.962	539970	11126	1.740
2	34.494	30491964	342836	98.260

Supplementary Figure 43. HPLC Spectra of compound **4u** (Method B)

(*R*)-3-((2,4-Dimethylphenyl)sulfonyl)-2-methyl-*N*-phenyl-2-(*p*-tolyl)propanamide (4aa)

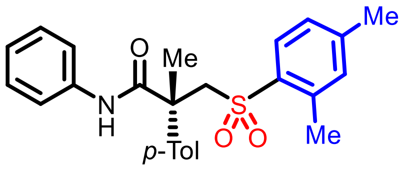

Method B: 81% yield, 91% ee.

^{1}H NMR (400 MHz, $CDCl_3$) δ 7.49 (d, *J* = 8.1 Hz, 1H), 7.31 – 7.23 (m, 4H), 7.19 (d, *J* = 8.0 Hz, 2H), 7.09 – 7.03 (m, 3H), 6.99 (s, 1H), 6.93 (d, *J* = 6.7 Hz, 2H), 4.12 (d, *J* = 14.8 Hz, 1H), 3.83 (d, *J* = 14.8 Hz, 1H), 2.59 (s, 3H), 2.31 (d, *J* = 8.1 Hz, 6H), 2.09 (s, 3H). **^{13}C NMR** (100 MHz, $CDCl_3$) δ 173.0, 144.0, 138.0, 137.4, 137.3, 136.3, 136.1, 133.1, 129.8, 129.6, 128.9, 126.9, 126.8, 124.6, 120.1, 62.9, 50.0, 22.8, 21.3, 21.1, 20.3. **HRMS** (ESI) m/z Calcd for $[C_{25}H_{27}NNaO_3S, M+Na]^+$: 444.1604, found: 444.1607. $[\alpha]_D^{20}$ = -35 (c= 0.1, EtOAc).

HPLC analysis: AD-H column, *n*-hexane/*i*-PrOH = 70/30, flow rate = 1.0 mL·min^{-1}, λ = 254 nm, t_R = 22.3 min (minor), 27.5 min (major).

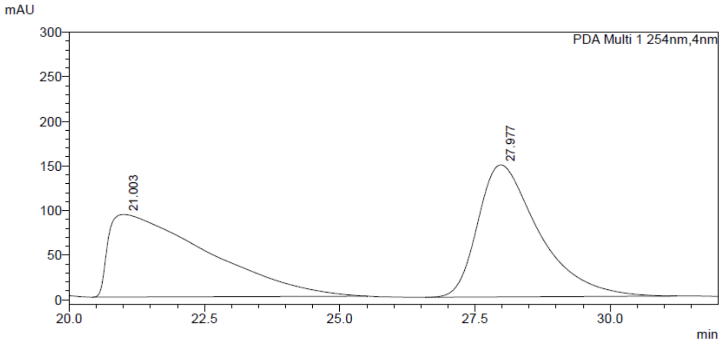

PDA Ch1 254nm

Peak#	Ret. Time	Area	Height	Aera%
1	21.003	11873459	92528	50.521
2	27.977	11628399	148032	49.479

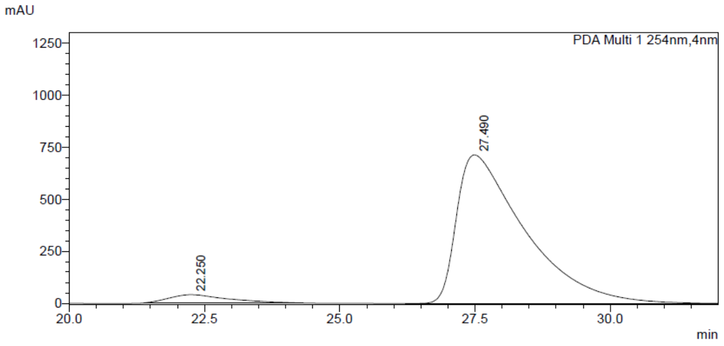

PDA Ch1 254nm

Peak#	Ret. Time	Area	Height	Aera%
1	22.250	2939768	39678	4.465
2	27.490	62895819	713656	95.535

Supplementary Figure 44. HPLC Spectra of compound **4aa** (Method B)

(*R*)-3-((3-Cyano-4-isobutoxyphenyl)sulfonyl)-2-methyl-*N*-phenyl-2-(*p*-tolyl)propanamide (4ab)

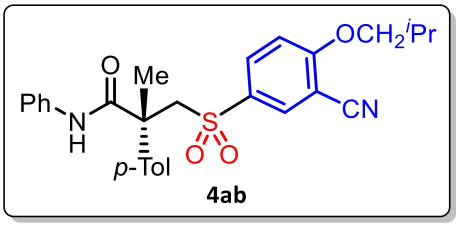

Method B: 58% yield, 93% ee.

^{1}H NMR (400 MHz, $CDCl_3$) δ 7.74 (dd, J = 8.9, 2.2 Hz, 1H), 7.44 (d, J = 2.2 Hz, 1H), 7.29 – 7.25 (m, 4H), 7.10 – 7.08 (m, 3H), 7.03 (d, J = 8.1 Hz, 2H), 6.86 (d, J = 8.9 Hz, 1H), 6.83 (s, 1H), 4.10 (s, 1H), 3.94 (d, J = 15.3 Hz, 1H), 3.85 (d, J = 6.4 Hz, 2H), 2.34 (s, 3H), 2.19 (dt, J = 13.2, 6.6 Hz, 1H), 2.09 (s, 3H), 1.12 – 1.07 (m, 6H). **^{13}C NMR** (100 MHz, $CDCl_3$) δ 172.9, 163.7, 138.6, 137.2, 135.2, 133.9, 133.8, 132.8, 129.8, 129.0, 127.2, 124.8, 120.1, 114.4, 112.0, 102.5, 76.0, 64.2, 49.8, 28.1, 22.5, 21.1, 19.0. **HRMS** (ESI) m/z Calcd for $[C_{28}H_{30}N_2NaO_4S, M+Na]^+$: 513.1818, found: 513.1824. $[\alpha]_D^{20}$ = -46 (c= 0.1, EtOAc).

HPLC analysis: AD-H column, *n*-hexane/*i*-PrOH = 70/30, flow rate = 1.0 mL·min^{-1}, λ = 254 nm, t_R = 17.3 min (minor), 20.0 min (major).

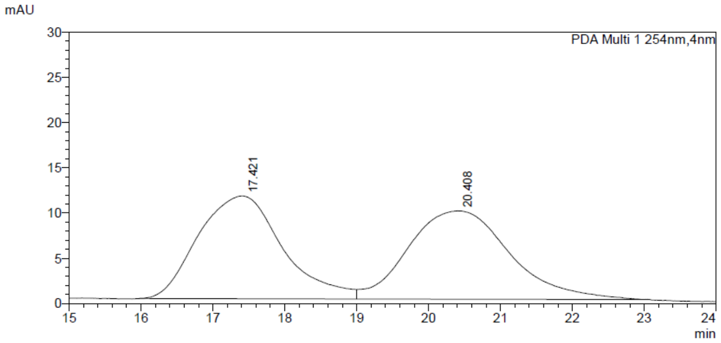

PDA Ch1 254nm

Peak#	Ret. Time	Area	Height	Aera%
1	17.421	928808	11351	49.237
2	20.408	957593	9764	50.763

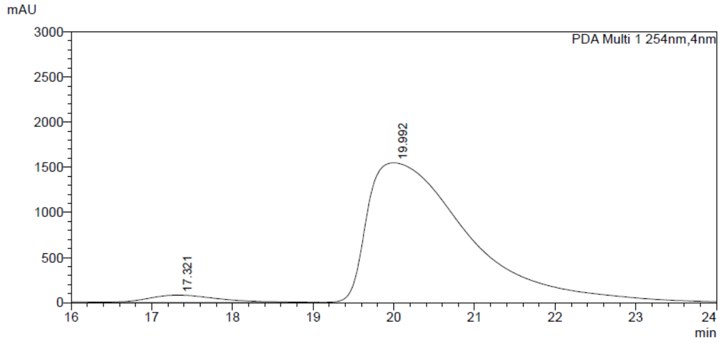

PDA Ch1 254nm

Peak#	Ret. Time	Area	Height	Aera%
1	17.321	5153263	82332	3.656
2	19.992	135812001	1548430	96.344

Supplementary Figure 45. HPLC Spectra of compound **4ab** (Method B)

(*R*)-2-Methyl-3-((4-phenoxyphenyl)sulfonyl)-*N*-phenyl-2-(*p*-tolyl)propanamide (4ac)

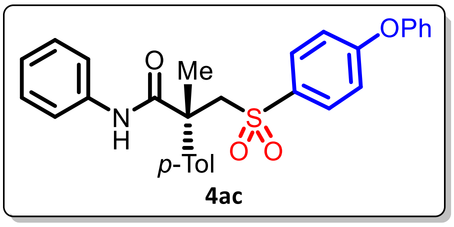

Method B: 58% yield, 96% ee.

^{1}H NMR (400 MHz, $CDCl_3$) δ 7.55 (d, *J* = 8.8 Hz, 2H), 7.43 – 7.39 (m, 2H), 7.32 (d, *J* = 7.6 Hz, 2H), 7.29 – 7.18 (m, 6H), 7.11 – 7.00 (m, 5H), 6.90 (s, 1H), 6.87 (d, *J* = 8.8 Hz, 2H), 4.14 (d, *J* = 14.9 Hz, 1H), 3.85 (d, *J* = 14.9 Hz, 1H), 2.32 (s, 3H), 2.11 (s, 3H). **^{13}C NMR** (100 MHz, $CDCl_3$) δ 172.9, 162.0, 155.0, 138.0, 137.4, 136.2, 134.6, 130.2, 129.8, 129.6, 128.9, 127.0, 125.0, 124.7, 120.3, 120.1, 117.3, 64.3, 50.0, 22.7, 21.1. **HRMS** (ESI) m/z Calcd for $[C_{29}H_{27}NNaO_4S, M+Na]^+$: 508.1553, found: 508.1562. $[\alpha]_D^{20}$ = -12 (c= 0.1, EtOAc).

HPLC analysis: IA column, *n*-hexane/*i*-PrOH = 90/10, flow rate = 1.0 mL·min^{-1}, λ = 254 nm, t_R = 19.7 min (minor), 25.0 min (major).

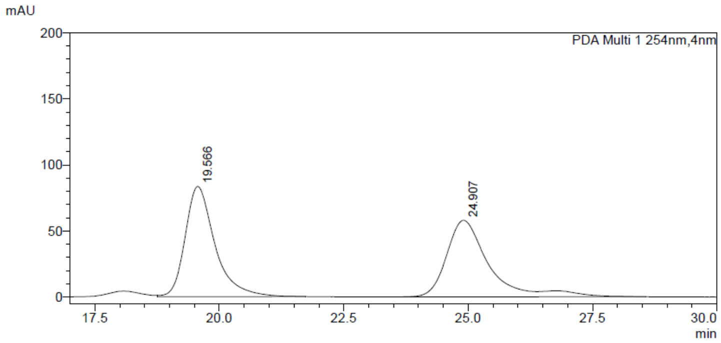

PDA Ch1 254nm

Peak#	Ret. Time	Area	Height	Aera%
1	19.566	3482281	83498	50.159
2	24.907	3460236	57951	49.841

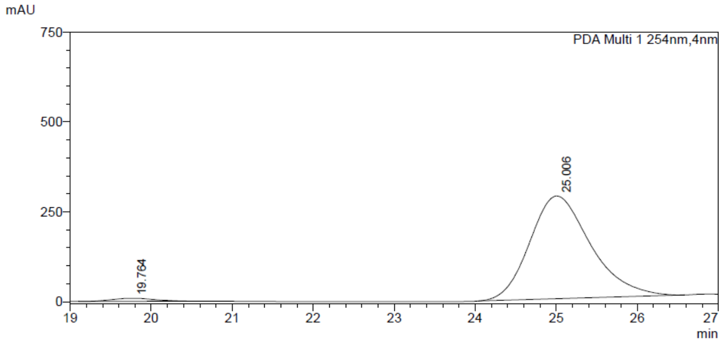

PDA Ch1 254nm

Peak#	Ret. Time	Area	Height	Aera%
1	19.764	315809	8694	2.068
2	25.006	14952606	285556	97.932

Supplementary Figure 46. HPLC Spectra of compound **4ac** (Method B)

(*R*)-2-Methyl-3-((4-(4-nitrophenoxy)phenyl)sulfonyl)-*N*-phenyl-2-(*p*-tolyl)propanamide (4ad)

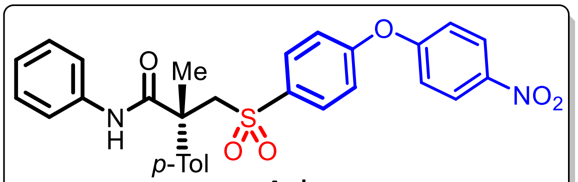

4ad

Method B: 45% yield, 95% ee.

^{1}H NMR (400 MHz, $CDCl_3$) δ 8.26 (d, *J* = 9.2 Hz, 2H), 7.70 (d, *J* = 8.8 Hz, 2H), 7.33 (d, *J* = 7.6 Hz, 2H), 7.30 – 7.20 (m, 4H), 7.12 - 7.07 (m, 5H), 7.03 (d, *J* = 8.7 Hz, 2H), 6.93 (s, 1H), 4.17 (d, *J* = 14.8 Hz, 1H), 3.86 (d, *J* = 14.8 Hz, 1H), 2.33 (s, 3H), 2.14 (s, 3H). **^{13}C NMR** (100 MHz, $CDCl_3$) δ 172.7, 161.2, 159.2, 143.8, 138.2, 137.3, 137.2, 136.3, 130.3, 129.7, 129.0, 127.0, 126.2, 124.8, 120.1, 119.5, 118.7, 64.4, 50.1, 22.7, 21.1. **HRMS** (ESI) m/z Calcd for $[C_{29}H_{26}N_2NaO_6S, M+Na]^+$: 553.1404, found: 553.1414. $[\alpha]_D^{20}$ = -3 (c= 0.1, EtOAc).

HPLC analysis: AD-H column, *n*-hexane/*i*-PrOH = 70/30, flow rate = 1.0 mL·min^{-1}, λ = 254 nm, t_R = 37.7 min (minor), 56.2 min (major).

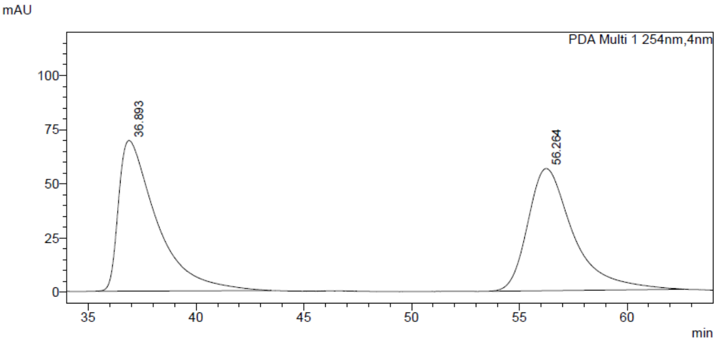

PDA Ch1 254nm

Peak#	Ret. Time	Area	Height	Aera%
1	36.893	8578124	69554	50.810
2	56.264	8304694	56399	49.190

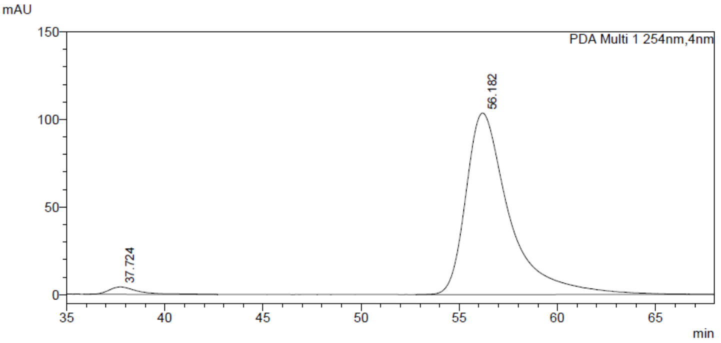

PDA Ch1 254nm

Peak#	Ret. Time	Area	Height	Aera%
1	37.724	433579	4173	2.630
2	56.182	16054825	103642	97.370

Supplementary Figure 47. HPLC data of **4ad** (Method B)

(*R*)-3-((4-Acetamidophenyl)sulfonyl)-2-methyl-*N*-phenyl-2-(*p*-tolyl)propanamide (4ae)

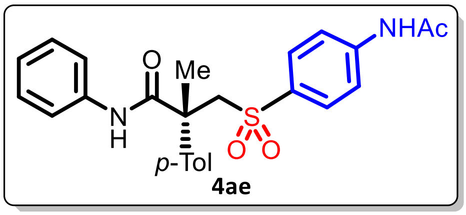

4ae

Method B: 61% yield, 96% ee.

^{1}H NMR (400 MHz, $CDCl_3$) δ 8.15 (s, 1H), 7.45-7.40 (m, 4H), 7.23 (d, *J* = 7.6 Hz, 2H), 7.19-7.15 (m, 2H), 7.09 (d, *J* = 8.2 Hz, 2H), 7.02 – 6.98 (m, 3H), 6.96 (s, 1H), 4.05 (d, *J* = 14.7 Hz, 1H), 3.73 (d, *J* = 14.8 Hz, 1H), 2.21 (s, 3H), 2.00 (d, *J* = 6.7 Hz, 6H). **^{13}C NMR** (100 MHz, $CDCl_3$) δ 172.0, 168.1, 141.8, 137.2, 136.1, 135.2, 134.0, 128.7, 127.9, 127.6, 125.7, 123.9, 119.4, 118.0, 63.1, 48.9, 23.5, 21.7, 19.9. **HRMS** (ESI) m/z Calcd for $[C_{25}H_{26}N_2NaO_4S, M+Na]^+$: 473.1505, found: 473.1518. $[\alpha]_D^{20}$ = 36 (c= 0.1, EtOAc).

HPLC analysis: AD-H column, *n*-hexane/*i*-PrOH = 70/30, flow rate = 1.0 mL·min^{-1}, λ = 254 nm, t_R = 7.8 min (minor), 18.5 min (major).

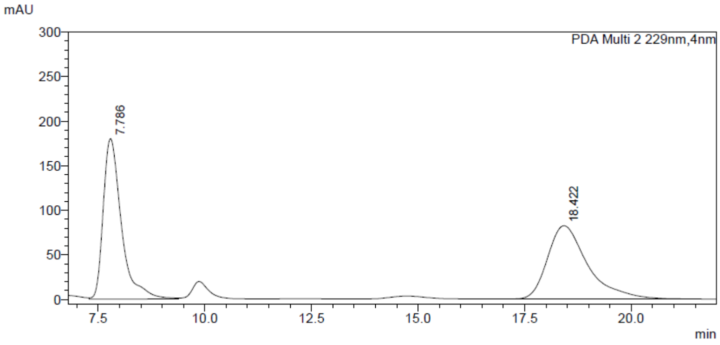

PDA Ch2 229nm

Peak#	Ret. Time	Area	Height	Aera%
1	7.786	5350647	180011	49.851
2	18.422	5382656	82258	50.149

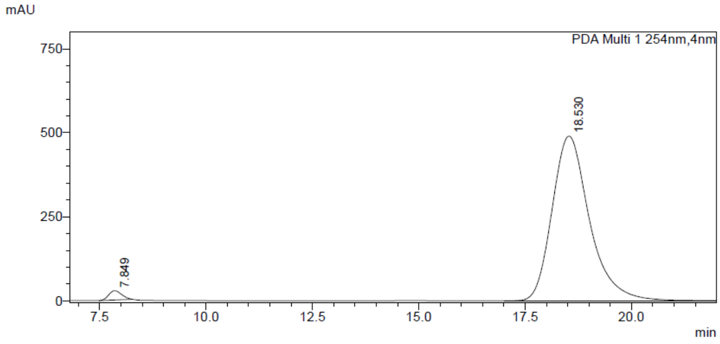

PDA Ch1 254nm

Peak#	Ret. Time	Area	Height	Aera%
1	7.849	576199	27418	1.887
2	18.530	29960274	490168	98.113

Supplementary Figure 48. HPLC Spectra of compound **4ae** (Method B)

(*R*)-2-Methyl-3-((4-(2-oxopyrrolidin-1-yl)phenyl)sulfonyl)-*N*-phenyl-2-(*p*-tolyl)propanamide(4af)

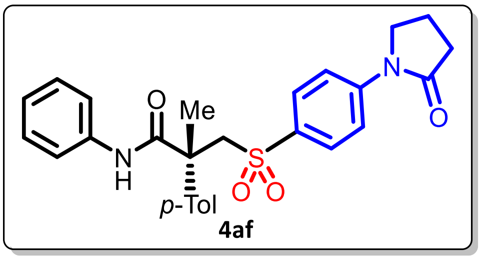

Method B: 66% yield, 97% ee.

^{1}H NMR (400 MHz, $CDCl_3$) δ 7.61 – 7.48 (m, 4H), 7.24 (d, *J* = 7.6 Hz, 2H), 7.21 – 7.14 (m, 2H), 7.11 (d, *J* = 8.2 Hz, 2H), 7.01 - 6.97 (m, 3H), 6.89 (s, 1H), 4.06 (d, *J* = 14.9 Hz, 1H), 3.85 – 3.68 (m, 3H), 2.55 (t, *J* = 8.1 Hz, 2H), 2.23 (s, 3H), 2.16 - 2.07 (m, 2H), 2.01 (s, 3H). **^{13}C NMR** (100 MHz, $CDCl_3$) δ 173.7, 171.8, 142.4, 137.0, 136.3, 135.2, 134.5, 128.6, 127.8, 127.5, 125.9, 123.6, 119.0, 117.7, 63.2, 49.0, 47.4, 31.8, 21.6, 20.0, 16.7. **HRMS** (ESI) m/z Calcd for $[C_{27}H_{28}N_2NaO_4S, M+Na]^+$: 499.1662, found: 499.1670. $[\alpha]_D^{20}$ = -26 (c= 0.1, EtOAc).

HPLC analysis: AD-H column, *n*-hexane/*i*-PrOH = 70/30, flow rate = 1.0 mL·min^{-1}, λ = 254 nm, t_R = 50.1 min (minor), 108.1 min (major).

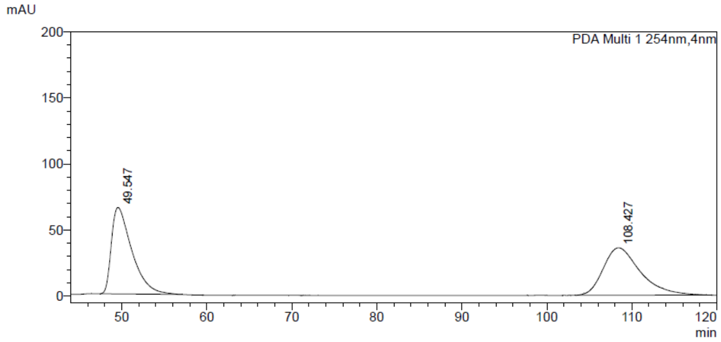

PDA Ch1 254nm

Peak#	Ret. Time	Area	Height	Aera%
1	49.547	10969108	65477	50.212
2	108.427	10876610	35878	49.788

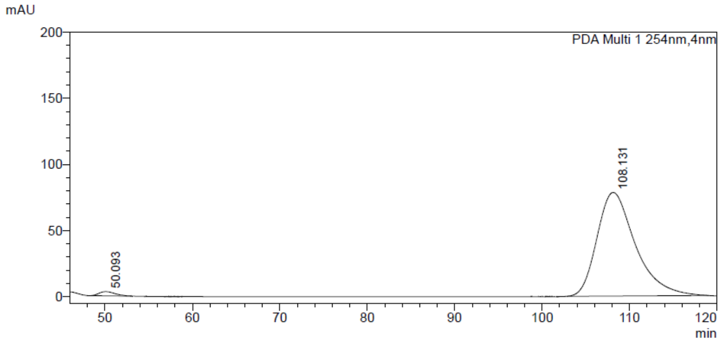

PDA Ch1 254nm

Peak#	Ret. Time	Area	Height	Aera%
1	50.093	402511	3194	1.654
2	108.131	23932812	78352	98.346

Supplementary Figure 49. HPLC Spectra of compound **4af** (Method B)

(*R*)-3-(Dibenzo[*b,d*]furan-2-ylsulfonyl)-2-methyl-*N*-phenyl-2-(*p*-tolyl)propanamide (4ag)

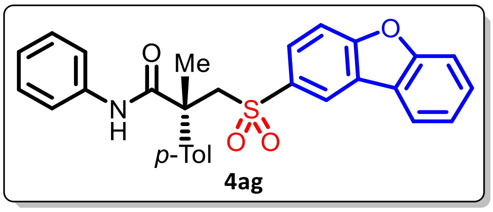

Method B: 60% yield, 96% ee.

^{1}H NMR (400 MHz, $CDCl_3$) δ 7.97 (d, J = 1.6 Hz, 1H), 7.88 (d, J = 7.7 Hz, 1H), 7.75 (dd, J = 8.7, 1.7 Hz, 1H), 7.63-7.57 (m, 1H), 7.51 (d, J = 8.7 Hz, 2H), 7.42 (t, J = 7.4 Hz, 1H), 7.28 – 7.21 (m, 5H), 7.08 (d, J = 8.2 Hz, 2H), 6.83 (d, J = 8.1 Hz, 2H), 6.77 (s, 1H), 4.20 (d, J = 15.2 Hz, 1H), 4.04 (d, J = 15.2 Hz, 1H), 2.13 (s, 3H), 1.80 (s, 3H). **^{13}C NMR** (100 MHz, $CDCl_3$) δ 173.1, 158.2, 156.9, 138.2, 137.3, 135.4, 135.1, 129.3, 128.9, 128.5, 127.1, 124.7, 123.7, 121.5, 121.2, 120.0, 64.2, 49.9, 22.7, 20.4. **HRMS** (ESI) m/z Calcd for [$C_{29}H_{25}NNaO_4S$, M+Na]$^+$: 506.1397, found: 506.1406. $[\alpha]_D^{20}$ = -71 (c= 0.1, EtOAc).

HPLC analysis: AD-H column, *n*-hexane/*i*-PrOH = 70/30, flow rate = 1.0 mL·min^{-1}, λ = 254 nm, t_R = 13.7 min (minor), 19.8 min (major).

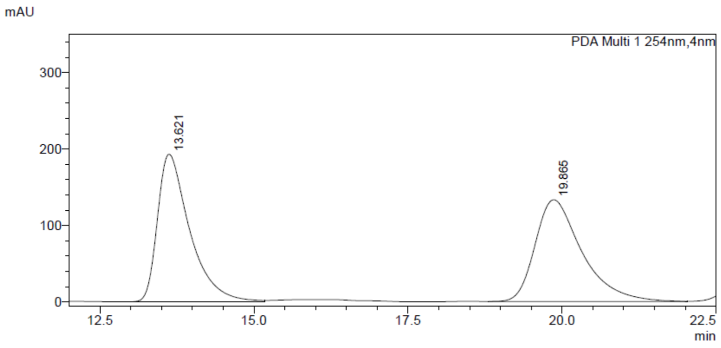

PDA Ch1 254nm

Peak#	Ret. Time	Area	Height	Aera%
1	13.621	7033100	192856	49.920
2	19.865	7055711	133055	50.080

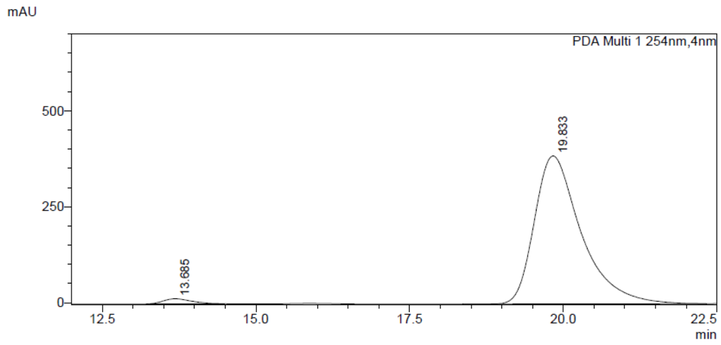

PDA Ch1 254nm

Peak#	Ret. Time	Area	Height	Aera%
1	13.685	406425	13064	1.950
2	19.833	20433924	385599	98.050

Supplementary Figure 50. HPLC Spectra of compound **4ag** (Method B)

(*R*)-2-Methyl-*N*,2-di-*p*-tolyl-3-tosylpropanamide (4ah)

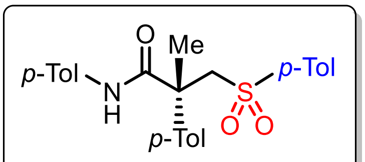

4ah

Method B: 79% yield, 91% ee.

^{1}H NMR (400 MHz, $CDCl_3$) δ 7.49 (d, *J* = 8.2 Hz, 2H), 7.19 - 7.13 (m, 6H), 7.06 - 7.02 (m, 4H), 6.89 (s, 1H), 4.11 (d, *J* = 14.9 Hz, 1H), 3.82 (d, *J* = 14.9 Hz, 1H), 2.37 (s, 3H), 2.30 (s, 3H), 2.27 (s, 3H), 2.08 (s, 3H). **^{13}C NMR** (100 MHz, $CDCl_3$) δ 172.8, 143.9, 138.1, 137.9, 136.3, 134.8, 134.3, 129.6, 129.5, 129.4, 127.6, 127.0, 120.2, 64.1, 49.9, 22.7, 21.6, 21.0, 20.9. **HRMS** (ESI) m/z Calcd for [$C_{25}H_{27}NNaO_3S$, M+Na]$^+$: 444.1604, found: 444.1606. $[\alpha]_D^{20}$ = 11 (c= 0.1, EtOAc). **HPLC analysis:** IA column, *n*-hexane/*i*-PrOH = 90/10, flow rate = 1.0 mL·min^{-1}, λ = 254 nm, t_R = 17.2 min (minor), 22.1 min (major).

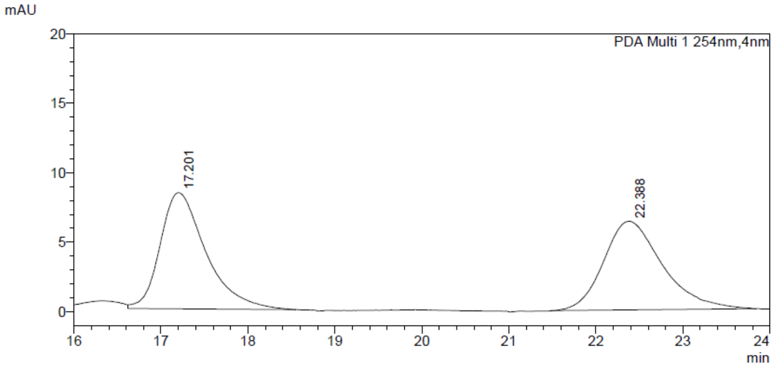

PDA Ch1 254nm

Peak#	Ret. Time	Area	Height	Aera%
1	17.201	305910	8359	50.414
2	22.388	300891	6381	49.586

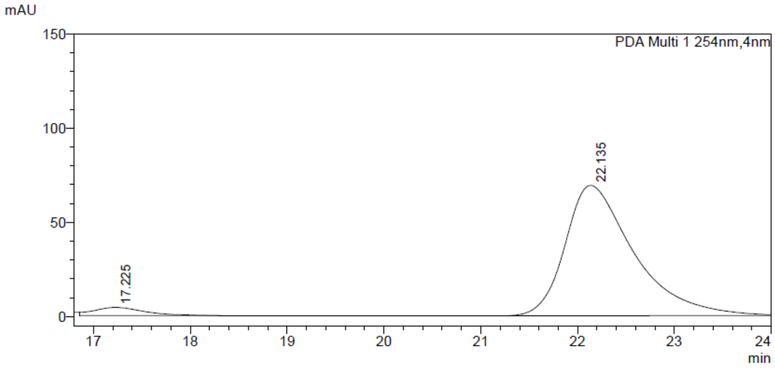

PDA Ch1 254nm

Peak#	Ret. Time	Area	Height	Aera%
1	17.225	160549	4339	4.479
2	22.135	3423653	69239	95.521

Supplementary Figure 51. HPLC Spectra of compound **4ah** (Method B)

(*R*)-*N*-Benzyl-2-methyl-2-(*p*-tolyl)-3-tosylpropanamide (4ai)

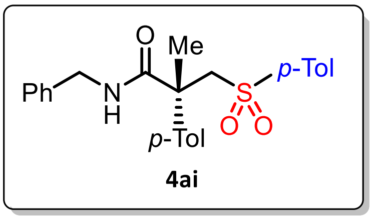

Method B: 64% yield, 91% ee.

^{1}H NMR (400 MHz, $CDCl_3$) δ 7.51 (d, *J* = 8.3 Hz, 2H), 7.29 – 7.21 (m, 3H), 7.16 (d, *J* = 8.0 Hz, 2H), 7.14 – 7.05 (m, 4H), 6.99 (d, *J* = 8.1 Hz, 2H), 5.67 (t, *J* = 5.6 Hz, 1H), 4.41 (dd, *J* = 15.0, 5.6 Hz, 1H), 4.29 (dd, *J* = 15.0, 5.6 Hz, 1H), 4.12 (d, *J* = 14.7 Hz, 1H), 3.75 (d, *J* = 14.7 Hz, 1H), 2.38 (s, 3H), 2.28 (s, 3H), 2.01 (s, 3H). **^{13}C NMR** (100 MHz, $CDCl_3$) δ 174.6, 143.9, 138.3, 138.0, 137.6, 136.8, 129.5, 129.4, 128.6, 127.6, 127.4, 126.8, 64.3, 49.1, 43.8, 22.5, 21.6, 21.0. **HRMS** (ESI) m/z Calcd for $[C_{25}H_{27}NNaO_3S, M+Na]^+$: 444.1604, found: 444.1602. $[\alpha]_D^{20}$ = -39 (c= 0.1, EtOAc).

HPLC analysis: AS-H column, *n*-hexane/*i*-PrOH = 80/20, flow rate = 1.0 mL·min^{-1}, λ = 254 nm, t_R = 22.4 min (minor), 28.9 min (major).

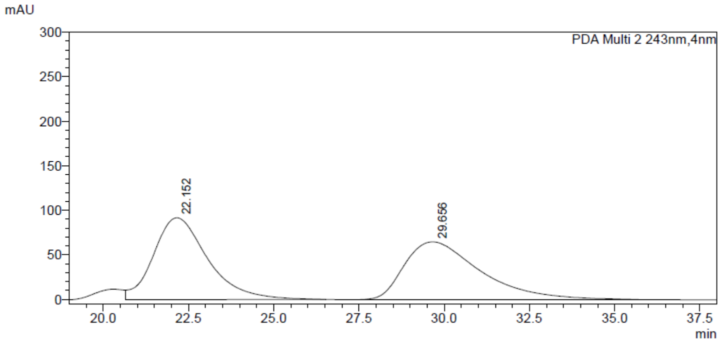

PDA Ch2 243nm

Peak#	Ret. Time	Area	Height	Aera%
1	22.152	10188783	91914	50.402
2	29.656	10026301	64754	49.598

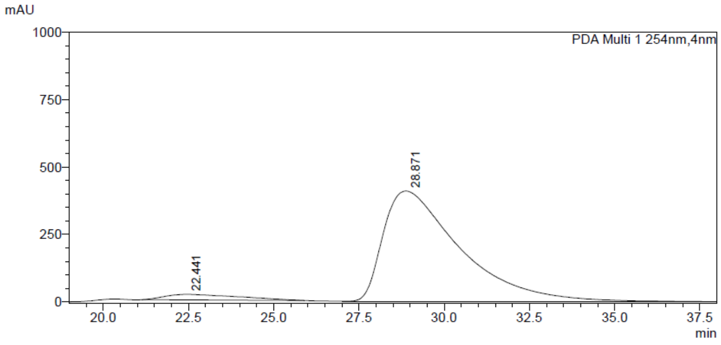

PDA Ch1 254nm

Peak#	Ret. Time	Area	Height	Aera%
1	22.441	3113104	20593	4.607
2	28.871	64460287	410414	95.393

Supplementary Figure 52. HPLC Spectra of compound **4ai** (Method B)

(*R*)-2-Methyl-3-(phenethylsulfonyl)-*N*-phenyl-2-(*p*-tolyl)propanamide (4aj)

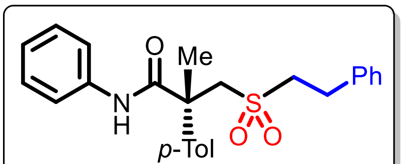

4aj

Method B: 60% yield, 79% ee.

^{1}H NMR (400 MHz, $CDCl_3$) δ 7.38 - 7.35 (m, 4H), 7.33 – 7.19 (m, 7H), 7.10 (t, *J* = 7.3 Hz, 1H), 7.03 (d, *J* = 7.1 Hz, 2H), 7.01 (s, 1H), 3.87 (d, *J* = 15.0 Hz, 1H), 3.62 (d, *J* = 15.0 Hz, 1H), 3.04 – 2.88 (m, 2H), 2.79 – 2.61 (m, 2H), 2.34 (s, 3H), 2.08 (s, 3H). **^{13}C NMR** (100 MHz, $CDCl_3$) δ 172.9, 138.6, 137.6, 137.3, 136.8, 130.0, 129.0, 128.8, 128.3, 127.1, 126.9, 124.8, 120.2, 61.5, 56.3, 50.0, 27.8, 22.8, 21.0. **HRMS** (ESI) m/z Calcd for $[C_{25}H_{27}NNaO_3S, M+Na]^+$: 444.1604, found: 444.1601. $[\alpha]_D^{20}$ = 4 (c= 0.7, EtOAc).

HPLC analysis: IA column, *n*-hexane/*i*-PrOH = 90/10, flow rate = 1.0 mL·min^{-1}, λ = 254 nm, t_R = 14.4 min (minor), 17.8 min (major).

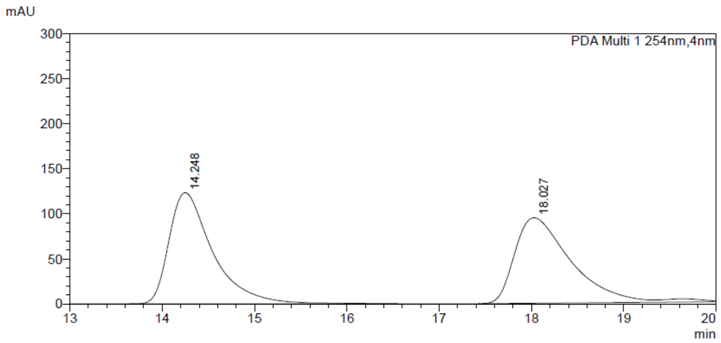

PDA Ch1 254nm

Peak#	Ret. Time	Area	Height	Aera%
1	14.248	4070358	123419	49.849
2	18.027	4095002	94947	50.151

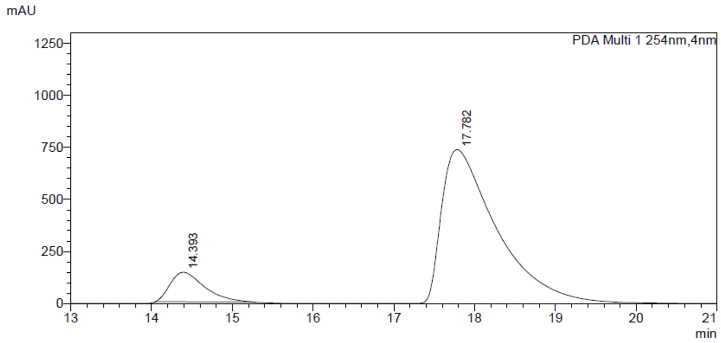

PDA Ch1 254nm

Peak#	Ret. Time	Area	Height	Aera%
1	14.393	4143984	141102	10.481
2	17.782	35393279	739296	89.519

Supplementary Figure 53. HPLC Spectra of compound **4aj** (Method B)

(*R*)-2-Methyl-*N*-phenyl-3-((4-phenylbutyl)sulfonyl)-2-(*p*-tolyl)propanamide (4ak)

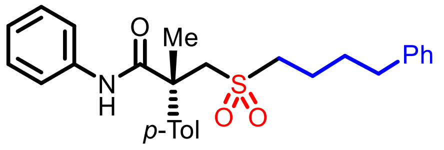

4ak

Method B: 75% yield, 81% ee.

^{1}H NMR (400 MHz, $CDCl_3$) δ 7.37 – 7.34 (m, 4H), 7.31 – 7.23 (m, 4H), 7.21 - 7.16 (m, 3H), 7.11 - 7.08 (m, 3H), 7.04 (s, 1H), 3.84 (d, J = 14.9 Hz, 1H), 3.59 (d, J = 14.9 Hz, 1H), 2.55 – 2.39 (m, 4H), 2.35 (s, 3H), 2.05 (s, 3H), 1.75 – 1.62 (m, 2H), 1.56 - 1.50 (m, 2H). **^{13}C NMR** (100 MHz, $CDCl_3$) δ 173.0, 141.4, 138.5, 137.4, 136.8, 130.0, 129.0, 128.5, 128.4, 127.0, 126.1, 124.8, 120.3, 61.0, 55.0, 50.0, 35.3, 30.2, 22.7, 21.5, 21.1. **HRMS** (ESI) m/z Calcd for $[C_{27}H_{31}NNaO_3S, M+Na]^+$: 472.1917, found: 472.1925. $[\alpha]_D^{20}$ = -36 (c= 0.2, EtOAc).

HPLC analysis: IA column, *n*-hexane/*i*-PrOH = 90/10, flow rate = 1.0 mL·min^{-1}, λ = 254 nm, t_R = 16.7 min (major), 23.1 min (minor).

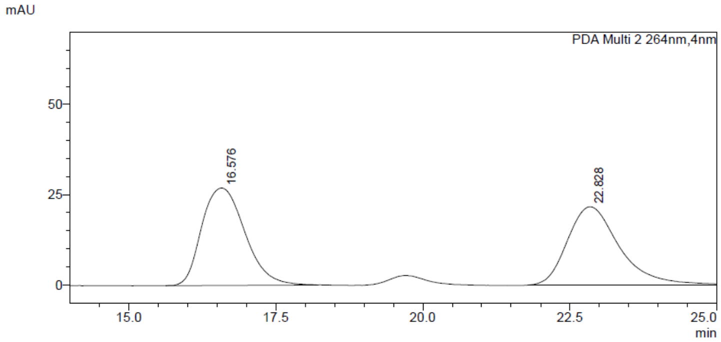

PDA Ch2 264nm

Peak#	Ret. Time	Area	Height	Aera%
1	16.576	1379986	26991	50.924
2	22.828	1329933	21606	49.076

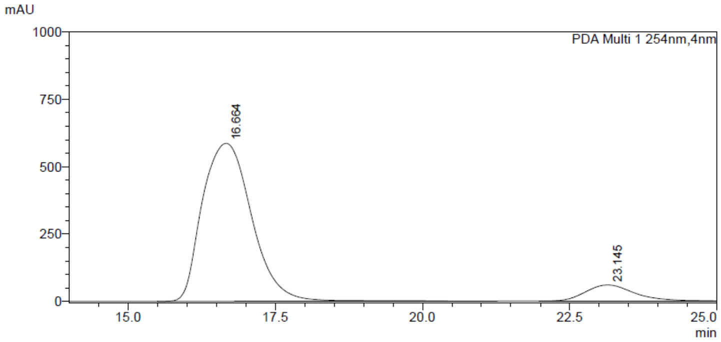

PDA Ch1 254nm

Peak#	Ret. Time	Area	Height	Aera%
1	16.664	34229756	586132	90.652
2	23.145	3529797	60053	9.348

Supplementary Figure 54. HPLC Spectra of compound **4ak** (Method B)

(*R*)-2-Methyl-*N*-phenyl-3-((2-(thiophen-2-yl)ethyl)sulfonyl)-2-(*p*-tolyl)propanamide (4al)

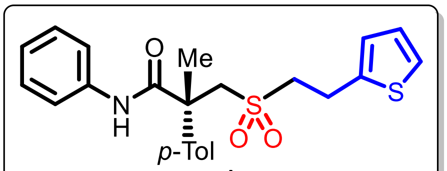

4al

Method B: 47% yield, 74% ee.

^{1}H NMR (400 MHz, $CDCl_3$) δ 7.37 - 7.35 (m, 4H), 7.31 – 7.21 (m, 4H), 7.16 – 7.07 (m, 2H), 7.00 (s, 1H), 6.89 (dd, *J* = 5.0, 3.5 Hz, 1H), 6.71 - 6.70 (m, 1H), 3.86 (d, *J* = 15.0 Hz, 1H), 3.63 (d, *J* = 15.0 Hz, 1H), 3.23 – 3.10 (m, 2H), 2.81 – 2.65 (m, 2H), 2.35 (s, 3H), 2.08 (s, 3H). **^{13}C NMR** (100 MHz, $CDCl_3$) δ 172.9, 139.7, 138.7, 137.3, 136.6, 130.1, 129.0, 127.0, 127.0, 125.5, 124.8, 124.3, 120.2, 61.6, 56.2, 50.0, 22.6, 22.4, 21.1. **HRMS** (ESI) m/z Calcd for $[C_{23}H_{25}NNaO_3S_2$, M+Na$]^+$: 450.1168, found: 450.1168. $[\alpha]_D^{20}$ = -20 (c= 0.7, EtOAc).

HPLC analysis: IA column, *n*-hexane/*i*-PrOH = 90/10, flow rate = 1.0 mL·min^{-1}, λ = 254 nm, t_R = 14.7 min (minor), 18.5 min (major).

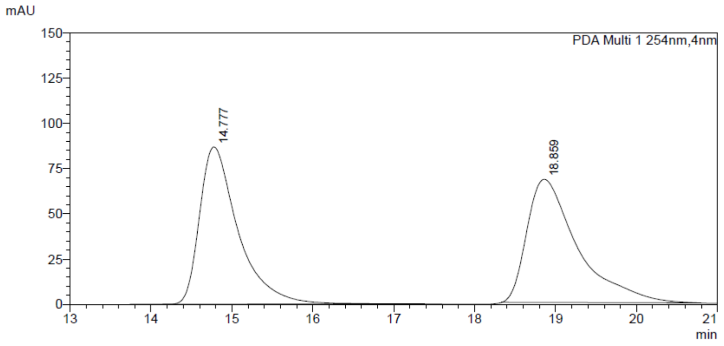

PDA Ch1 254nm

Peak#	Ret. Time	Area	Height	Aera%
1	14.777	2878410	86981	49.353
2	18.859	2953868	68033	50.647

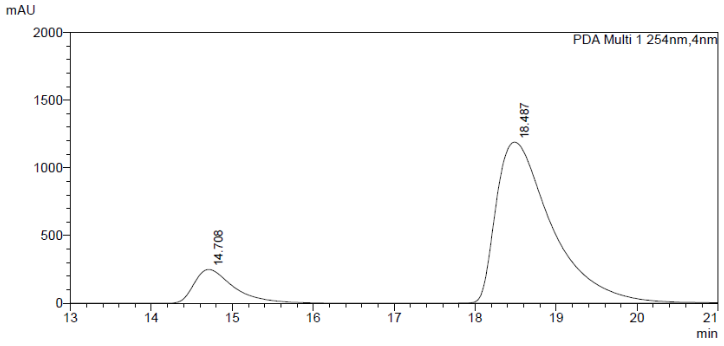

PDA Ch1 254nm

Peak#	Ret. Time	Area	Height	Aera%
1	14.708	8724071	249896	13.209
2	18.487	57320083	1189198	86.791

Supplementary Figure 55. HPLC Spectra of compound **4al** (Method B)

(*R*)-3-((3-Bromopropyl)sulfonyl)-2-methyl-*N*-phenyl-2-(*p*-tolyl)propanamide (4am)

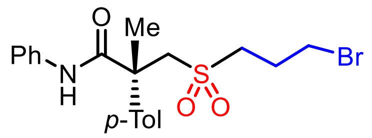

4am

Method B: 62% yield, 79% ee.

^{1}H NMR (400 MHz, $CDCl_3$) δ 7.37 – 7.35 (m, 4H), 7.32 – 7.20 (m, 4H), 7.11 (t, *J* = 7.3 Hz, 1H), 7.01 (s, 1H), 3.92 (d, *J* = 15.0 Hz, 1H), 3.63 (d, *J* = 15.0 Hz, 1H), 3.36 (t, *J* = 6.3 Hz, 2H), 2.77 – 2.56 (m, 2H), 2.37 (s, 3H), 2.30 – 2.15 (m, 2H), 2.07 (s, 3H). **^{13}C NMR** (100 MHz, $CDCl_3$) δ 172.8, 138.7, 137.2, 136.6, 130.1, 129.0, 126.9, 124.9, 120.3, 61.9, 53.6, 50.0, 31.1, 24.8, 22.6, 21.1. **HRMS** (ESI) m/z Calcd for $[C_{20}H_{24}BrNNaO_3S, M+Na]^+$: 460.0552 (462.0532), found: 460.0559 (462.0543). $[\alpha]_D^{20}$ = -10 (c= 0.1, EtOAc).

HPLC analysis: IA column, *n*-hexane/*i*-PrOH = 90/10, flow rate = 1.0 mL·min^{-1}, λ = 254 nm, t_R = 18.8 min (major), 22.5 min (minor).

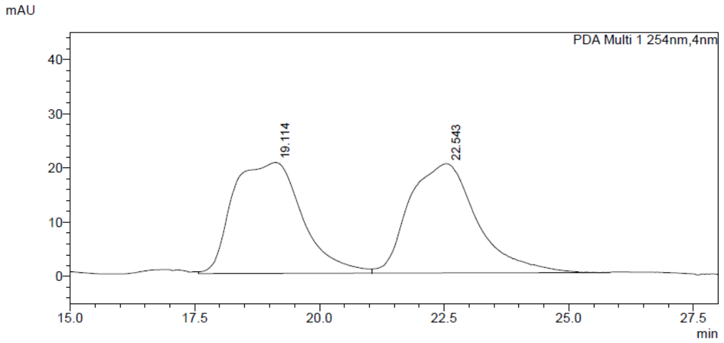

PDA Ch1 254nm

Peak#	Ret. Time	Area	Height	Aera%
1	19.114	1977049	20462	50.843
2	22.543	1911477	20152	49.157

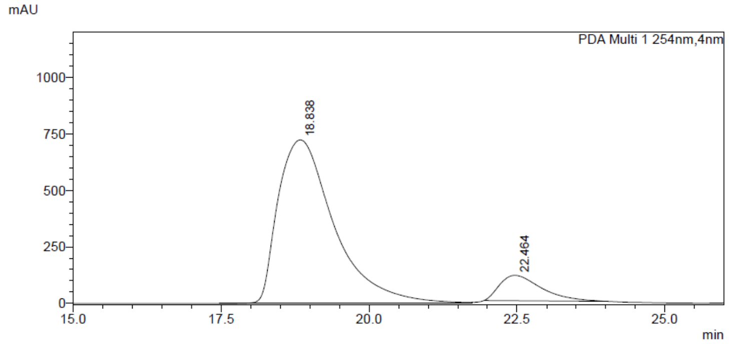

PDA Ch1 254nm

Peak#	Ret. Time	Area	Height	Aera%
1	18.838	47728455	722988	89.578
2	22.464	5552923	112231	10.422

Supplementary Figure 56. HPLC Spectra of compound **4am** (Method B)

(*R*)-2-(4-Bromophenyl)-2-methyl-*N*-phenyl-3-tosylpropanamide (4an)

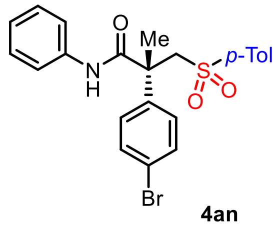

4an

Method B: 58% yield, 96% ee.

^{1}H NMR (400 MHz, $CDCl_3$) δ 7.47 (d, *J* = 8.2 Hz, 2H), 7.34 – 7.25 (m, 6H), 7.19 – 7.08 (m, 5H), 6.89 (s, 1H), 4.07 (d, *J* = 15.0 Hz, 1H), 3.84 (d, *J* = 15.0 Hz, 1H), 2.41 (s, 3H), 2.10 (s, 3H). **^{13}C NMR** (100 MHz, $CDCl_3$) δ 172.1, 144.4, 138.2, 137.7, 137.1, 132.0, 129.7, 129.0, 128.8, 127.5, 124.9, 122.6, 120.2, 63.8, 49.9, 22.5, 21.6. HRMS (ESI) m/z Calcd for [$C_{23}H_{22}BrNNaO_3S$, M+Na]+: 494.0401 (496.0381), found: 494.0398 (494.0378). $[\alpha]_D^{20}$ = -32 (c= 0.1, EtOAc).

HPLC analysis: IA column, *n*-hexane/*i*-PrOH = 80/20, flow rate = 1.0 mL·min^{-1}, λ = 254 nm, t_R = 18.7 min (minor), 23.8 min (major).

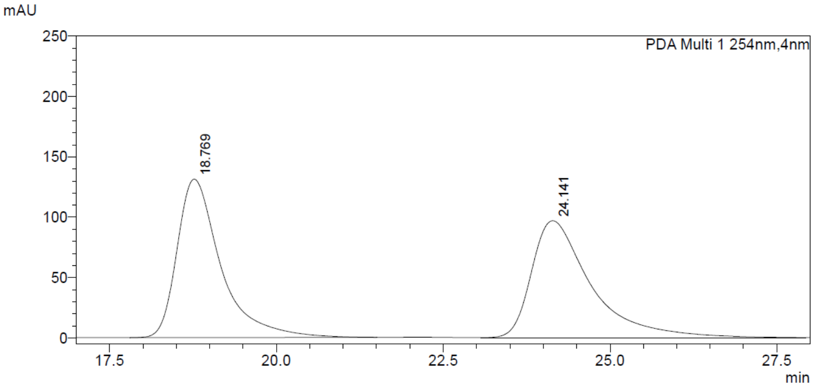

PDA Ch1 254nm

Peak#	Ret. Time	Area	Height	Aera%
1	18.769	6001837	131214	49.951
2	24.141	6013706	96850	50.049

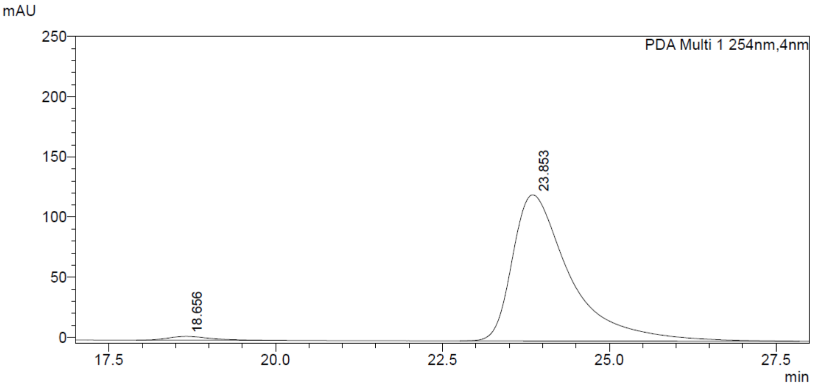

PDA Ch1 254nm

Peak#	Ret. Time	Area	Height	Aera%
1	18.656	137964	3208	1.855
2	23.853	7300576	121407	98.145

Supplementary Figure 57. HPLC Spectra of compound **4an** (Method B)

(*R*)-*N*-benzyl-2-(*p*-tolyl)-3-tosylpropanamide (4ao)

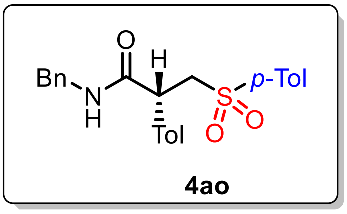

Method B: 45% yield, 94% ee.

^{1}H NMR (400 MHz, $CDCl_3$) δ 7.68 (d, *J* = 8.0 Hz, 2H), 7.30 – 7.16 (m, 6H), 7.12 – 7.04 (m, 6H), 6.07 (t, *J* = 4.8 Hz, 1H)., 4.35 – 4.22 (m, 3H), 4.09 – 4.06 (m, 1H), 3.37 (dd, *J* = 14.1, 5.1 Hz, 1H), 2.41 (s, 3H), 2.29 (s, 3H). **^{13}C NMR** (100 MHz, $CDCl_3$) δ 170.4, 144.6, 137.8, 137.7, 136.5, 134.4, 129.8, 129.7, 128.6, 128.0, 127.7, 127.5, 127.4, 58.9, 46.5, 43.9, 21.7, 21.1. **HRMS** (ESI) m/z Calcd for $[C_{24}H_{25}NNaO_3S, M+Na]^+$: 430.1453, found: 430.1448. $[\alpha]_D^{20}$ = -21 (c= 0.1, EtOAc).

HPLC analysis: OD-H column, *n*-hexane/*i*-PrOH = 90/10, flow rate = 1.0 mL·min^{-1}, λ = 232 nm, t_R = 20.6 min (major), 36.7 min (minor).

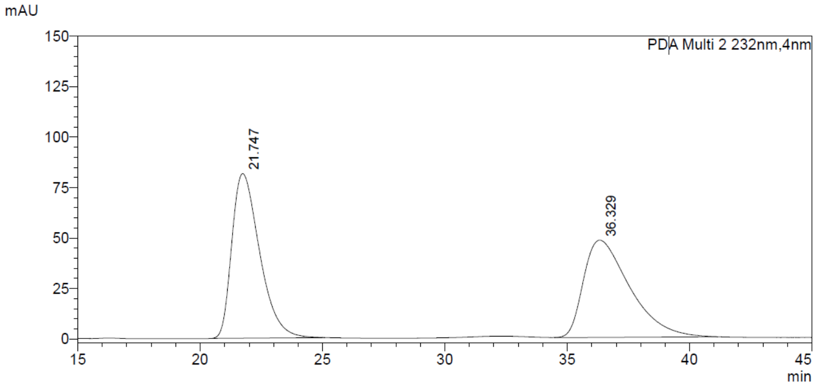

PDA Ch2 232nm

Peak#	Ret. Time	Area	Height	Aera%
1	21.747	6454099	81560	50.496
2	36.329	6327382	48121	49.504

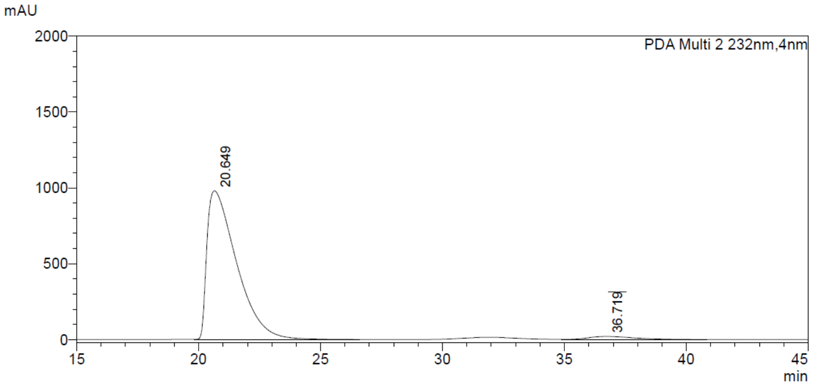

PDA Ch2 232nm

Peak#	Ret. Time	Area	Height	Aera%
1	20.649	83059337	980021	96.794
2	36.719	2751121	20793	3.206

Supplementary Figure 58. HPLC data of **4ao** (Method B)

(2*R*)-2-Methyl-3-((4-((5-methyl-2, 4-dioxo-5-(4-phenoxyphenyl)oxazolidin-3-yl)amino)phenyl) sulfonyl)-*N*-phenyl-2-(*p*-tolyl)propanamide (4ap)

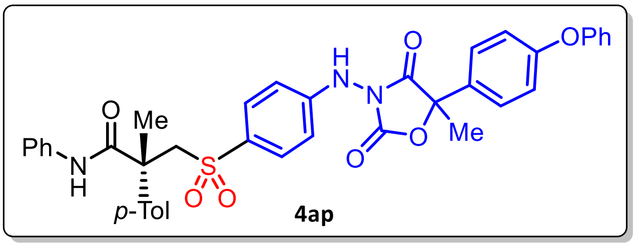

Method B: 66% yield, 98% de.

^{1}H NMR (400 MHz, $CDCl_3$) δ 7.50 (d, *J* = 8.7 Hz, 2H), 7.44 (d, *J* = 8.4 Hz, 2H), 7.39-7.35 (m, 2H), 7.29 (d, *J* = 7.8 Hz, 2H), 7.27 – 7.20 (m, 2H), 7.19 - 7.13 (m, 3H), 7.08 – 7.00 (m, 7H), 6.99 (s, 1H), 6.91 (s, 1H), 6.49 (d, *J* = 8.2 Hz, 2H), 4.07 (d, *J* = 14.8 Hz, 1H), 3.77 (d, *J* = 14.8 Hz, 1H), 2.29 (s, 1.5H), 2.27 (s, 1.5H), 2.05 (s, 3H), 1.94 (s, 3H). **^{13}C NMR** (100 MHz, $CDCl_3$) δ 173.1, 171.6, 158.7, 156.1, 152.3, 148.5, 138.2, 137.2, 136.2, 136.1, 133.9, 130.0, 129.9, 129.7, 129.5, 129.0, 126.9, 126.1, 124.8, 124.2, 120.3, 119.7, 118.6, 112.6, 85.3, 64.1, 50.0, 25.5, 22.8, 21.0, 21.0. **HRMS** (ESI) m/z Calcd for $[C_{39}H_{35}N_3NaO_7S, M+Na]^+$: 712.2088, found: 712.2106 (462.0543). $[\alpha]_D^{20}$ = 18 (c= 0.1, EtOAc).

HPLC analysis: AD-H column, *n*-hexane/*i*-PrOH = 70/30, flow rate = 1.0 mL·min^{-1}, λ = 254 nm, t_R = 33.8 min (minor), 56.7 min (major), 68.9 min (minor), 113.1 min (major).

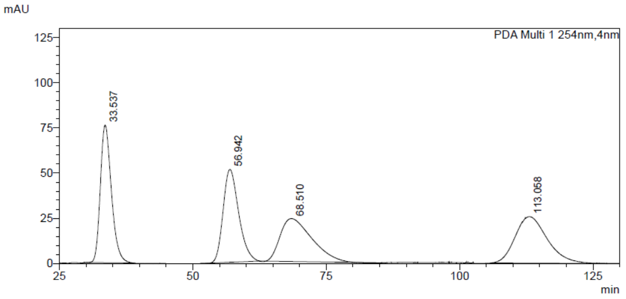

PDA Ch1 254nm

Peak#	Ret. Time	Area	Height	Aera%
1	33.537	10992071	76210	26.176
2	56.942	10469522	51466	24.932
3	68.510	9802429	23774	23.343
4	113.058	10728403	25701	25.548

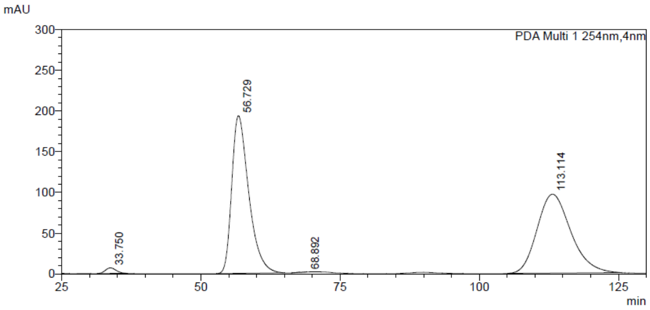

PDA Ch1 254nm

Peak#	Ret. Time	Area	Height	Aera%
1	33.750	939945	6956	1.137
2	56.729	41069746	193496	49.671
3	68.892	1900	15	0.002
4	113.114	40671938	96807	49.190

Supplementary Figure 59. HPLC Spectra of compound **4ap** (Method B)

(*R*)-3-(8*R*,9*S*,13*S*,14*S*)-3-Methoxy-13-methyl-17-oxo-7,8,9,11,12,13,14,15,16,17-decahydro-6*H*-cyclopenta[*a*]phenanthren-2-yl)sulfonyl)-2-methyl-*N*-phenyl-2-(*p*-tolyl)propanamide (4aq)

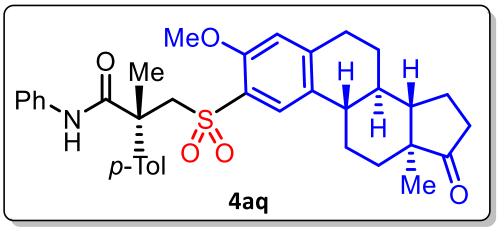

Method B: 70% yield, 94% de.

^{1}H NMR (400 MHz, $CDCl_3$) δ 7.45 (s, 1H), 7.30 (d, *J* = 7.8 Hz, 2H), 7.31 – 7.29 (m, 2H), 7.19 (d, *J* = 8.1 Hz, 2H), 7.09 – 7.02 (m, 2H), 6.99 (d, *J* = 8.0 Hz, 2H), 6.53 (s, 1H), 4.26 (d, *J* = 15.1 Hz, 1H), 4.19 (d, *J* = 15.1 Hz, 1H), 3.87 (s, 3H), 2.93 – 2.80 (m, 2H), 2.54 – 2.47 (m, 1H), 2.27 (s, 4H), 2.20 – 1.95 (m, 9H), 1.63 (d, *J* = 11.3 Hz, 1H), 1.54 – 1.46 (m, 2H), 1.43 (d, *J* = 9.7 Hz, 2H), 0.94 (s, 3H). **^{13}C NMR** (100 MHz, $CDCl_3$) δ 173.2, 154.5, 144.6, 137.5, 137.5, 136.6, 131.8, 129.3, 128.9, 127.0, 126.9, 125.6, 124.5, 119.9, 112.2, 61.6, 56.1, 50.2, 50.0, 47.9, 43.6, 38.1, 35.8, 31.4, 29.9, 26.1, 25.5, 23.2, 21.6, 21.2, 13.9. **HRMS** (ESI) m/z Calcd for $[C_{36}H_{41}NNaO_5S, M+Na]^+$: 622.2598, found: 622.2614. $[\alpha]_D^{20}$ = 64 (c= 0.1, EtOAc).

HPLC analysis: IA column, *n*-hexane/*i*-PrOH = 60/40, flow rate = 1.0 mL·min^{-1}, λ = 254 nm, t_R = 18.4 min (minor), 23.0 min (major).

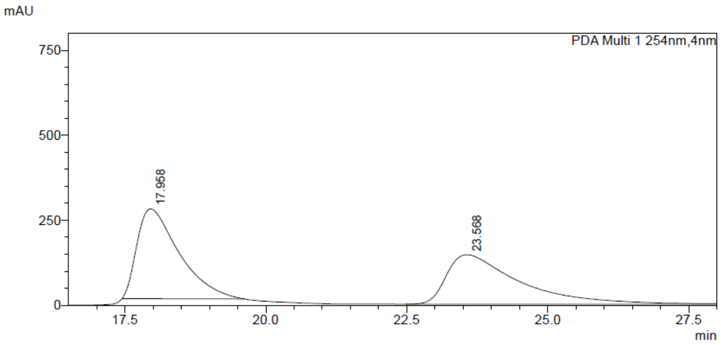

<

PDA Ch1 254nm

Peak#	Ret. Time	Area	Height	Aera%
1	17.958	13750700	263786	50.879
2	23.568	13275718	145377	49.121

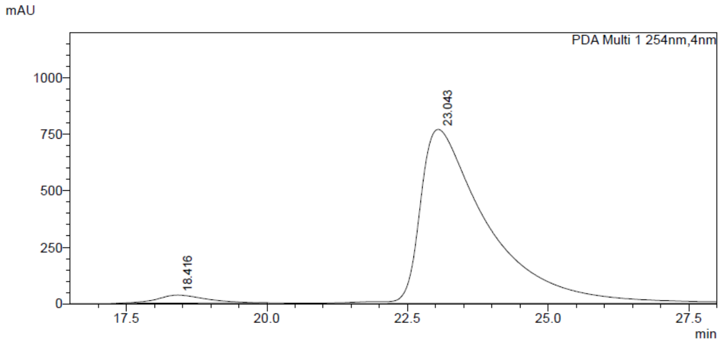

PDA Ch1 254nm

Peak#	Ret. Time	Area	Height	Aera%
1	18.416	2180902	35886	3.218
2	23.043	65600804	770682	96.782

Supplementary Figure 60. HPLC Spectra of compound **4aq** (Method B)

2.6 Crystal data and structure refinement for 4p

The crystal structure of compound **4p** has been deposited at the Cambridge Crystallographic Data Centre (**CCDC 2208406**).

The data is available free of charge at www.ccdc.cam.ac.uk/conts/retrieving.html.

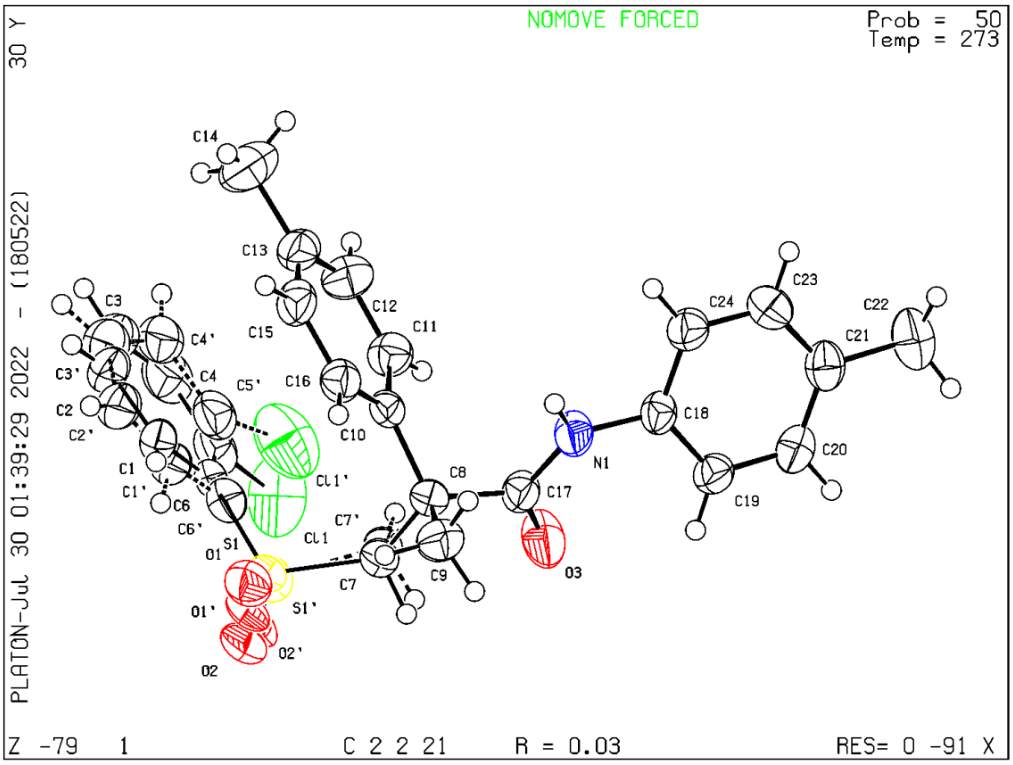

Supplementary Figure 61. Crystal data and structure refinement for **4p** (CCDC 2208406)

Supplementary Table 3. Crystal data and structure refinement for **4p**.

Identification code	4p
Empirical formula	$C_{24}H_{24}ClNO_3S$
Formula weight	441.95
Temperature/K	273.15
Crystal system	orthorhombic
Space group	$C222_1$
a/Å	13.418(2)
b/Å	17.769(2)
c/Å	19.232(3)
α/°	90
β/°	90
γ/°	90
Volume/Å^3	4585.4(11)
Z	8
ρ_{calc}g/cm^3	1.280

μ/mm^{-1}	0.282
F(000)	1856.0
Crystal size/mm^3	0.26 × 0.25 × 0.24
Radiation	MoKα (λ = 0.71073)
2Θ range for data collection/°	5.694 to 54.798
Index ranges	-15 ≤ h ≤ 16, -22 ≤ k ≤ 11, -24 ≤ l ≤ 24
Reflections collected	13754
Independent reflections	5103 [R_{int} = 0.0282, R_{sigma} = 0.0357]
Data/restraints/parameters	5103/520/372
Goodness-of-fit on F^2	0.906
Final R indexes [I>=2σ (I)]	R_1 = 0.0366, wR_2 = 0.0994
Final R indexes [all data]	R_1 = 0.0555, wR_2 = 0.1181
Largest diff. peak/hole / e Å^{-3}	0.20/-0.19
Flack parameter	0.01(2)

Supplementary Table 4. Fractional atomic coordinates (×10^4) and equivalent isotropic displacement parameters (Å^2×10^3) for **4p**. U_{eq} is defined as 1/3 of of the trace of the orthogonalised U_{IJ} tensor.

**Atom**	***x***	***y***	***z***	**U(eq)**
C7	5218(4)	7052(4)	5027(3)	45.8(13)
S1	4948.8(16)	7302.8(15)	4146.6(11)	43.6(5)
O1	5334(4)	8041(2)	3996(2)	55.8(10)
O2	3887(2)	7190(3)	4071(2)	62.9(12)
C1	6203(5)	6962(3)	3089(4)	50.7(13)
C2	6666(5)	6501(4)	2606(3)	64.6(15)
C3	6517(5)	5741(4)	2623(3)	72.5(16)
C4	5877(6)	5425(3)	3103(3)	70.4(15)
C5	5381(4)	5881(3)	3584(3)	57.2(13)
Cl1	4569(4)	5448.0(15)	4154.9(12)	96.8(10)
C6	5559(7)	6655(3)	3584(4)	43.7(9)
C7'	5260(30)	6890(30)	5032(19)	45.8(13)
S1'	4768(14)	7093(12)	4184(9)	60(3)
O1'	4950(20)	7908(15)	4048(16)	60(5)
O2'	3798(16)	6830(20)	4166(14)	62(5)
C1'	6040(40)	7070(20)	3120(30)	53(3)
C2'	6580(30)	6760(20)	2590(20)	59(3)
C3'	6720(30)	6020(20)	2540(20)	64(4)
C4'	6320(30)	5514(18)	3019(18)	62(3)
C5'	5690(30)	5815(15)	3539(19)	58(3)
Cl1'	5080(20)	5287(12)	4163(10)	106(4)
C6'	5520(50)	6600(16)	3570(30)	52(3)

O3	5508.4(18)	6584.3(19)	6310.4(13)	72.8(8)
N1	7154(2)	6839.2(18)	6387.3(14)	47.5(7)
C8	6262(2)	7220.4(19)	5328.7(15)	41.4(7)
C9	6372(3)	8074(2)	5461(2)	54.9(8)
C10	7086(2)	6894.7(17)	4865.1(14)	36.7(6)
C11	7218(2)	6115.4(19)	4825.3(18)	49.2(8)
C12	7898(3)	5807(2)	4374(2)	58.0(9)
C13	8475(2)	6243(2)	3936.3(18)	51.1(8)
C14	9188(3)	5895(3)	3417(3)	82.9(13)
C15	8359(2)	7013(2)	3980.9(16)	51.2(8)
C16	7677(2)	7337(2)	4441.6(16)	45.2(7)
C17	6269(2)	6841.4(19)	6054.7(15)	43.0(7)
C18	7366(2)	6495.5(17)	7039.4(14)	40.0(7)
C19	6661(2)	6450.4(17)	7569.3(16)	44.3(7)
C20	6911(3)	6099.5(19)	8191.3(16)	48.8(8)
C21	7849(3)	5797.8(19)	8307.0(16)	48.7(8)
C22	8103(3)	5392(3)	8978(2)	71.3(11)
C23	8542(3)	5870(2)	7780.0(18)	53.7(8)
C24	8319(2)	6209(2)	7152.4(17)	48.6(8)

Supplementary Table 5. Anisotropic displacement parameters (Å^2×10^3) for **4p**. The anisotropic displacement factor exponent takes the form: $-2\pi^2[h^2a^{*2}U_{11}+2hka^*b^*U_{12}+\ldots]$.

**Atom**	**U_{11}**	**U_{22}**	**U_{33}**	**U_{23}**	**U_{13}**	**U_{12}**
C7	33.8(16)	65(3)	38.7(15)	0.3(17)	-1.4(12)	7.2(18)
S1	34.9(7)	56.1(10)	40.0(6)	1.4(6)	-5.1(5)	9.9(6)
O1	62(3)	49.5(17)	55.5(19)	1.8(14)	-4.3(19)	10.8(15)
O2	34.2(16)	97(3)	58(2)	8(2)	-9.8(13)	14.3(17)
C1	50(3)	63(3)	39(2)	-3(2)	-4(2)	7(2)
C2	59(3)	90(4)	45(2)	-17(3)	-4.8(19)	16(3)
C3	71(3)	87(4)	60(3)	-33(3)	-15(2)	21(3)
C4	76(4)	62(3)	73(3)	-24(2)	-29(3)	10(2)
C5	60(3)	56(2)	56(2)	-3.0(18)	-23(2)	-1.3(19)
Cl1	127(2)	77.8(12)	85.9(10)	5.6(8)	-10.0(14)	-47.6(14)
C6	42.6(19)	51.8(19)	36.7(17)	-2.1(16)	-14.4(16)	7.0(17)
C7'	33.8(16)	65(3)	38.7(15)	0.3(17)	-1.4(12)	7.2(18)
S1'	54(5)	75(5)	50(4)	4(4)	-5(3)	18(4)
O1'	65(11)	64(8)	51(9)	9(6)	-5(9)	18(7)
O2'	51(7)	93(12)	42(9)	8(9)	-7(6)	10(7)
C1'	54(6)	63(6)	42(6)	-8(5)	-10(5)	11(5)
C2'	59(6)	69(6)	49(6)	-14(5)	-9(5)	9(6)

C3'	66(6)	68(6)	56(6)	-17(6)	-14(6)	9(6)
C4'	64(7)	62(6)	60(6)	-13(5)	-20(6)	8(6)
C5'	59(6)	60(5)	56(6)	-7(5)	-21(5)	5(5)
Cl1'	104(9)	108(8)	106(7)	19(6)	-26(7)	-37(8)
C6'	50(5)	61(5)	45(5)	-4(4)	-11(5)	8(4)
O3	40.2(13)	128(2)	50.6(14)	22.9(15)	-5.2(11)	-22.4(14)
N1	34.5(14)	71.8(19)	36.3(13)	7.0(12)	1.6(11)	-9.6(13)
C8	32.0(14)	54.0(18)	38.3(15)	-0.4(13)	-0.9(11)	5.0(13)
C9	57(2)	53.1(19)	55(2)	-3.2(16)	4.6(16)	10.2(16)
C10	29.6(13)	48.2(16)	32.1(13)	0.7(12)	-3.6(11)	0.8(12)
C11	46.7(18)	49.6(19)	51.2(18)	4.9(14)	4.9(15)	-0.4(15)
C12	57(2)	52(2)	64(2)	-8.7(17)	5.5(18)	4.6(17)
C13	39.2(17)	68(2)	45.6(17)	-11.7(15)	-0.7(13)	0.3(16)
C14	68(3)	102(3)	79(3)	-31(3)	18(2)	2(2)
C15	42.5(17)	72(2)	39.3(17)	4.4(14)	4.1(13)	-6.8(15)
C16	42.8(16)	51.9(18)	40.8(15)	2.9(14)	0.0(13)	-2.6(14)
C17	37.2(15)	57.7(19)	34.2(15)	-0.9(13)	1.3(11)	-3.7(14)
C18	39.4(16)	48.0(18)	32.5(14)	-3.1(12)	-2.8(12)	-7.2(12)
C19	37.6(15)	55.1(18)	40.2(16)	-4.5(14)	1.8(13)	-1.1(13)
C20	54(2)	56(2)	36.5(15)	-2.1(13)	4.6(14)	-6.8(15)
C21	60(2)	45.6(17)	40.4(16)	-1.9(13)	-7.0(15)	-7.5(15)
C22	90(3)	72(3)	52(2)	11.9(18)	-15(2)	-4(2)
C23	43.6(19)	62(2)	55(2)	0.4(16)	-7.2(15)	2.6(16)
C24	39.1(17)	64(2)	42.6(17)	-2.5(15)	2.5(13)	-2.4(15)

Supplementary Table 6. Bond lengths for **4p.**

Atom	Atom	Length/Å	Atom	Atom	Length/Å
C7	S1	1.788(4)	C5'	Cl1'	1.72(2)
C7	C8	1.546(5)	C5'	C6'	1.42(2)
S1	O1	1.439(4)	O3	C17	1.221(4)
S1	O2	1.446(3)	N1	C17	1.349(4)
S1	C6	1.779(4)	N1	C18	1.424(4)
C1	C2	1.386(7)	C8	C9	1.544(5)
C1	C6	1.397(6)	C8	C10	1.534(4)
C2	C3	1.364(8)	C8	C17	1.550(4)
C3	C4	1.381(9)	C10	C11	1.398(5)
C4	C5	1.398(7)	C10	C16	1.382(4)
C5	Cl1	1.727(5)	C11	C12	1.373(5)
C5	C6	1.396(6)	C12	C13	1.382(5)
C7'	S1'	1.80(2)	C13	C14	1.515(5)

C7'	C8	1.57(2)	C13	C15	1.379(5)
S1'	O1'	1.49(2)	C15	C16	1.397(5)
S1'	O2'	1.386(19)	C18	C19	1.393(4)
S1'	C6'	1.78(2)	C18	C24	1.393(4)
C1'	C2'	1.37(2)	C19	C20	1.390(5)
C1'	C6'	1.39(2)	C20	C21	1.386(5)
C2'	C3'	1.34(2)	C21	C22	1.517(5)
C3'	C4'	1.39(3)	C21	C23	1.382(5)
C4'	C5'	1.42(2)	C23	C24	1.382(5)

Supplementary Table 7. Bond angles for **4p.**

**Atom**	**Atom**	**Atom**	**Angle/°**	**Atom**	**Atom**	**Atom**	**Angle/°**
C8	C7	S1	119.4(4)	C17	N1	C18	126.5(3)
O1	S1	C7	110.2(2)	C7	C8	C9	109.8(3)
O1	S1	O2	117.4(2)	C7	C8	C17	105.0(3)
O1	S1	C6	107.6(2)	C9	C8	C7'	120(2)
O2	S1	C7	105.1(2)	C9	C8	C17	106.1(3)
O2	S1	C6	107.6(3)	C10	C8	C7	111.2(3)
C6	S1	C7	108.8(3)	C10	C8	C7'	105(2)
C2	C1	C6	120.2(5)	C10	C8	C9	113.4(3)
C3	C2	C1	120.2(5)	C10	C8	C17	110.8(2)
C2	C3	C4	120.7(5)	C17	C8	C7'	99.9(12)
C3	C4	C5	120.1(5)	C11	C10	C8	119.8(3)
C4	C5	Cl1	117.5(4)	C16	C10	C8	122.8(3)
C4	C5	C6	119.3(5)	C16	C10	C11	117.3(3)
C6	C5	Cl1	123.1(4)	C12	C11	C10	121.0(3)
C1	C6	S1	116.5(4)	C11	C12	C13	122.2(4)
C1	C6	C5	119.4(4)	C12	C13	C14	121.7(4)
C5	C6	S1	124.0(4)	C15	C13	C12	117.1(3)
C8	C7'	S1'	125(2)	C15	C13	C14	121.2(4)
O1'	S1'	C7'	106.9(19)	C13	C15	C16	121.4(3)
O1'	S1'	C6'	105.5(16)	C10	C16	C15	121.0(3)
O2'	S1'	C7'	107.6(16)	O3	C17	N1	122.9(3)
O2'	S1'	O1'	118.9(17)	O3	C17	C8	121.3(3)
O2'	S1'	C6'	110(2)	N1	C17	C8	115.7(3)
C6'	S1'	C7'	107(2)	C19	C18	N1	122.2(3)
C2'	C1'	C6'	120(3)	C19	C18	C24	119.2(3)
C3'	C2'	C1'	121(3)	C24	C18	N1	118.5(3)
C2'	C3'	C4'	123(3)	C20	C19	C18	119.4(3)
C3'	C4'	C5'	117(2)	C21	C20	C19	122.1(3)

C4'	C5'	Cl1'	125(2)	C20	C21	C22	121.7(3)
C4'	C5'	C6'	120(2)	C23	C21	C20	117.2(3)
C6'	C5'	Cl1'	116(2)	C23	C21	C22	121.1(3)
C1'	C6'	S1'	114(2)	C24	C23	C21	122.4(3)
C1'	C6'	C5'	119(2)	C23	C24	C18	119.6(3)
C5'	C6'	S1'	127(2)				

Supplementary Table 8. Torsion angles for **4p.**

A	B	C	D	Angle/°	A	B	C	D	Angle/°
C7	S1	C6	C1	-124.0(8)	C4'	C5'	C6'	S1'	-179(5)
C7	S1	C6	C5	60.1(9)	C4'	C5'	C6'	C1'	-5(10)
C7	C8	C10	C11	-69.9(4)	Cl1'	C5'	C6'	S1'	1(9)
C7	C8	C10	C16	105.8(4)	Cl1'	C5'	C6'	C1'	175(6)
C7	C8	C17	O3	-9.5(5)	C6'	C1'	C2'	C3'	-8(9)
C7	C8	C17	N1	172.6(4)	N1	C18	C19	C20	179.2(3)
S1	C7	C8	C9	73.3(5)	N1	C18	C24	C23	-179.7(3)
S1	C7	C8	C10	-53.1(5)	C8	C7	S1	O1	-43.2(5)
S1	C7	C8	C17	-173.0(4)	C8	C7	S1	O2	-170.6(4)
O1	S1	C6	C1	-4.7(9)	C8	C7	S1	C6	74.5(6)
O1	S1	C6	C5	179.4(7)	C8	C7'	S1'	O1'	-40(5)
O2	S1	C6	C1	122.7(8)	C8	C7'	S1'	O2'	-168(4)
O2	S1	C6	C5	-53.2(9)	C8	C7'	S1'	C6'	73(5)
C1	C2	C3	C4	2.4(9)	C8	C10	C11	C12	174.9(3)
C2	C1	C6	S1	-176.5(6)	C8	C10	C16	C15	-174.2(3)
C2	C1	C6	C5	-0.4(13)	C9	C8	C10	C11	165.8(3)
C2	C3	C4	C5	-0.7(8)	C9	C8	C10	C16	-18.5(4)
C3	C4	C5	Cl1	178.4(4)	C9	C8	C17	O3	106.7(4)
C3	C4	C5	C6	-1.6(9)	C9	C8	C17	N1	-71.2(4)
C4	C5	C6	S1	177.8(6)	C10	C8	C17	O3	-129.7(3)
C4	C5	C6	C1	2.1(12)	C10	C8	C17	N1	52.4(4)
Cl1	C5	C6	S1	-2.2(11)	C10	C11	C12	C13	-0.6(5)
Cl1	C5	C6	C1	-177.9(7)	C11	C10	C16	C15	1.6(4)
C6	C1	C2	C3	-1.9(11)	C11	C12	C13	C14	-177.1(4)
C7'	S1'	C6'	C1'	-115(6)	C11	C12	C13	C15	1.6(5)
C7'	S1'	C6'	C5'	58(7)	C12	C13	C15	C16	-0.9(5)
C7'	C8	C10	C11	-60.6(15)	C13	C15	C16	C10	-0.7(5)
C7'	C8	C10	C16	115.1(14)	C14	C13	C15	C16	177.8(3)
C7'	C8	C17	O3	-19(2)	C16	C10	C11	C12	-1.0(5)
C7'	C8	C17	N1	163(2)	C17	N1	C18	C19	-33.3(5)
S1'	C7'	C8	C9	65(5)	C17	N1	C18	C24	148.0(3)

S1'	C7'	C8	C10	-65(4)	C17	C8	C10	C11	46.5(4)
S1'	C7'	C8	C17	-180(4)	C17	C8	C10	C16	-137.8(3)
O1'	S1'	C6'	C1'	-2(7)	C18	N1	C17	O3	5.5(6)
O1'	S1'	C6'	C5'	172(6)	C18	N1	C17	C8	-176.6(3)
O2'	S1'	C6'	C1'	128(6)	C18	C19	C20	C21	0.8(5)
O2'	S1'	C6'	C5'	-59(7)	C19	C18	C24	C23	1.6(5)
C1'	C2'	C3'	C4'	0(8)	C19	C20	C21	C22	-177.8(3)
C2'	C1'	C6'	S1'	-175(5)	C19	C20	C21	C23	1.1(5)
C2'	C1'	C6'	C5'	11(11)	C20	C21	C23	C24	-1.6(5)
C2'	C3'	C4'	C5'	4(7)	C21	C23	C24	C18	0.3(5)
C3'	C4'	C5'	Cl1'	178(3)	C22	C21	C23	C24	177.3(4)
C3'	C4'	C5'	C6'	-2(7)	C24	C18	C19	C20	-2.1(5)

Supplementary Table 9. Hydrogen atom coordinates (Å×10^4) and isotropic displacement parameters (Å^2×10^3) for **4p.**

**Atom**	***x***	***y***	***z***	**U(eq)**
H7A	4733	7303	5320	55
H7B	5103	6515	5074	55
H1	6322	7478	3084	61
H2	7079	6710	2269	78
H3	6850	5434	2308	87
H4	5776	4907	3107	84
H7'A	4749	7031	5361	55
H7'B	5327	6349	5058	55
H1'	6017	7587	3179	64
H2'	6864	7079	2253	71
H3'	7101	5827	2176	76
H4'	6461	5002	2999	75
H1A	7610(30)	6970(20)	6190(20)	57
H9A	7037	8180	5616	82
H9B	5904	8228	5810	82
H9C	6243	8343	5037	82
H11	6840	5802	5108	59
H12	7973	5287	4363	70
H14A	9588	5522	3646	124
H14B	9611	6279	3228	124
H14C	8816	5663	3049	124
H15	8743	7324	3698	61
H16	7621	7858	4464	54
H19	6028	6653	7507	53

H20	6433	6066	8541	59
H22A	7710	5595	9351	107
H22B	8797	5459	9080	107
H22C	7961	4865	8927	107
H23	9181	5683	7850	64
H24	8803	6247	6808	58

Supplementary Table 10. Atomic occupancy for **4p.**

**Atom**	***Occupancy***	**Atom**	***Occupancy***	**Atom**	***Occupancy***
C7	0.866(11)	H7A	0.866(11)	H7B	0.866(11)
S1	0.866(11)	O1	0.866(11)	O2	0.866(11)
C1	0.866(11)	H1	0.866(11)	C2	0.866(11)
H2	0.866(11)	C3	0.866(11)	H3	0.866(11)
C4	0.866(11)	H4	0.866(11)	C5	0.866(11)
Cl1	0.866(11)	C6	0.866(11)	C7'	0.134(11)
H7'A	0.134(11)	H7'B	0.134(11)	S1'	0.134(11)
O1'	0.134(11)	O2'	0.134(11)	C1'	0.134(11)
H1'	0.134(11)	C2'	0.134(11)	H2'	0.134(11)
C3'	0.134(11)	H3'	0.134(11)	C4'	0.134(11)
H4'	0.134(11)	C5'	0.134(11)	Cl1'	0.134(11)
C6'	0.134(11)				

3 Supplementary Figures

3.1 NMR Spectra of substrates 1

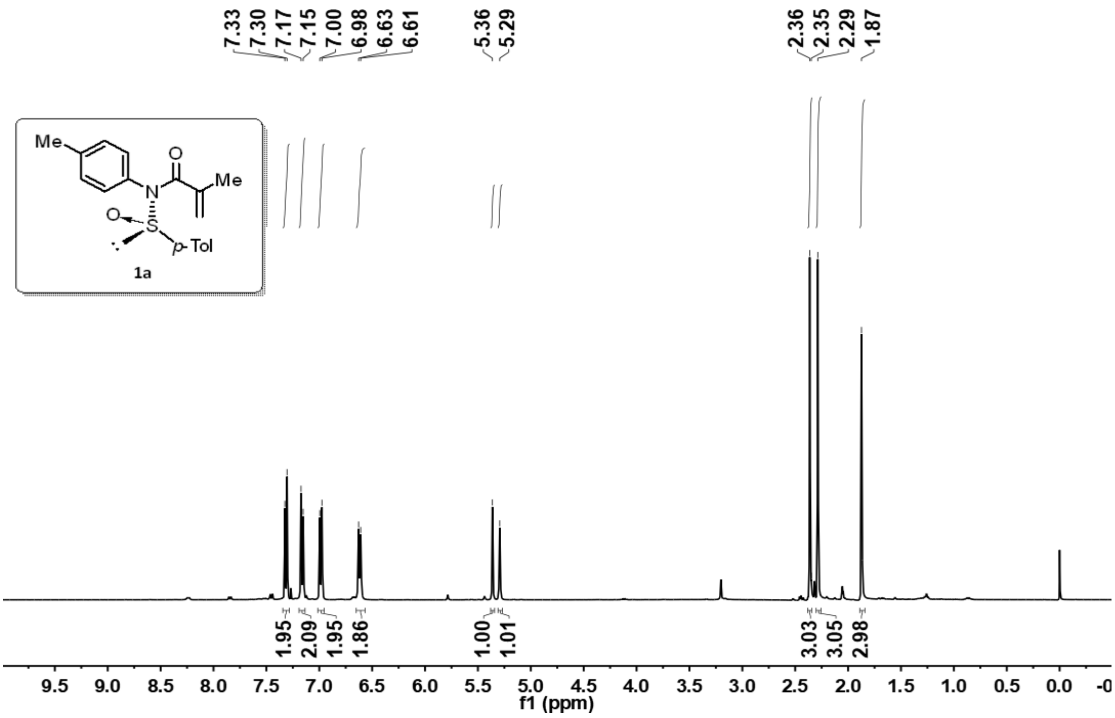

Supplementary Figure 62. ^{1}H NMR-spectrum of **1a**, recorded at 400 MHz and 25 °C in $CDCl_3$.

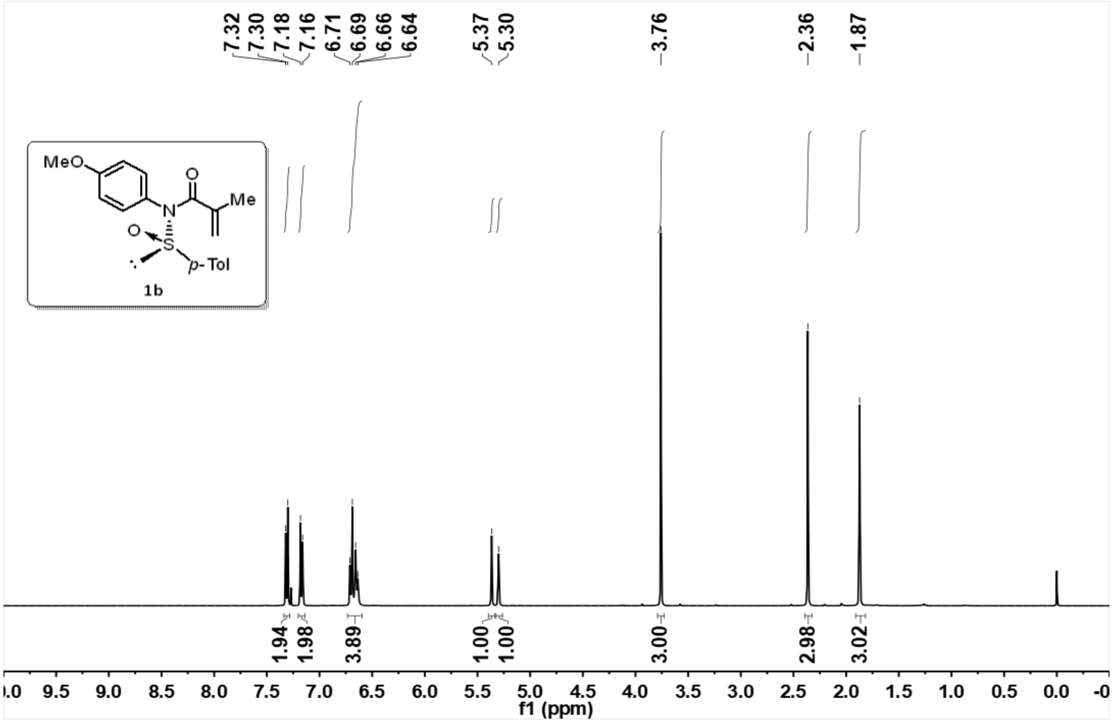

Supplementary Figure 63. ^{1}H NMR-spectrum of **1b**, recorded at 400 MHz and 25 °C in $CDCl_3$

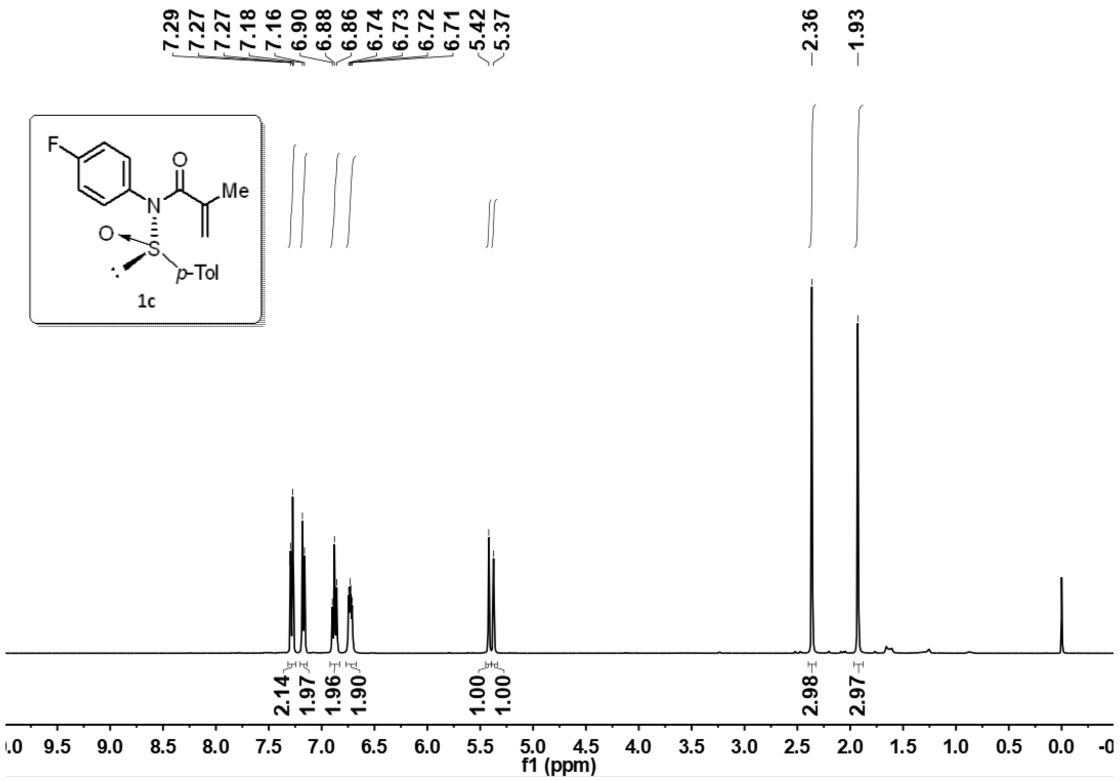

Supplementary Figure 64. ^{1}H NMR-spectrum of **1c**, recorded at 400 MHz and 25 °C in $CDCl_3$

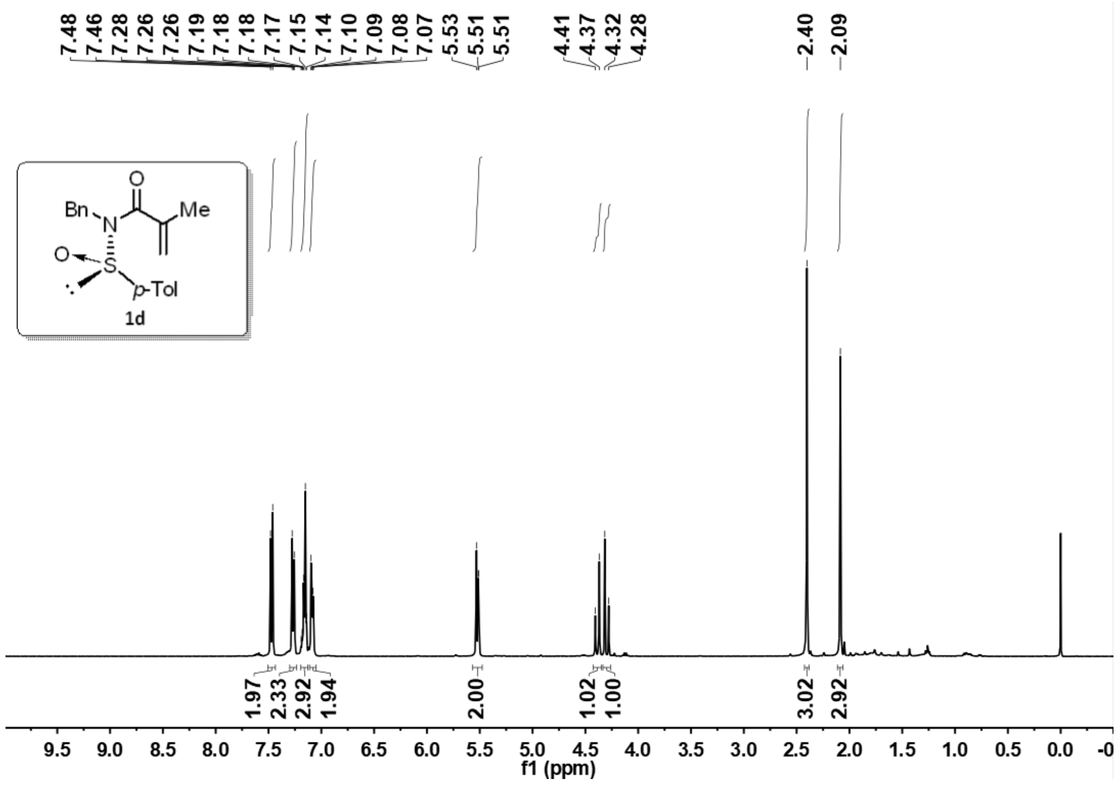

Supplementary Figure 65. ^{1}H NMR-spectrum of **1d**, recorded at 400 MHz and 25 °C in $CDCl_3$

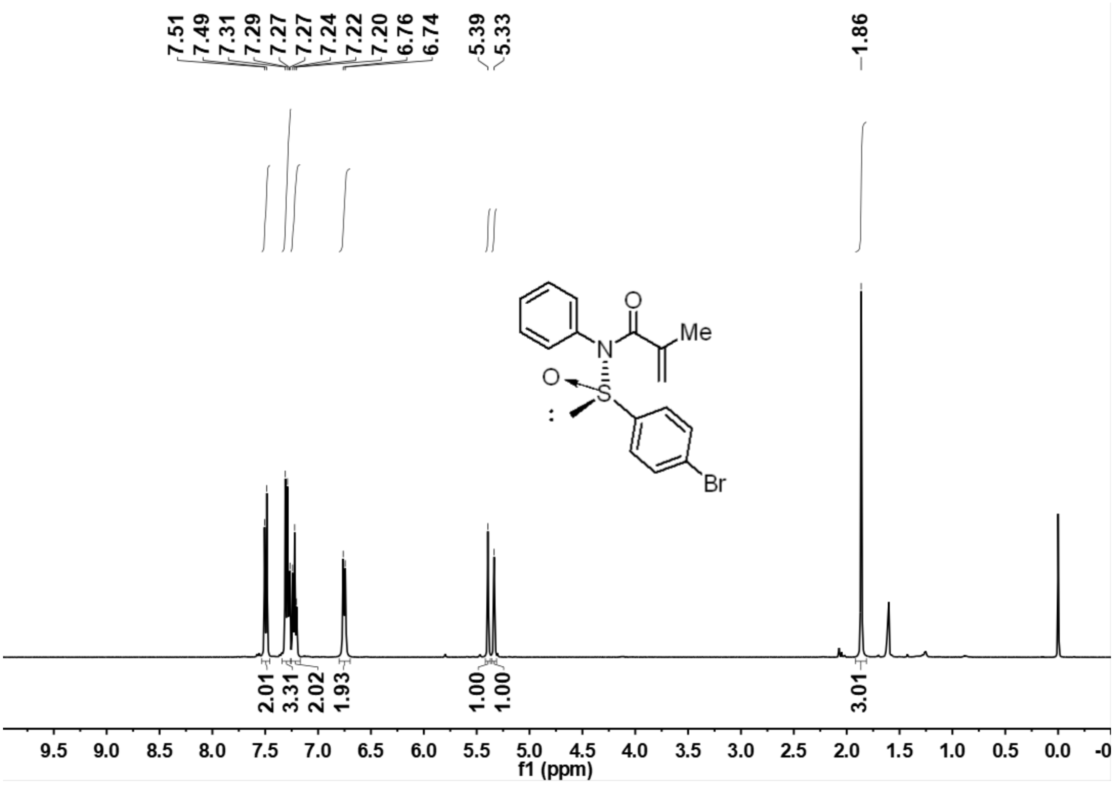

Supplementary Figure 66. ^{1}H NMR-spectrum of **1e**, recorded at 400 MHz and 25 °C in $CDCl_3$

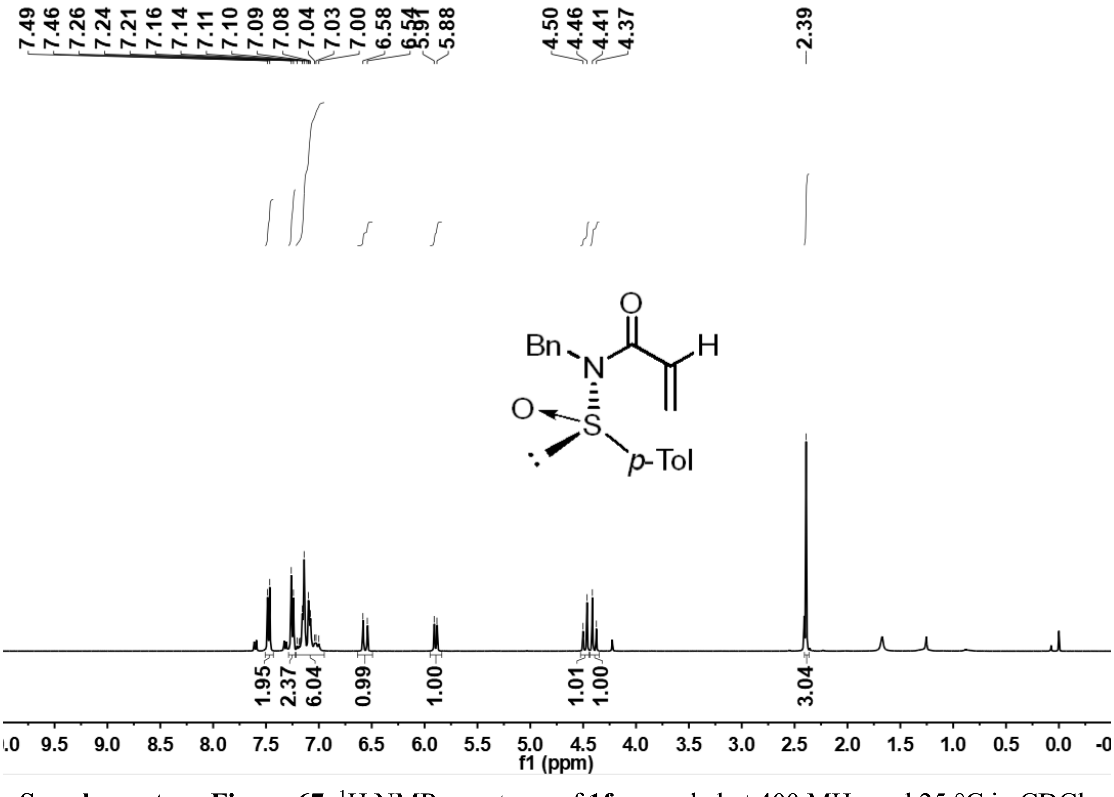

Supplementary Figure 67. ^{1}H NMR-spectrum of **1f**, recorded at 400 MHz and 25 °C in $CDCl_3$

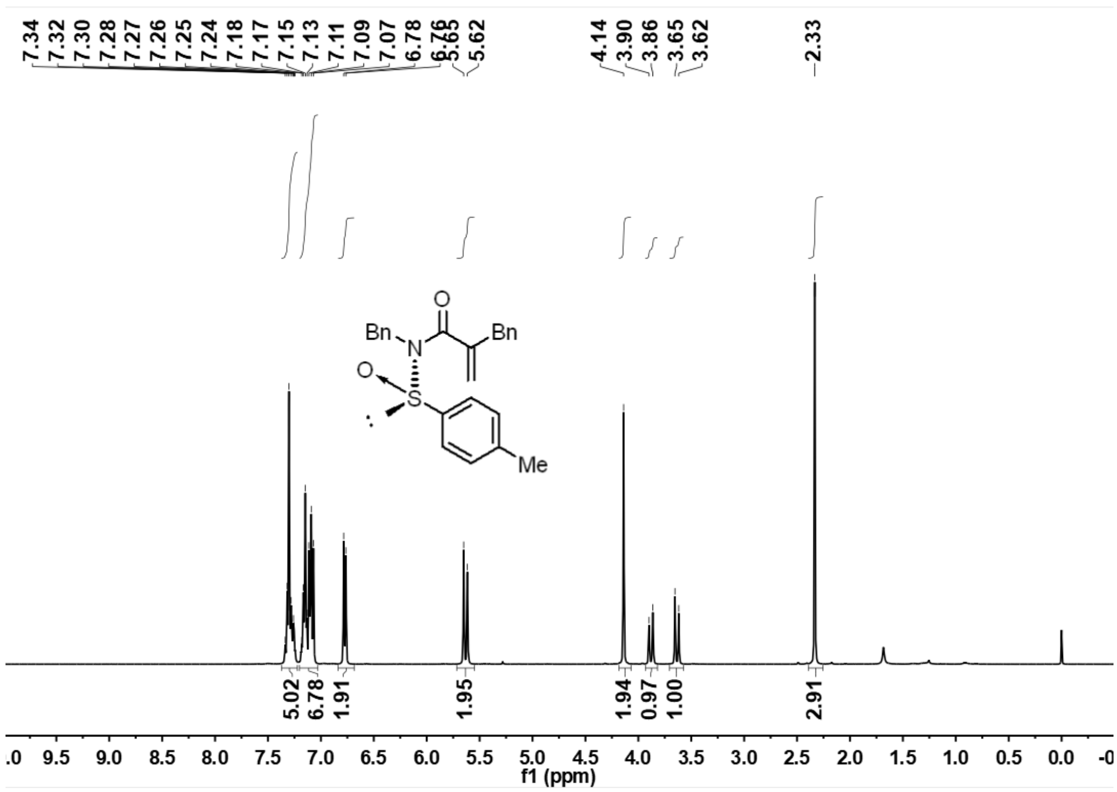

Supplementary Figure 68. ^{1}H NMR-spectrum of **1g**, recorded at 400 MHz and 25 °C in $CDCl_3$

3.2 NMR Spectra of products 4

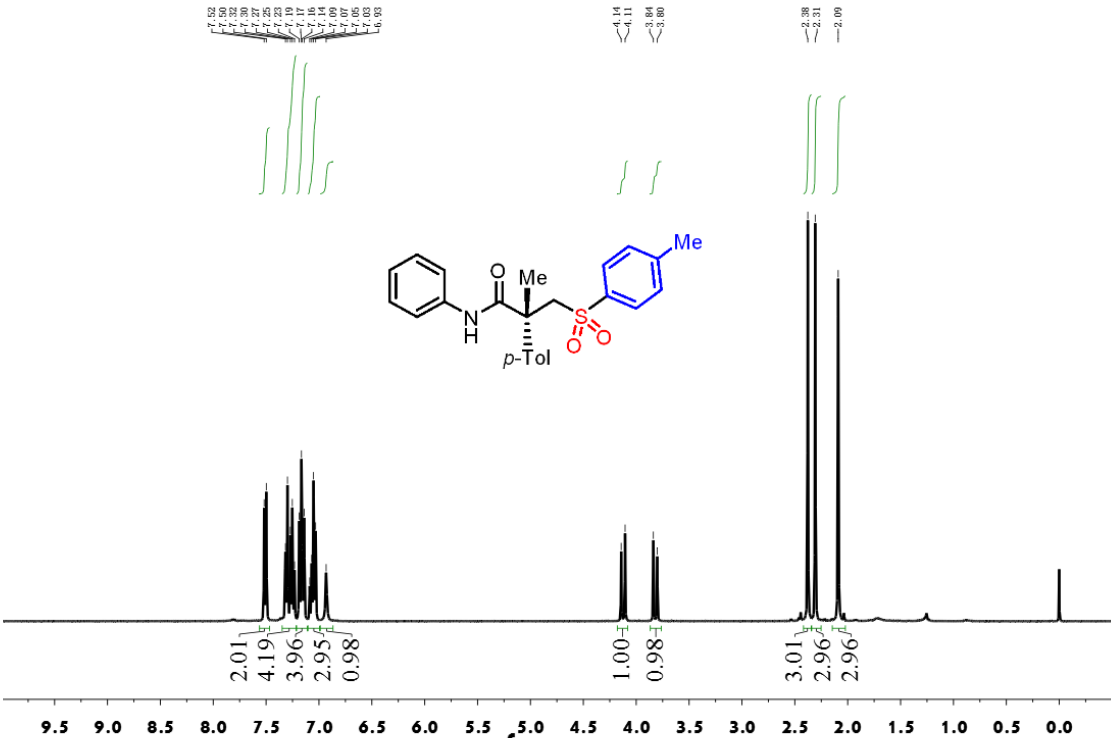

Supplementary Figure 69. ^{1}H NMR-spectrum of **4a**, recorded at 400 MHz and 25 °C in $CDCl_3$

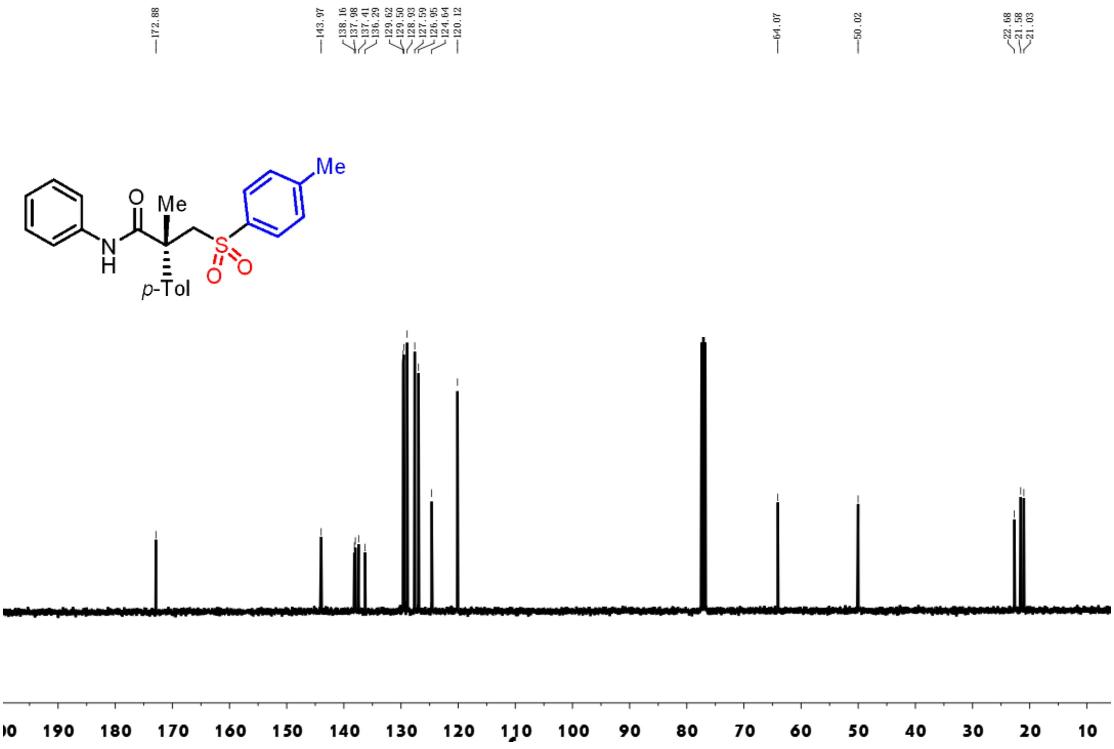

Supplementary Figure 70. ^{13}C NMR-spectrum of **4a**, recorded at 400 MHz and 25 °C in $CDCl_3$

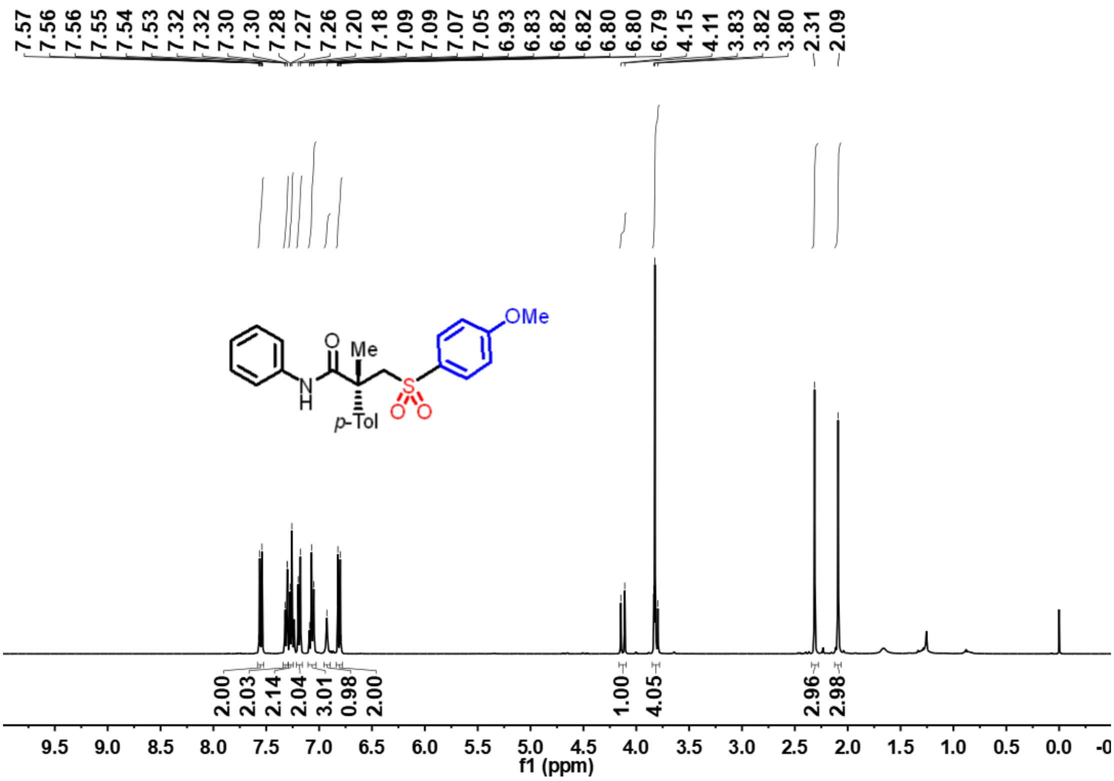

Supplementary Figure 71. ^{1}H NMR-spectrum of **4b**, recorded at 400 MHz and 25 °C in $CDCl_3$

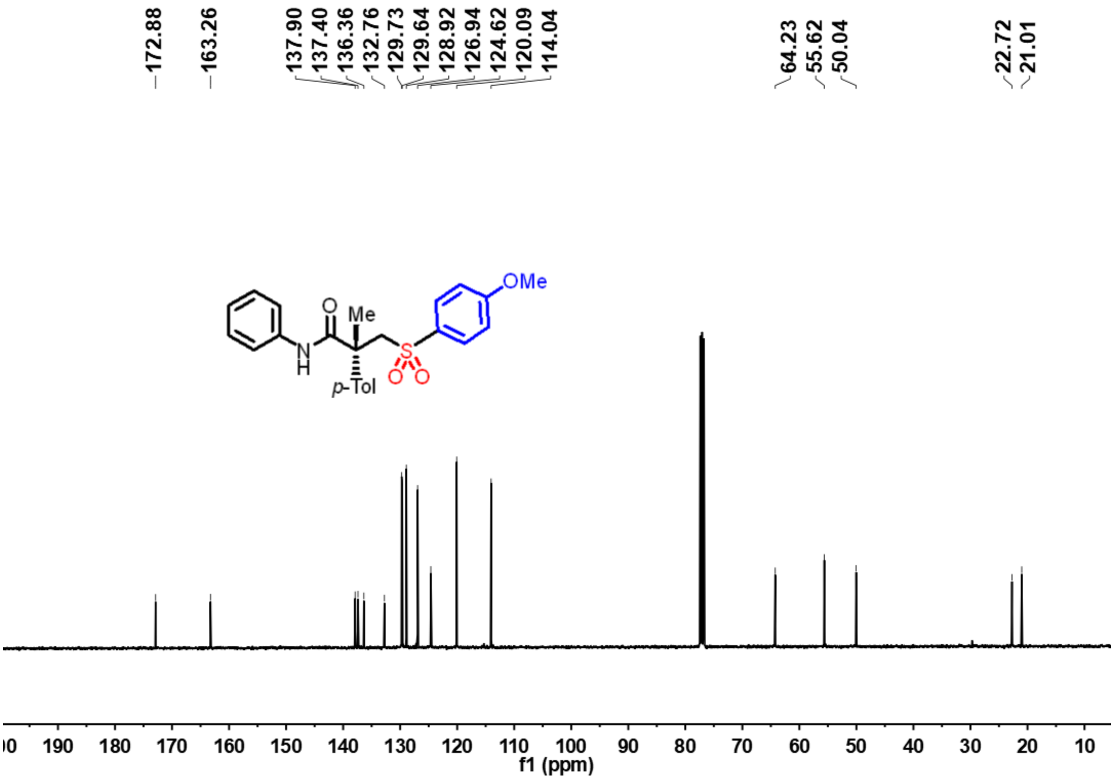

Supplementary Figure 72. ^{13}C NMR-spectrum of **4b**, recorded at 400 MHz and 25 °C in $CDCl_3$

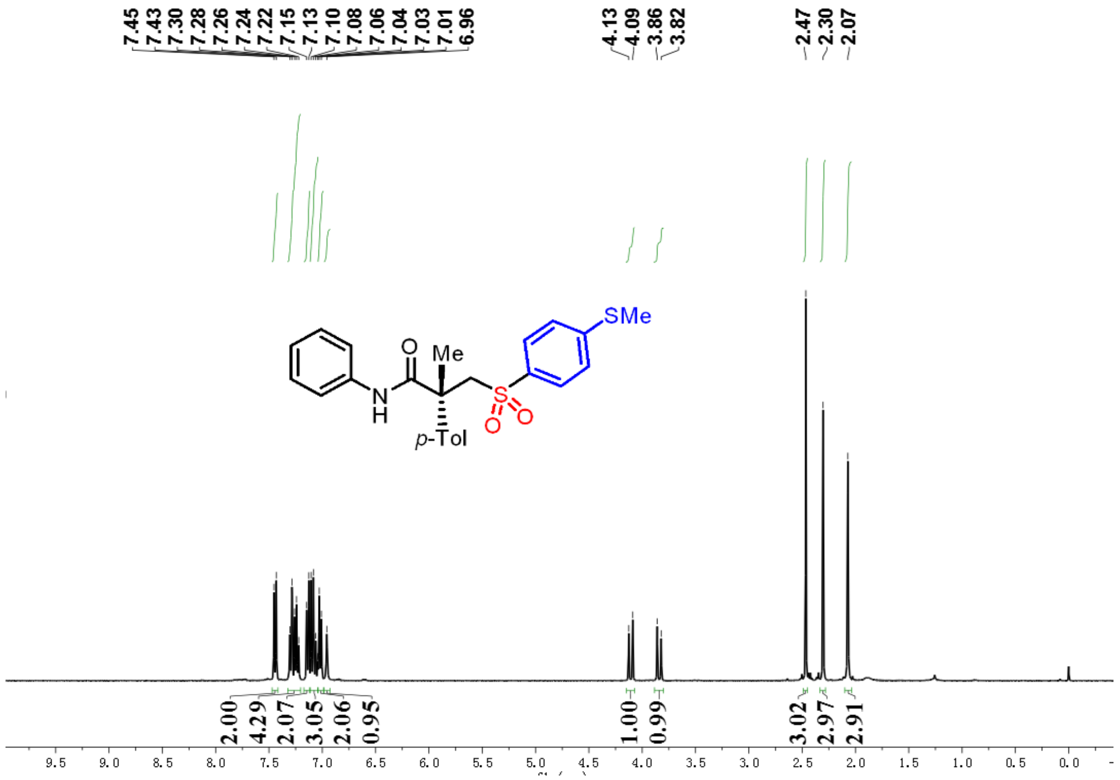

Supplementary Figure 73. ^{1}H NMR-spectrum of **4c**, recorded at 400 MHz and 25 °C in $CDCl_3$

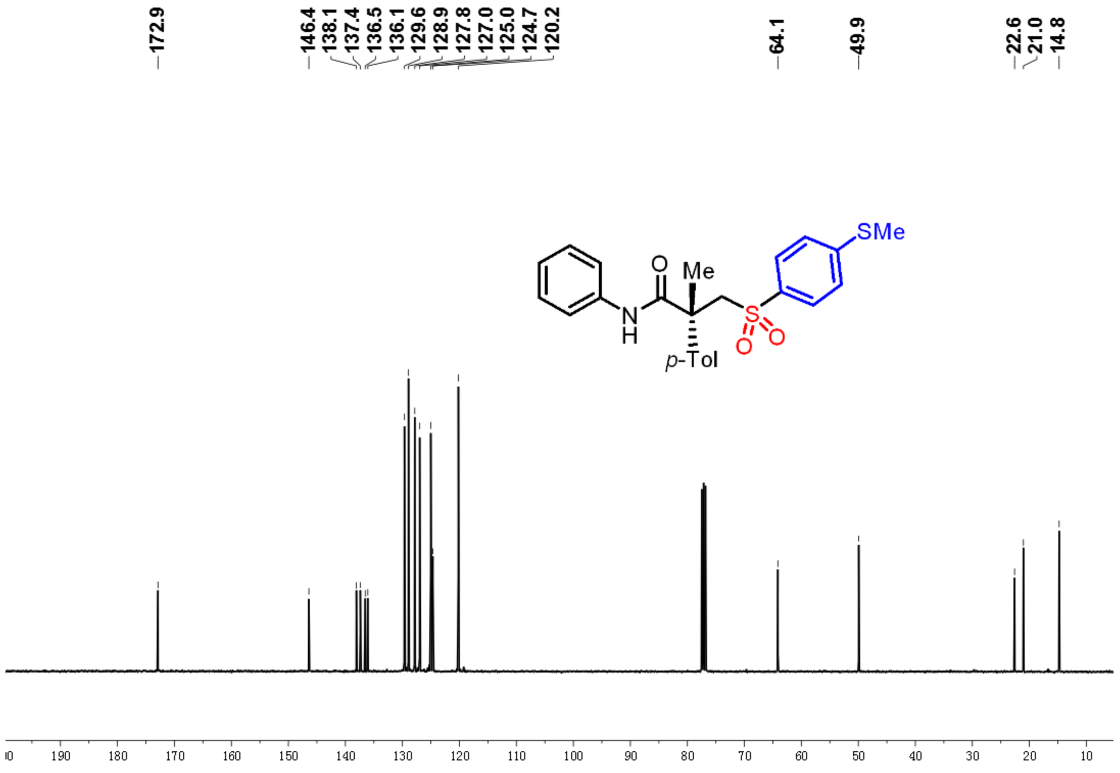

Supplementary Figure 74. ^{13}C NMR-spectrum of **4c**, recorded at 400 MHz and 25 °C in $CDCl_3$

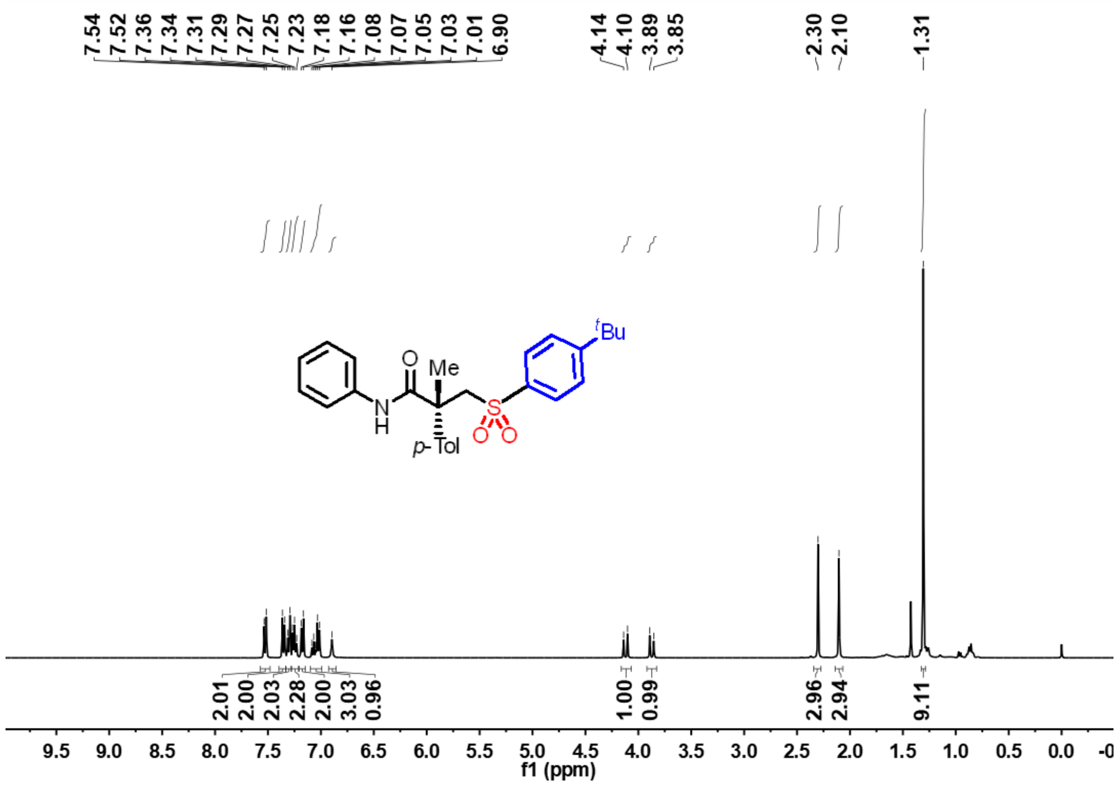

Supplementary Figure 75. ^{1}H NMR-spectrum of **4d**, recorded at 400 MHz and 25 °C in $CDCl_3$

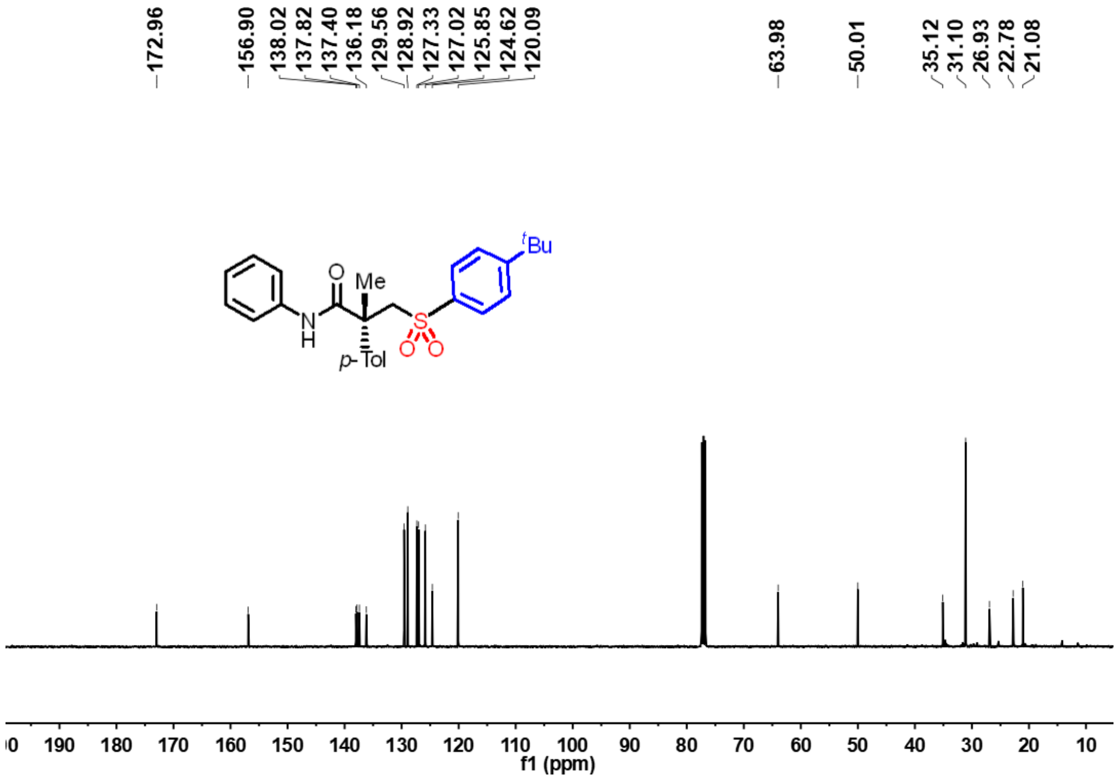

Supplementary Figure 76. ^{13}C NMR-spectrum of **4d**, recorded at 400 MHz and 25 °C in $CDCl_3$

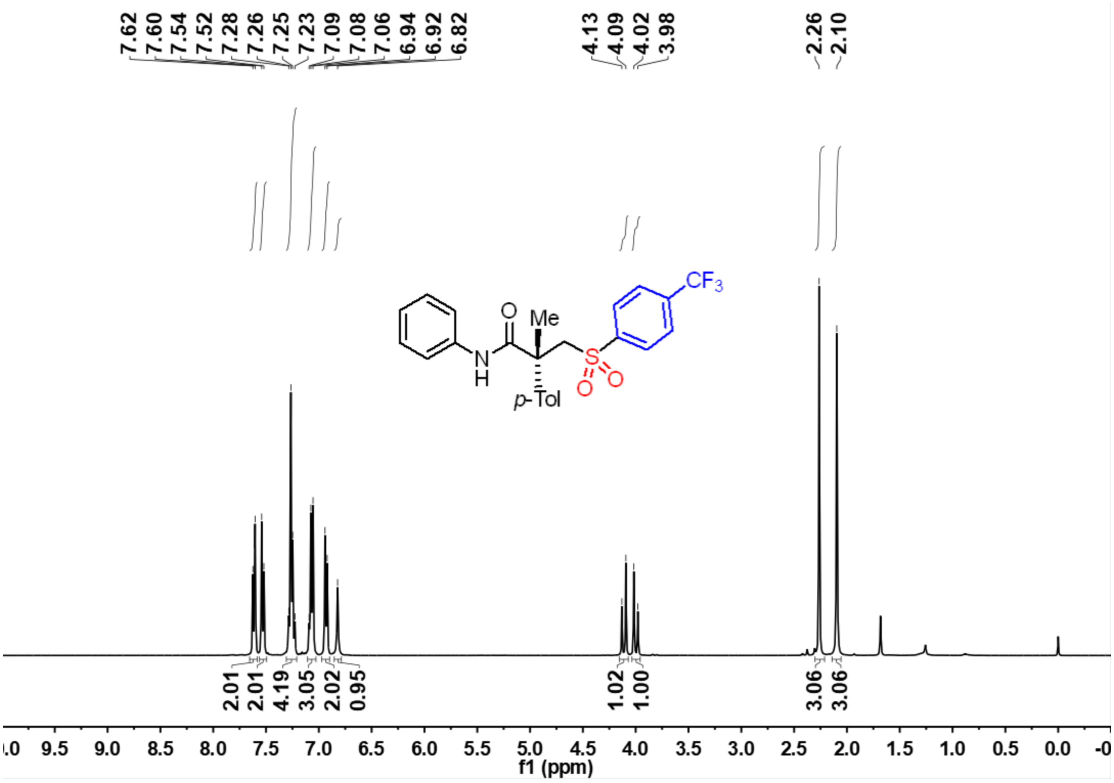

Supplementary Figure 77. ^{1}H NMR-spectrum of **4e**, recorded at 400 MHz and 25 °C in $CDCl_3$

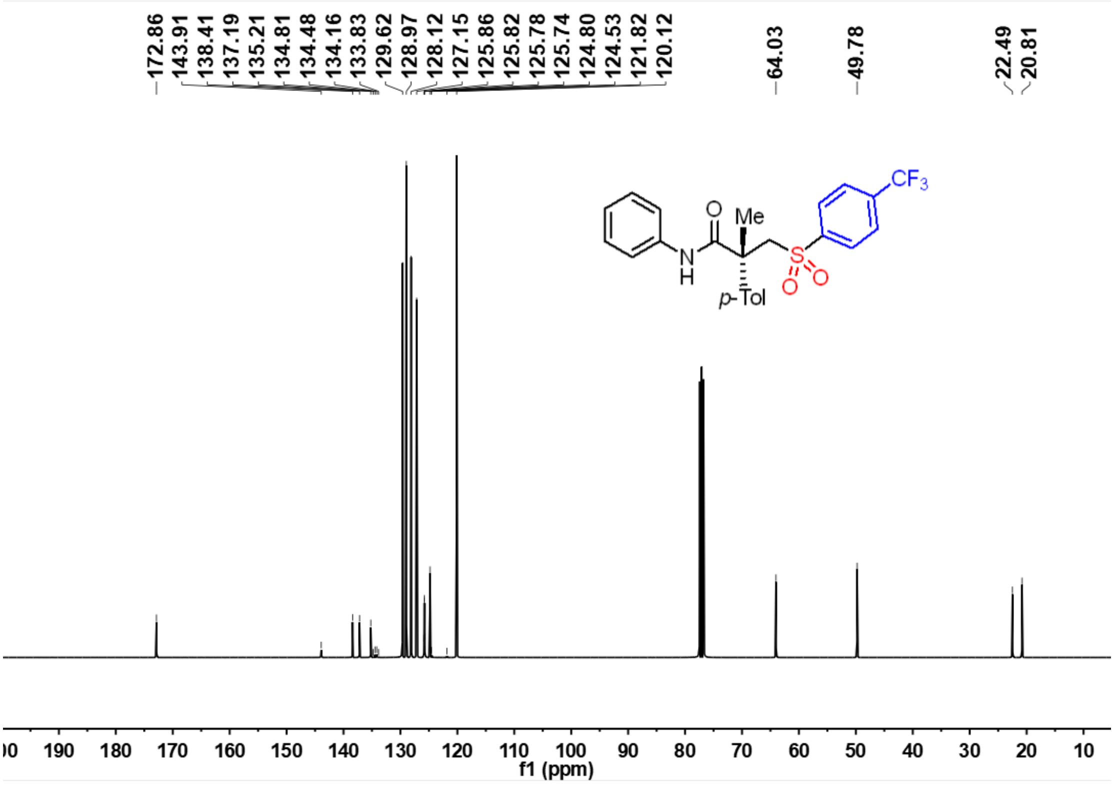

Supplementary Figure 78. ^{13}C NMR-spectrum of **4e**, recorded at 400 MHz and 25 °C in $CDCl_3$

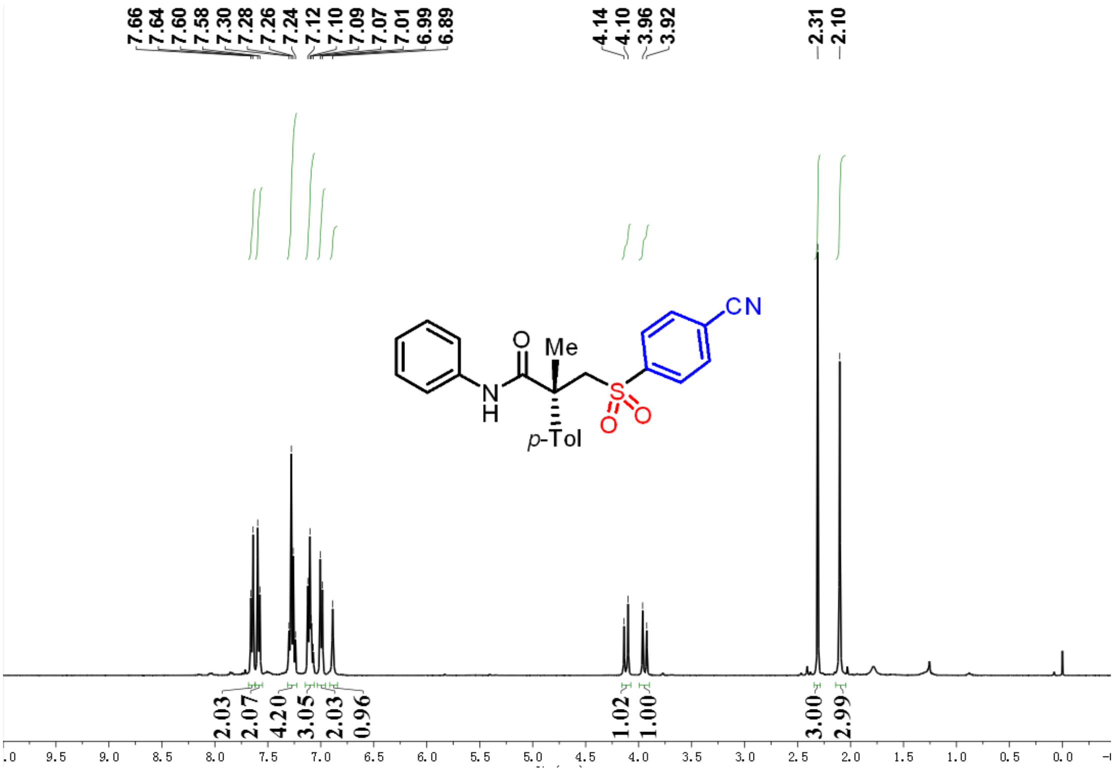

Supplementary Figure 79. ^{1}H NMR-spectrum of **4f**, recorded at 400 MHz and 25 °C in $CDCl_3$

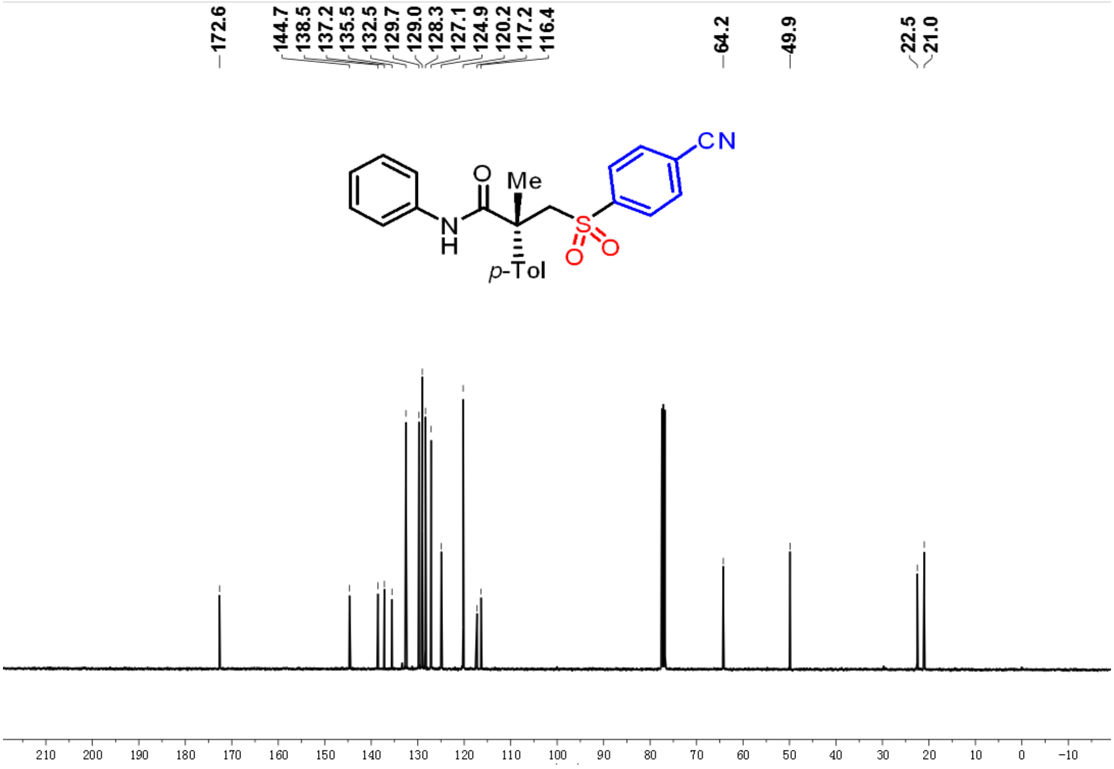

Supplementary Figure 80. ^{13}C NMR-spectrum of **4f**, recorded at 400 MHz and 25 °C in $CDCl_3$

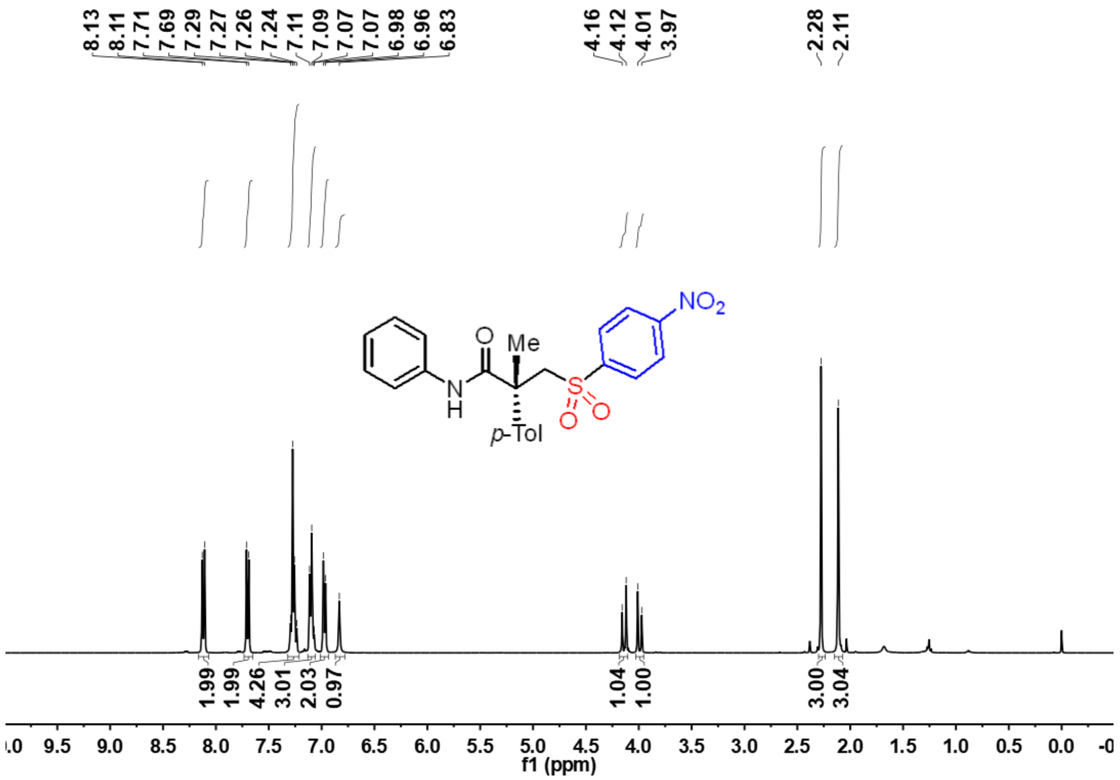

Supplementary Figure 81. ^{1}H NMR-spectrum of **4g**, recorded at 400 MHz and 25 °C in $CDCl_3$

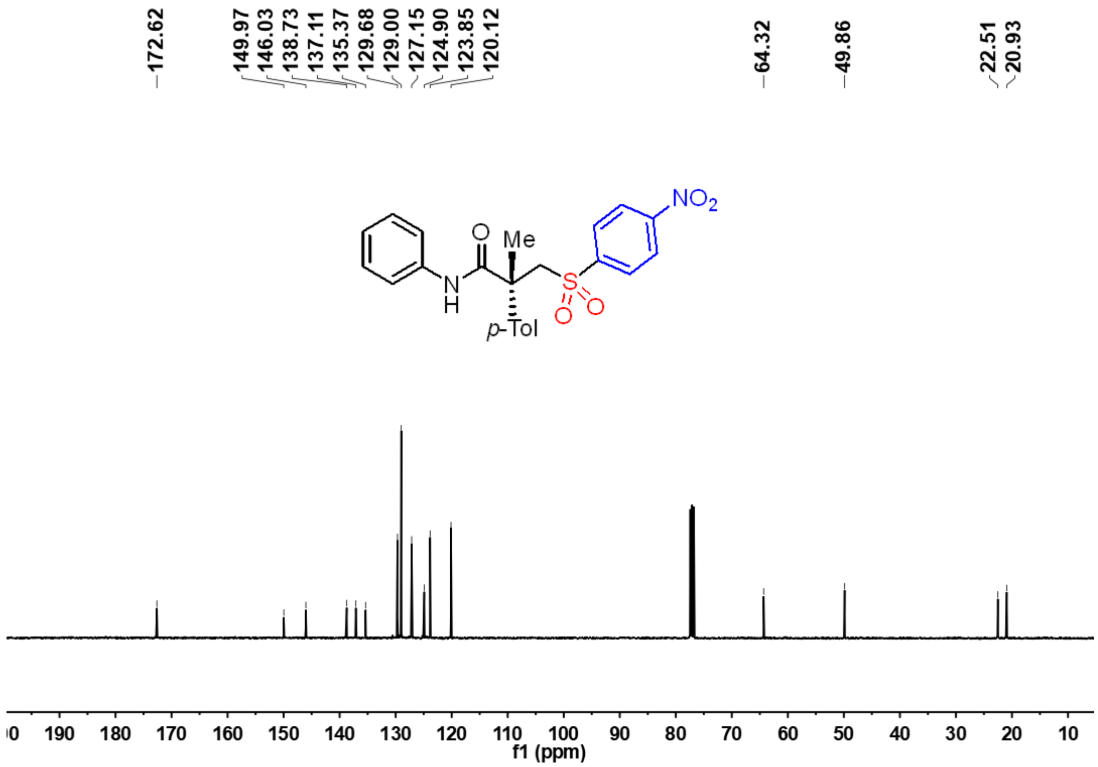

Supplementary Figure 82. ^{13}C NMR-spectrum of **4g**, recorded at 400 MHz and 25 °C in $CDCl_3$

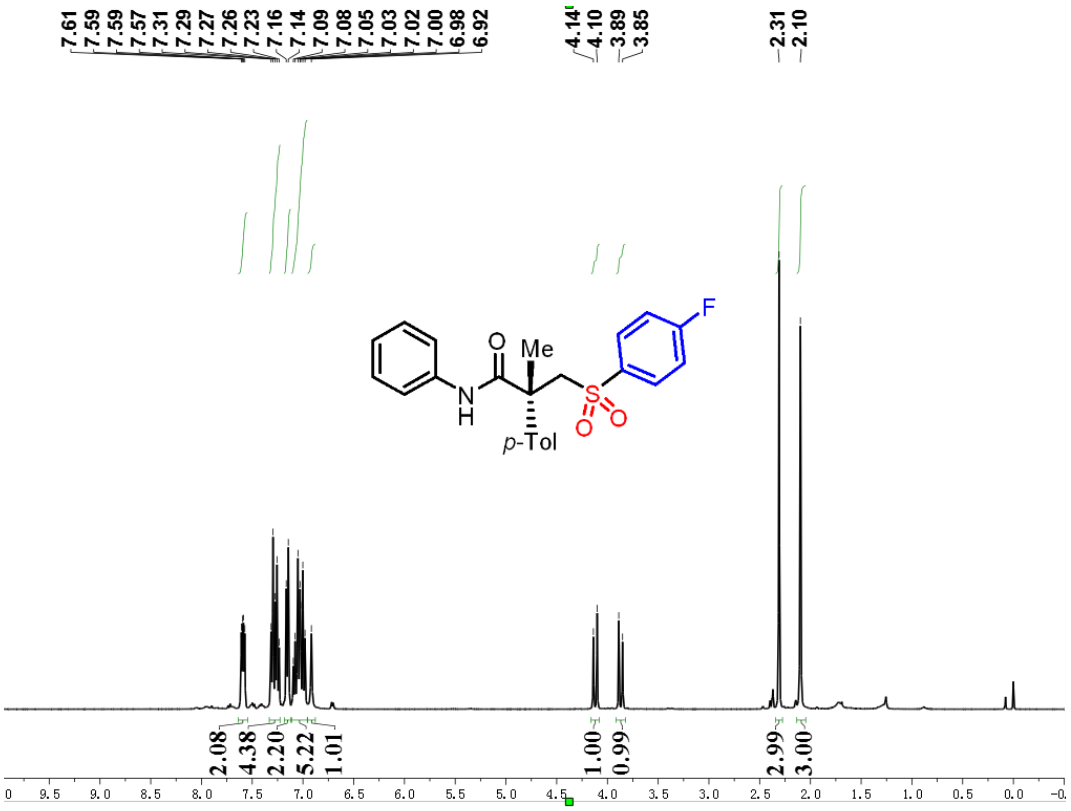

Supplementary Figure 83. ^{1}H NMR-spectrum of **4h**, recorded at 400 MHz and 25 °C in $CDCl_3$

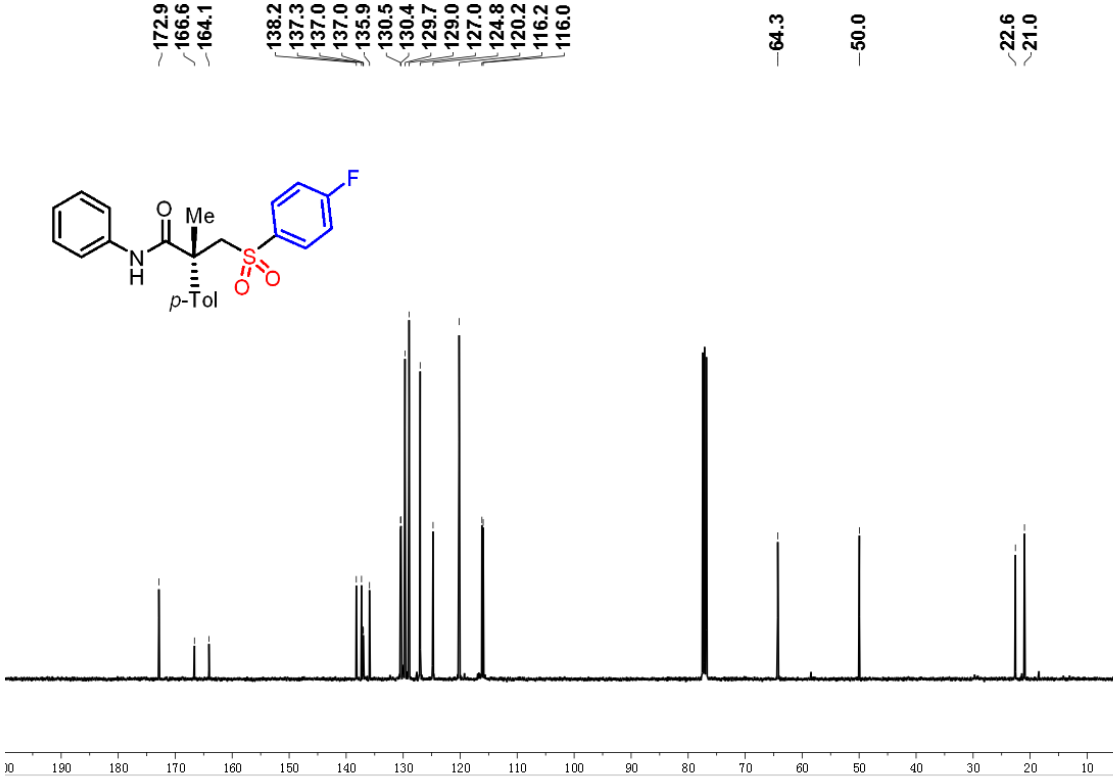

Supplementary Figure 84. ^{13}C NMR-spectrum of **4h**, recorded at 400 MHz and 25 °C in $CDCl_3$

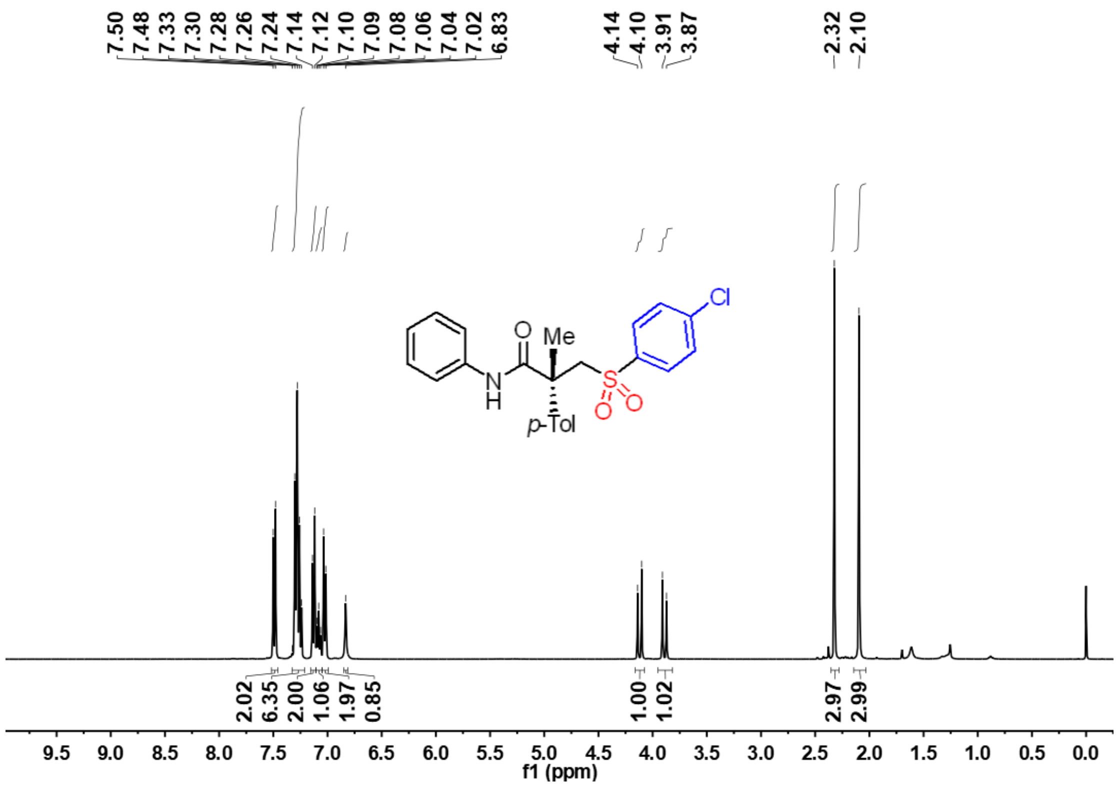

Supplementary Figure 85. ^{1}H NMR-spectrum of **4i**, recorded at 400 MHz and 25 °C in $CDCl_3$

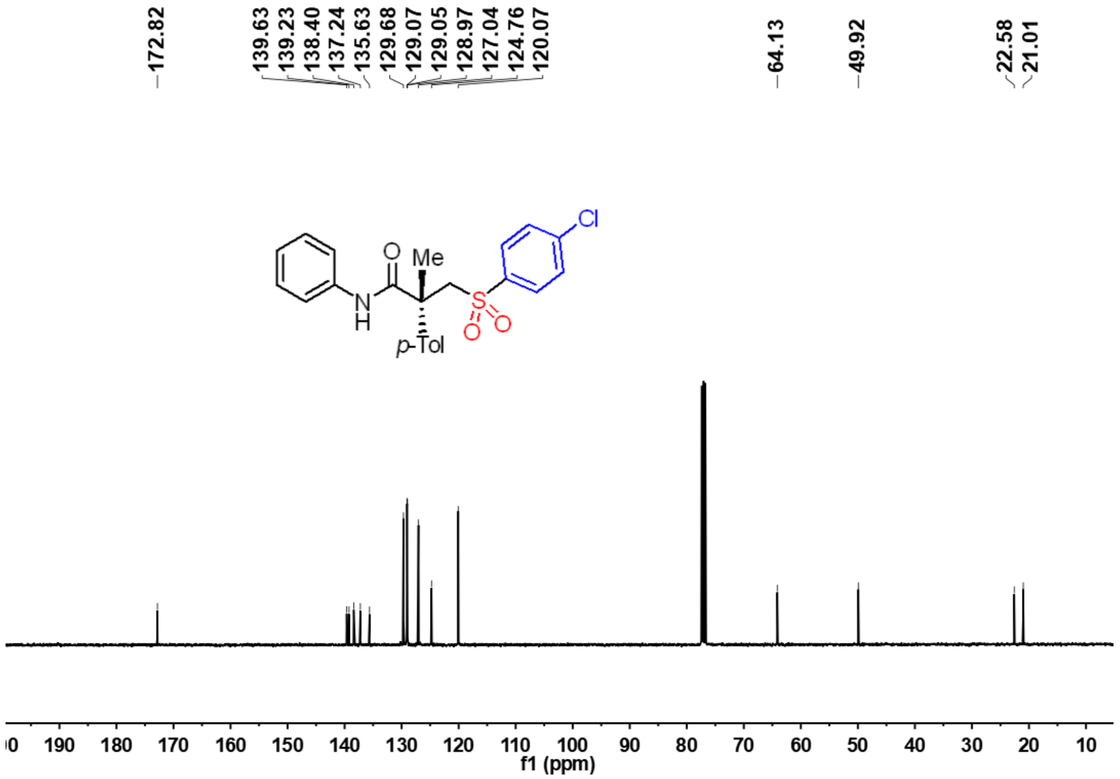

Supplementary Figure 86. ^{13}C NMR-spectrum of **4i**, recorded at 400 MHz and 25 °C in $CDCl_3$

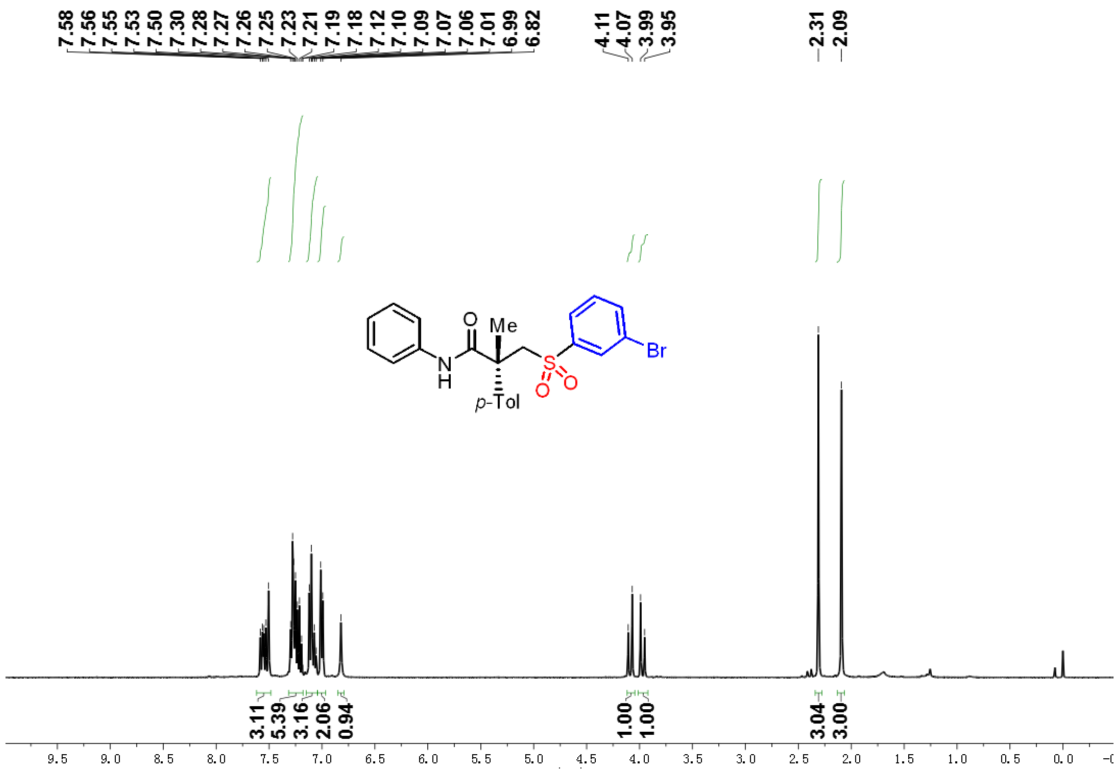

Supplementary Figure 87. ^{1}H NMR-spectrum of **4j**, recorded at 400 MHz and 25 °C in $CDCl_3$

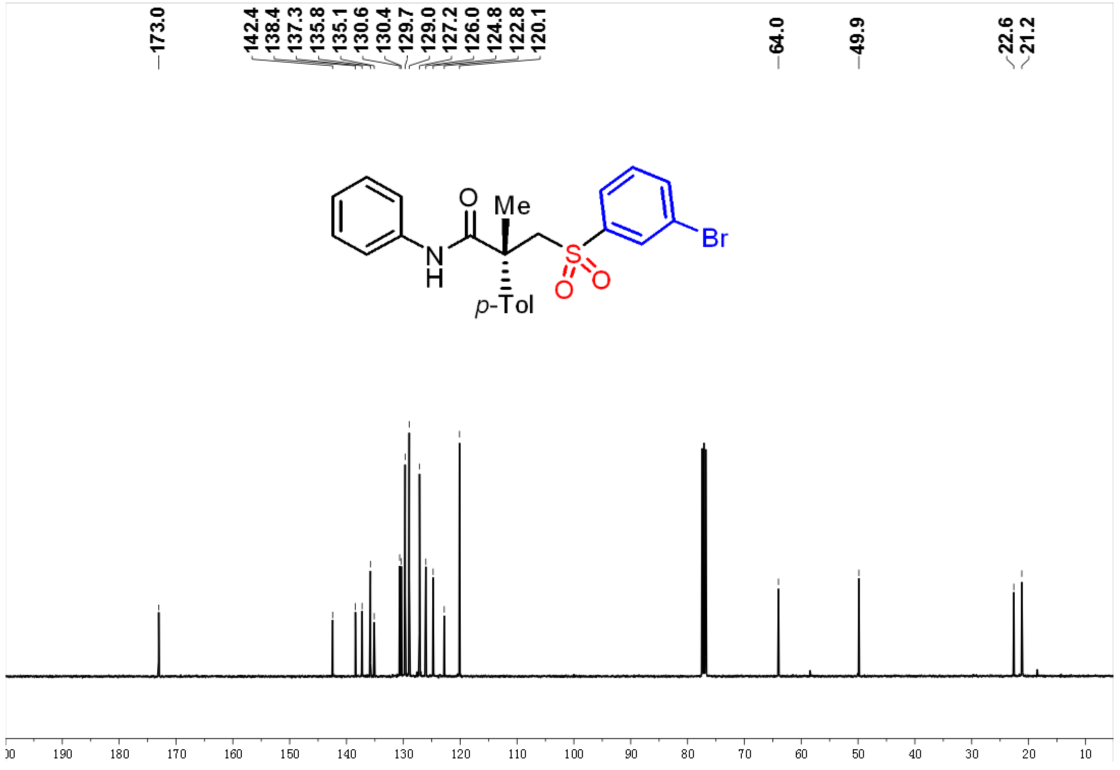

Supplementary Figure 88. ^{13}C NMR-spectrum of **4j**, recorded at 400 MHz and 25 °C in $CDCl_3$

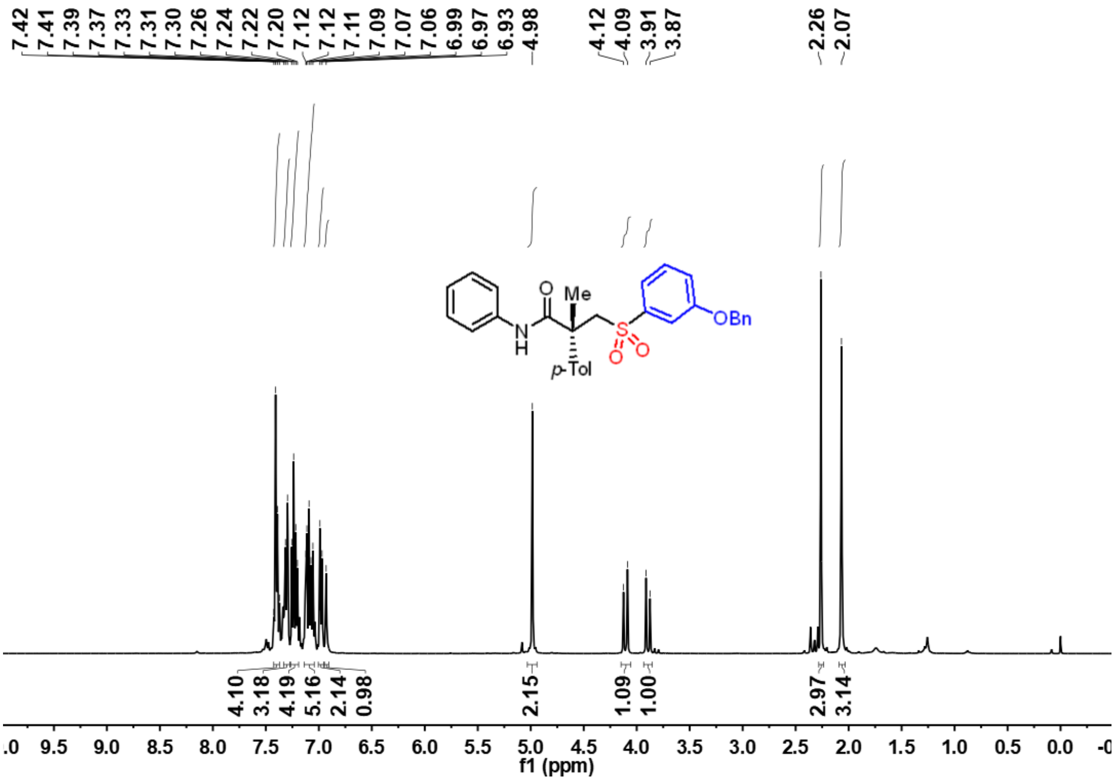

Supplementary Figure 89. ^{1}H NMR-spectrum of **4k**, recorded at 400 MHz and 25 °C in $CDCl_3$

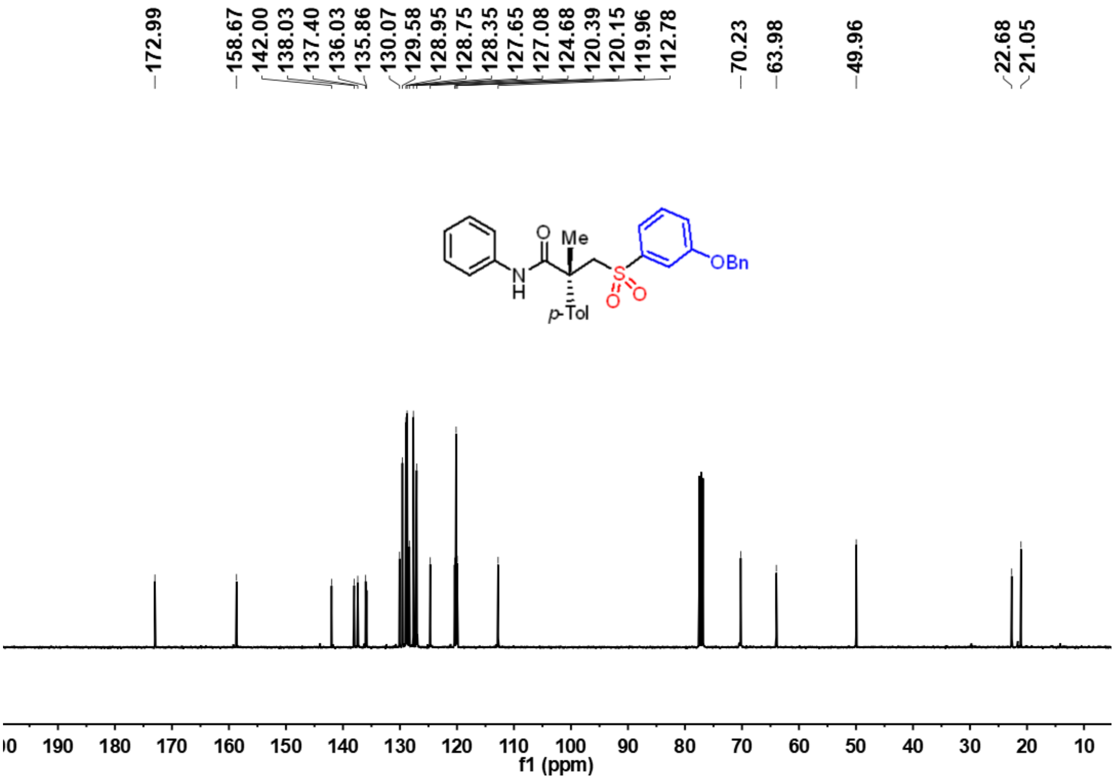

Supplementary Figure 90. ^{13}C NMR-spectrum of **4k**, recorded at 400 MHz and 25 °C in $CDCl_3$

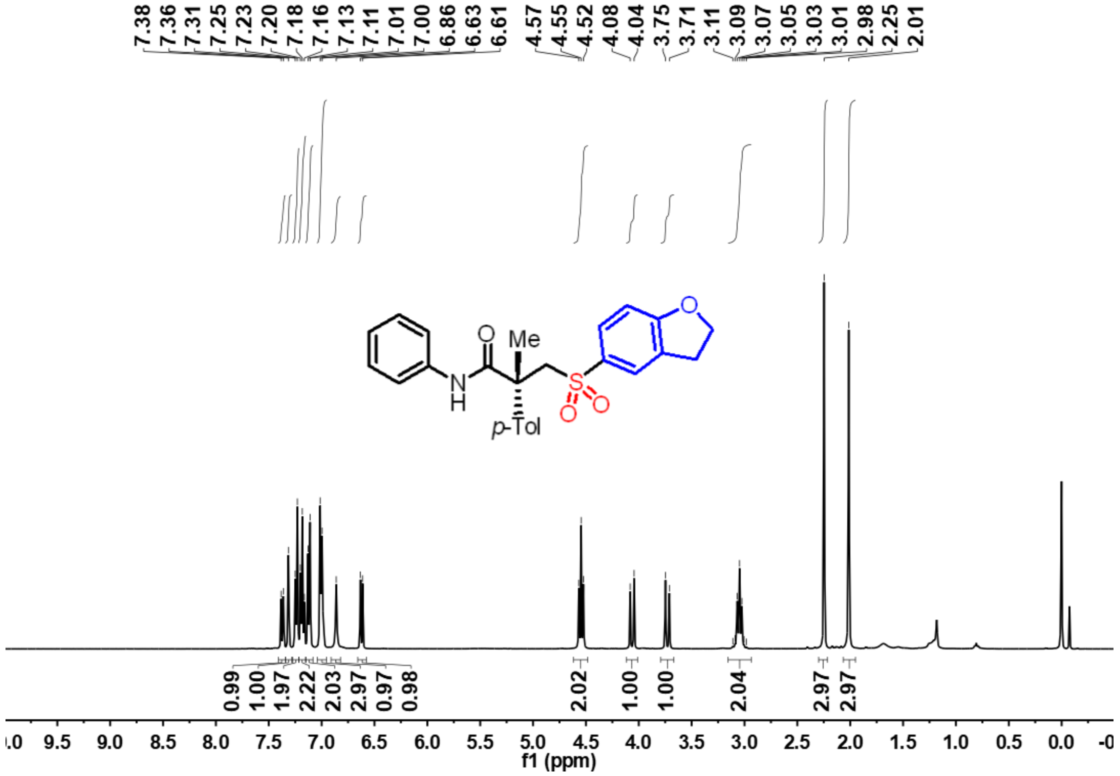

Supplementary Figure 91. ^{1}H NMR-spectrum of **4l**, recorded at 400 MHz and 25 °C in $CDCl_3$

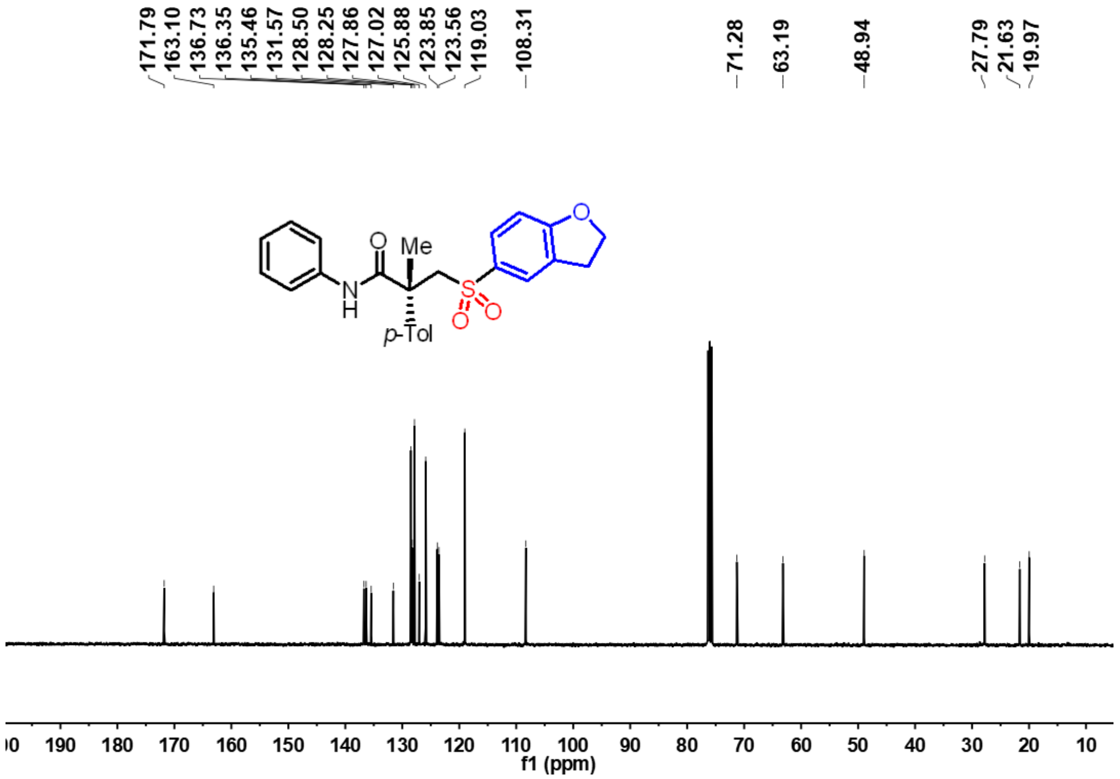

Supplementary Figure 92. ^{13}C NMR-spectrum of **4l**, recorded at 400 MHz and 25 °C in $CDCl_3$

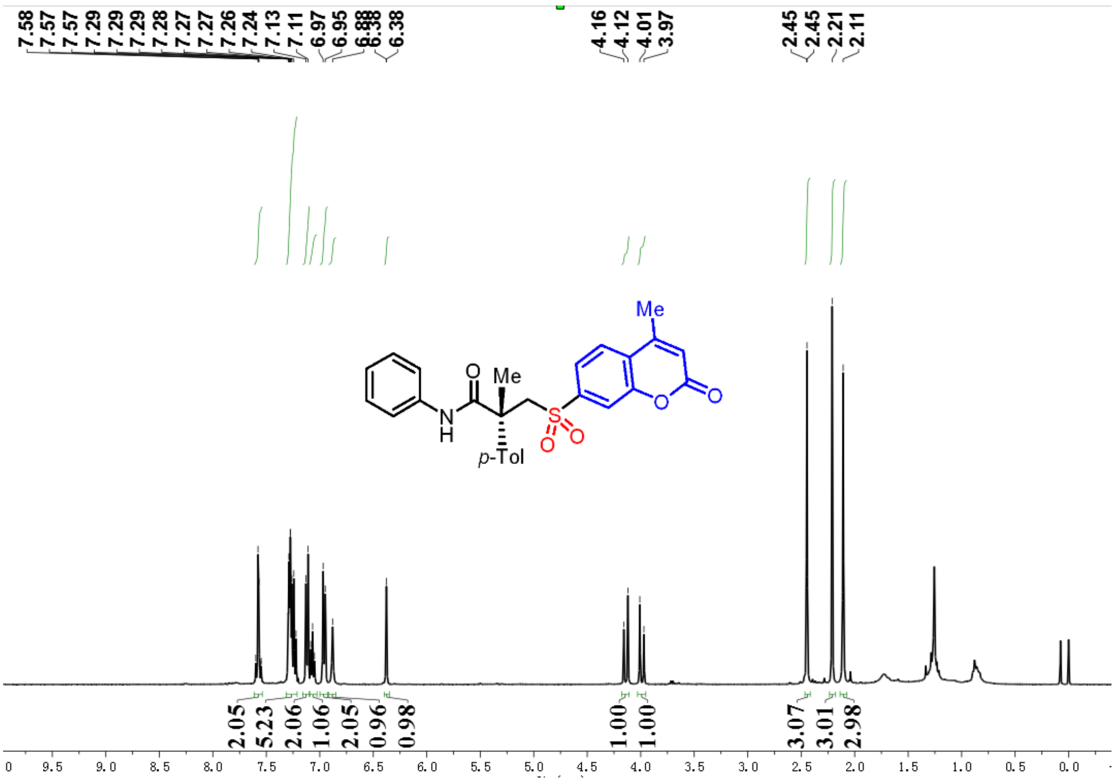

Supplementary Figure 93. ^{1}H NMR-spectrum of **4m**, recorded at 400 MHz and 25 °C in $CDCl_3$

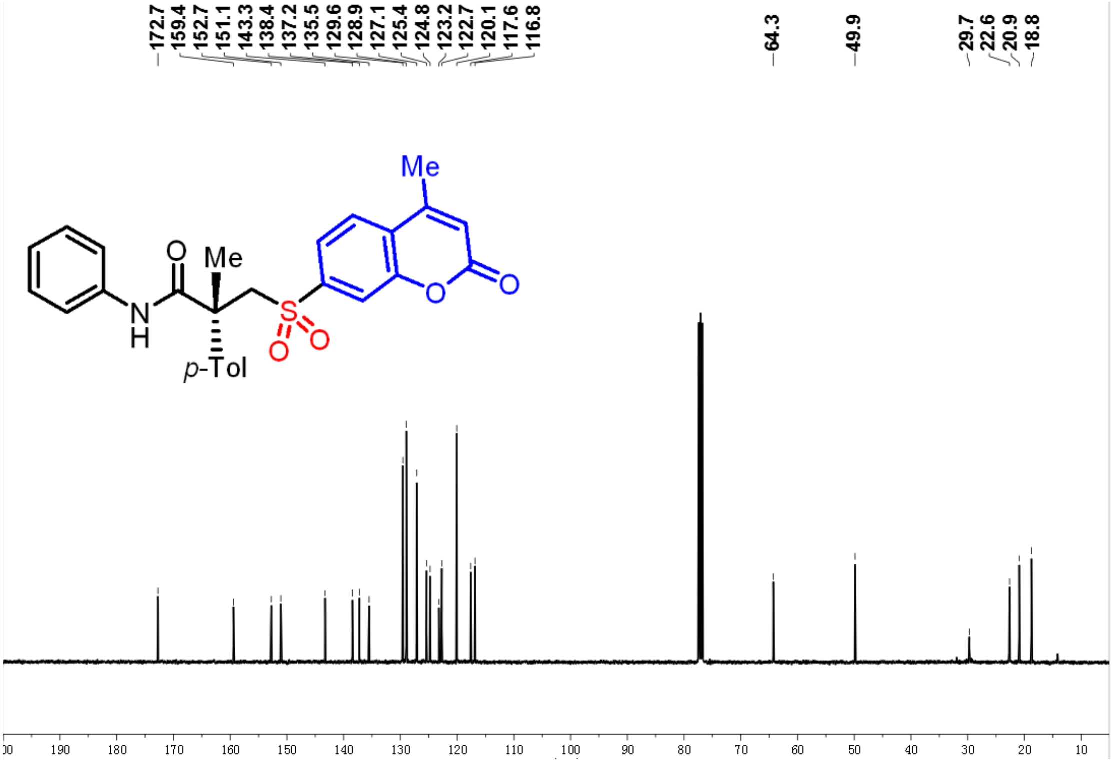

Supplementary Figure 94. ^{13}C NMR-spectrum of **4m**, recorded at 400 MHz and 25 °C in $CDCl_3$

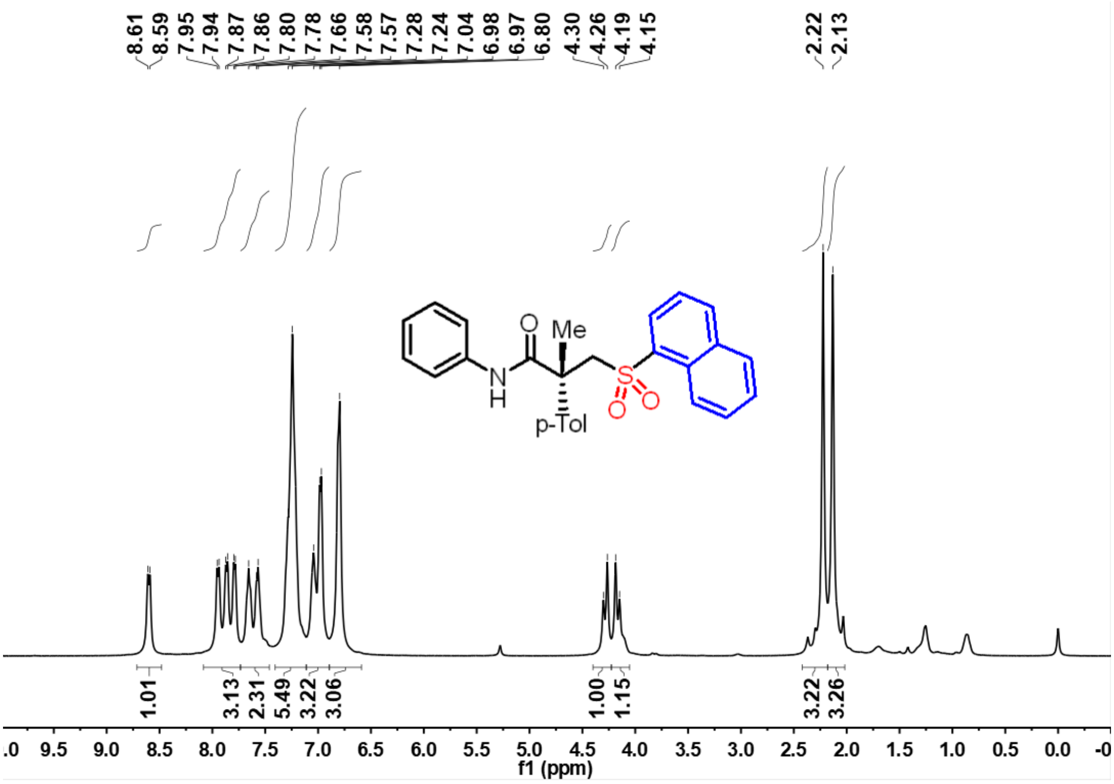

Supplementary Figure 95. ^{1}H NMR-spectrum of **4n**, recorded at 400 MHz and 25 °C in $CDCl_3$

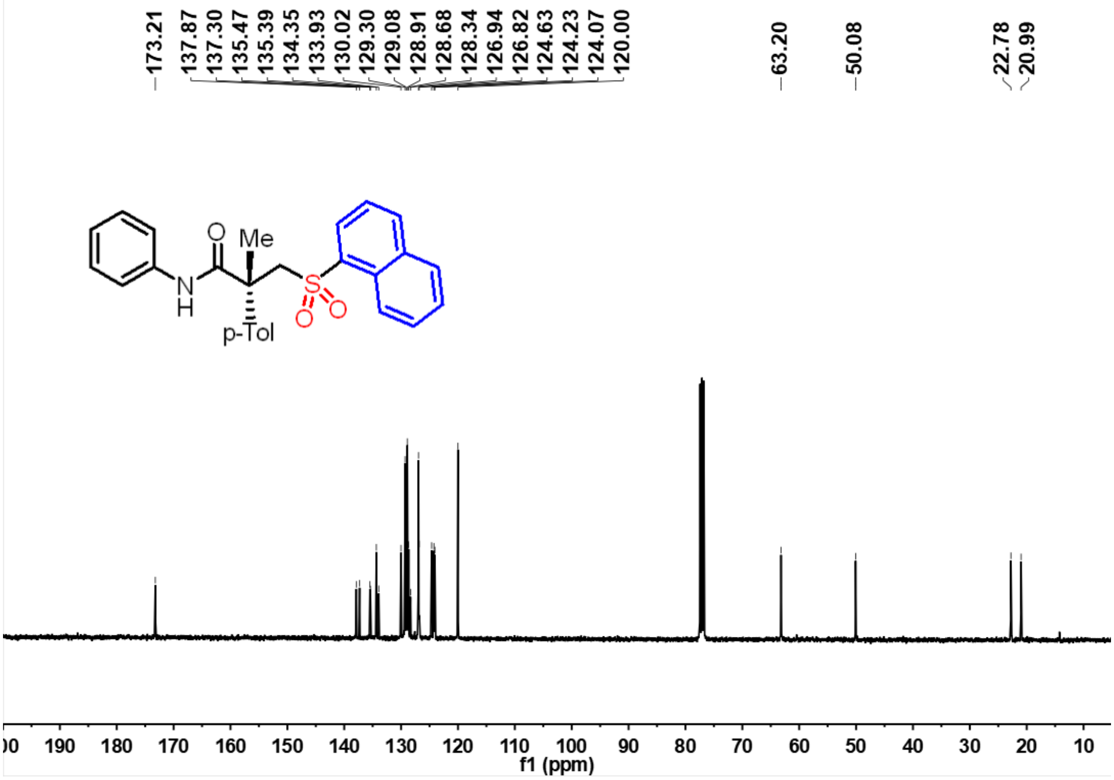

Supplementary Figure 96. ^{13}C NMR-spectrum of **4n**, recorded at 400 MHz and 25 °C in $CDCl_3$

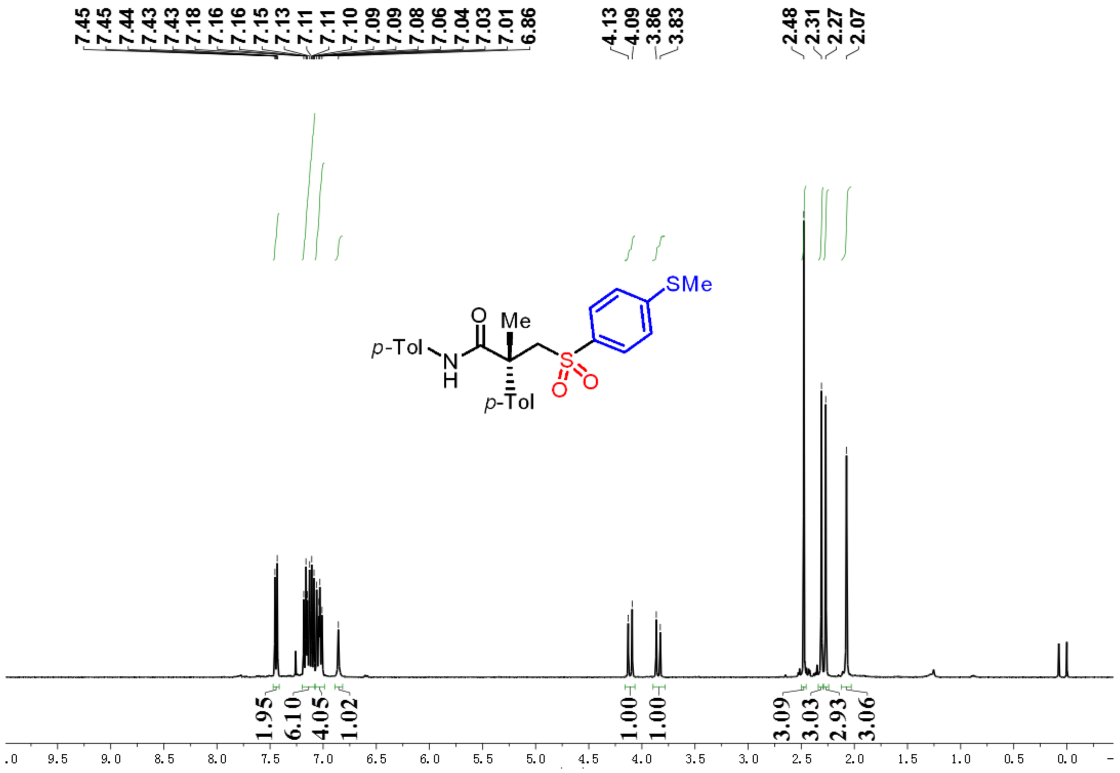

Supplementary Figure 97. ^{1}H NMR-spectrum of **4o**, recorded at 400 MHz and 25 °C in $CDCl_3$

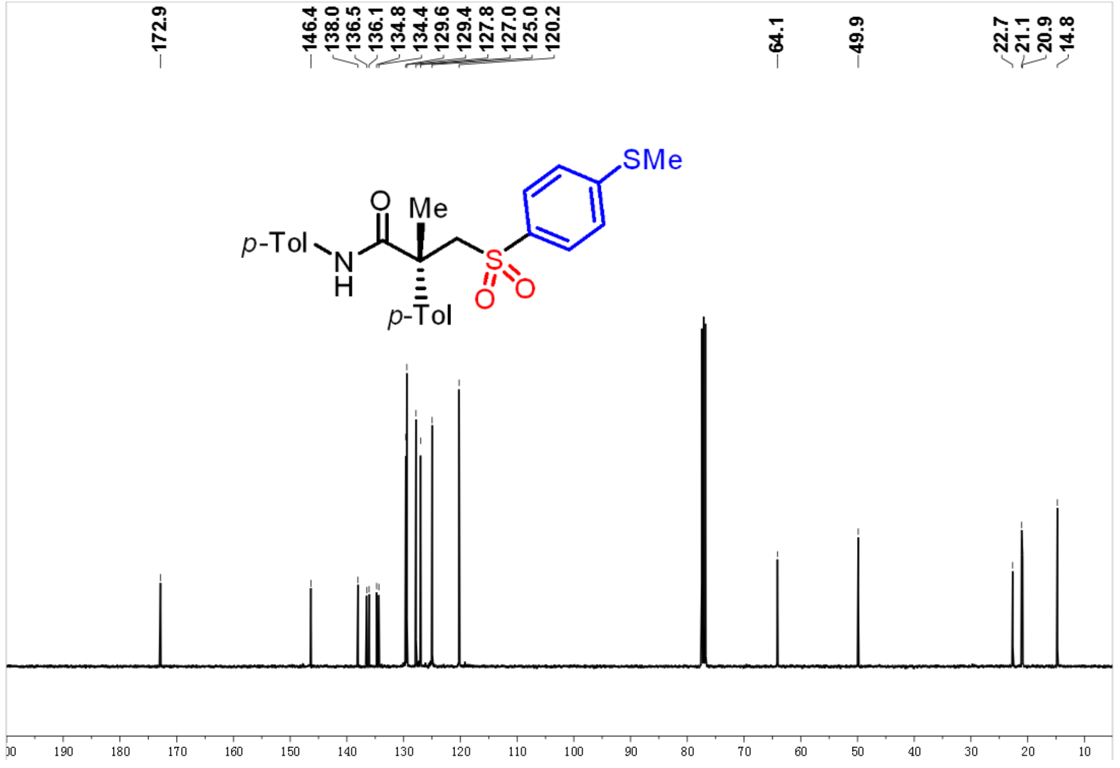

Supplementary Figure 98. ^{13}C NMR-spectrum of **4o**, recorded at 400 MHz and 25 °C in $CDCl_3$

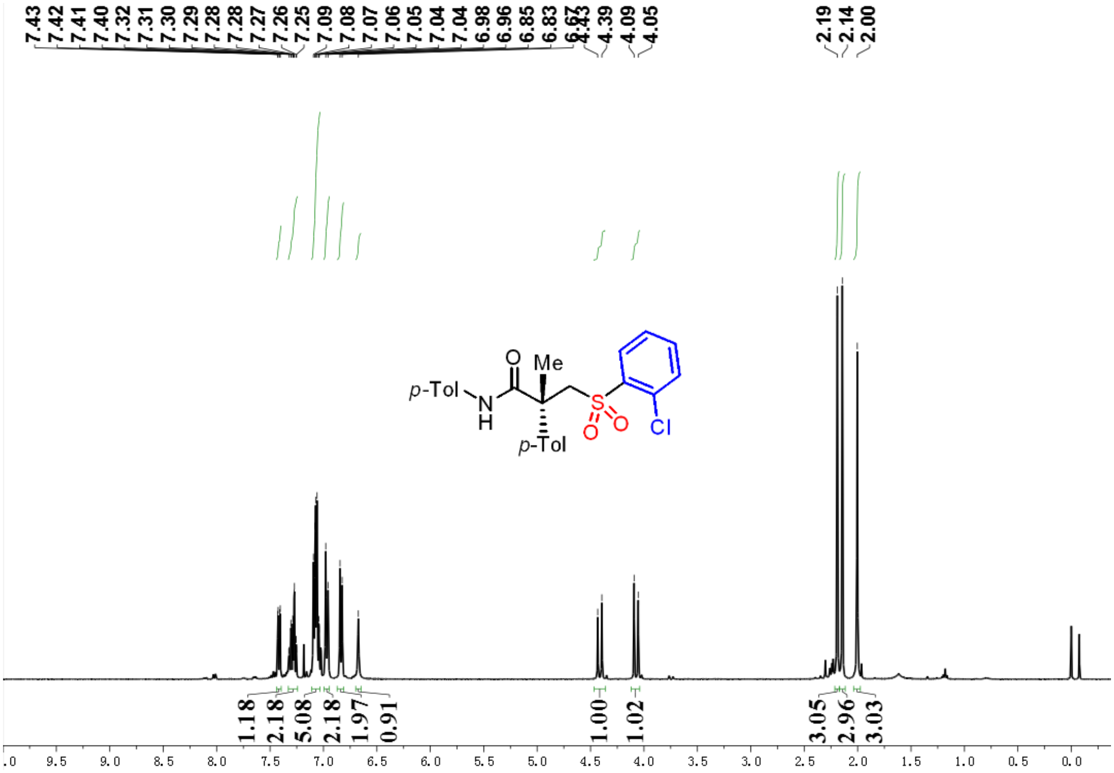

Supplementary Figure 99. ^{1}H NMR-spectrum of **4p**, recorded at 400 MHz and 25 °C in $CDCl_3$

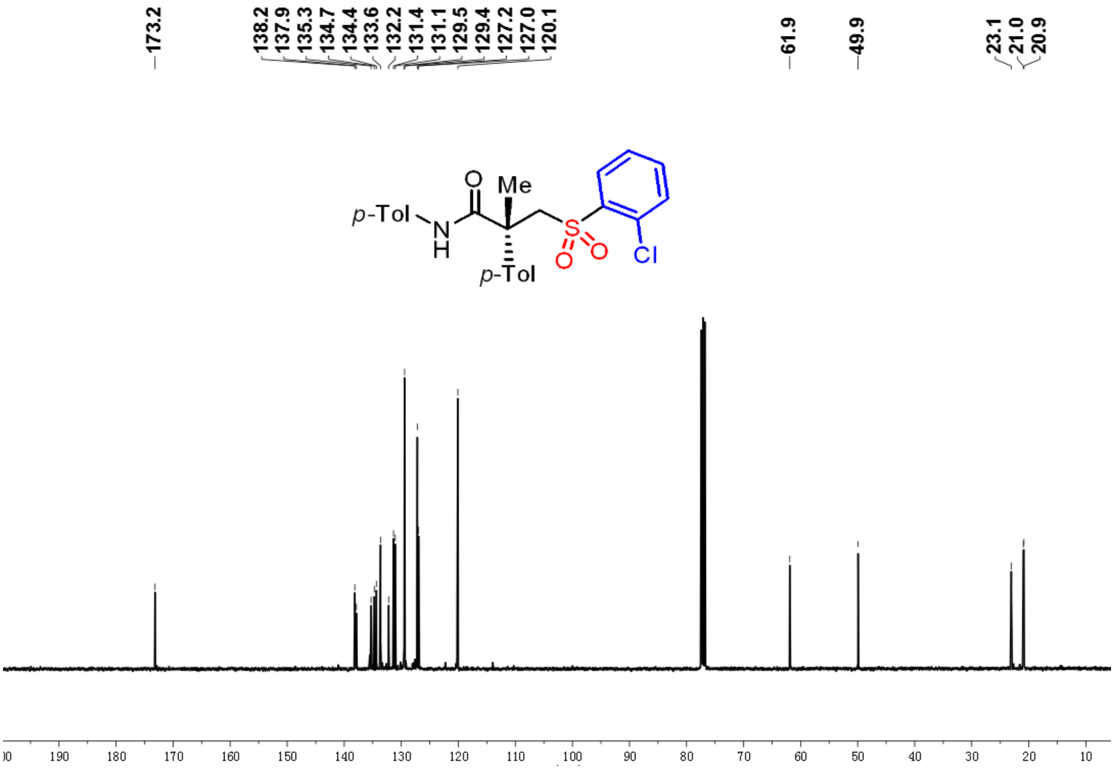

Supplementary Figure 100. ^{13}C NMR-spectrum of **4p**, recorded at 400 MHz and 25 °C in $CDCl_3$

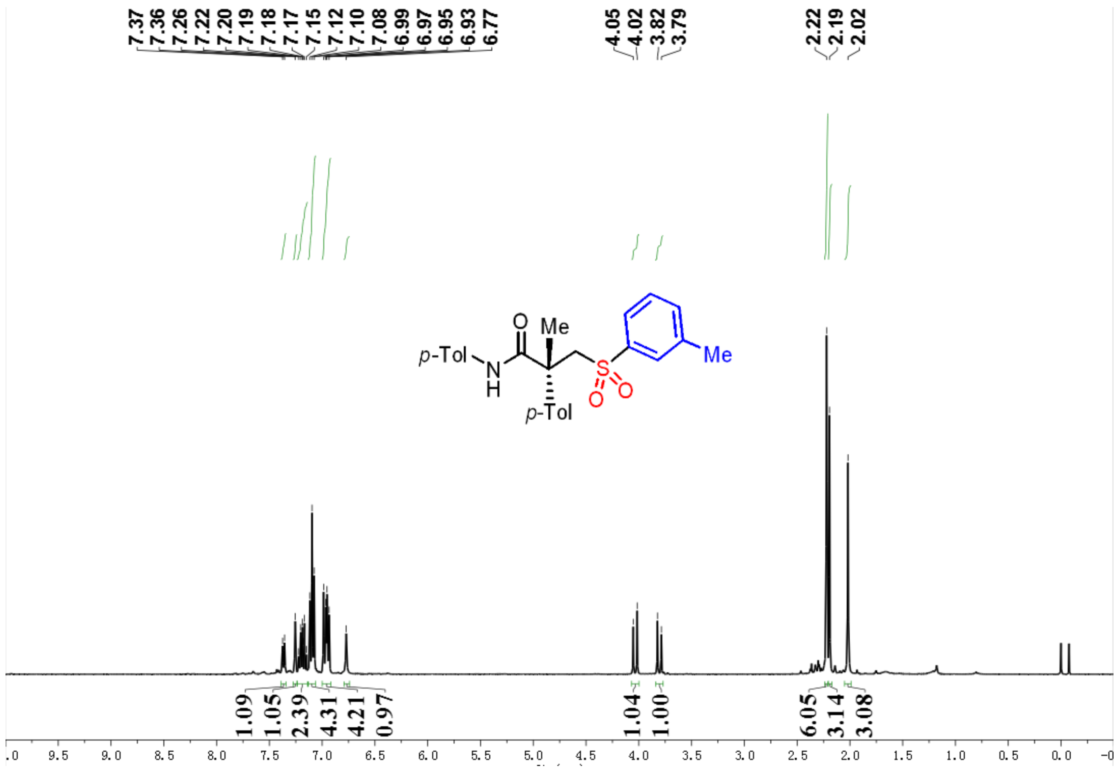

Supplementary Figure 101. ^{1}H NMR-spectrum of **4q**, recorded at 400 MHz and 25 °C in $CDCl_3$

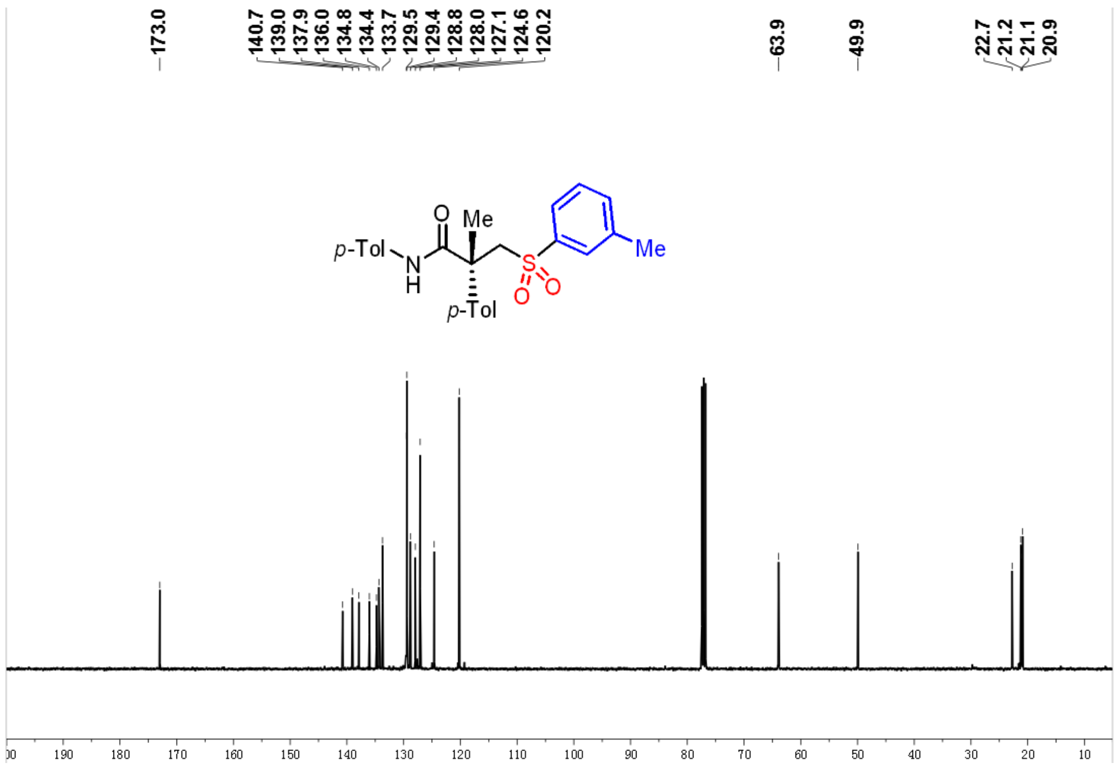

Supplementary Figure 102. ^{13}C NMR-spectrum of **4q**, recorded at 400 MHz and 25 °C in $CDCl_3$

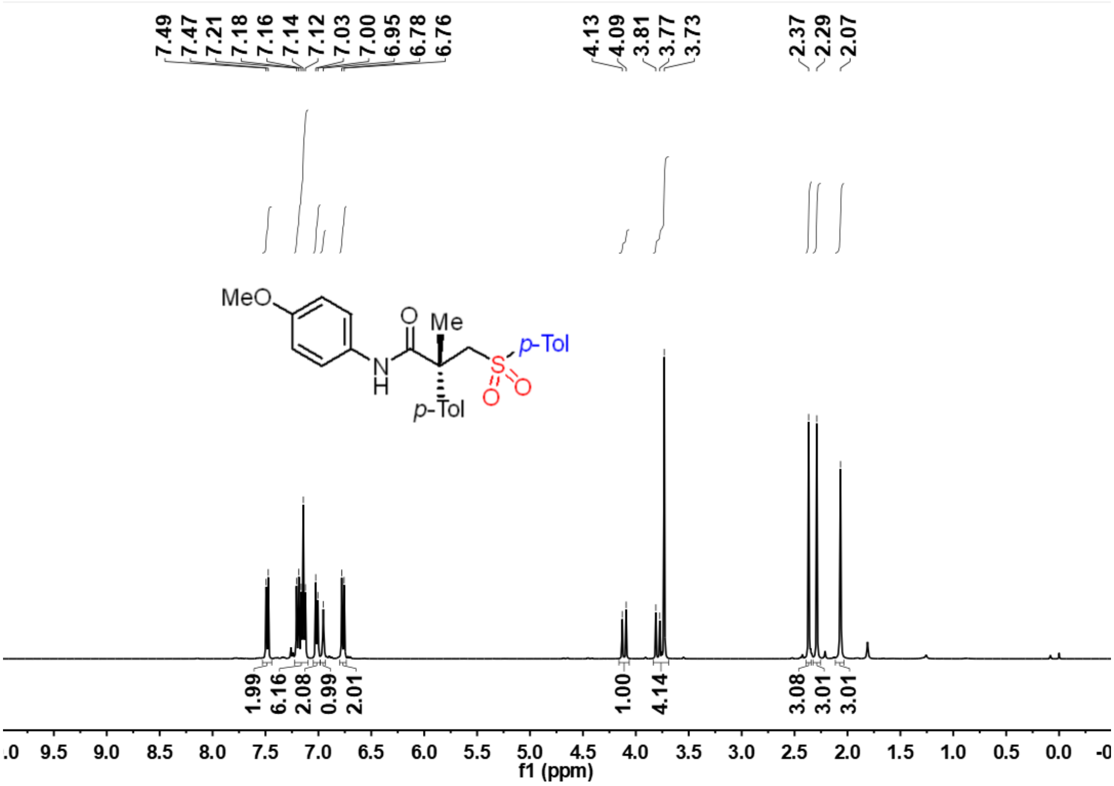

Supplementary Figure 103. ^{1}H NMR-spectrum of **4r**, recorded at 400 MHz and 25 °C in $CDCl_3$

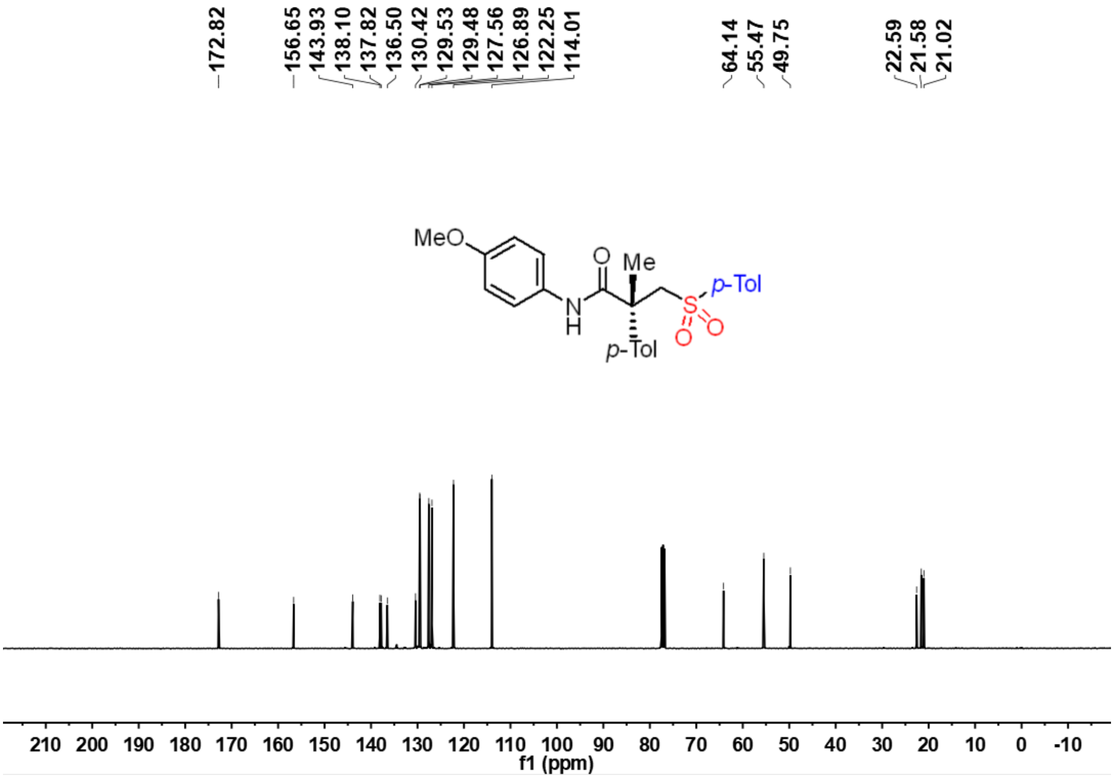

Supplementary Figure 104. ^{13}C NMR-spectrum of **4r**, recorded at 400 MHz and 25 °C in $CDCl_3$

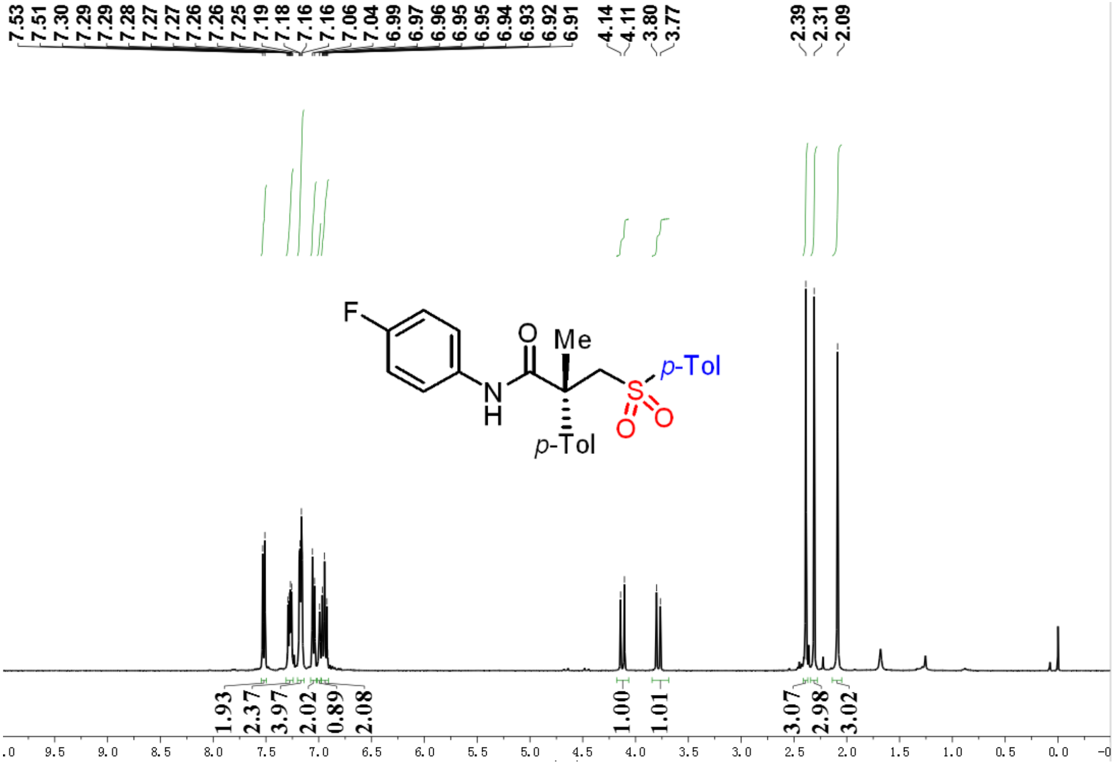

Supplementary Figure 105. ^{1}H NMR-spectrum of **4s**, recorded at 400 MHz and 25 °C in $CDCl_3$

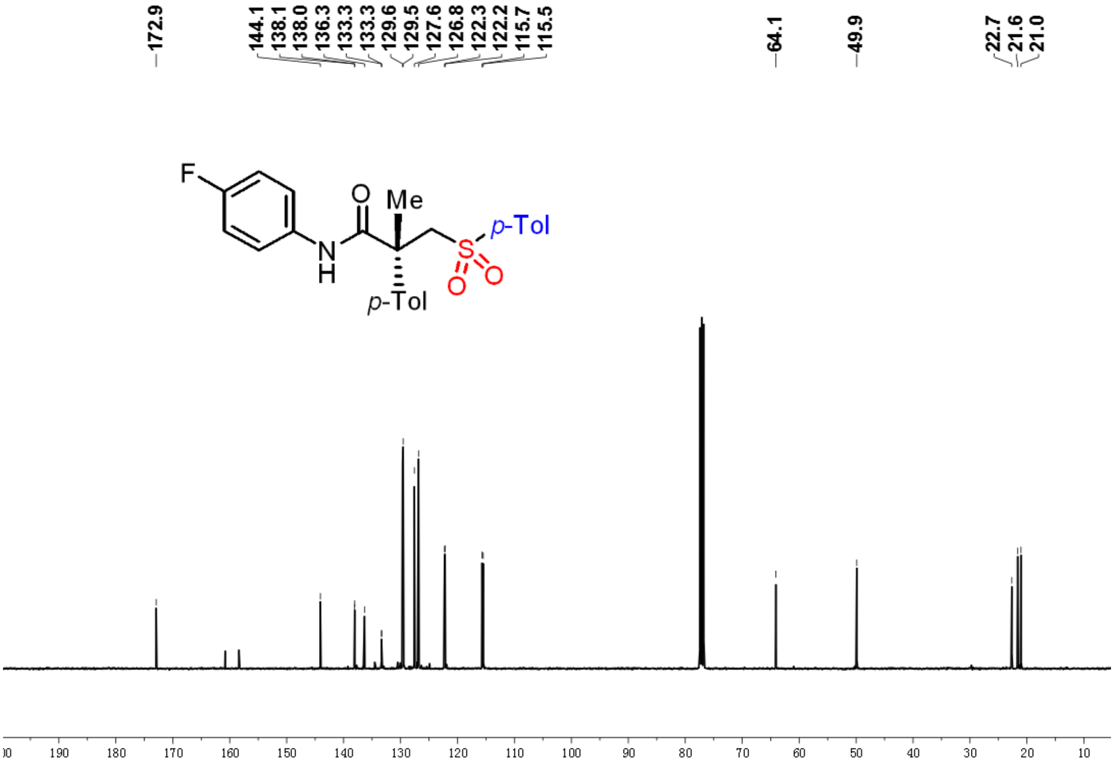

Supplementary Figure 106. ^{13}C NMR-spectrum of **4s**, recorded at 400 MHz and 25 °C in $CDCl_3$

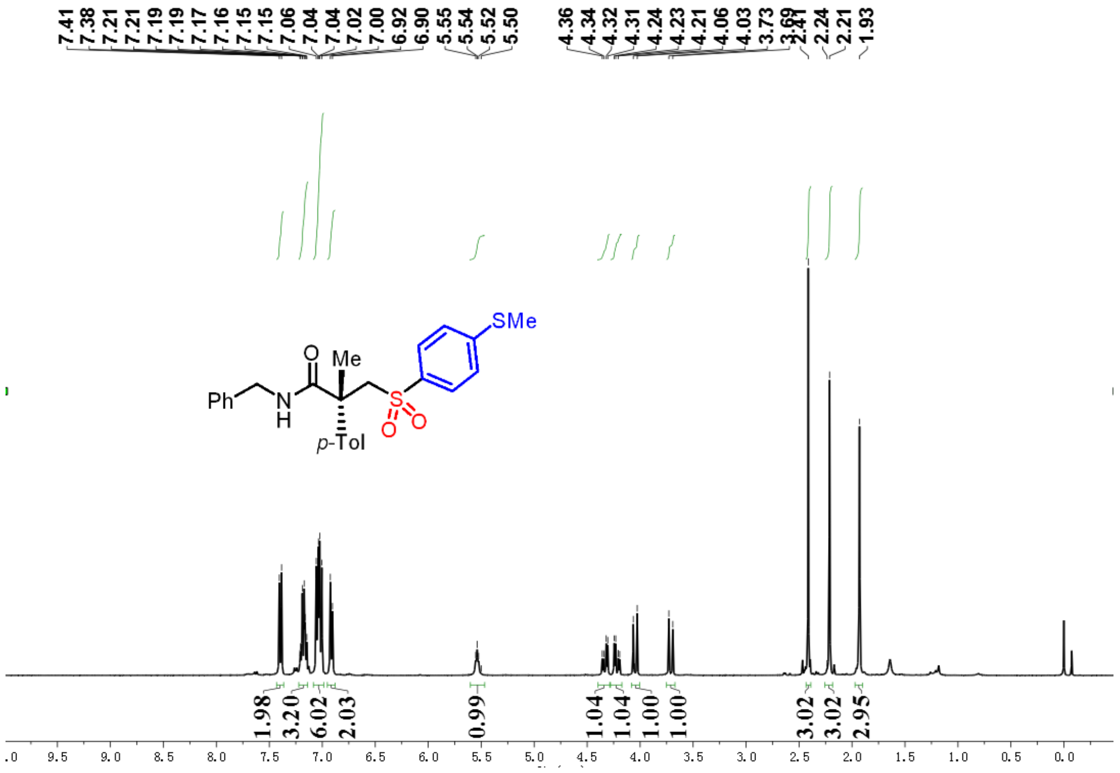

Supplementary Figure 107. ^{1}H NMR-spectrum of **4t**, recorded at 400 MHz and 25 °C in $CDCl_3$

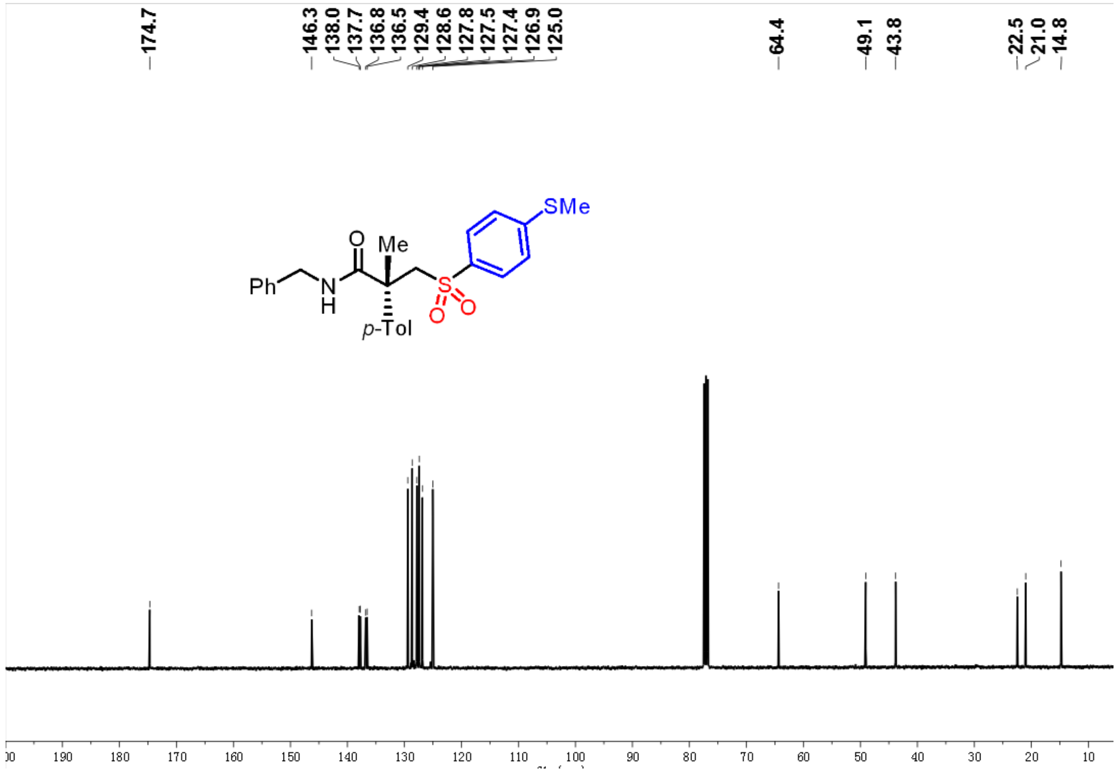

Supplementary Figure 108. ^{13}C NMR-spectrum of **4t**, recorded at 400 MHz and 25 °C in $CDCl_3$

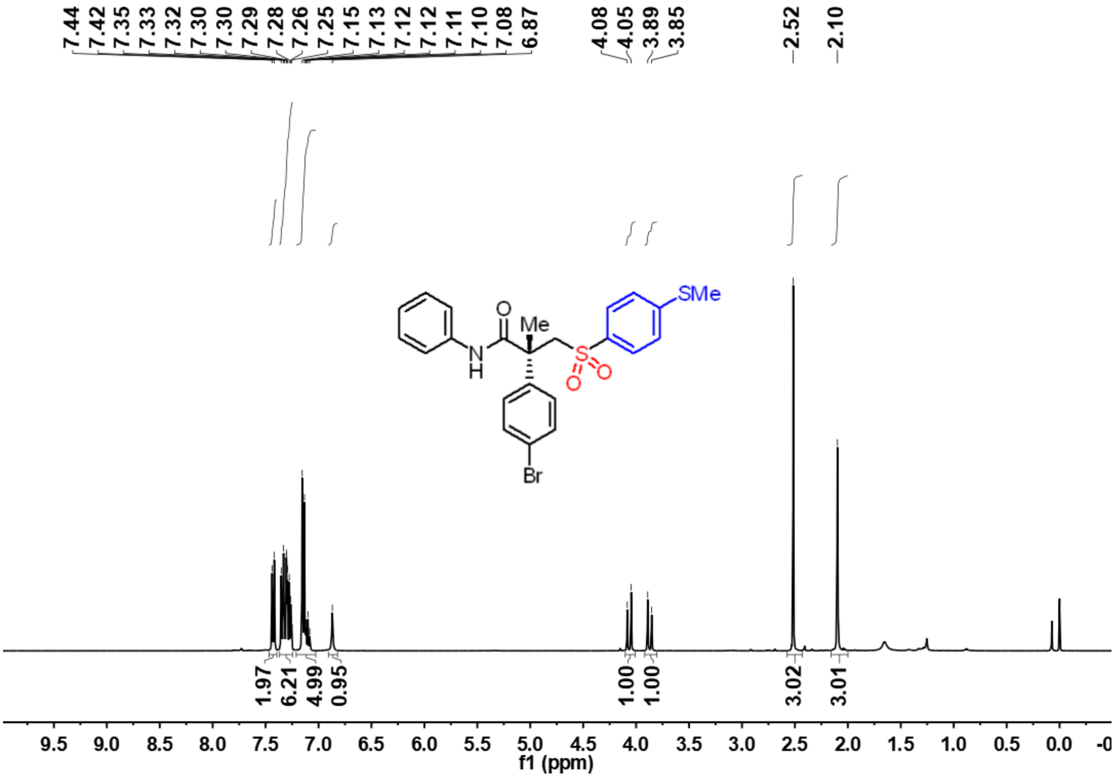

Supplementary Figure 109. ^{1}H NMR-spectrum of **4u**, recorded at 400 MHz and 25 °C in $CDCl_3$

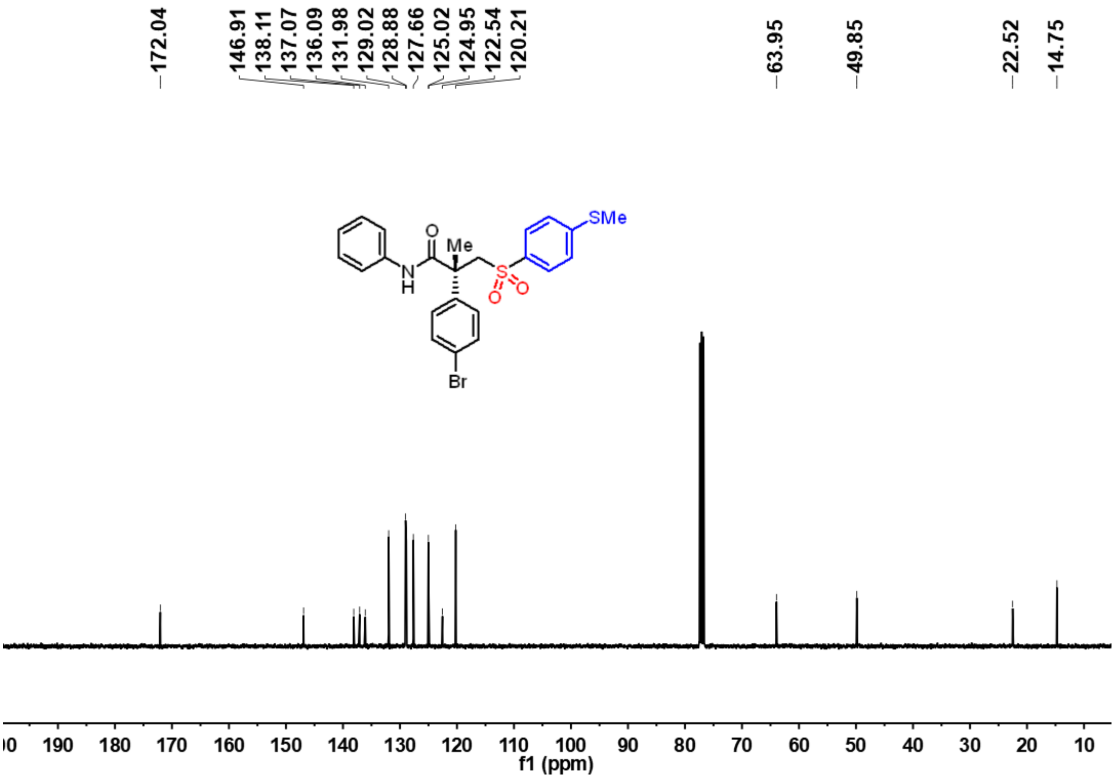

Supplementary Figure 110. ^{13}C NMR-spectrum of **4u**, recorded at 400 MHz and 25 °C in $CDCl_3$

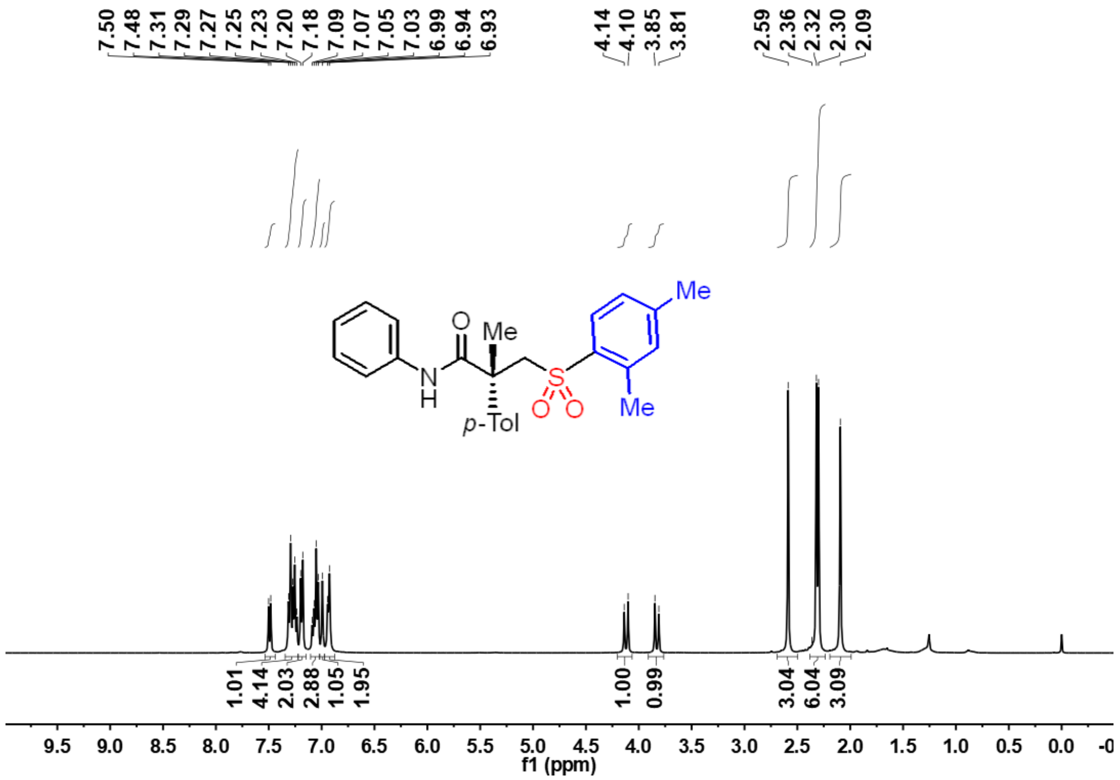

Supplementary Figure 111. ^{1}H NMR-spectrum of **4aa**, recorded at 400 MHz and 25 °C in $CDCl_3$

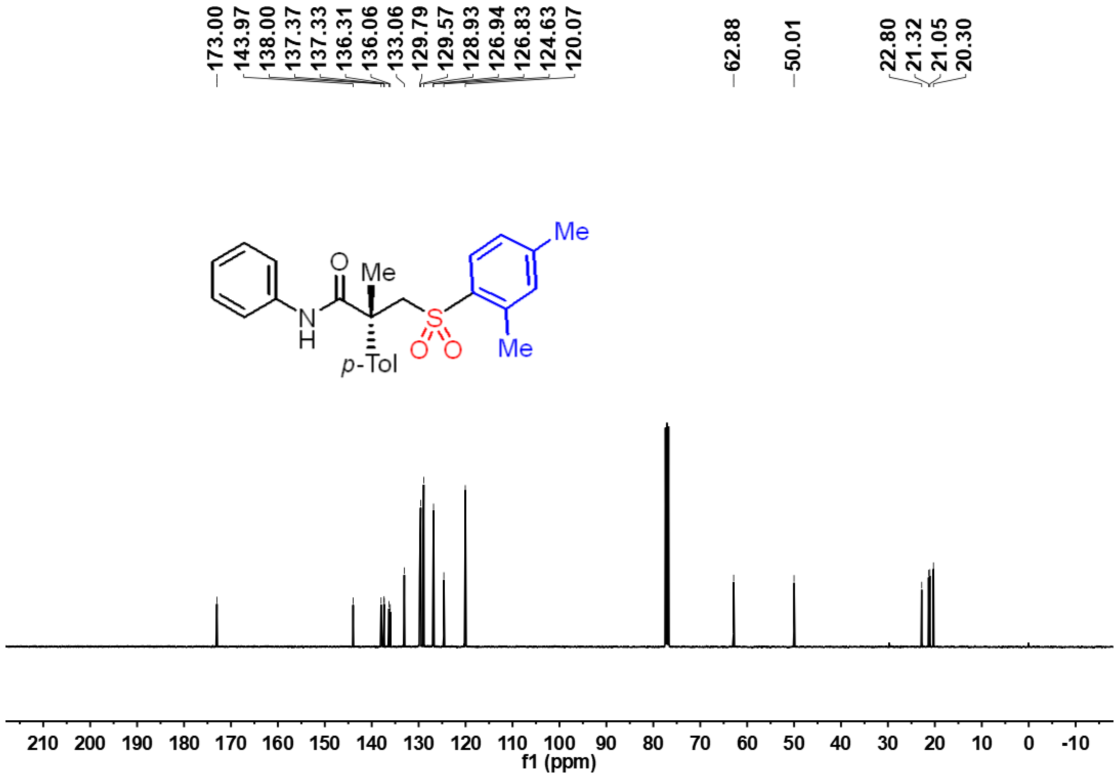

Supplementary Figure 112. ^{13}C NMR-spectrum of **4aa**, recorded at 400 MHz and 25 °C in $CDCl_3$

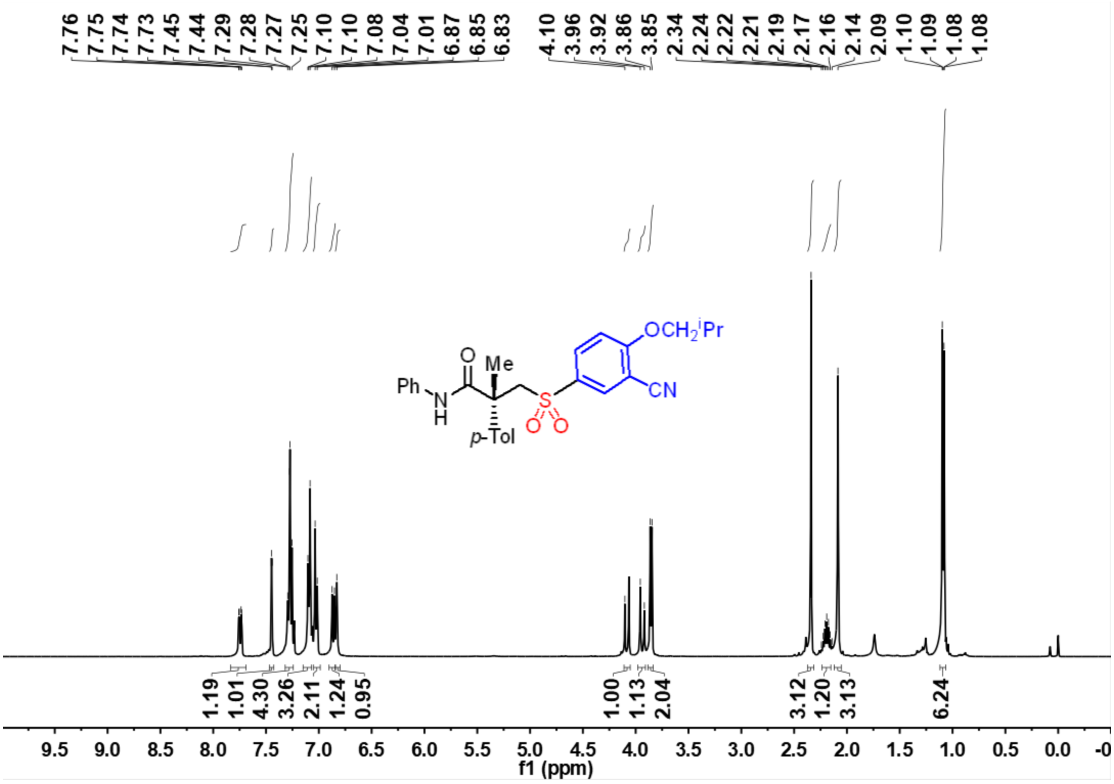

Supplementary Figure 113. ^{1}H NMR-spectrum of **4ab**, recorded at 400 MHz and 25 °C in $CDCl_3$

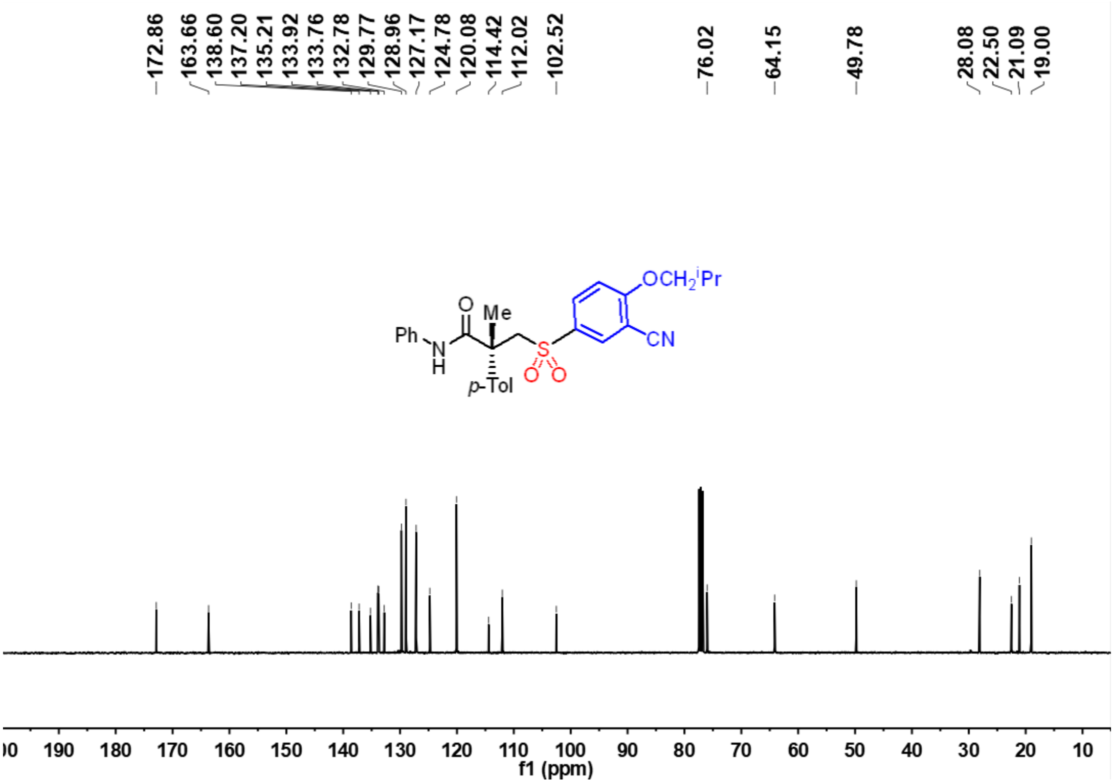

Supplementary Figure 114. ^{13}C NMR-spectrum of **4ab**, recorded at 400 MHz and 25 °C in $CDCl_3$

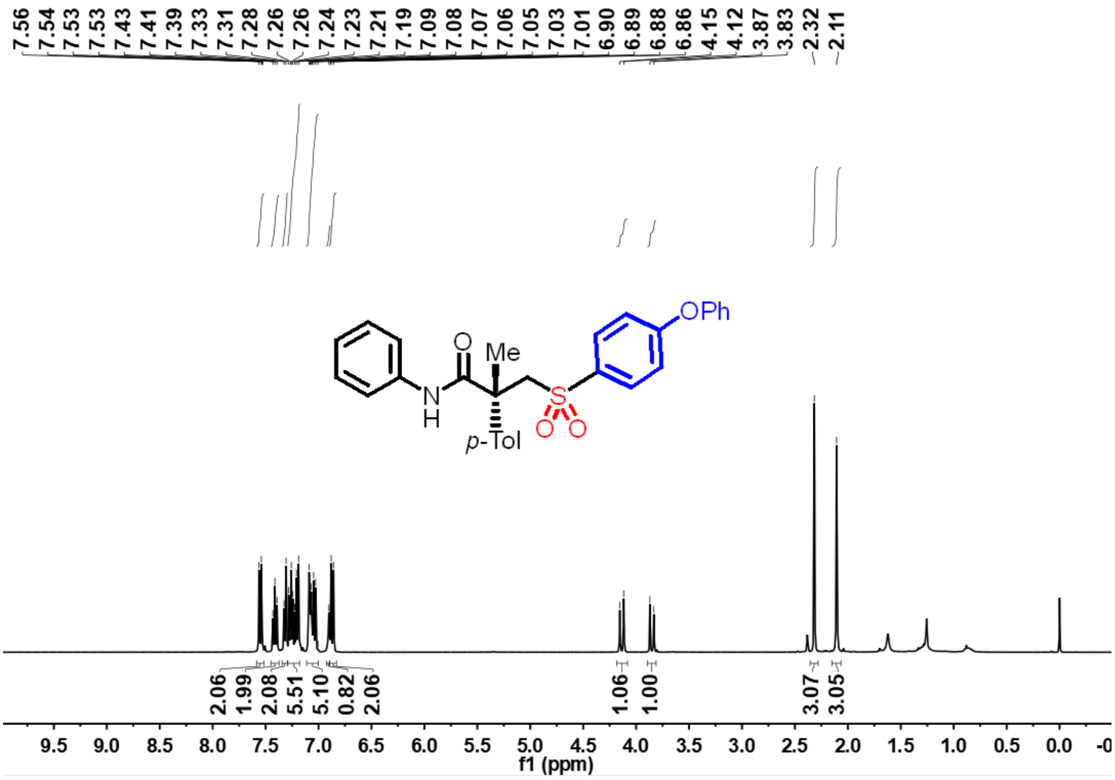

Supplementary Figure 115. ^{1}H NMR-spectrum of **4ac**, recorded at 400 MHz and 25 °C in $CDCl_3$

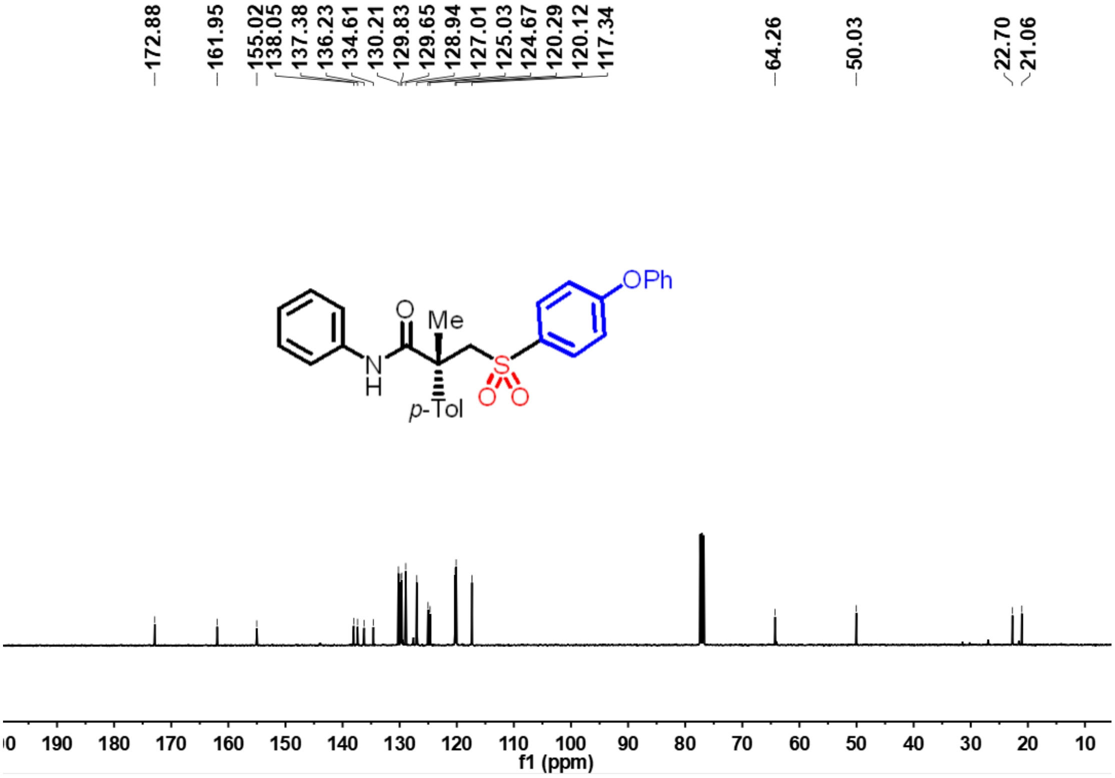

Supplementary Figure 116. ^{13}C NMR-spectrum of **4ac**, recorded at 400 MHz and 25 °C in $CDCl_3$

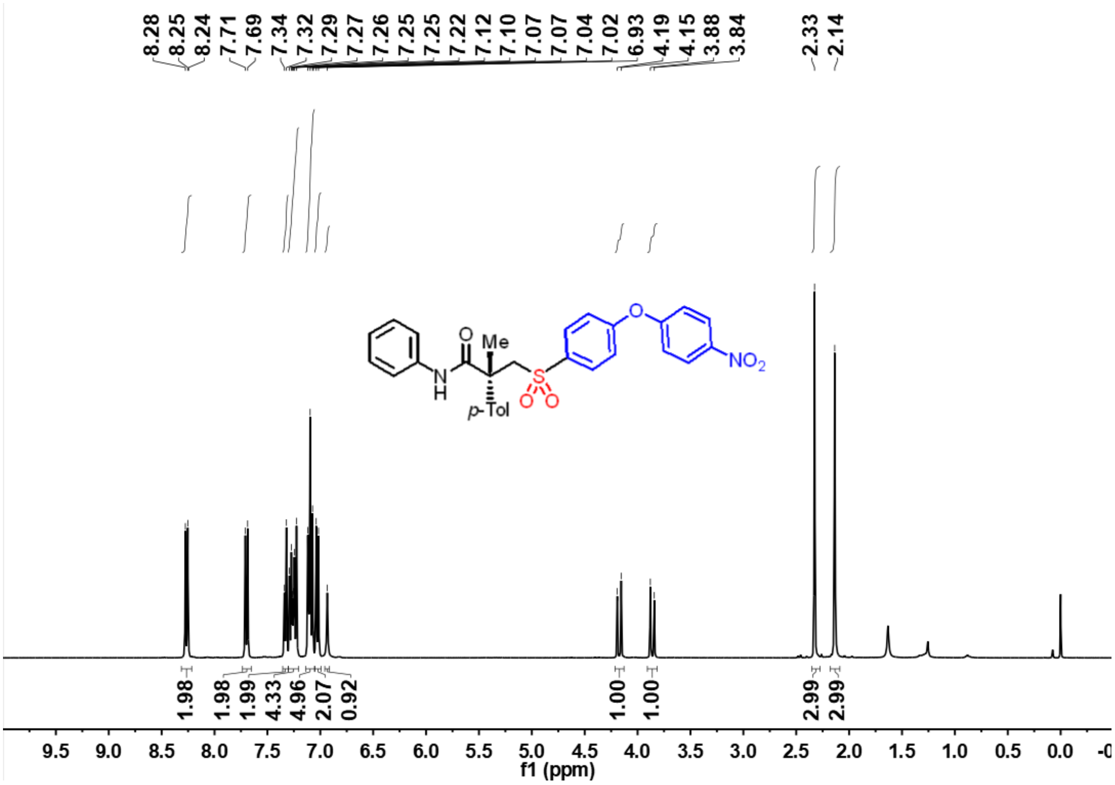

Supplementary Figure 117. ^{1}H NMR-spectrum of **4ad**, recorded at 400 MHz and 25 °C in $CDCl_3$

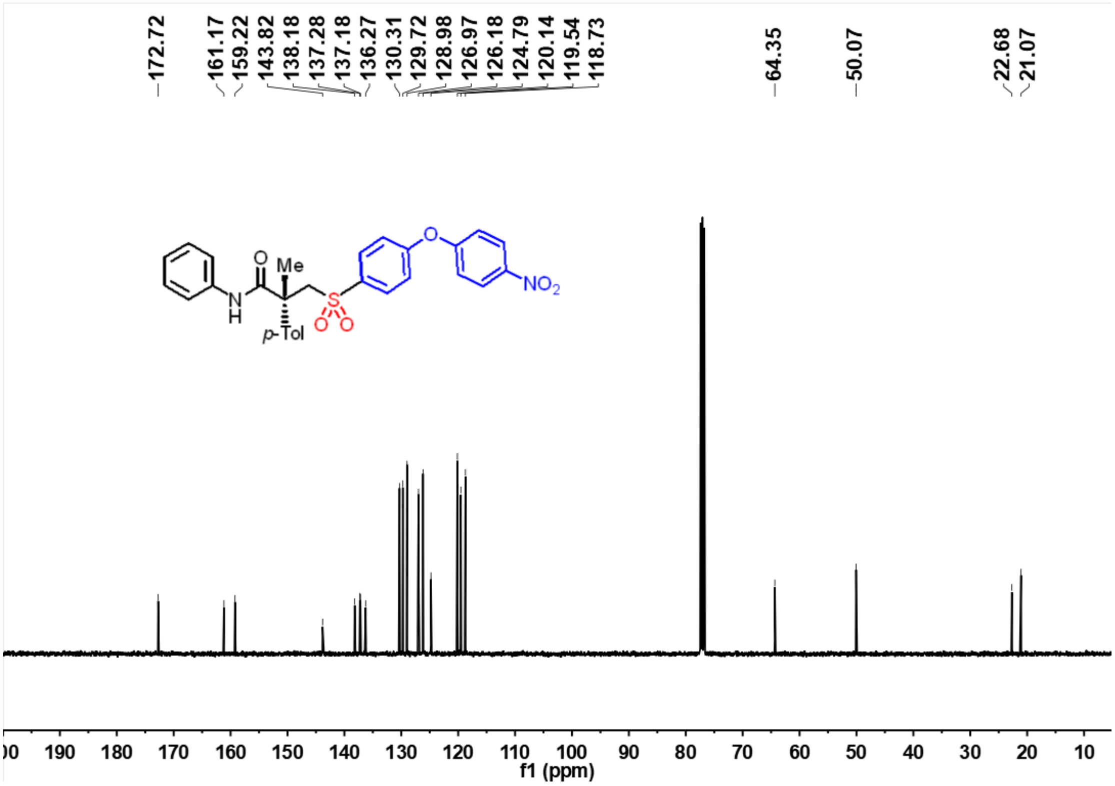

Supplementary Figure 118. ^{13}C NMR-spectrum of **4ad**, recorded at 400 MHz and 25 °C in $CDCl_3$

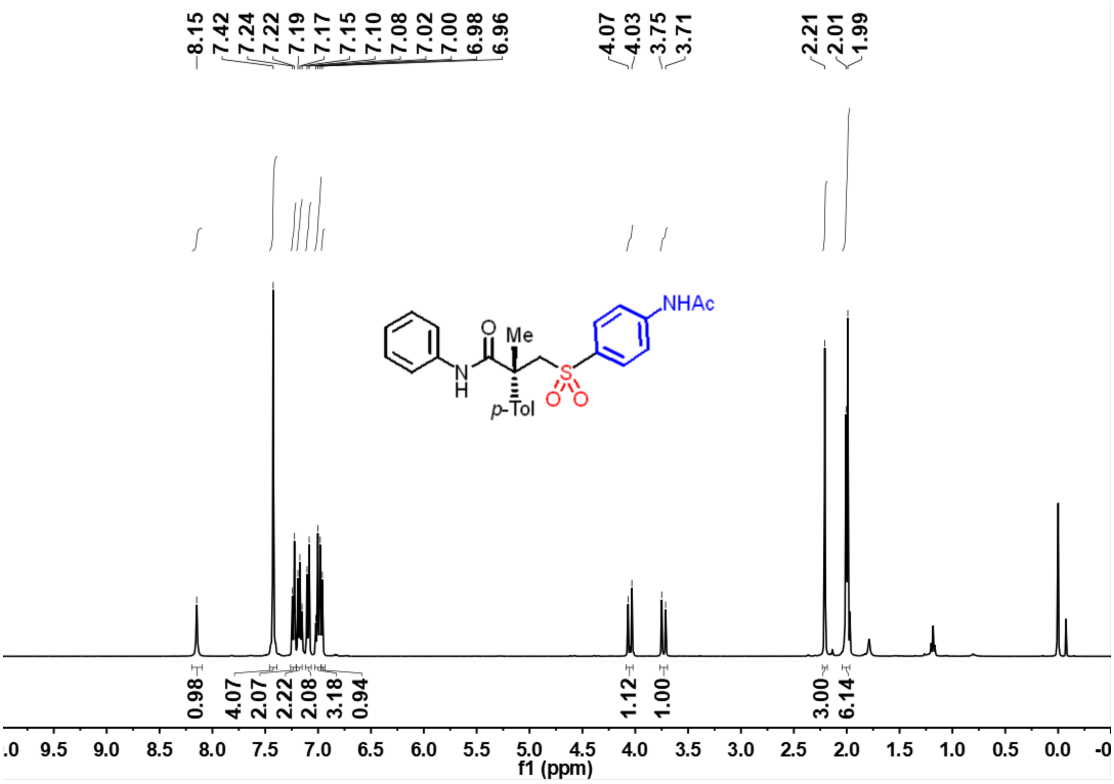

Supplementary Figure 119. ^{1}H NMR-spectrum of **4ae**, recorded at 400 MHz and 25 °C in $CDCl_3$

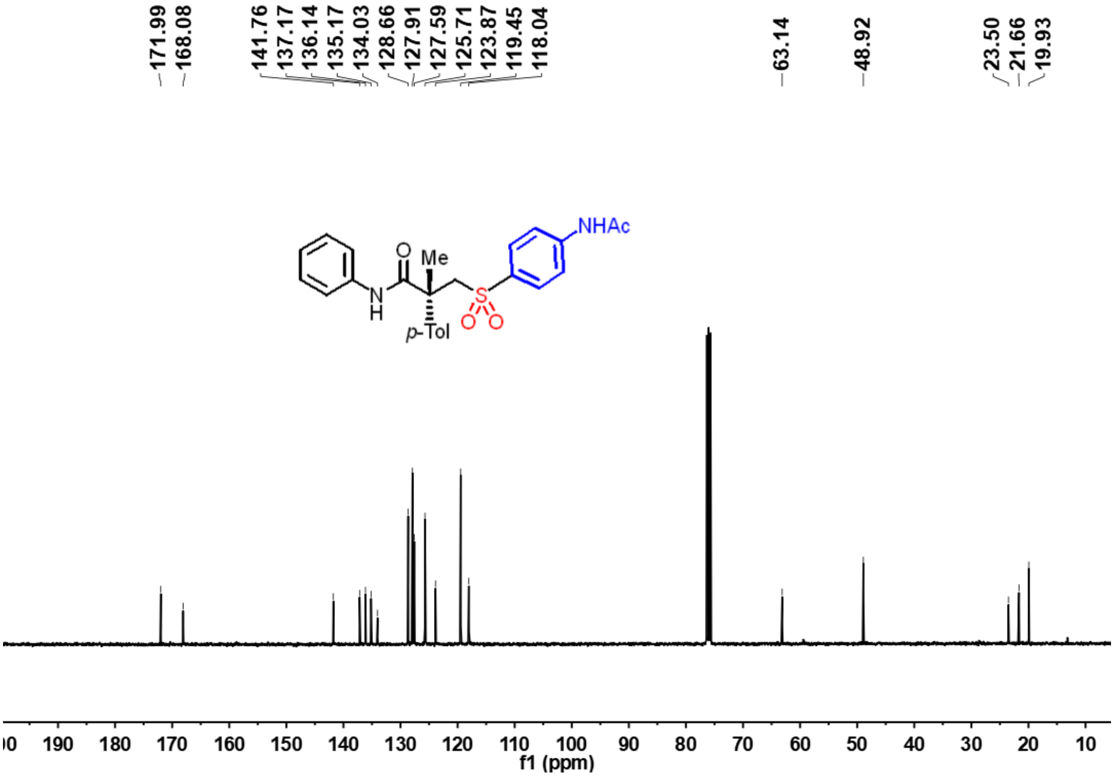

Supplementary Figure 120. ^{13}C NMR-spectrum of **4ae**, recorded at 400 MHz and 25 °C in $CDCl_3$

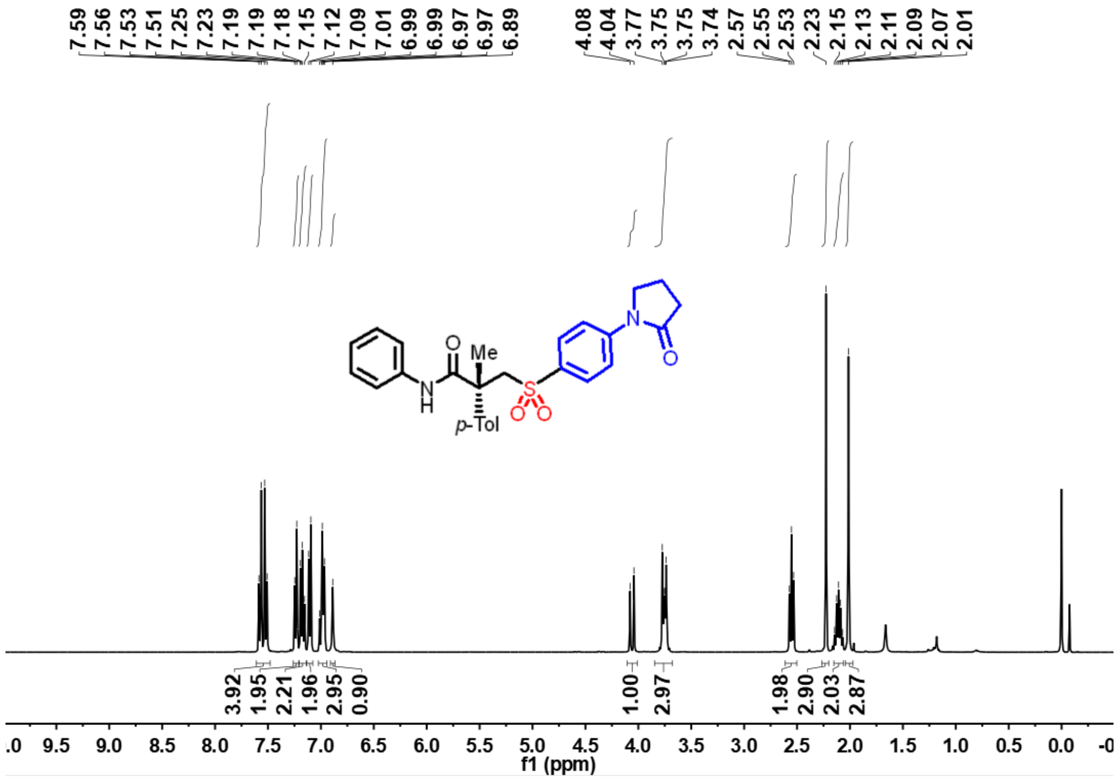

Supplementary Figure 121. ^{1}H NMR-spectrum of **4af**, recorded at 400 MHz and 25 °C in $CDCl_3$

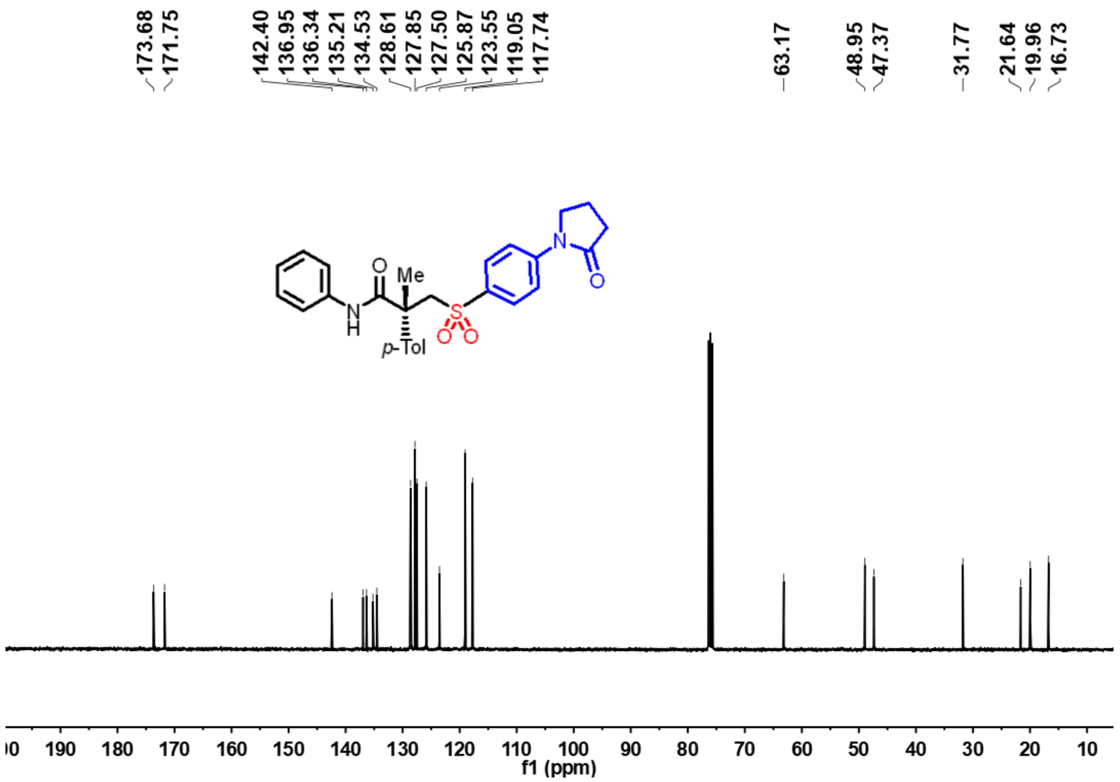

Supplementary Figure 122. ^{13}C NMR-spectrum of **4af**, recorded at 400 MHz and 25 °C in $CDCl_3$

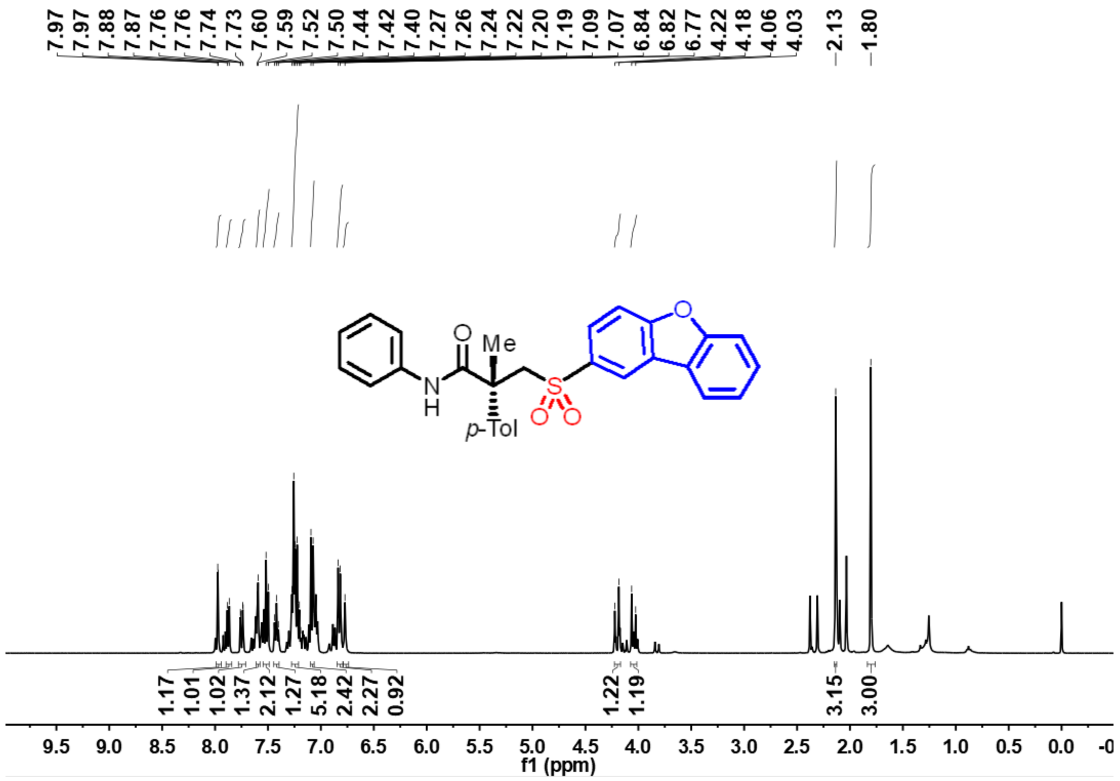

Supplementary Figure 123. ^{1}H NMR-spectrum of **4ag**, recorded at 400 MHz and 25 °C in $CDCl_3$

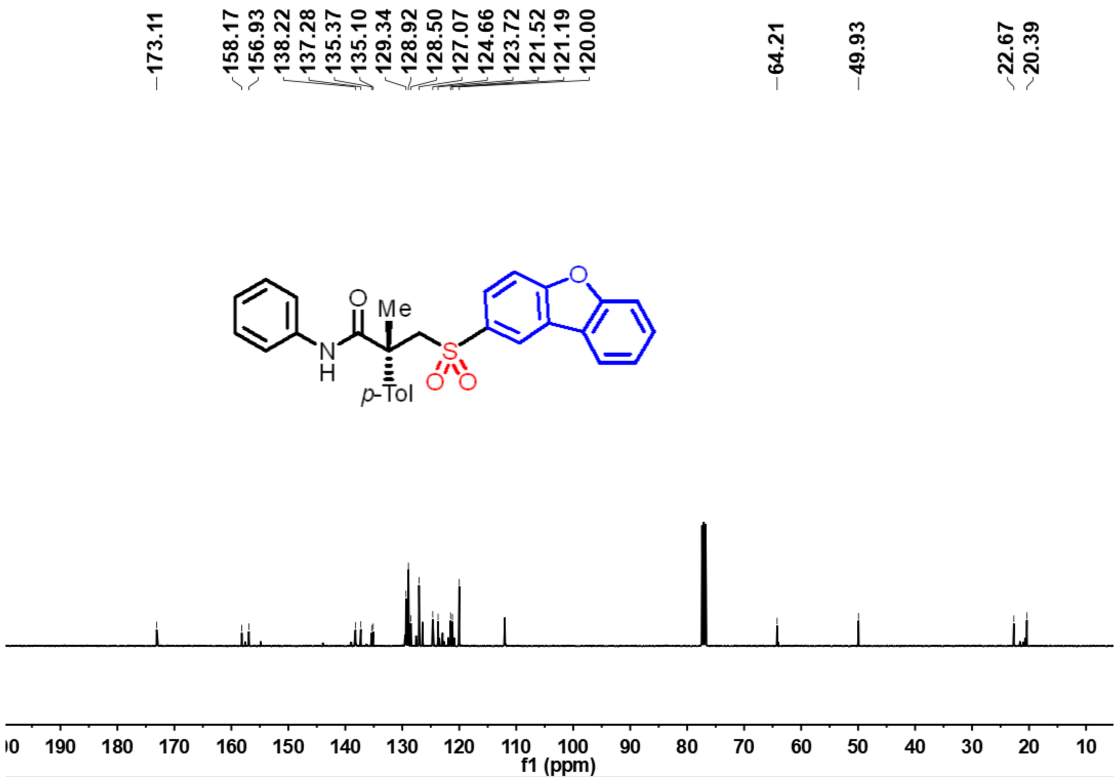

Supplementary Figure 124. ^{13}C NMR-spectrum of **4ag**, recorded at 400 MHz and 25 °C in $CDCl_3$

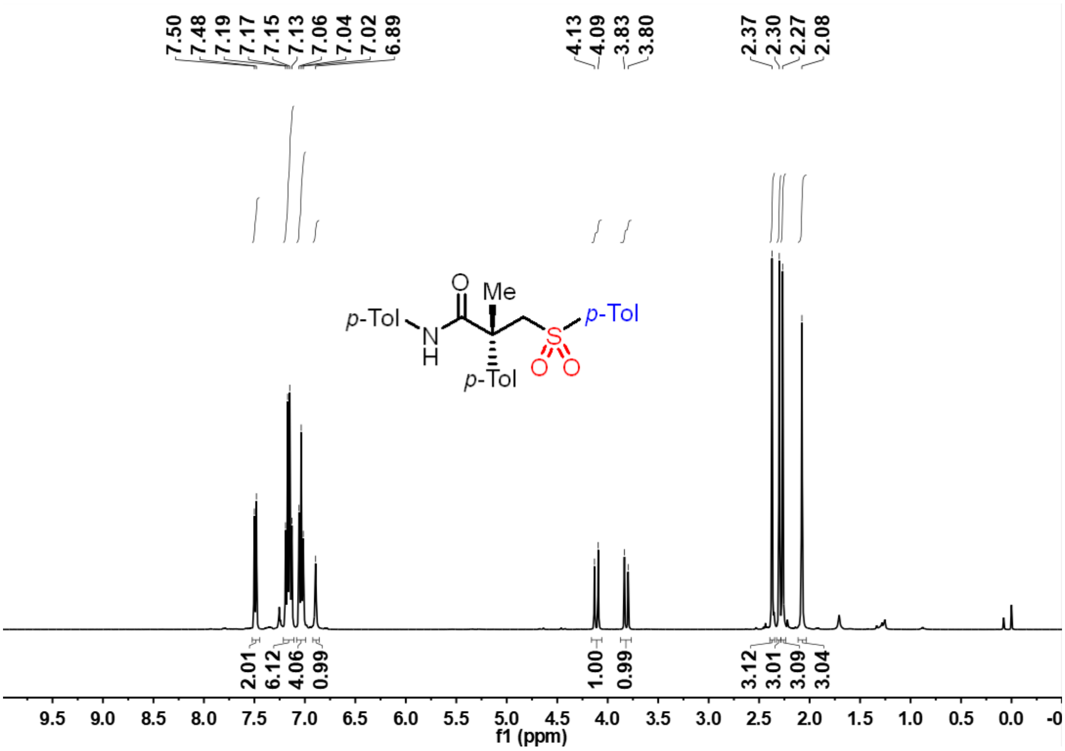

Supplementary Figure 125. ^{1}H NMR-spectrum of **4ah**, recorded at 400 MHz and 25 °C in $CDCl_3$

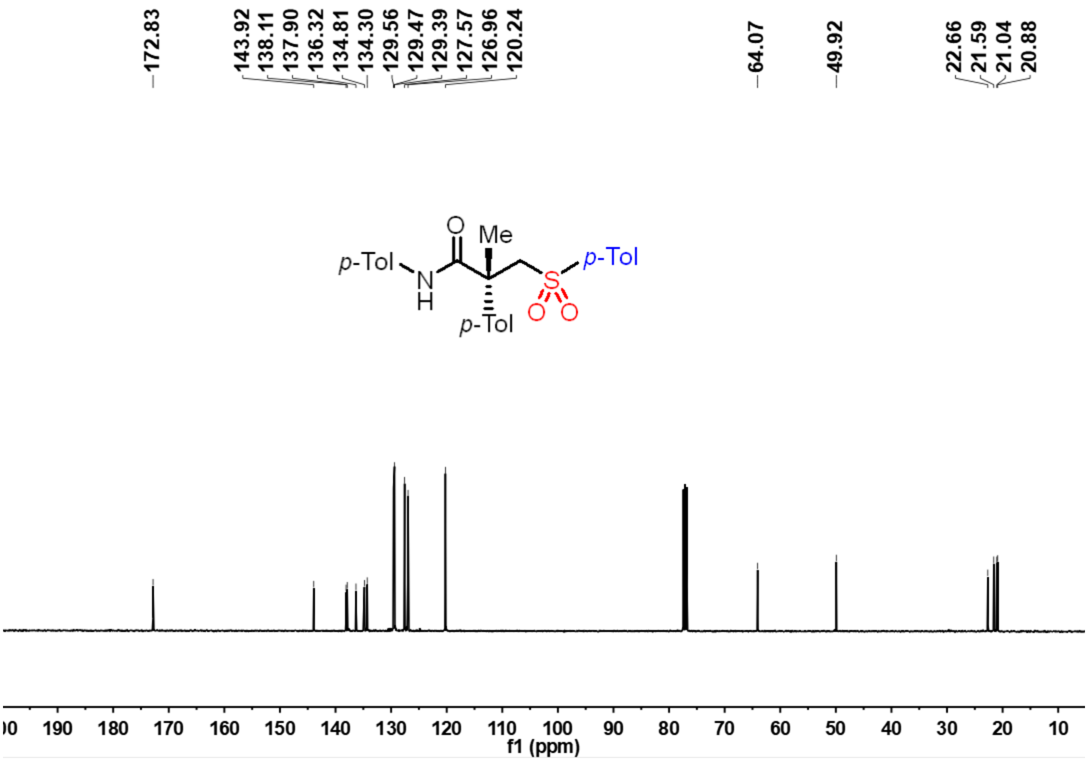

Supplementary Figure 126. ^{13}C NMR-spectrum of **4ah**, recorded at 400 MHz and 25 °C in $CDCl_3$

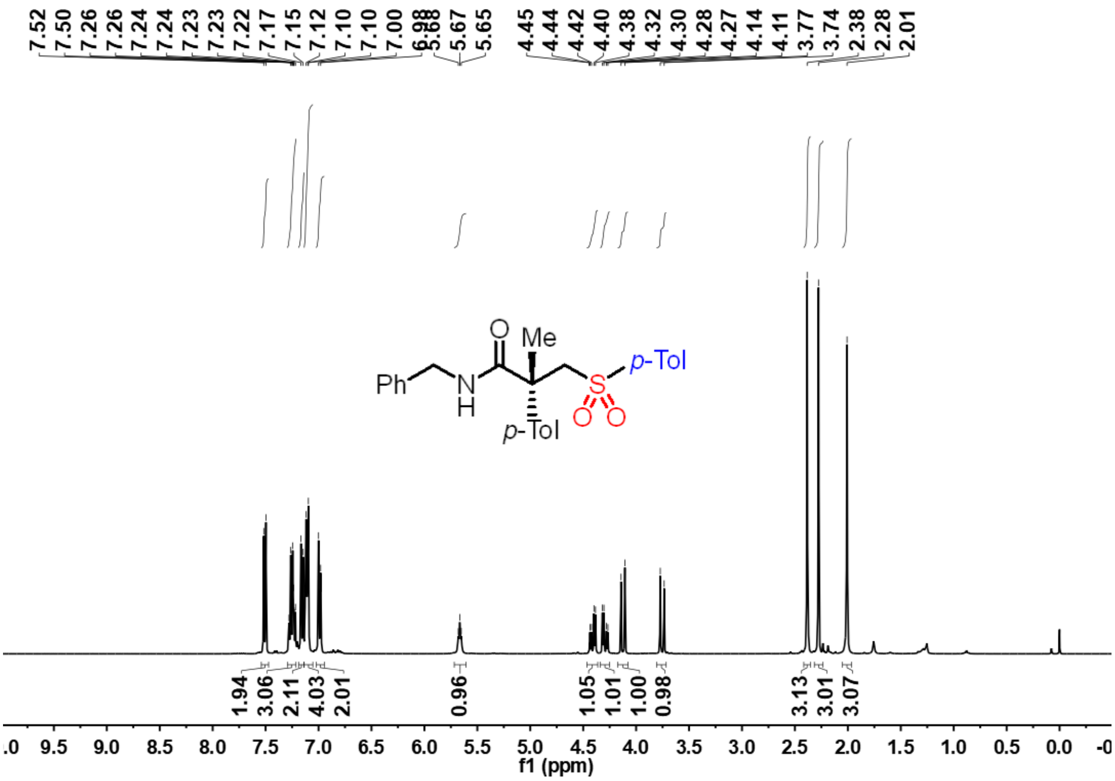

Supplementary Figure 127. ^{1}H NMR-spectrum of **4ai**, recorded at 400 MHz and 25 °C in $CDCl_3$

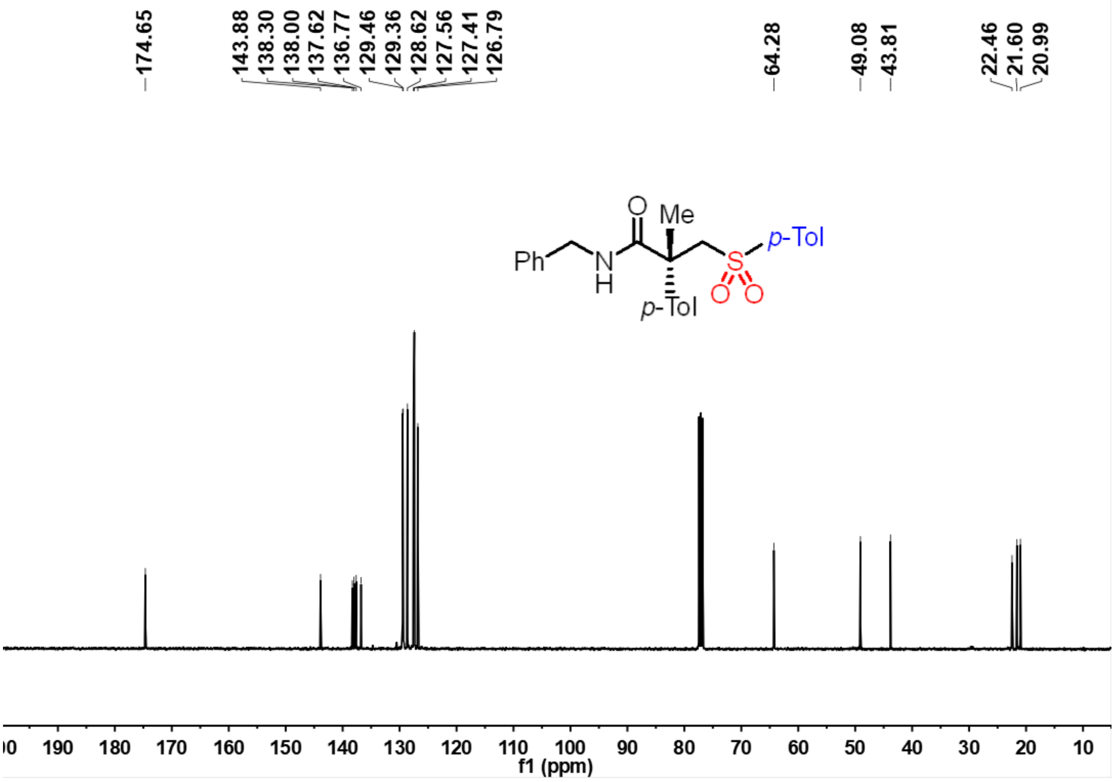

Supplementary Figure 128. ^{13}C NMR-spectrum of **4ai**, recorded at 400 MHz and 25 °C in $CDCl_3$

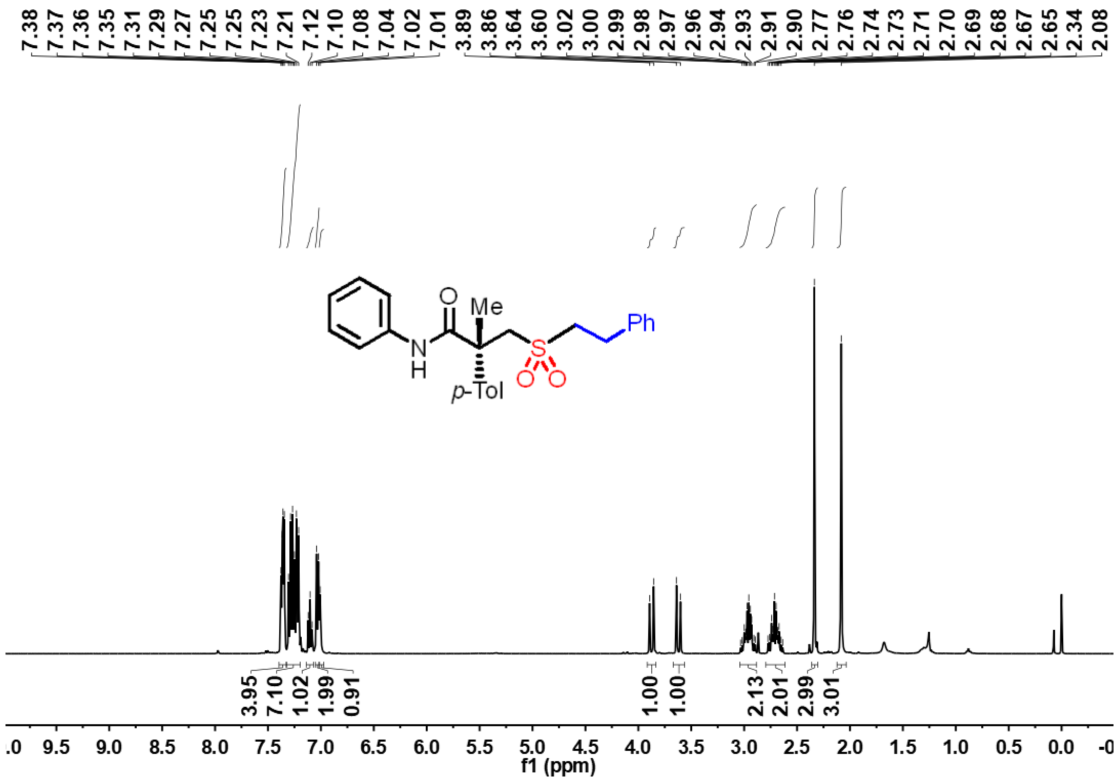

Supplementary Figure 129. ^{1}H NMR-spectrum of **4aj**, recorded at 400 MHz and 25 °C in $CDCl_3$

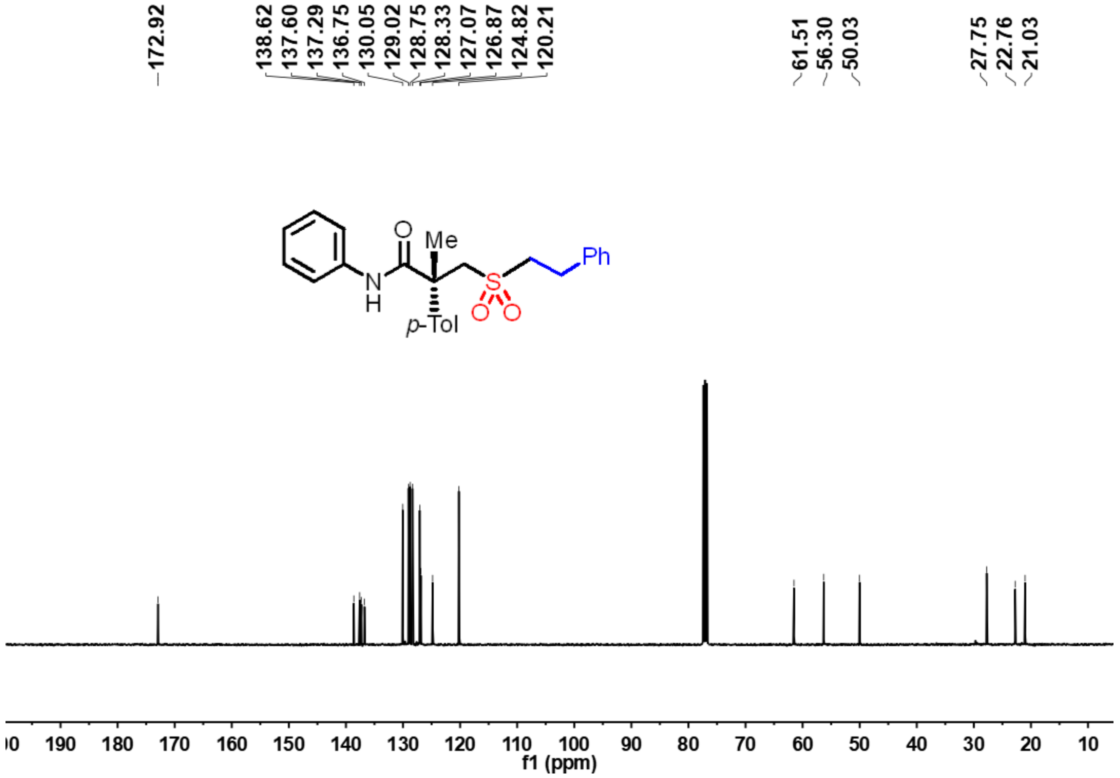

Supplementary Figure 130. ^{13}C NMR-spectrum of **4aj**, recorded at 400 MHz and 25 °C in $CDCl_3$

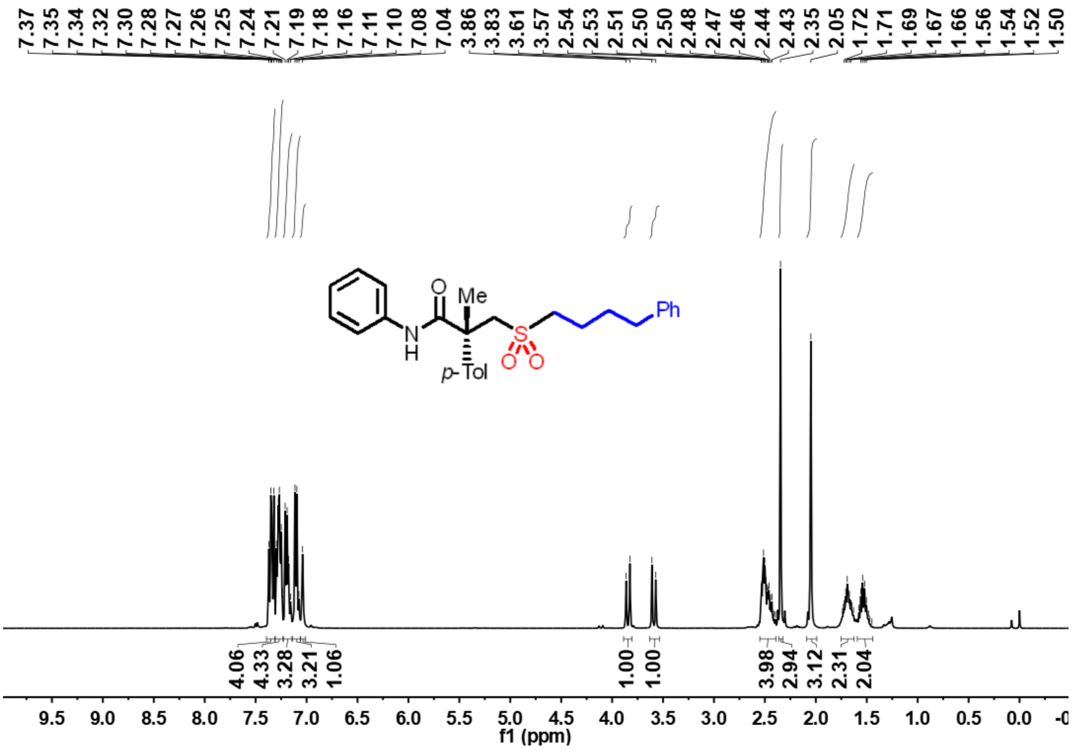

Supplementary Figure 131. ^{1}H NMR-spectrum of **4ak**, recorded at 400 MHz and 25 °C in $CDCl_3$

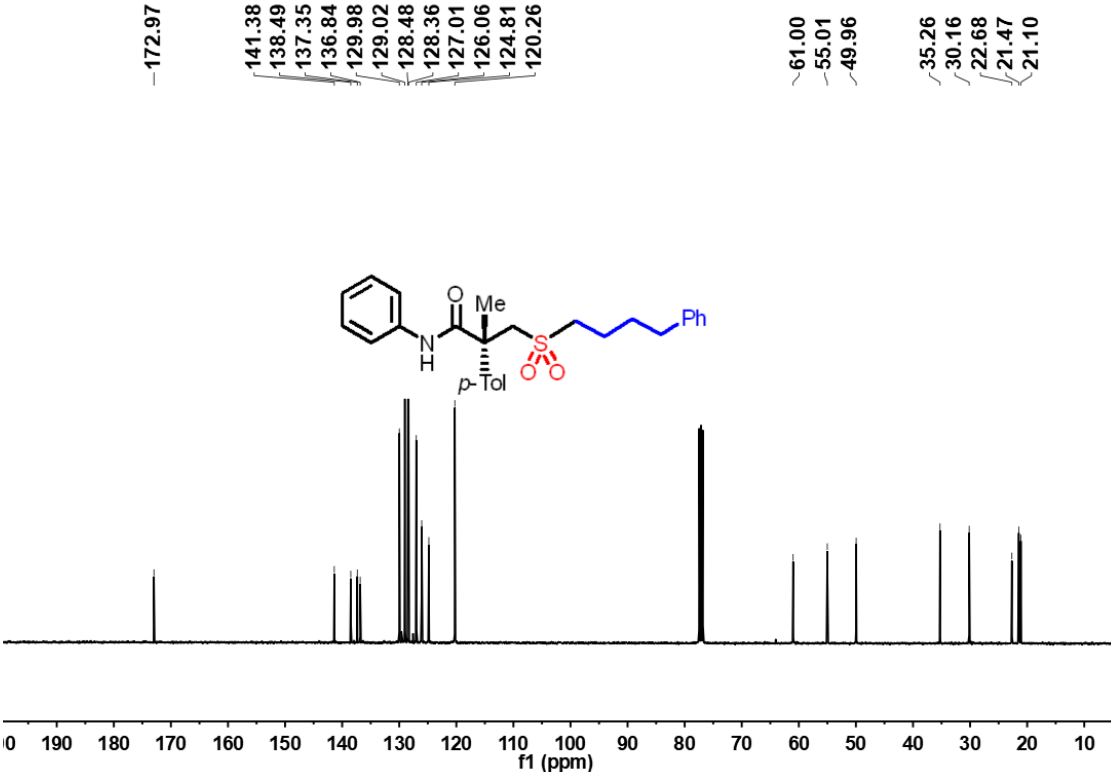

Supplementary Figure 132. ^{13}C NMR-spectrum of **4ak**, recorded at 400 MHz and 25 °C in $CDCl_3$

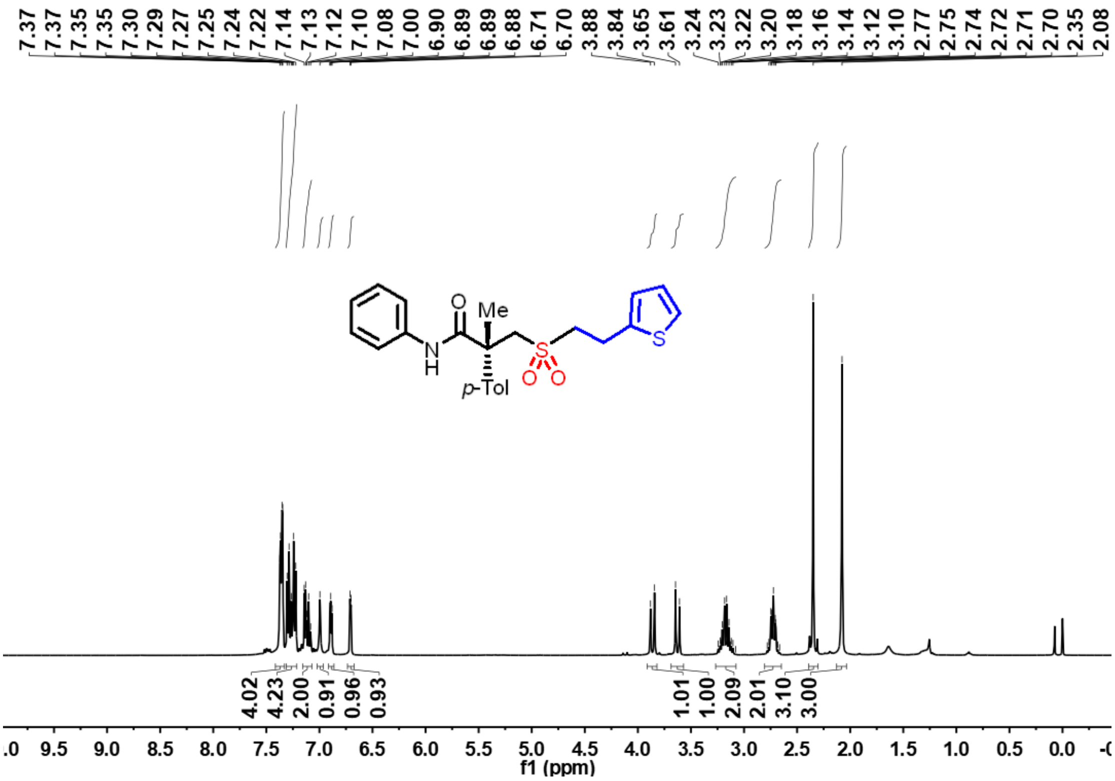

Supplementary Figure 133. ^{1}H NMR-spectrum of **4al**, recorded at 400 MHz and 25 °C in $CDCl_3$

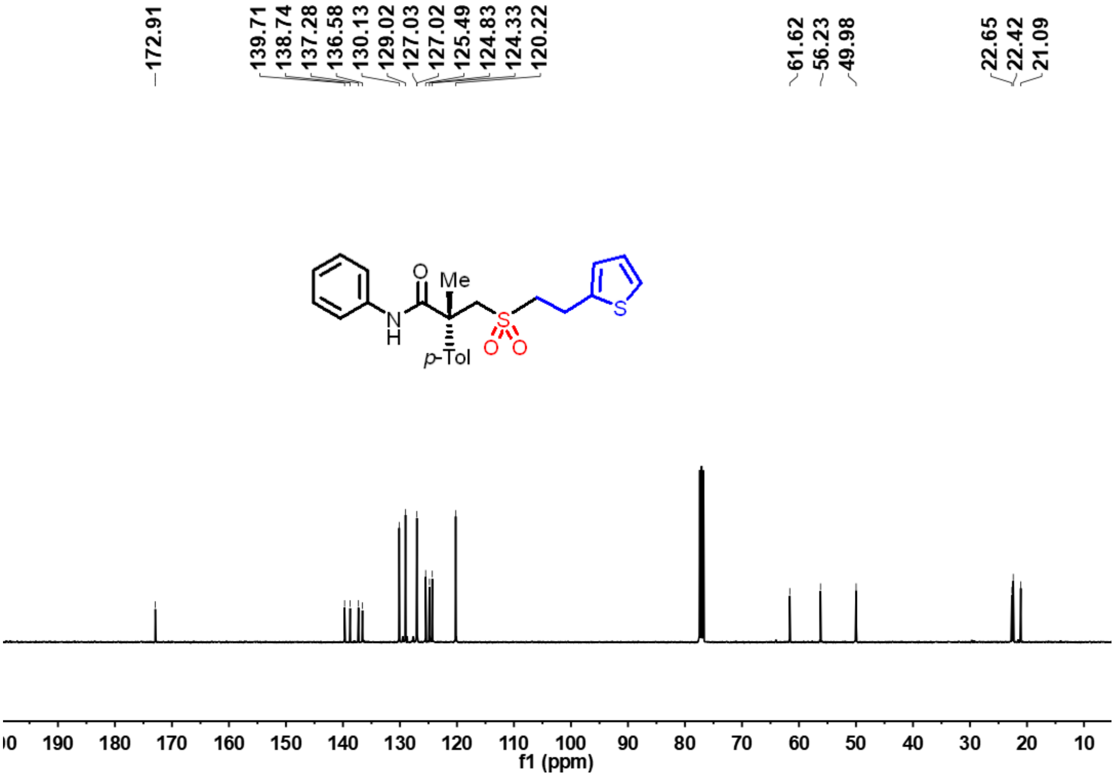

Supplementary Figure 134. ^{13}C NMR-spectrum of **4al**, recorded at 400 MHz and 25 °C in $CDCl_3$

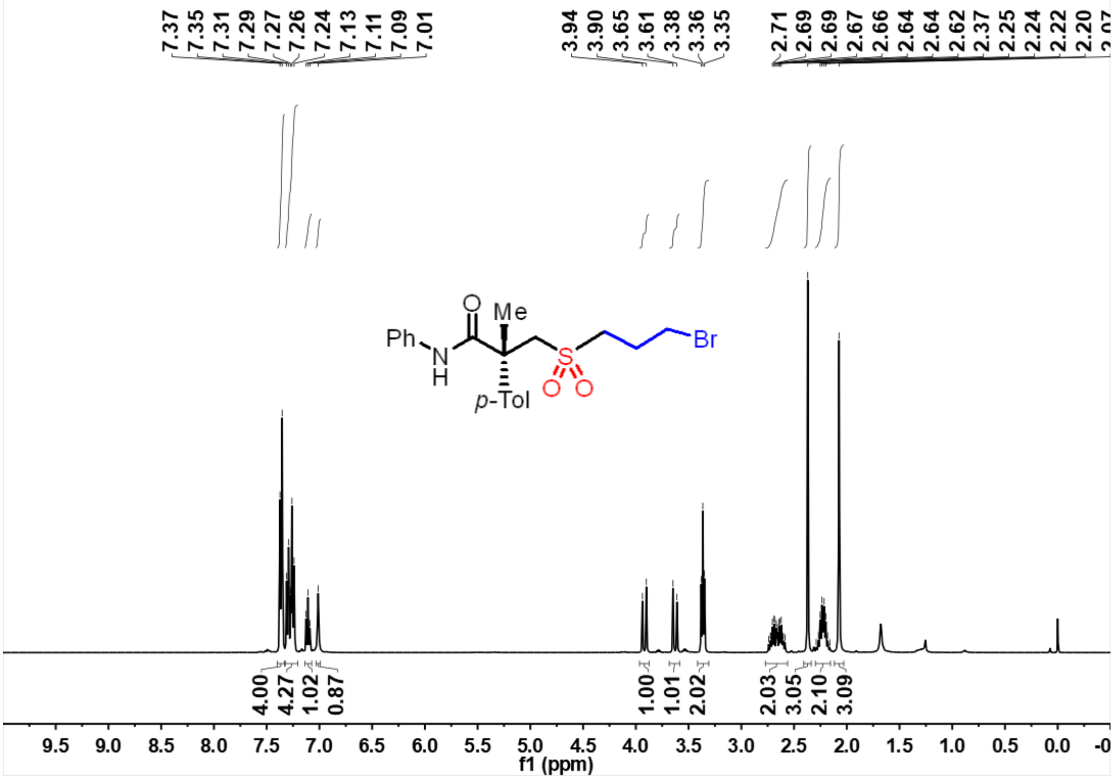

Supplementary Figure 135. ^{1}H NMR-spectrum of **4am**, recorded at 400 MHz and 25 °C in $CDCl_3$

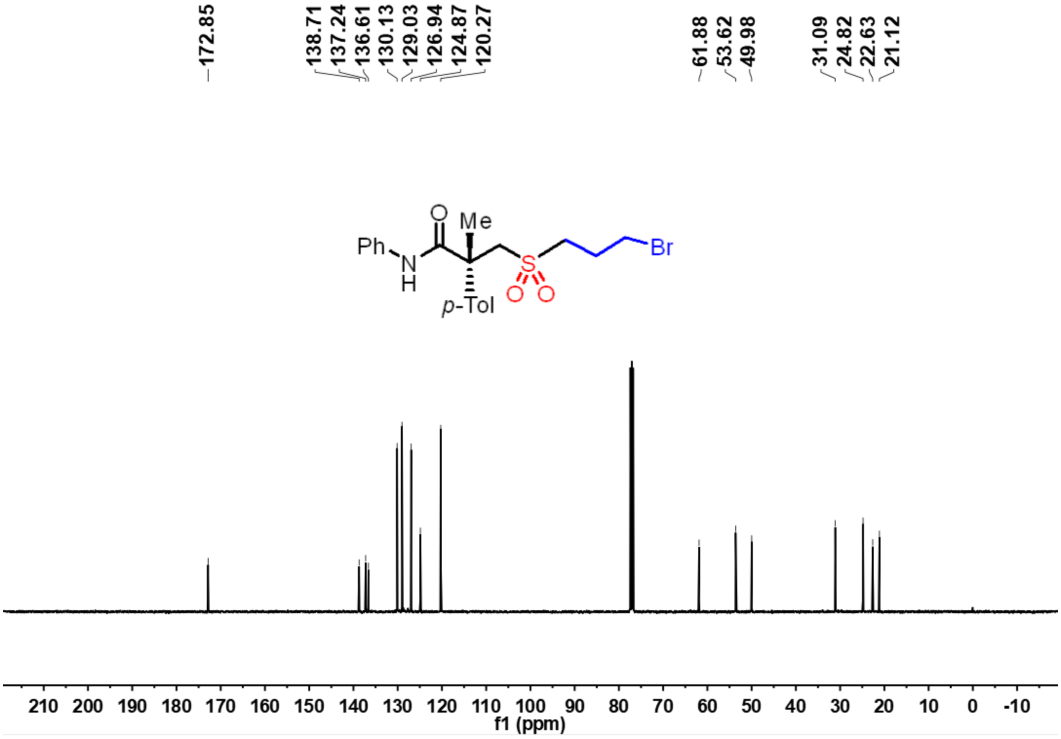

Supplementary Figure 136. ^{13}C NMR-spectrum of **4am**, recorded at 400 MHz and 25 °C in $CDCl_3$

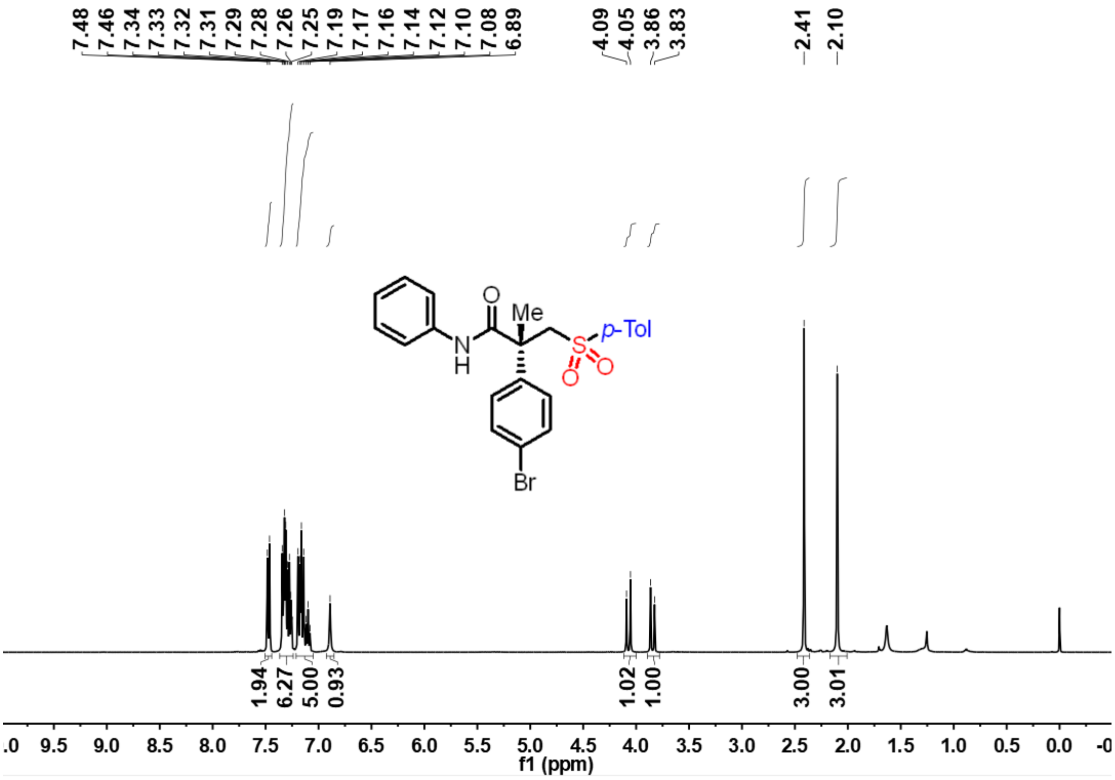

Supplementary Figure 137. ^{1}H NMR-spectrum of **4an**, recorded at 400 MHz and 25 °C in $CDCl_3$

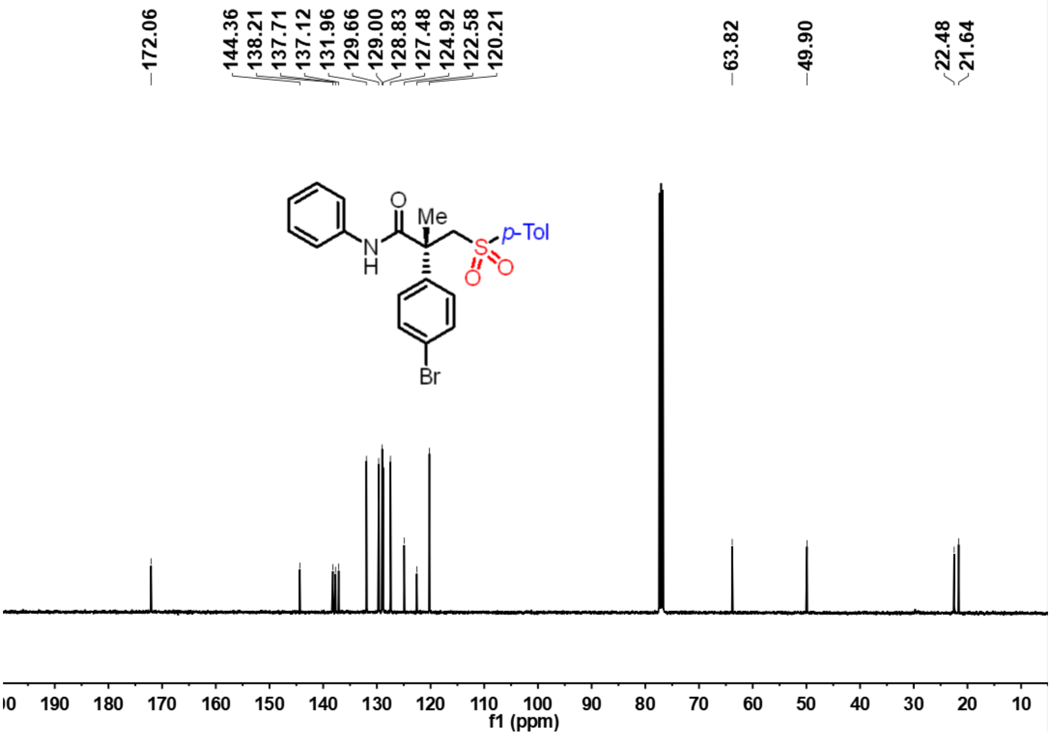

Supplementary Figure 138. ^{13}C NMR-spectrum of **4an**, recorded at 400 MHz and 25 °C in $CDCl_3$

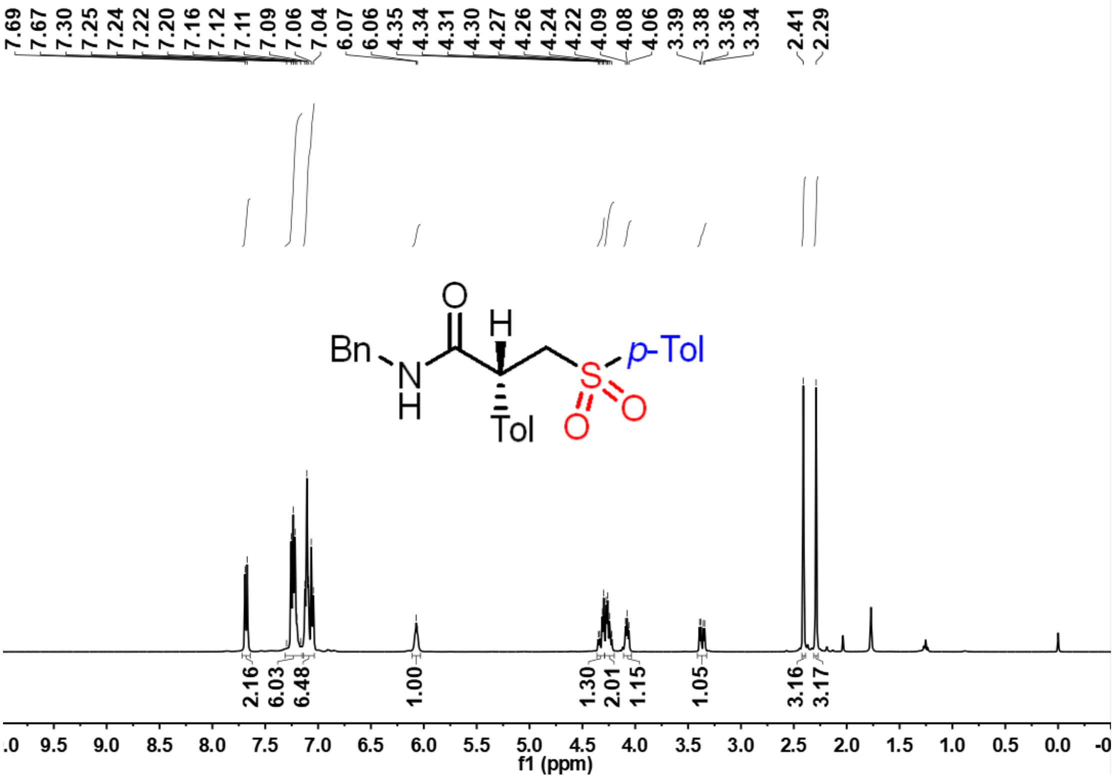

Supplementary Figure 139. ^{1}H NMR-spectrum of **4ao**, recorded at 400 MHz and 25 °C in $CDCl_3$

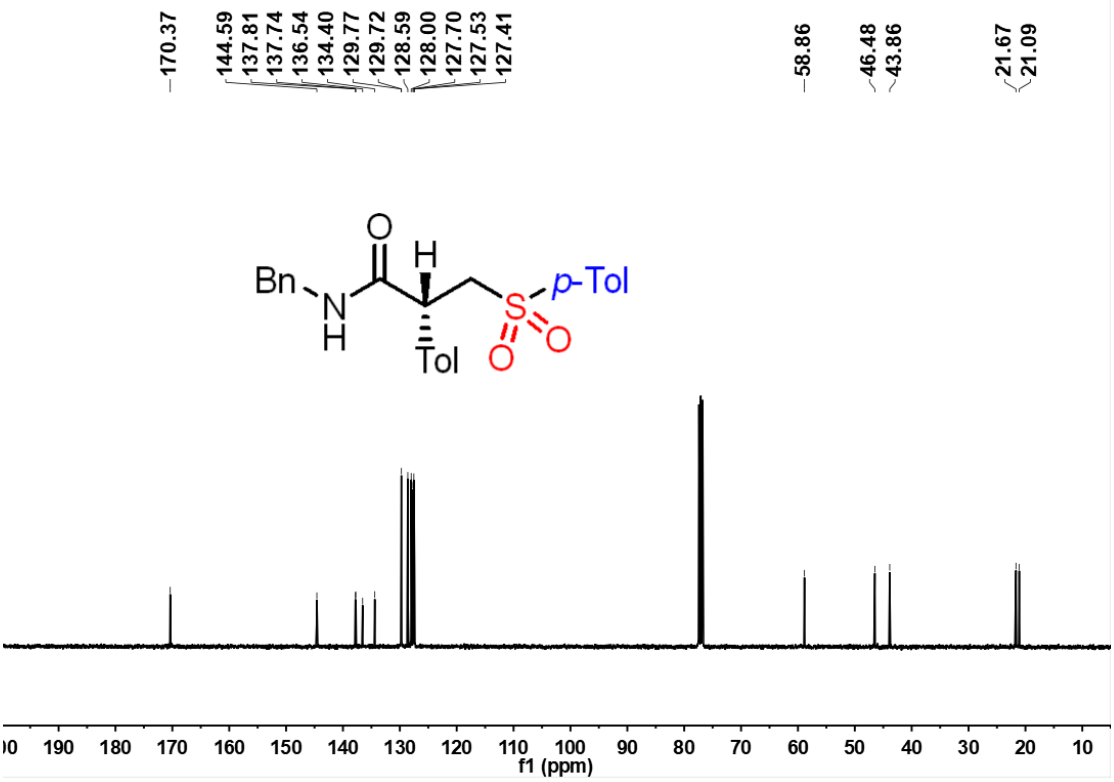

Supplementary Figure 140. ^{13}C NMR-spectrum of **4ao**, recorded at 400 MHz and 25 °C in $CDCl_3$

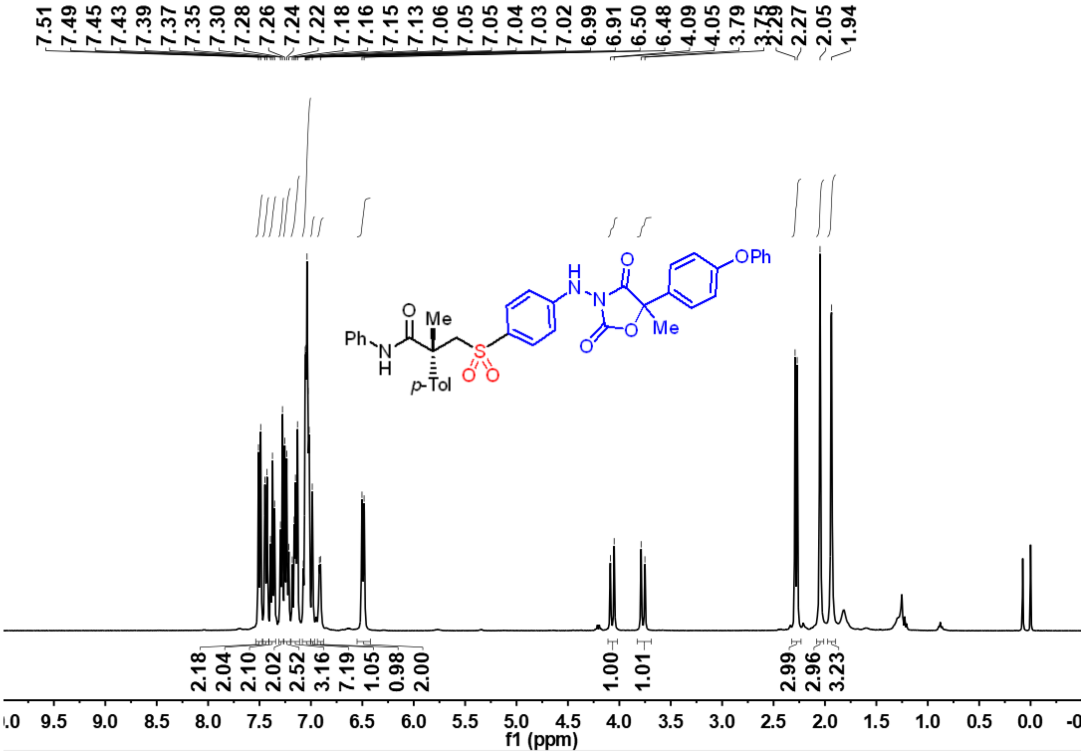

Supplementary Figure 141. ^{1}H NMR-spectrum of **4ap**, recorded at 400 MHz and 25 °C in $CDCl_3$

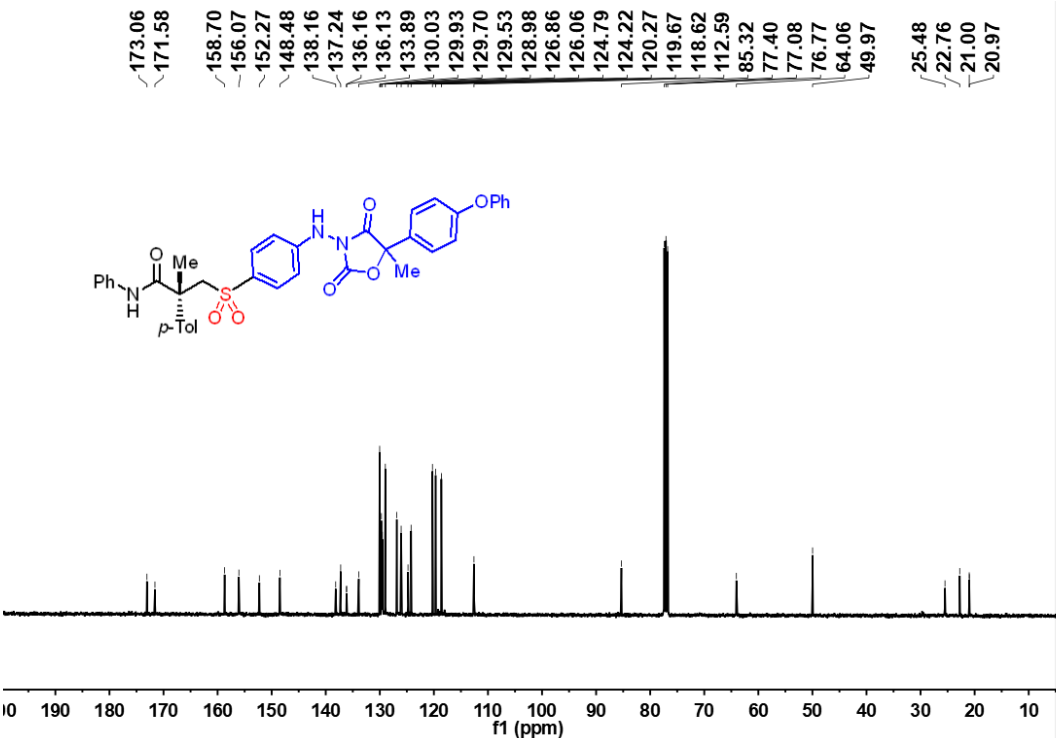

Supplementary Figure 142. ^{13}C NMR-spectrum of **4ap**, recorded at 400 MHz and 25 °C in $CDCl_3$

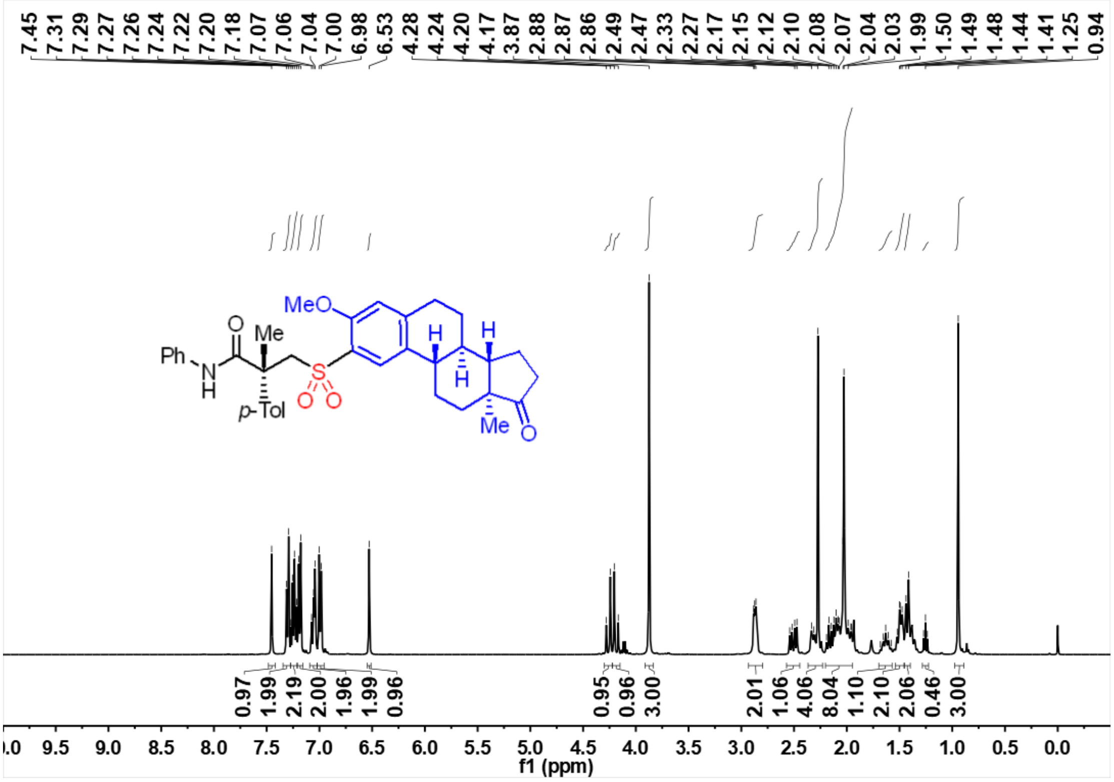

Supplementary Figure 143. ^{1}H NMR-spectrum of **4aq**, recorded at 400 MHz and 25 °C in $CDCl_3$

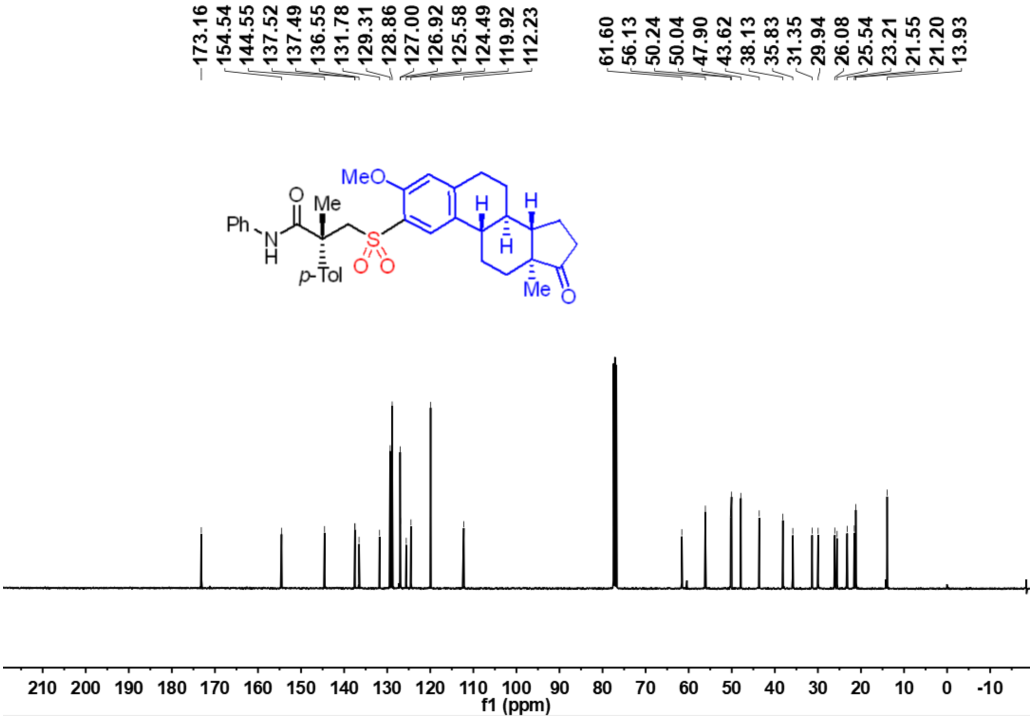

Supplementary Figure 144. ^{13}C NMR-spectrum of **4aq**, recorded at 400 MHz and 25 °C in $CDCl_3$

4 Supplementary References

1. Cismesia, M. A., & Yoon, T. P. *Chem. Sci.* **6**, 5426–5434 (2015).
2. Hervieu, C. et al. *Nat. Chem.* **13**, 327-334 (2021).
3. Kotha, S. & Khedkar, P. *Chem. Rev.* **112**, 1650-1680 (2012).
4. He, F.-S., Zhang, M., Zhang, M., Luo, X. & Wu, J. *Org. Chem. Front*. **8**, 3746-3751 (2021).
5. Li, Q., Huang, J., Cao, Z., Zhang, J. & Wu, J. *Org. Chem. Front*. **9**, 3781-3785 (2022).
6. Sheng, R.-L., Okada, K. & Sekiguchi, S. *J. Org. Chem*. **43**, 441-447 (1978).
